# Supplementary material for: Barriers to engagement in the care cascade for tuberculosis disease in India: A systematic review of quantitative studies
Source: PLoS Med. 2024 May 28;21(5):e1004409. doi: 10.1371/journal.pmed.1004409 (PMC11166313; doi:10.1371/journal.pmed.1004409)
Supplement: S2 Appendix — (PDF) [file pmed.1004409.s002.pdf]

S2 Appendix. Methods and study characteristics for the systematic review of barriers to completion of the TB diagnostic workup (Gap 2)

Supplement to:
Barriers to engagement in the care cascade for tuberculosis disease in India: a systematic review of quantitative studies

Authors:
Tulip A. Jhaveri, Disha Jhaveri, Amith Galivanche, Maya Lubeck-Schricker, Dominic Voehler, Mei Chung, Pruthu Thekkur, Vineet Chadha, Ruvandhi Nathavitharana, Ajay M.V. Kumar, Hemant Deepak Shewade, Katherine Powers, Kenneth H. Mayer, Jessica E. Haberer, Paul Bain, Madhukar Pai, Srinath Satyanarayana, Ramnath Subbaraman

Correspondence:
Ramnath Subbaraman, MD, MSc, FACP
Tufts University School of Medicine
Department of Public Health and Community Medicine
136 Harrison Ave., MV120
Boston, MA 02130, USA
Email: ramnath.subbaraman@tufts.edu

Table of Contents

***Methods* 3**

Objectives 3

Search strategy 4

Table A. Search strategy to identify manuscripts regarding non-completion of the diagnostic workup for TB patients in India (Gap 2). This same search was also used to identify relevant articles for Gaps 3 and 4. 5

Inclusion and exclusion criteria 5

Study selection 6

Fig A. PRISMA flowchart: study selection for the systematic review of non-completion of the diagnostic workup, pretreatment loss to follow-up, and on-treatment loss to follow-up for TB patients in India (Gaps 2, 3 and 4) 7

Quality assessment of quantitative studies 8

Table B. Criteria for assessing quality of quantitative studies evaluating failure to complete the diagnostic workup for TB patients in India 8

Data extraction and analysis 9

Table C. Characteristics of the included studies for individuals with presumptive tuberculosis who did not complete specific steps of the diagnostic workup (Gap 2) 10

Table D. Factors associated with non-completion of the tuberculosis (TB) diagnostic workup by individuals with presumptive TB or presumptive drug-resistant TB in India (Gap 2) 15

***References* 27**

Methods

Objectives

The objective of this systematic review was to understand into why some individuals with presumptive tuberculosis (TB) presenting to TB diagnostic facilities—government designated microscopy centers (DMCs) or private facilities—do not complete the appropriate diagnostic workup for TB (Gap 2 in the care cascade). We defined individuals with presumptive TB as people with cough >2 weeks or other symptoms that could be suggestive of active TB, as defined by the participant inclusion criteria used in each study. We defined individuals with presumptive drug-resistant TB (DR TB) as comprising individuals who met criteria for undergoing drug-susceptibility testing and other workup for DR TB in India's TB program, including being a person living with human immunodeficiency virus (PLHIV), having a prior history of TB treatment, or experiencing failure during treatment for drug-susceptible TB. The protocol was registered in PROSPERO in April 2020 under ID CRD42020159361.

Notably, Stage 2 of the care cascade comprises multiple steps in the diagnostic workup that vary depending on the type of presumed TB (e.g., smear-positive TB, smear-negative TB, DR TB, etc.). In general, for each type of presumed TB, a different diagnostic algorithm was used, usually culminating in the completion of a specific diagnostic test that would diagnose that form of TB. For example, sputum smear microscopy had to be completed to diagnose smear-positive TB; chest X-ray had to be completed to diagnose smear-negative TB; and nucleic acid amplification testing (NAAT, e.g., Xpert MTB/RIF testing, Truenat) or mycobacterial culture had to be completed to diagnose rifampin-resistant TB (RR TB) or multidrug-resistant TB (MDR TB), respectively. As such, an assumption of our approach to reporting findings is that non-completion of the diagnostic workup varies based on the specific diagnostic test required.

We therefore break down our reporting of Gap 2 findings as follows:

(a) *Non-pursual of diagnostic workup despite referral*: These studies evaluated patients recognized as having presumptive TB who were referred for, but did not pursue, any diagnostic workup.

(b) *Non-completion of sputum microscopy evaluation*: These studies evaluated non-completion of sputum microscopy testing, which comprised not completing submission of either two or three sputum samples, depending on the study year. Prior to April 2009, the workup for TB in India started with collection of three sputum samples. After April 2009, the workup for TB in India started with collection of only two sputum samples.

(c) *Non-completion of chest X-ray*: Nearly all of these studies related to the diagnostic workup for presumed smear-negative TB. While the recommended diagnostic algorithm for presumptive smear-negative TB evolved over time, all such algorithms culminated in the need to complete a chest X-ray to confirm the diagnosis. In addition, one recent study evaluated non-completion of chest X-ray after 2017, when the diagnostic algorithm in India's National TB Elimination Program (NTEP) was changed such that all individuals with presumptive TB were supposed to undergo chest X-ray. Given that the availability of chest X-ray facilities (rather than the specific diagnostic algorithm) seemed to drive non-completion, we compare findings across all of these studies that evaluated non-completion of chest X-ray, regardless of the diagnostic algorithm used.

(d) *Non-completion of NAAT, line probe assay, or mycobacterial culture*: Several studies evaluated non-completion of the diagnostic algorithm for presumed DR TB or RR TB. While the recommended diagnostic algorithm for DR TB evolved over time, with the exception a few papers that evaluated completion of mycobacterial culture or line probe assay, most such algorithms culminated in completion of NAAT testing for the papers included in our review. In addition, one recent study evaluated non-completion of NAAT after 2017, when the diagnostic algorithm in India's National TB Elimination Program (NTEP) was changed such that patients with positive sputum microscopy tests, chest X-ray evidence of TB, or who were PLHIV were supposed to undergo NAAT. Again, given that the availability of the testing modality seemed to drive non-completion of this workup, we compare findings across all of these studies that evaluated non-completion of NAAT, line probe assay, or mycobacterial culture, regardless of the diagnostic algorithm used.

We extracted three types of quantitative data that help to understand non-completion of the TB diagnostic workup:

(a) *Factors associated with not having completed a specific diagnostic test*: For studies comparing individuals who had or had not completed a specific diagnostic test (e.g., sputum microscopy, chest X-ray, or NAAT), we extracted effect estimates for variables associated with non-completion. Effect estimates included odds ratios, risk ratios, hazard ratios, or beta-coefficients, depending on the approach to analysis.

(b) *Reasons reported by patients for non-completion of the diagnostic workup*: For studies that surveyed patients who had not completed a specific diagnostic test, we extracted the proportion of individuals who reported a given reason for not completing the workup. *Notably, the denominator for these analyses was the number of patients who did not complete a specific workup. The numerator in these analyses was the number of patients who reported a given reason for not completing the workup.*

(c) *Reasons from health system records for non-completion of the diagnostic workup*: For individuals with presumptive DR or RR TB, multiple studies extracted data from medical records that describe health system-related reasons for non-completion of tests (e.g., healthcare providers not recognizing patients had presumptive DR TB, misplacement of sputum samples during transportation to a testing center, etc.). Notably, these analyses reported findings differently than studies that directly interviewed patients who had not completed the diagnostic workup. *Specifically, the denominator in these analyses was the overall number of patients with presumptive DR TB—rather than the number of patients who did not complete the diagnostic workup. The numerator in these analyses was the number of patients who experienced a specific health system problem contributing to non-completion of the diagnostic workup.*

Search strategy

Three separate searches were conducted to identify articles. The first search was conducted as part of a previously published study quantifying gaps in India's TB care cascade [2]. We used articles identified for that review that evaluated Gap 2 in the TB care cascade but that also reported factors and reasons associated with not completing the TB diagnostic workup. For that review, a medical librarian searched PubMed, Embase, and Web of Science for studies published between January 1, 2000 and February 26, 2015, without language restrictions, using

search terms and related variants for “tuberculosis”, “India”, and “loss to follow-up”, including “pretreatment loss to follow-up” and “initial default” to include early losses preceding treatment initiation, including during the diagnostic workup (Table A). We also carried out electronic searches of key Indian journals that were not indexed for that entire time window: the Indian Journal of Tuberculosis, Lung India, the Indian Journal of Chest and Allied Sciences, the India Journal of Public Health, and the Indian Journal of Community Medicine. Additional studies were identified by searching reference lists of the primary studies and relevant review articles. Notably, given similarities in the search terms for identifying patient losses during the diagnostic workup (Gap 2), pretreatment loss to follow-up (Gap 3), and poor outcomes during treatment (Gap 4), this single search was used to identify studies related to all of these gaps. We screened all identified studies from this previous review for potential inclusion in our current review; however, different data were extracted from studies that met our inclusion criteria.

To update our review, we conducted a second refresher search using the same search terms for October 2, 2015 to October 1, 2019. We did not repeat hand searches of the Indian journals listed above, because all of these journals had been indexed in PubMed prior to the time period of this more recent search. Due to the extensive time required to extract data from the articles identified for this systematic review, we performed a third refresher search using the same search terms for October 2, 2019 to August 14, 2023. Finally, additional studies were identified by looking through the reference lists of the included primary studies and relevant review articles that were identified by the searches and by outreach to experts in the field.

Table A. Search strategy to identify manuscripts regarding non-completion of the diagnostic workup for individuals with presumptive tuberculosis (TB) in India (Gap 2). This same search was also used to identify relevant articles for Gaps 3 and 4.

Terms for tuberculosis:	“tuberculosis”[Mesh] OR *Mycobacterium tuberculosis*[tiab] OR TB[tiab] OR MDRTB[tiab] OR XDRTB[tiab]
Terms for India:	“India”[Mesh] OR India[tiab] OR India[ad] OR Indian[tiab] OR Indians[tiab]
Terms for loss to follow-up or other poor outcomes:	“patient dropouts”[tiab] OR “treatment refusal”[Mesh] OR “patient compliance”[Mesh] OR lost to follow up[tiab] OR loss to follow up[tiab] OR default*[tiab] OR compliance[tiab] OR adherence[tiab] OR noncompliance[tiab] OR nonadherence[tiab] OR diagnostic dropout [tiab] OR patient cooperation[tiab] OR dropout*[tiab] OR linkage to care[tiab] OR retention[tiab] OR attrition[tiab] OR cascade of care[tiab] OR treatment cascade[tiab] OR treatment success*[tiab] OR treatment completion[tiab] OR cure[tiab] OR pretreatment loss to follow-up[tiab] OR initial default[tiab]; treatment failure [tiab]

Inclusion and exclusion criteria

We applied the following criteria for inclusion and exclusion of studies for this systematic review.

Inclusion criteria included the following:

(1) Studies that followed individuals who were recognized by healthcare providers as having presumptive TB and then referred for diagnostic evaluation. As described above, depending on the patient population being studied, these studies may have followed patients to see if they pursued TB diagnostic workup after initial referral, completed sputum microscopy evaluation, completed chest X-ray, or complete NAAT, line probe assay, or mycobacterial culture.
(2) Studies also had to have assessed reasons that individuals in the study may not have completed the diagnostic workup, by comparing characteristics of those who did or did not complete the workup (e.g., regression analyses) or by follow-up structured interviews with patients who did not complete the workup.

Exclusion criteria included the following:

(1) Studies that only described the proportion of TB patients who did not complete a given diagnostic workup, without evaluating reasons for non-completion.
(2) Studies with data collected prior to the year 2000, as India's Revised National TB Control Programme (now called the National TB Elimination Programme) did not achieve nationwide coverage until the early 2000s.
(3) Studies only containing qualitative data evaluating why individuals did not complete the TB diagnostic workup. Findings from studies containing qualitative data will be reported in a separate paper.

Study selection

Each citation identified by the search was independently assessed by at least two reviewers (among TJ, DJ, AG, DV, MLS and KP) for their eligibility at the title and abstract evaluation stage and again subsequently at the full text evaluation stage (Fig A). Disagreements between the two reviewers were resolved by discussion or, if necessary, through consultation of a third reviewer (RS). Independent selection of articles at the title and abstract and full text stages was conducted using Covidence software (Veritas Health Innovations, Melbourne, Australia); however, quality assessment and extraction of study findings was conducted using an Excel spreadsheet.

Fig A. PRISMA flowchart: study selection for the systematic review of non-completion of the diagnostic workup, pretreatment loss to follow-up, and on-treatment loss to follow-up for people with tuberculosis disease in India (Gaps 2, 3 and 4)

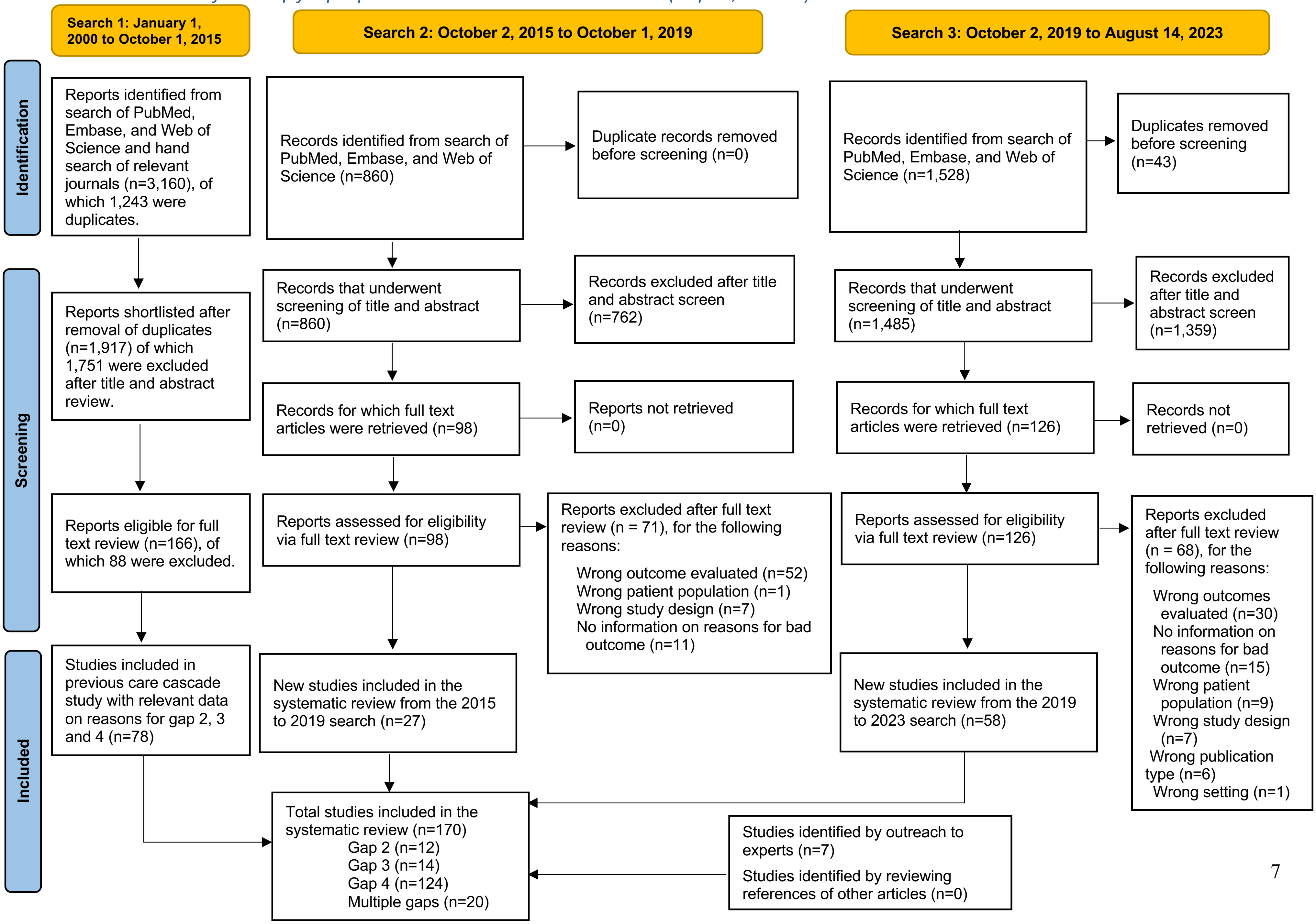

Quality assessment of quantitative studies

In our previous systematic review, we developed quality criteria relevant to studies focused on identifying patients who did not complete the TB diagnostic workup (Table B), because there were no existing standardized guidelines for assessing the quality of these types of studies. Although we extracted different data from these studies in the current systematic review, we used the same quality criteria for studies that included relevant quantitative information, because these criteria are still appropriate for assessing the overall quality of these types of observational studies.

We classified the health facility-based strategy for sampling patients in each study based on whether it was comprehensive, random, or convenience. Studies using convenience sampling were excluded from analysis. Studies that assessed more than 150 patients and at over one government or private sector diagnostic facility were rated as being higher in quality than studies that assessed less than 150 patients at a single diagnostic facility. Studies that assessed outcomes within a shorter time frame after the start of diagnostic workup (e.g., within 1-2 months) were rated as being higher in quality than studies that assessed these indicators 3 or more months after the start of diagnostic workup, since tracking patients to find out accurate outcomes becomes much harder with time due to patient mobility. Specifically, as the time increases between the start of patient's diagnostic evaluation and follow-up of the patient's outcome by a study team, there is a higher likelihood of misclassifying a patient's outcome as "non-completion of diagnostic workup" simply because it is not possible to find the patient because they have moved, obtained a new cell phone, etc. For the same reason, studies in which a dedicated research team was used to track patients were rated as being higher in quality than those relying on self-report by local TB programs to evaluate non-completion of the diagnostic workup.

Table B. Criteria for assessing quality of quantitative studies evaluating failure to complete the diagnostic workup for individuals with presumptive tuberculosis (TB) in India

**Criterion**	**Quality level**
**Sampling strategy**	
Random (i.e., probability-based) or comprehensive sampling at selected TB diagnostic facilities	High
Convenience sampling or not reported	Low (exclude from analysis of findings)
**Sample size**	
>1 diagnostic center and 150+ patients	High
Single diagnostic center study with 150+ patients	Medium
<150 patients or not reported	Low
**Time frame of research fieldwork after start of diagnostic evaluation**	
2 weeks to 1 month after start of diagnostic evaluation	High
1-3 months after start of diagnostic evaluation	Medium
>3 months after start of diagnostic evaluation or not reported	Low
**Method of evaluating outcome**	
Patient tracking by a dedicated research team	High
Relying on self-report by government TB program or not reported	Medium to Low

Data extraction and analysis

Multiple reviewers (TJ, AG, DV, MLS, and DJ) independently extracted data from each included study into a structured form on an Excel spreadsheet; however, we ensured that every article had data independently extracted by at least two or more reviewers. Disagreements were resolved by discussion or, if necessary, by consulting a third reviewer (RS). For each study, we extracted information on the study design, location, setting (i.e., urban versus rural), sample size, and variables of interest (Table C).

For studies that compared individuals who had completed a specific part of the TB diagnostic workup against those who had not completed that part of the workup, we extracted adjusted and unadjusted effect estimates (odds ratios, risk ratios, hazard ratios, and beta-coefficients) from regression analyses. For studies that did not report effect estimates, we calculated unadjusted odds ratios from the data provided, if possible. For studies that reported reasons why individuals had not completed part of the diagnostic workup, we extracted the proportion of individuals surveyed who reported a given reason for noncompletion or the proportion of patients or patients samples who did not complete part of the workup due to a specific health system problem (e.g., misplaced or untested sputum samples). For effect estimates and proportions, we extracted information on 95% confidence intervals (95% CIs) where available; if 95% CIs were not reported, we calculated these from the data provided, if possible.

For the regression analyses, some studies reported factors associated with having completed part of the TB diagnostic workup, while others reported factors associated with not having completed part of the diagnostic workup. For consistency, we “flipped” effect estimates and 95% CIs as needed so that all findings represented associations with the outcome of not having completed the diagnostic workup. For some variables, we also changed the reference group as needed for consistency of reporting across studies. For example, because most studies compared men to the reference group of women, we “flipped” effect estimates and confidence intervals for studies that presented men as the reference group. This allowed us to consistently present women as the reference group for findings regarding gender.

After “flipping” selected effect estimates from the regression analyses, we reported all unadjusted and adjusted effect estimates, regardless of statistical significance, organized by study (Table D). For the main manuscript and Forest plot, we restricted ourselves to presenting statistically significant adjusted effect estimates from multivariable analyses, as these may represent more meaningful associations from higher-quality analyses. After extracting this subset of findings, we organized findings into categories using our framework of demand- and supply-side factors (main manuscript, Table 1). For findings on reasons why individuals had not completed part of the TB diagnostic workup, we presented all reported proportions in the Forest Plots in the main manuscript; we also organized these findings using our framework of demand- and supply-side categories.

To visualize quantitative findings, we generated Forest plots of effect estimates odds ratios, risk ratios, hazard ratios, beta-coefficients, and proportions using Stata version 16.1 (College Station, TX, USA). As described earlier, we present these Forest plots organized by the specific type of diagnostic modality that was not completed (i.e., noncompletion of sputum microscopy, chest X-ray, or NAAT). We did not conduct meta-analyses of data, because we extracted findings representing a diverse set of variables from every study.

Table C. Characteristics of the included studies for individuals with presumptive tuberculosis (TB) who did not complete specific steps of the diagnostic workup (Gap 2)

**Citation** (year)	**Location**	**Urban, rural, both, or unknown**	**Type of population**	**Single or multiple TB diagnostic centers**	**Number of overall individuals with presumptive TB followed in the study**	**Time frame of research follow-up after initial patient evaluation**	**Methodology** (Patient tracking by a dedicated research team versus self-report by the government TB program)	**Type of findings included in the study** (N=sample or denominator of patients for given analysis)
**Non-pursual of diagnostic workup despite referral**								
Dey (2019)[3]	West Bengal	Urban	Individuals with symptoms identified via active case finding in high TB burden wards	Multiple	1,132	0-4 weeks	Patient tracking by dedicated research team	Relative risk regression (N=1,132)
Garg (2020) [4]	Bihar	Rural	Individuals with symptoms identified via active case finding in the general population	Multiple	11,146	0-4 weeks	Patient tracking by dedicated research team	Relative risk regression (N=11,146)
Ismail (2020) [5]	Karnataka	Both	Individuals with symptoms identified via passive case finding (i.e., routine care) at government clinics	Multiple	8,822	0-4 weeks	Self-report by government TB program*	Relative risk regression (N=8,822)
**Non-completion of sputum microscopy evaluation**								
Balasubramanian (2004) [6]	Tamil Nadu	Rural	Adults >14 years with TB symptoms	Multiple	8,646	1-3 months*	Patient tracking by dedicated research team	Logistic regression (N=8,646)[a]
Chandrasekaran (2005) [7]	Tamil Nadu	Urban	Adults >15 years with TB symptoms	Multiple	1,000	2-4 weeks	Patient tracking by dedicated research team	Reasons for non-completion by patient interview

								(proportions) (N=92)*
Dandona (2004) [8]	Andhra Pradesh, Maharashtra, Rajasthan, and Tamil Nadu	Both	General population of people with TB symptoms	Multiple	4,310	NR*	Patient tracking by dedicated research team	Reasons for non-completion by patient interview (proportions) (N=314); Logistic regression (N=4,310)
Das (2019)[b] [9]	Chhattisgarh	Rural	General population of people with TB symptoms in a conflict-affected area	Multiple	763	2-4 weeks	Patient tracking by dedicated research team	Logistic regression (N=763)[a]
Tripathy (2013) [10]	Punjab	Rural	General population of people with TB symptoms	Multiple	1,708	2-4 weeks	Self-report by government TB program*	Logistic regression (N=1,708)[a]
**Non-completion of chest X-ray**								
Chadha (2013) [11]	Karnataka	Both	General population of people with TB symptoms who had negative initial sputum microscopy test results	Multiple	256	2-4 weeks and 1-3 months	Patient tracking by dedicated research team	Reasons for non-completion by patient interview (proportions) (N=243)
Kanakaraju (2020) [12]	Karnataka	Rural	General population of people with TB symptoms	Multiple	732	NR*	Patient tracking by dedicated research team	Relative risk regression (N=732)
Sarkar (2011) [13]	West Bengal	Both	General population of people with TB symptoms who had negative initial sputum microscopy test results	Multiple	4,875	NR*	Patient tracking by dedicated research team	Logistic regression (N=4,875)
Thomas (2006) [14]	Tamil Nadu	Both	General population of people with TB symptoms who had negative initial sputum microscopy test results	Multiple	423	NR*	Patient tracking by dedicated research team	Reasons for non-completion by patient interview (proportions) (N=148)*
**Non-completion of NAAT, line probe assay, or**								

**mycobacterial culture**								
Chadha (2011)[c] [1]	Andhra Pradesh	Both	People who met criteria for being at higher risk for having drug-resistant TB	Multiple	559	>3 months*	Self-report by government*	Reasons for non-completion from health system records (proportions) (N=559)
Kanakaraju (2020)[d] [12]	Karnataka	Rural	General population of people with TB symptoms	Multiple	732	NR*	Patient tracking by dedicated research team	Logistic regression (N=732)[a]
Natrajan (2018) [15]	Madhya Pradesh	Both	People who met criteria for being at higher risk for having drug-resistant TB	Multiple	318	NR*	Patient tracking by dedicated research team	Reasons for non-completion from health system records (proportions) (N=318)
Ranganath (2022)[e] [16]	Karnataka	Both	General population of people who receive a diagnosis of TB disease	Multiple	217	NR*	Self-report by government TB program*	Relative risk regression for non-completion of Xpert MTB/RIF or line probe assay for first line drugs among people diagnosed with TB disease (N=217)
Shankar (2021)[f] [17]	Karnataka	Both	General population of people who receive a diagnosis of TB disease	Multiple	1,660	>3 months*	Self-report by government TB program*	Relative risk regression for non-completion of the diagnostic algorithm among samples reaching reference laboratories (N=1,208)
Shewade (2017) [18]	Madhya Pradesh	Urban	People who met criteria for being at higher risk for having drug-resistant TB	Multiple	770	>3 months*	Patient tracking by dedicated research team	Reasons for non-completion from health system records (proportions) (N=770); Relative

								risk regression (N=770)
Shewade (2017) [19]	Tamil Nadu	Urban	People who met criteria for being at higher risk for having drug-resistant TB	Multiple	628	>3 months*	Patient tracking by dedicated research team	Reasons for non-completion from health system records (proportions) (N=628); Relative risk regression (N=628)
Shewade (2015) [20]	Puducherry	Urban	People who met criteria for being at higher risk for having drug-resistant TB	Multiple	341	1-3 months*	Patient tracking by dedicated research team	Reasons for non-completion from health system records (proportions) (N=341); Relative risk regression (N=341)
Shewade (2016) [21]	Puducherry	Urban	People who met criteria for being at higher risk for having drug-resistant TB	Multiple	212	NR*	Patient tracking by dedicated research team	Reasons for non-completion from health system records (proportions) (N=212); Relative risk regression (N=212)
Singla (2014)[g] [22]	Delhi	Urban	People who met criteria for being at higher risk for having drug-resistant TB	Single*	3,814	NR*	Patient tracking by dedicated research team	Logistic regression (N=3,814)

NAAT=cartridge-based nucleic acid amplification testing (e.g., Xpert MTB/RIF, Truenat), NR=Not reported, TB=tuberculosis.
*Medium or low quality for this indicator
[a]Unadjusted odds ratios and/or p-values were estimated by the systematic review team from the raw data, as these were not provided in the original study.
[b]This study compared rates of completion of the diagnostic workup between initial testing with sputum microscopy as compared to a later time period after Xpert MTB/RIF had been implemented as the initial test.
[c]This study evaluated non-completion of mycobacterial culture for DR TB evaluation and was conducted before widespread rollout of NAAT testing.
[d]This study evaluated non-completion of NAAT implemented as a part of evaluation of all individuals with presumptive TB, rather than as part of focused evaluation of individuals with presumptive drug-resistant (DR) or rifampin-resistant (RR) TB.
[e]This study evaluated non-completion of rapid genotypic first-line drug-susceptibility testing with either Xpert MTB/RIF or line probe assay for first line drugs, among people who were diagnosed with TB disease but not otherwise known to have drug-resistant disease.

[f]This study evaluated non-completion of a multi-step algorithm for drug resistance testing among all people diagnosed with TB, after implementation of a policy in which all people diagnosed with TB were supposed to undergo this algorithm. In this algorithm, people with an initial Xpert MTB/RIF test result suggesting rifampin-sensitive TB were supposed to undergo line probe assay for first-line drugs, followed by repeat Xpert MTB/RIF testing for discordant results or line probe assay for second-line drugs in cases of isoniazid monoresistance. People with an initial Xpert MTB/RIF test result suggesting rifampin-resistant TB were supposed to undergo line probe assay for second-line drugs, followed by liquid culture and drug-susceptibility testing for any evidence of resistance to second line drugs.
[g]This study evaluated non-completion of DR TB evaluation before and after introduction of line probe assay.

Table D. Factors associated with non-completion of the tuberculosis (TB) diagnostic workup by individuals with presumptive TB or presumptive drug-resistant TB in India (Gap 2)

Study	Exposure / independent variable	Unadjusted Effect Estimate (95% Confidence Interval)	P-Value	Adjusted Effect Estimate (95% Confidence Interval)	P-Value
**Non-pursual of diagnostic workup despite referral**					
**Dey 2019 (West Bengal)[a] [3]**		Values below are relative risk ratios		Values below are adjusted relative risk ratios	
	**Age (years)**				
	<=14	Ref		Ref	
	15-29	1.1 (0.6,1.9)	Not reported	1.3 (0.7,2.2)	Not reported
	30-44	1.3 (0.8,2.3)	Not reported	1.5 (0.8,2.5)	Not reported
	45-59	1.2 (0.7,2.1)	Not reported	1.4 (0.8,2.3)	Not reported
	60-74	1.3 (0.7,2.2)	Not reported	1.4 (0.8,2.5)	Not reported
	>=75	0.5 (0.2,1.3)	Not reported	0.5 (0.2,1.5)	Not reported
	Age not recorded	3.4 (2.0,5.9)*	Not reported	3.3 (1.9,5.6)*	Not reported
	**Sex[e]**				
	Male	0.8 (0.7,0.9)*	Not reported	0.77 (0.63,0.83)*	Not reported
	Female	Ref		Ref	
	**Presenting symptoms**				
	No cough but other symptoms	Ref		Ref	
	Cough with other symptoms	1.1 (0.7,1.6)	Not reported	1.1 (0.8,1.6)	Not reported
	Only cough	1.9 (1.6,2.4)*	Not reported	1.8 (1.5,2.2)*	Not reported
	Note, the risk ratio was flipped for the Forest plot to show:<br>No cough but other symptoms vs. only cough	0.52 (0.42,0.63)*	Not reported	0.56 (0.45,0.67)*	Not reported
	**Previous history of TB**				
	Yes	Ref		Ref	
	No	1.2 (0.8,1.6)	Not reported	1.0 (0.8,1.4)	Not reported
	**Family history of TB**				
	No	Ref		Ref	
	Yes	1.7 (1.5,2.0)*	Not reported	1.5 (1.3,1.8)*	Not reported
	**Diabetes**				
	Yes	Ref		Ref	
	No/Unknown	1.6 (1.2,1.9)	Not reported	1.3 (1.0,1.6)	Not reported

	**HIV**				
	Yes	Ref		Ref	
	No/Unknown	4.9 (0.7,32.0)	Not reported	6.0 (0.9,39.8)	Not reported
	**Alcohol use**				
	No	Ref		Ref	
	Yes	1.7 (1.4,2.2)*	Not reported	1.7 (1.3,2.2)*	Not reported
	**Tobacco use**				
	No	Ref		Ref	
	Yes	1.2 (1.0,1.4)	Not reported	1.2 (1.0,1.4)	Not reported
**Garg 2020 (Bihar)[a] [4]**		Values below are relative risk ratios		Values below are adjusted relative risk ratios	
	**Age (years)**				
	>=65	Ref		Ref	
	<15	1.4 (1.3,1.4)*	<0.001	1.2 (1.2,1.3)*	<0.001
	15-44	1.0 (1.0,1.1)	0.139	1.0 (1.0,1.1)	0.366
	45-64	1.0 (1.0,1.1)	0.212	1.0 (1.0,1.1)	0.164
	Note, the risk ratio was flipped for the Forest plot to show: >=65 vs. <15	0.71 (0.71,0.77)*	<0.001	0.83 (0.77,0.83)*	<0.001
	**Block**				
	Sarairanjan	Ref			
	Ujiarpur	1.0 (1.0,1.1)	0.022		
	Bibhutipur	1.0 (1.0,1.1)	0.033		
	**Sex**				
	Female	Ref		Ref	
	Male	1.1 (1.0,1.1)*	<0.001	1.1 (1.0,1.1)*	0.001
	**Previous history of anti-TB treatment**				
	No	Ref		Ref	
	Yes	0.7 (0.6,0.7)*	<0.001	0.7 (0.7,0.8)*	<0.001
	**Presenting symptoms**				
	Absence of listed signs	Ref			
	Hemoptysis in last 6 months	0.6 (0.6,0.7)*	<0.001	0.7 (0.6,0.7)*	<0.001
	Cough ≥2 weeks	0.7 (0.6,0.7)*	<0.001		
	Sputum	0.6 (0.6,0.7)*	<0.001		
	Chest pain in last 1 month	0.6 (0.6,0.7)*	<0.001		
	Fever ≥2 weeks	0.8 (0.8,0.8)*	<0.001		

	Night sweats	0.8 (0.7,0.8)*	<0.001		
	Severe weight loss in last 3 months	0.7 (0.7,0.8)*	<0.001		
	Swelling in a lymph node	1.4 (1.4,1.5)*	<0.001		
	**Alcohol use**				
	No	Ref			
	Yes	1.0 (0.9,1.2)	0.862		
	**Tobacco use**				
	No	Ref			
	Yes	0.8 (0.8,0.9)*	<0.001		
	**Source of referral**				
	Registered medical practitioner	Ref		Ref	
	Accredited Social Health Activist (ASHA)	1.3 (1.2,1.4)*	<0.001	1.2 (1.1,1.3)*	<0.001
	Anganwadi worker	1.3 (1.1,1.6)	0.003	1.2 (1.0,1.5)*	0.025
	Community practitioner	1.4 (1.3,1.5)*	<0.001	1.3 (1.2,1.4)*	<0.001
**Ismail 2020 (Karnataka)[a] [5]**		Values below are relative risk ratios		Values below are adjusted relative risk ratios	
	**Age (years)**				
	35-44	Ref		Ref	
	<15	1.19 (0.85,1.67)	Not reported	1.07 (0.77,1.5)	0.66
	15-24	0.94 (0.69,1.28)	Not reported	0.93 (0.69,1.25)	0.651
	25-34	1.17 (0.91,1.51)	Not reported	1.16 (0.91,1.48)	0.211
	45-54	0.97 (0.77,1.23)	Not reported	1.0 (0.80,1.26)	0.951
	55-64	0.97 (0.76,1.22)	Not reported	0.97 (0.77,1.22)	0.82
	>=65	0.92 (0.73,1.16)	Not reported	0.88 (0.69,1.10)	0.276
	**Sex**				
	Male	Ref		Ref	
	Female	1.05 (0.91,1.20)	Not reported	1.04 (0.91,1.19)	0.521
	Transgender	2.35 (0.68,8.17)	Not reported	1.97 (0.54,7.19)	0.301
	**Contact of TB case**				
	No	Ref		Ref	
	Yes	0.4 (0.17,0.95)	Not reported	0.42 (0.17,1.00)	0.052
	**Diabetes**				
	No	Ref		Ref	
	Yes	0.51 (0.28,0.92)	Not reported	0.76 (0.42,1.37)	0.368

	**HIV**				
	Negative	Ref		Ref	
	Unknown	2.27 (1.95,2.65)*	Not reported	2.06 (1.76,2.41)*	<0.001
	Positive	1.79 (0.97,3.27)	Not reported	1.79 (0.97,3.28)	0.059
	**Tobacco use**				
	No	Ref		Ref	
	Yes	0.65 (0.42,1.01)	Not reported	0.9 (0.91,1.39)	0.665
	**Enrollment center**				
	Peripheral health institute	Ref		Ref	
	District	1.6 (1.03,2.48)	Not reported	1.41 (0.91,2.18)	0.122
	Private chemist	2.87 (0.84,9.77)	Not reported	1.62 (0.45,5.83)	0.459
	Private health facility	2.09 (1.46,2.97)*	Not reported	1.86 (1.31,2.65)*	<0.001
	Private lab	0.76 (0.11,5.09)	Not reported	0.62 (0.09,4.16)	0.625
	TB unit	4.34 (3.59,5.25)*	Not reported	3.66 (3.0,4.42)*	<0.001
**Non-completion of sputum microscopy evaluation**					
**Balasubramanian 2004 (Tamil Nadu)[b] [6]**		Values below are odds ratios			
	**Age (years)**				
	15-24	Ref			
	25-34	0.93 (0.77,1.14)	0.487		
	35-44	0.96 (0.79,1.16)	0.644		
	45-54	0.78 (0.64,0.95)	0.013		
	55-64	0.85 (0.70,1.04)	0.106		
	65+	0.89 (0.71,1.12)	0.322		
	**Sex**				
	Female	Ref			
	Male	1.15 (1.02,1.30)*	0.024		
**Dandona 2004 (Multi-site, 8 Indian districts)[c] [8]**				Values below are adjusted odds ratios	
	**Age (years)**				
	16-30			Ref	
	31-50			0.99 (0.68,1.45)	Not reported
	>50			2.07 (1.41,3.04)*	Not reported
	**Sex**				
	Male			Ref	
	Female			1.17 (0.88,1.56)	Not reported
	**Marital status**				

	Others (separated/widowed)			Ref	
	Never married			2.63 (1.12,6.16)*	Not reported
	Married			2.67 (1.31,5.45)*	Not reported
	**Literacy**				
	Illiterate			Ref	
	Literate			1.09 (0.82,1.45)	Not reported
	**Monthly family income (Rs)**				
	<=3000			Ref	
	3001-5000			0.99 (0.60,1.64)	Not reported
	>5000			1.43 (0.89,2.29)	Not reported
	**Duration of symptoms**				
	>15 days			Ref	
	<=15 days			3.02 (2.31,3.95)*	Not reported
	**Family-related factors**				
	Person accompanying patient to clinic visit				
	Health worker			Ref	
	None			2.33 (1.47,3.68)*	Not reported
	Family/friend			0.86 (0.54,1.38)	Not reported
	**Person informed of suspicion of TB by health personnel**				
	Yes			Ref	
	No			7.75 (5.66,10.60)*	Not reported
	Do not remember			4.83 (2.24,10.39)*	Not reported
**Das 2019 (Chhattisgarh)[b] [9]**		Values below are odds ratios			
	**Diagnostic modality—sputum microscopy versus Xpert MTB/RIF**				
	Post-Xpert MTB/RIF testing	Ref			
	Pre-Xpert MTB/RIF testing (testing with sputum microscopy)	6.68 (3.48,12.79)*	<0.0001		
**Tripathy 2013 (Punjab)[b] [10]**		Values below are odds ratios			
	**Distance to DMC**				
	Distance <= 10km	Ref			
	Distance > 10km	4.65 (1.65,13.11)*	0.0036		
**Non-completion of chest X-ray**					
**Kanakaraju 2020 (Karnataka)[a,d] [12]**		Values below are relative risk ratios		Values below are adjusted relative risk ratios	

	**Age (years)**				
	25-34	Ref		Ref	
	<25	1.19 (0.91,1.56)	0.775	1.06 (0.72,1.57)	Not reported
	35-44	1.72 (1.26,2.36)*	0.281	1.23 (0.82,1.85)	Not reported
	45-54	1.47 (1.12,1.93)*	0.664	0.93 (0.63,1.36)	Not reported
	55-64	1.56 (1.19,2.05)*	0.558	1.12 (0.77,1.63)	Not reported
	>64	1.54 (1.19,1.99)*	0.761	1.06 (0.75,1.52)	Not reported
	**Sex**				
	Male	Ref		Ref	
	Female	1.14 (0.94,1.37)	0.559	1.09 (0.85,1.39)	Not reported
	Transgender	N/A		N/A	
	**Place of residence**				
	Rural	Ref			
	Urban	0.47 (0.44,0.49)*	N/A		
	**Predominant symptom**				
	Cough	Ref		Ref	
	Cough and fever	0.23 (0.18,0.3)*	0.116	0.52 (0.23,1.18)	Not reported
	Hemoptysis	0.20 (0.16,0.26)*	0.115	0.20 (0.03,1.49)	Not reported
	Not recorded	2.04 (1.26,3.31)*	0.985	1.01 (0.4,2.56)	Not reported
	**Sputum examination results**				
	Negative	Ref		Ref	
	Positive	0.79 (0.61,1.04)	0.135	0.75 (0.50,1.11)	Not reported
	**Designated microscopy center**				
	District hospital	Ref		Ref	
	Taluka hospital 1	0.25 (0.19,0.31)*	0.047	0.44 (0.19,0.99)*	Not reported
	Taluka hospital 2	3.03 (1.52,6.06)*	0.059	3.03 (0.94,9.77)	Not reported
	Primary health center 1	2.38 (0.78,7.23)	0.122	2.56 (0.79,8.37)	Not reported
	Primary health center 2	0		0	Not reported
	Primary health center 3	0		0	Not reported
	Primary health center 4	0.52 (0.32,0.82)*	0.776	0.77 (0.31,1.90)	Not reported
**Sarkar 2011 (West Bengal)[c] [13]**		Values below are odds ratios		Values below are adjusted odds ratios	
	**Income**				
	Above Poverty line	Ref			
	Below poverty line	132.7 (103.7,168.5)*			
	**Can afford x-rays from private hospital**				
	Yes	Ref		Ref	

	No	63.3(42.8,93.5)*		22.1 (13,37.7)*	Not reported
	**Knew that x-rays are needed for diagnosis**				
	Yes	Ref		Ref	
	No	4.2 (2.9,6.1)*		22.7 (8.4,61)*	Not reported
	**Distance from nearest public health facility with x-ray**				
	<30 km	Ref			
	>30 km	37.7 (29.2,48.7)*			
	**Below poverty line and >30 km from nearest public health facility with chest x-ray**			82.9 (64.7,106.3)*	Not reported
**Non-completion of NAAT, line probe assay, or mycobacterial culture**					
**Kanakaraju 2020 (Karnataka)**[b,d] [12]		Values below are odds ratios			
	**Age (years)**				
	25-34	Ref	0.74		
	<25	1.2031 (0.39,3.68)	0.42		
	35-44	1.5780 (0.52,4.76)	0.75		
	45-54	0.8594 (0.34,2.18)	0.86		
	55-64	0.9297 (0.37,2.32)	0.72		
	>64	1.1892 (0.47,3.03)			
	**Sex**				
	Male	Ref			
	Female	1.3607 (0.72,2.56)	0.338		
	Transgender	0.03 (0.003,0.22)*	0.027		
	**Place of residence**				
	Urban	Ref			
	Rural	2.78 (1.16,6.65)*	0.02		
	**Predominant symptom**				
	Cough	Ref			
	Cough and fever	0.62 (0.29,1.31)	0.21		
	Hemoptysis	0.14 (0.0056,3.71)	0.24		
	Not recorded	0.44 (0.21,0.93)*	0.03		
	**Sputum examination results**				
	Positive	Ref			
	Negative	102.04 (45.63,228.17)*	0.0001		

	**Designated microscopy center**				
	District hospital	Ref			
	Taluka hospital 1	0.47 (0.21,1.05)	0.07		
	Taluka hospital 2	0.30 (0.13,0.73)*	0.01		
	Primary health center 1	0.37 (0.10,1.45)	0.15		
	Primary health center 2	2.71 (0.15,48.86)	0.50		
	Primary health center 3	0.62 (0.07,5.21)	0.66		
	Primary health center 4	0.38 (0.10,1.50)	0.17		
	**Underwent chest radiography**				
	Yes	Ref			
	No	1.6773 (0.93,3.02)	0.08		
**Ranganath 2022 (Karnataka)[a] [16]**		Values below are relative risk ratios		Values below are adjusted relative risk ratios	
	**Age**				
	15-44 years	Ref		Ref	
	<15 years	8.57 (4.68,15.69)*	NR	1.95 (1.10,3.48)*	NR
	45-64 years	1.54 (0.71,3.35)	NR	0.97 (0.50,1.86)	NR
	>=65 years	1.17 (0.4,3.42)	NR	0.70 (0.25,1.93)	NR
	**Gender**				
	Female	Ref			
	Male	0.93 (0.49,1.77)			
	**Key populations (including people with HIV)**				
	Yes	Ref			
	No	1.92 (0.62,5.93)			
	**Diabetes mellitus**				
	No	Ref		Ref	
	Yes	NR (no people with diabetes mellitus reported)	NR		
	Missing	2.48 (1.36,4.52)*	NR	1.74 (1.12,2.70)*	NR
	**Bacteriological confirmation on sputum microscopy**				
	Yes	Ref		Ref	
	No	8.70 (4.18,18.13)*	NR	6.66 (3.09,14.3)*	NR
	**Prior treatment history**				
	New	Ref			
	Previously treated for TB	1.45 (0.62,3.39)	NR		

**Shankar 2021 (Karnataka)[a] [17]**		Values below are relative risk ratios		Values below are adjusted relative risk ratios	
	**Health facility type**				
	Government	Ref		Ref	
	Private	1.50 (0.86,2.59)	NR	1.64 (0.95,2.82)	NR
	**Specimen type**				
	Sputum	Ref		Ref	
	Extrapulmonary	0.52 (0.08,3.62)	NR	0.53 (0.07,4.04)	NR
	Not recorded	0.44 (0.14,1.36)	NR	0.35 (0.09,1.42)	NR
	**Rifampin resistance results**				
	Rifampin-susceptible	Ref		Ref	
	Rifampin-resistant	8.88 (5.86,13.46)*	NR	8.47 (5.36,13.39)*	NR
	**Reference laboratory**				
	Intermediate reference laboratory Bengaluru	Ref		Ref	
	National TB Institute Bengaluru	1.21 (0.8,1.83)	NR	0.68 (0.42,1.12)	
	**Smear microscopy results at reference laboratory**				
	Positive	Ref		Ref	
	Negative	2.67 (1.61,4.44)*	NR	2.69 (1.42,5.12)*	NR
	Not recorded	1.71 (0.66,4.47)	NR	1.45 (0.48,4.43)	NR
**Shewade 2017 (Madhya Pradesh)[a] [18]**		Values below are relative risk ratios		Values below are adjusted relative risk ratios	
	**Age (years)**				
	<14	0.9 (0.5,1.9)	Not reported	1.0 (0.6,1.9)	Not reported
	14-44	1.0 (0.9,1.2)	Not reported	1.0 (0.9,1.2)	Not reported
	45-64	Ref		Ref	
	>=65	1.3 (1.1,1.6)*	Not reported	1.3 (1.1,1.7)*	Not reported
	**Sex**				
	Female	Ref		Ref	
	Male	1.0 (0.9,1.2)	Not reported	1.1 (1.0,1.2)	Not reported
	**Presumptive MDR-TB criteria**				
	Follow up smear positive	Ref		Ref	
	Previously treated-recurrent	1.2 (0.9,1.5)*	Not reported	1.3 (1.0,1.6)*	Not reported
	Treatment after failure	1.2 (0.7,1.9)	Not reported	1.3 (0.8,2.0)	Not reported
	Treatment after LTFU	1.5 (1.2,2.0)*	Not reported	1.5 (1.1,2.1)*	Not reported
	Previously treated-others	1.7 (1.4,2.1)*	Not reported	1.6 (1.1,2.3)*	Not reported
	New patient with TB-HIV	1.6 (0.7,3.6)	Not reported	1.8 (0.8,4.2)	Not reported

	**Site of TB**				
	Pulmonary smear positive	Ref		Ref	
	Extrapulmonary	1.6 (1.4,1.9)*	Not reported	1.5 (1.0,2.2)*	Not reported
	Pulmonary smear negative	1.4 (1.2,1.6)	Not reported	1.2 (0.8,1.7)	Not reported
	**Health facility**				
	District level	Ref		Ref	
	Primary/Secondary level	1.0 (0.9,1.1)	Not reported	1.0 (0.9,1.1)	Not reported
	Medical College	1.2 (1.0,1.4)*	Not reported	1.2 (1.02,1.4)*	Not reported
**Shewade 2017 (Tamil Nadu)[a] [19]**		Values below are relative risk ratios			
	**Age (years)**				
	45-64	Ref			
	<14	3.6 (0.7,18.7)			
	14-44	1.5 (0.5,2.3)			
	>=65	2.0 (0.8,4.8)			
	**Sex**				
	Male	Ref			
	Female	1.4 (0.9,2.4)			
	**Presumptive MDR-TB criteria**				
	Retreatment- LTFU	Ref			
	Retreatment-relapse	1.2 (0.7,2.2)			
	Retreatment- failure	0.7 (0.2,3.0)			
	Retreatment- other	2.7 (1.5,5.1)*			
	Follow up smear positive	1.0 (0.5,2.3)			
	New Patient with TB/HIV	1.5 (0.7,3.2)			
	**Site of TB**				
	Pulmonary smear positive	Ref			
	Pulmonary smear negative	2.2 (1.3,3.6)*			
	Extrapulmonary	2.9 (1.6,5.4)*			
	**Health facility**				
	Primary/Secondary level	Ref			
	District level	1.5 (0.9,2.4)			
	Medical College/other	2.2 (0.9,5.4)			
**Shewade 2016 (Puducherry)[a] [21]**		Values below are relative risk ratios			
	**Age (years)**				
	45-64	Ref			

	<14	None			
	14-44	1.1 (0.7,1.8)			
	>=65	0.3 (0.04,1.9)			
	**Sex**				
	Male	Ref			
	Female	1.6 (0.9,2.7)			
	**Presumptive MDR-TB criteria**				
	Retreatment	Ref			
	Follow up smear positive	1.4 (0.8,2.4)			
	New Patient with TB/HIV	3.6 (2.0,6.5)*			
	**Sputum status**				
	Smear-positive pulmonary	Ref			
	Smear-negative pulmonary	3.0 (1.1,8.3)*			
	Smear-negative extrapulmonary	5.6 (4.0,7.8)*			
	**Health facility**				
	District level	Ref			
	Primary/Secondary level	1.4 (0.6,3.0)			
	Medical College	1.7 (0.9,3.1)			
	Other facilities	4.2 (2.6,6.9)*			
**Shewade 2015 (Puducherry)[a] [20]**		Values below are relative risk ratios			
	**Age (years)**				
	45-64	Ref			
	<14	None			
	14-44	1.08 (0.84,1.39)			
	>=65	1.38 (0.93,2.04)			
	**Sex**				
	Male	Ref			
	Female	1.46 (1.13,1.88)*			
	**Presumptive MDR-TB criteria**				
	Retreatment	Ref			
	Follow-up smear positive	1.05 (0.82,1.36)			
	New patient with TB/HIV	1.71 (1.26,2.31)*			
	**Extrapulmonary TB**				
	No	Ref			
	Yes	2.31 (1.97,2.73)*			
	**Health facility**				

	District level	Ref			
	PHC/CHC	1.80 (1.35,2.40)*			
	Medical College	1.59 (1.19,3.27)*			
	Other facilities	2.43 (1.81,3.27)*			
**Singla 2014 (Delhi)[b] [22]**		Values below are odds ratios			
	**Line-probe assay**				
	Post-line probe assay availability	Ref			
	Pre-line probe assay availability	6.94 (5.49,8.76)*	<0.0001		

NAAT, cartridge-based nucleic acid amplification test; HIV, human immunodeficiency virus; MDR-TB, multidrug-resistant tuberculosis; DMC, designated microscopy center; LTFU, loss to follow-up; PHC/CHC, primary health center/community health center

[a]Effect estimates comprise relative risk or adjusted relative risk ratios

[b]Unadjusted odds ratios and p-values were estimated by the systematic review team from the raw data, as these were not provided in the original study.

[c]Effect estimates comprise odds ratios or adjusted odds ratios

[d]Study reported completing the workup or test as the outcome, so effect estimates (odds ratio or relative risk and 95% confidence interval) were flipped to show effect estimates for the outcome of not completing the diagnostic test or workup.

[e]Reference group of the variable was switched, resulting in flipping of the effect estimate (odds ratio and 95% confidence interval), to facilitate consistency in reference groups across studies or to allow for more intuitive interpretation of the study finding.

*Indicates statistical significance at the 5% level.

References

1. Chadha SS, Sharath BN, Reddy K, Jaju J, Vishnu PH, Rao S, et al. Operational challenges in diagnosing multi-drug resistant TB and initiating treatment in Andhra Pradesh, India. PLoS One. 2011;6: e26659. doi:10.1371/journal.pone.0026659

2. Subbaraman R, Nathavitharana RR, Satyanarayana S, Pai M, Thomas BE, Chadha VK, et al. The Tuberculosis Cascade of Care in India's Public Sector: A Systematic Review and Meta-analysis. PLoS Med. 2016;13: e1002149. doi:10.1371/journal.pmed.1002149

3. Dey A, Thekkur P, Ghosh A, Dasgupta T, Bandopadhyay S, Lahiri A, et al. Active Case Finding for Tuberculosis through TOUCH Agents in Selected High TB Burden Wards of Kolkata, India: A Mixed Methods Study on Outcomes and Implementation Challenges. Trop Med Infect Dis. 2019;4. doi:10.3390/tropicalmed4040134

4. Garg T, Gupta V, Sen D, Verma M, Brouwer M, Mishra R, et al. Prediagnostic loss to follow-up in an active case finding tuberculosis programme: a mixed-methods study from rural Bihar, India. BMJ Open. 2020;10: e033706. doi:10.1136/bmjopen-2019-033706

5. Ismail IM, Kibballi Madhukeshwar A, Naik PR, Nayarmoole BM, Satyanarayana S. Magnitude and Reasons for Gaps in Tuberculosis Diagnostic Testing and Treatment Initiation: An Operational Research Study from Dakshina Kannada, South India. J Epidemiol Glob Health. 2020;10: 326–336. doi:10.2991/jegh.k.200516.001

6. Balasubramanian R, Garg R, Santha T, Gopi PG, Subramani R, Chandrasekaran V, et al. Gender disparities in tuberculosis: report from a rural DOTS programme in south India. Int J Tuberc Lung Dis. 2004;8: 323–332.

7. Chandrasekaran V, Ramachandran R, Cunningham J, Balasubramanian R, Thomas A, Sudha G, et al. Factors leading to tuberculosis diagnostic drop-out and delayed treatment initiation in Chennai, India. Int J Tuberc Lung Dis. 2005;9: S172.

8. Dandona R, Dandona L, Mishra A, Dhingra S, Venkatagopalakrishna K, Chauhan LS. Utilization of and barriers to public sector tuberculosis services in India. Natl Med J India. 2004;17: 292–299.

9. Das M, Pasupuleti D, Rao S, Sloan S, Mansoor H, Kalon S, et al. GeneXpert and Community Health Workers Supported Patient Tracing for Tuberculosis Diagnosis in Conflict-Affected Border Areas in India. Trop Med Infect Dis. 2019;5. doi:10.3390/tropicalmed5010001

10. Tripathy JP, Srinath S, Naidoo P, Ananthakrishnan R, Bhaskar R. Is physical access an impediment to tuberculosis diagnosis and treatment? A study from a rural district in North India. Public Health Action. 2013;3: 235–239. doi:10.5588/pha.13.0044

11. Chadha VK, Praseeja P, Hemanthkumar NK, Shivshankara BA, Sharada MA, Nagendra N, et al. Implementation efficiency of a diagnostic algorithm in sputum smear-negative presumptive tuberculosis patients. Int J Tuberc Lung Dis. 2014;18: 1237–1242. doi:10.5588/ijtld.14.0218

12. Kanakaraju M, Nagaraja SB, Satyanarayana S, Babu YR, Madhukeshwar AK, Narasimhaiah S. Chest Radiography and Xpert MTB/RIF® Testing in Persons with Presumptive Pulmonary TB: Gaps and Challenges from a District in Karnataka, India. Tuberc Res Treat. 2020;2020: 5632810. doi:10.1155/2020/5632810

13. Sarkar J, Murhekar MV. Factors associated with low utilization of x-ray facilities among the sputum negative chest symptomatics in Jalpaiguri district (West Bengal) 2009. Indian J Tuberc. 2011;58: 208–211.

14. Thomas A, Gopi PG, Santha T, Jaggarajamma K, Charles N, Prabhakaran E, et al. Course of action taken by smear negative chest symptomatics: A report from a rural area in South India. Indian Journal of Tuberculosis. 2006;53: 4–6.

15. Natrajan S, Singh AR, Shewade HD, Verma M, Bali S. Pre-diagnosis attrition in patients with presumptive MDR-TB in Bhopal, India, 2015: a follow-up study. Public Health Action. 2018;8: 95–96. doi:10.5588/pha.18.0015

16. Ranganath R, Shewade HD, Bahadur AK, Naik V, Nagaraja SB, Kumar AMV, et al. Uptake of universal drug susceptibility testing among people with TB in a south Indian district: How are we faring? Trans R Soc Trop Med Hyg. 2022;116: 43–49. doi:10.1093/trstmh/trab051

17. Shankar S U, Kumar AMV, Venkateshmurthy NS, Nair D, Kingsbury R, R P, et al. Implementation of the new integrated algorithm for diagnosis of drug-resistant tuberculosis in Karnataka State, India: How well are we doing? PLoS One. 2021;16: e0244785. doi:10.1371/journal.pone.0244785

18. Shewade HD, Kokane AM, Singh AR, Verma M, Parmar M, Chauhan A, et al. High pre-diagnosis attrition among patients with presumptive MDR-TB: an operational research from Bhopal district, India. BMC Health Serv Res. 2017;17: 249. doi:10.1186/s12913-017-2191-6

19. Shewade HD, Nair D, Klinton JS, Parmar M, Lavanya J, Murali L, et al. Low pre-diagnosis attrition but high pre-treatment attrition among patients with MDR-TB: An operational research from Chennai, India. J Epidemiol Glob Health. 2017;7: 227–233. doi:10.1016/j.jegh.2017.07.001

20. Shewade HD, Govindarajan S, Sharath BN, Tripathy JP, Chinnakali P, Kumar AMV, et al. MDR-TB screening in a setting with molecular diagnostic techniques: who got tested, who didn’t and why? Public Health Action. 2015;5: 132–139. doi:10.5588/pha.14.0098

21. Shewade HD, Govindarajan S, Thekkur P, Palanivel C, Muthaiah M, Kumar AMV, et al. MDR-TB in Puducherry, India: reduction in attrition and turnaround time in the diagnosis and treatment pathway. Public Health Action. 2016;6: 242–246. doi:10.5588/pha.16.0075

22. Singla N, Satyanarayana S, Sachdeva KS, Van den Bergh R, Reid T, Tayler-Smith K, et al. Impact of introducing the line probe assay on time to treatment initiation of MDR-TB in Delhi, India. PLoS One. 2014;9: e102989. doi:10.1371/journal.pone.0102989
