# Supplementary material for: Barriers to engagement in the care cascade for tuberculosis disease in India: A systematic review of quantitative studies
Source: PLoS Med. 2024 May 28;21(5):e1004409. doi: 10.1371/journal.pmed.1004409 (PMC11166313; doi:10.1371/journal.pmed.1004409)
Supplement: S3 Appendix — (PDF) [file pmed.1004409.s003.pdf]

S3 Appendix. Methods and study characteristics for the systematic review of barriers to treatment initiation for people diagnosed with tuberculosis disease (Gap 3)

Supplement to:
Barriers to engagement in the care cascade for tuberculosis disease in India: a systematic review of quantitative studies

Authors:
Tulip A. Jhaveri, Disha Jhaveri, Amith Galivanche, Maya Lubeck-Schricker, Dominic Voehler, Mei Chung, Pruthu Thekkur, Vineet Chadha, Ruvandhi Nathavitharana, Ajay M.V. Kumar, Hemant Deepak Shewade, Katherine Powers, Kenneth H. Mayer, Jessica E. Haberer, Paul Bain, Madhukar Pai, Srinath Satyanarayana, Ramnath Subbaraman

Correspondence:
Ramnath Subbaraman, MD, MSc, FACP
Tufts University School of Medicine
Department of Public Health and Community Medicine
136 Harrison Ave., MV120
Boston, MA 02130, USA
Email: ramnath.subbaraman@tufts.edu

Table of Contents

Methods **... 3**

Objectives 3

Search strategy 3

Table A. Search strategy to identify manuscripts regarding diagnosed TB patients not getting successfully started on, or registered in, treatment (Gap 3). This same search was also used to identify relevant articles for Gaps 2 and 4. 4

Inclusion and exclusion criteria 4

Study selection...5

Fig A. PRISMA flowchart: study selection for the systematic review of non-completion of the diagnostic workup, pretreatment loss to follow-up, and on-treatment loss to follow-up for TB patients in India (Gaps 2, 3 and 4) 6

Quality assessment of quantitative studies 7

Table B. Criteria for assessing quality of quantitative studies evaluating failure of diagnosed TB patients not getting successfully registered in treatment. 7

Data extraction and analysis 7

Table C. Characteristics of the included studies for patients diagnosed with tuberculosis who did not start on, or get registered in, treatment (Gap 3) 9

Table D. Factors associated with patients diagnosed with tuberculosis not getting started on, or registered in, treatment (Gap 3) 13

References **.. 24**

Methods

Objectives

The objective of this systematic review is to understand why some patients who are diagnosed with tuberculosis (TB)—in either public or private sector facilities in India—do not get successfully registered in treatment, a problem also known as pretreatment loss to follow-up (PTLFU) (Gap 3 in the care cascade). We broadly defined patients diagnosed with TB as comprising individuals identified by India's National TB Elimination Program (NTEP) or private sector healthcare providers as having active TB based on a microbiological diagnosis (e.g., positive sputum smear microscopy result, positive Xpert MTB/RIF result, or histological finding) or empirical diagnosis (e.g., based on consistent clinical symptoms, clinical findings, or findings on a non-specific test such as chest X-ray). The protocol was registered in PROSPERO in April 2020 under ID CRD42020159361.

We searched for studies that followed such patients after their diagnosis to determine whether they subsequently started treatment for active TB and, ideally, got notified (or registered) as being on treatment to India's NTEP. When such information is available, we use notification or registration to define a successful outcome, as notification indicates that the patient has been reported to the NTEP as having been started on treatment, such that healthcare providers are accountable for the patient's treatment outcome [1].

We extracted two types of quantitative findings that help to understand PTLFU:

(a) *Factors associated with PTLFU in regression analyses:* For studies comparing patients who did or did not start on, or get registered in, TB treatment, we extracted effect estimates for independent variables (i.e., exposures or predictors) associated with PTLFU. Effect estimates included odds ratios, risk ratios, hazard ratios, or beta-coefficients, depending on the approach to analysis.

(b) *Reasons reported by patients for PTLFU*: For studies that surveyed patients who experienced PTLFU regarding reasons they had not started on, or registered in, TB treatment, we extracted the proportion of patients who reported a given reason for PTLFU.

Search strategy

Three separate searches were conducted to identify articles. The first search was conducted as part of a previously published study quantifying gaps in India's TB care cascade [2]. We used articles identified for that review that evaluated Gap 3 in the TB care cascade but that also reported factors and reasons associated with PTLFU. For that review, a medical librarian searched PubMed, Embase, and Web of Science for studies published between January 1, 2000 and February 26, 2015, without language restrictions, using search terms and related variants for "tuberculosis", "India", and "loss to follow-up", including "pretreatment loss to follow-up" and "initial default" to include early losses preceding treatment initiation (Table A). We also carried out electronic searches of key Indian journals that were not indexed for that entire time window: the Indian Journal of Tuberculosis, Lung India, the Indian Journal of Chest and Allied Sciences, the India Journal of Public Health, and the Indian Journal of Community Medicine. Additional studies were identified by searching reference lists of the primary studies and review articles. Notably, given similarities in the search terms for identifying losses during the

diagnostic workup (Gap 2), pretreatment loss to follow-up (Gap 3), and poor outcomes on treatment (Gap 4), this single search was used to identify studies related to all of these gaps. We screened all identified studies from this previous review for potential inclusion in our current review; however, different data were extracted from studies that met inclusion criteria.

To update our review, we conducted a second refresher search using the same search terms for October 2, 2015 to October 1, 2019. We did not repeat hand searches of the Indian journals listed above, because all of these journals had been indexed in PubMed prior to the time period of this more recent search. Due to the extensive time required to extract data from the articles identified for this systematic review, we performed a third refresher search using the same search terms for October 2, 2019 to August 14, 2023. Finally, additional studies were identified by looking through the reference lists of the included primary studies and relevant review articles that were identified by the searches and by outreach to experts in the field.

Table A. Search strategy to identify manuscripts regarding diagnosed tuberculosis (TB) patients not getting successfully started on, or registered in, treatment (Gap 3). This same search was also used to identify relevant articles for Gaps 2 and 4.

Terms for tuberculosis:	“tuberculosis”[Mesh] OR *Mycobacterium tuberculosis*[tiab] OR TB[tiab] OR MDRTB[tiab] OR XDRTB[tiab]
Terms for India:	“India”[Mesh] OR India[tiab] OR India[ad] OR Indian[tiab] OR Indians[tiab]
Terms for loss to follow-up or other poor outcomes:	“patient dropouts”[tiab] OR “treatment refusal”[Mesh] OR “patient compliance”[Mesh] OR lost to follow up[tiab] OR loss to follow up[tiab] OR default*[tiab] OR compliance[tiab] OR adherence[tiab] OR noncompliance[tiab] OR nonadherence[tiab] OR diagnostic dropout [tiab] OR patient cooperation[tiab] OR dropout*[tiab] OR linkage to care[tiab] OR retention[tiab] OR attrition[tiab] OR cascade of care[tiab] OR treatment cascade[tiab] OR treatment success*[tiab] OR treatment completion[tiab] OR cure[tiab] OR pretreatment loss to follow-up[tiab] OR initial default[tiab]; treatment failure [tiab]

Inclusion and exclusion criteria

We applied the following criteria for inclusion and exclusion of studies for this systematic review.

Inclusion criteria included the following:

(1) Studies that followed patients who were diagnosed with any form of active TB to evaluate whether these patients got successfully started on, or registered in, treatment.
(2) Studies also had to have assessed reasons that individuals in the study did not get started on, or registered in, treatment. These studies could have compared characteristics of those who did or did not experience PTLFU (e.g., regression analyses) or conducted structured interviews with patients who experienced PTLFU to understand reasons for this outcome.

Exclusion criteria included the following:

(1) Studies that only described the proportion of patients who experienced PTLFU without stating reasons for not starting on, or registering in, treatment.
(2) Studies with data collected prior to the year 2000, as India's Revised National TB Control Programme (now called the NTEP) did not achieve nationwide coverage until the early 2000s.
(3) Studies only containing qualitative data evaluating on PTLFU. Findings from studies containing qualitative data will be reported in a separate paper.

Study selection

Each citation identified by the search was independently assessed by at least two reviewers (among TJ, DJ, AG, DV, MLS and KP) for their eligibility at the title and abstract evaluation stage and again subsequently at the full text evaluation stage (Fig A). Disagreements between the two reviewers were resolved by discussion or, if necessary, through consultation of a third reviewer (RS). Independent selection of articles at the title and abstract and full text stages was conducted using Covidence software (Veritas Health Innovations, Melbourne, Australia); however, quality assessment and extraction of study findings was conducted using an Excel spreadsheet.

Fig A. PRISMA flowchart: study selection for the systematic review of non-completion of the diagnostic workup, pretreatment loss to follow-up, and on-treatment loss to follow-up for people with tuberculosis (TB) in India (Gaps 2, 3 and 4)

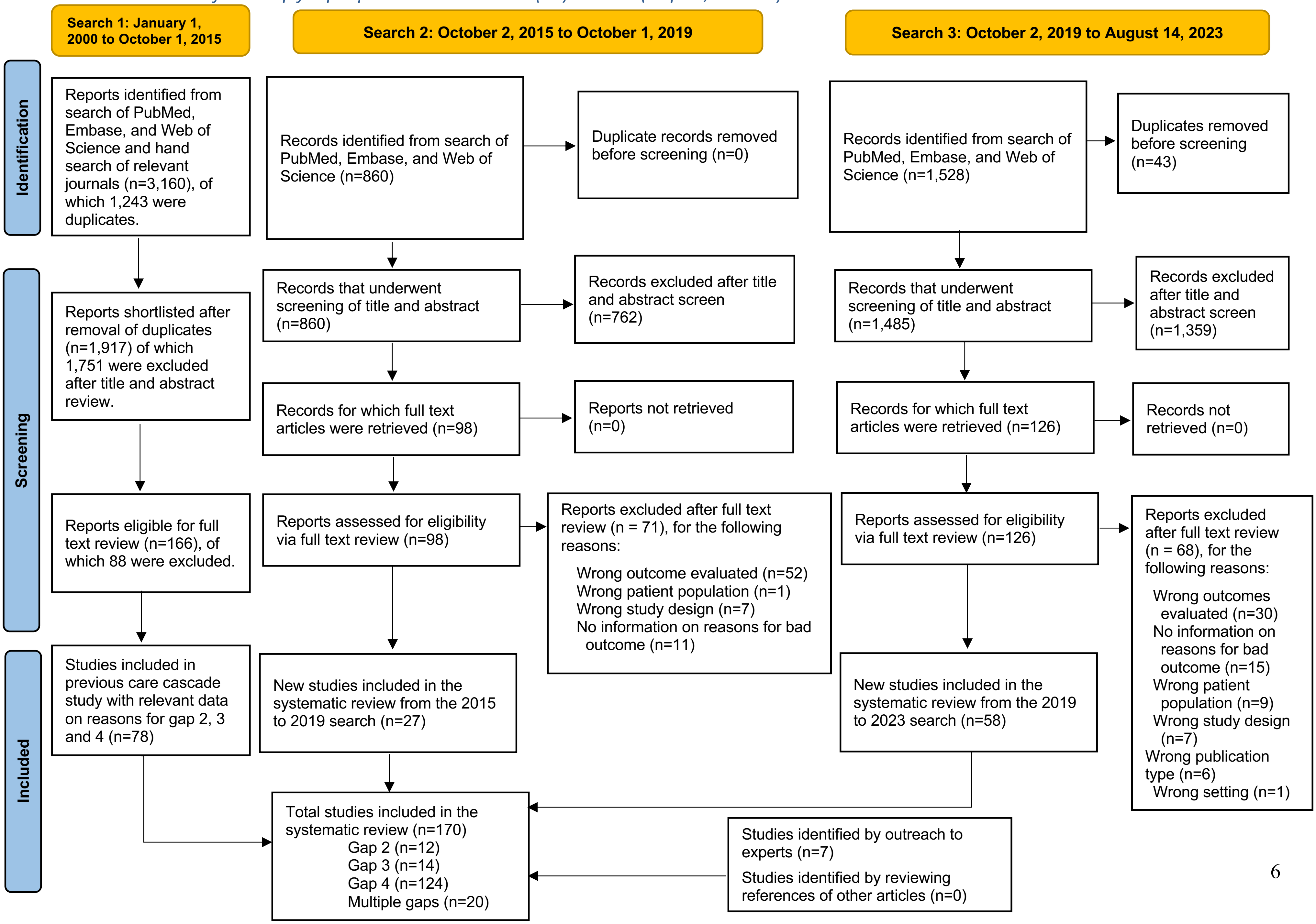

Quality assessment of quantitative studies

In our previous systematic review, we developed quality criteria relevant to studies focused on identifying patients who did not get successfully started on, or registered in, TB treatment (Table B), because there were no existing standardized guidelines for assessing the quality of these types of studies. Although we extracted different data from these studies in the current systematic review, we used the same quality criteria for studies that included relevant quantitative information, because these criteria are still appropriate for assessing the overall quality of these types of observational studies.

We classified the health facility-based strategy for sampling patients in each study based on whether it was comprehensive, random, or convenience. Studies using convenience sampling were excluded from analysis. Studies that assessed more than 150 patients and at over one government designated microscopy center (DMC) were rated as being higher in quality than studies that assessed less than 150 patients at a single government DMC. Studies that assessed PTLFU within a shorter time frame after diagnosis (e.g., within 1-2 months) were rated as being higher in quality than studies that assessed this outcome 3 or more months after diagnosis, since tracking patients to find out accurate outcomes becomes much harder with time due to patient mobility. Studies in which a dedicated research team was used to track PTLFU patients were rated as being higher in quality than studies that relied on self-report by local TB programs to evaluate PTLFU.

Table B. Criteria for assessing quality of quantitative studies evaluating failure of diagnosed TB patients not getting successfully registered in treatment.

Criterion	Quality level
**Sampling strategy**	
Random or comprehensive sampling at selected facilities	High
Convenience sampling or not reported	Low (exclude findings from analysis)
**Sample size**	
>1 center and 150+ patients	High
Single center study with 150+ patients	Medium
<150 patients or not reported	Low
**Time frame of research fieldwork after patient diagnosis**	
2 weeks to 1 month after diagnosis	High
1-3 months after diagnosis	Medium
>3 months after diagnosis or not reported	Low
**Method of evaluating outcome**	
Patient tracking by a dedicated research team	High
Relying on self-report by government TB program or not reported	Medium to Low

Data extraction and analysis

Multiple reviewers (TJ, AG, DV, MLS, and DJ) independently extracted data from each included study into a structured form on an Excel spreadsheet; however, we ensured that every article had data independently extracted by at least two or more reviewers. Disagreements were

resolved by discussion or, if necessary, by consulting a supervising reviewer (RS). From each study, we extracted information on the study design, location, setting (i.e., urban versus rural), sample size, and variables of interest (Table C). We also divided studies based on whether they evaluated PTLFU for patients with drug-susceptible versus drug-resistant TB.

For studies that compared patients who did or did not complete PTLFU, we extracted adjusted and unadjusted effect estimates (odds ratios, risk ratios, hazard ratios, and beta-coefficients) from regression analyses. For studies that did not report effect estimates, we calculated unadjusted odds ratios from the data provided, if possible. For studies that reported reasons why patients experienced PTLFU, we extracted the proportion of patients surveyed who reported a given reason for PTLFU. Of note, some studies considered initiation of TB treatment but where reporting was delayed or who initiated treatment at other government centers or in the private sector to be a negative outcome; however, we considered treatment initiation in such situations to be a potentially successful outcome. As such, for such studies, we modified the denominator of patients surveyed to exclude those who had initiated treatment in these other scenarios. We also excluded patients who had positive sputum smear microscopy results as part of treatment follow-up from the denominator. For effect estimates and proportions, we extracted information on 95% confidence intervals (95% CIs) where available; if 95% CIs were not reported, we calculated these from the data provided, if possible. We also recalculated 95% CIs for studies in which the denominator had been modified to excluded patients who had positive sputum smear microscopy results as part of treatment follow-up, or who initiated treatment with delayed reporting, at other government sites, or in the private sector.

For some variables, we also changed the reference group as needed for consistency of reporting across studies. For example, because most studies compared men to the reference group of women, we “flipped” effect estimates and confidence intervals for studies that presented men as the reference group. This allowed us to consistently present women as the reference group for findings regarding gender.

After “flipping” selected effect estimates from the regression analyses, we reported all unadjusted and adjusted effect estimates, regardless of statistical significance, organized by study (Table D). For the main manuscript and Forest plot, we restricted ourselves to presenting statistically significant adjusted effect estimates from multivariable analyses, as these may represent more meaningful associations from higher-quality analyses. After extracting this subset of findings, we organized findings into categories using our framework of demand- and supply-side factors (main manuscript, Table 1). For findings on reasons why patients experienced PTLFU, we presented all reported proportions in the Forest Plots in the main manuscript; we also organized these findings using our framework of demand- and supply-side categories.

To visualize quantitative findings, we generated Forest plots of effect estimates odds ratios, risk ratios, hazard ratios, beta-coefficients, and proportions using Stata version 16.1 (College Station, TX, USA). We did not conduct meta-analyses of data, because we extracted findings representing a diverse set of variables from every study.

Table C. Characteristics of the included studies for patients diagnosed with tuberculosis (TB) who did not start on, or get registered in, treatment (Gap 3)

**Citation** (year)	**Location**	**Urban, rural, both, or unknown**	**Type of population** (type of TB or other aspects of the patient population)	**Single or multiple designated microscopy centers (DMCs)**	**Sample size** (overall sample studied to identify PTLFU patients)	**Time frame of research fieldwork after patient diagnosis**	**Methodology** (Patient tracking by a dedicated research team versus self-report by the government TB program)	**Type of findings included in the study** (N=sample or denominator of patients for given analysis)
Drug-susceptible adult TB patients								
Ahmed (2009) [3]	Karnataka	Rural	General population (mostly drug-susceptible TB)	Multiple	232	0-4 weeks	Self-report by the government TB program*	Logistic regression (N=232) (only summary of findings presented)
Balasubramanian (2004) [4]	Tamil Nadu	Rural	General population (mostly drug-susceptible TB)	Multiple	1,064	1-3 months*	Both	Logistic regression (N=1,064)[a]
Dandona (2004) [5]	Andhra Pradesh, Maharashtra, Rajasthan, Tamil Nadu	Both	General population (mostly drug-susceptible TB)	Multiple	3,994	Not reported*	Patient tracking by a dedicated research team	Logistic regression (N=3,994)[a]<br><br>Reasons for PTLFU (proportions) (N=43)[b]
Das (2019) [6]	Chhattisgarh	Rural	General population (mostly drug-susceptible TB); Conflict-affected border areas	Multiple	297	0-4 weeks	Patient tracking by a dedicated research team	Logistic regression (N=297)[a]
Dave (2013) [7]	Gujarat	Both	General population (mostly drug-susceptible TB)	Multiple	3,159	Not reported*	Patient tracking by a dedicated research team	Reasons for PTLFU (proportions) (N=2,494)[b]

Gopi (2005) [8]	Tamil Nadu	Rural	General population (mostly drug-susceptible TB)	Multiple	1,292	1-3 months*	Patient tracking by a dedicated research team	Logistic regression (N=1,292)[a]<br><br>Reasons for PTLFU (N=200 of whom 90 were interviewed)[b]
Ismail (2020) [9]	Karnataka	Both	General population (mostly drug-susceptible TB)	Multiple	822	>3 months*	Patient tracking by a dedicated research team	Relative risk regression (N=822)
Khandekar (2013) [10]	Delhi	Urban	General population (mostly drug-susceptible TB)	Single*	1,361	Not reported*	Patient tracking by a dedicated research team	Logistic regression (N=1,283)[a]
Kumar (2013) [11]	Delhi	Urban	General population (mostly drug-susceptible TB)	Single*	184	1-3 months*	Self-report by the government TB program*	Logistic regression (N=184)[a]
Mandal (2014) [12]	West Bengal	Both	General population (mostly drug-susceptible TB)	Multiple	132*	0-4 weeks	Patient tracking by a dedicated research team	Reasons for PTLFU (proportions) (N=132)
Majella (2021) [13]	Puducherry	Urban	General population (mostly drug-susceptible TB)	Single*	310	0-4 weeks	Patient tracking by a dedicated research team	Logistic regression (N=310)
Mehra (2013) [14]	Uttarakhand	Urban	General population (mostly drug-susceptible TB)	Single*	555	0-4 weeks	Patient tracking by a dedicated research team	Logistic regression (N=555)[a]<br><br>Reasons for PTLFU (proportions) (N=98)
Pardeshi (2018) [15]	Multi-state (representative population-based sample across 29 states and 7 union territories)	Both	General population (mostly drug-susceptible TB)	Multiple	5,607	Not reported*	Self-report by the government TB program*	Logistic regression (N=5,607)

Pillai (2015) [16]	Puducherry	Urban	General population (mostly drug-susceptible TB)	Multiple	950	Not reported*	Patient tracking by a dedicated research team	Reasons for PTLFU (proportions) (N=20)
Rawat (2012) [17]	Uttarakhand	Rural	General population (mostly drug-susceptible TB)	Single*	466	Not reported*	Self-report by the government TB program*	Reasons for PTLFU (proportions) (N=21) (only summary of findings presented for some reasons)
Sai Babu (2008) [18]	Andhra Pradesh and Telangana	Both	General population (mostly drug-susceptible TB)	Multiple	15,631	Not reported*	Patient tracking by a dedicated research team	Reasons for PTLFU (proportions) (N=552)[b]
Selvaraju (2022) [19]	Tamil Nadu	Rural	General population (mostly drug-susceptible TB)	Multiple	149*	Not reported*	Patient tracking by a dedicated research team	Logistic regression (N=149)[a]
Singh (2020) [20]	Uttarakhand	Both	General population (mostly drug-susceptible TB)	Multiple	256	Not reported*	Self-report by the government TB program*	Logistic regression (N=256)[a]
Thomas (2018) [1]	Tamil Nadu	Urban	General population (mostly drug-susceptible TB)	Multiple	344	0-4 weeks	Patient tracking by a dedicated research team	Logistic regression (N=344)<br><br>Reasons for PTLFU (proportions) (N=76)
Tripathy (2013) [21]	Punjab	Rural	General population (mostly drug-susceptible TB)	Multiple	156	Not reported*	Self-report by the government TB program*	Logistic regression (N=156)[a]
Drug-resistant adult TB patients								
Chadha (2011) [22]	Andhra Pradesh	Both	Drug-resistant TB patients	Multiple	169	>3 months*	Self-report by the government TB program*	Reasons for PTLFU (proportions) (N=48)[b]

Jain (2018) [23]	Madhya Pradesh	Urban	Drug-resistant TB patients	Single*	474	0-4 weeks	Patient tracking by a dedicated research team	Logistic regression (N=474) (only summary of findings presented)
Natrajan (2018) [24]	Madhya Pradesh	Both	Drug-resistant TB patients	Multiple	74*	Not reported*	Patient tracking by a dedicated research team	Reasons for PTLFU (proportions) (N=6)[b]
Shewade (2017) [25]	Tamil Nadu	Urban	Drug-resistant TB patients	Multiple	74*	Not reported*	Patient tracking by a dedicated research team	Relative risk regression (N=74)
Shewade (2018) [26]	Gujarat	Both	Drug-resistant TB patients	Multiple	257	>3 months*	Patient tracking by a dedicated research team	Relative risk regression (N=257)
Singla (2014) [27]	Delhi	Urban	Drug-resistant TB patients	Single*	688	Not reported*	Patient tracking by a dedicated research team	Logistic regression (N=688)[a]
Pediatric TB patients								
Raizada (2018) [28]	Tamil Nadu, Delhi, Andhra Pradesh, West Bengal	Urban	Children (0-14 years) with drug-susceptible and drug-resistant TB	Multiple	3,340	Not reported*	Patient tracking by a dedicated research team	Logistic regression for drug-susceptible TB (N=3,045)[a]<br><br>Logistic regression for drug-resistant TB (N=295)[a]

PTLFU, pretreatment loss to follow-up; TB, tuberculosis,
*Medium or low quality for this indicator
[a]Unadjusted odds ratios and p-values were estimated by the systematic review team from the raw data, as these were not provided in the original study.
[b]Denominator reported here varies from what is reported in the original study, as we excluded patients from the denominator who had initiated treatment with delayed reporting or who initiated treatment at other government sites or in the private sector, as these represented potentially successful outcomes. We also excluded patients who had positive sputum smear microscopy results as part of follow-up testing while already on treatment.

Table D. Factors associated with patients diagnosed with tuberculosis (TB) not getting started on, or registered in, treatment (Gap 3)

Study	Exposure / Independent variable	Unadjusted Effect Estimate (95% Confidence Interval)	P-value	Adjusted Effect Estimate (95% Confidence Interval)	P-value
Drug-susceptible TB					
**Ahmed 2009 (Karnataka) [3]**					
	**Sex**	Study reported higher PTLFU among men (23%) than women (9%) but data not provided to estimate odds ratio.	0.004*		
**Balasubramanian 2004 (Tamil Nadu)[a,b] [4]**		Values below are odds ratios			
	**Sex**				
	Female	Ref			
	Male	1.11 (0.77,1.67)	0.6		
	**Age (Years)**		Note: paper reports p=0.02 using Chi-Squared for trend for age		
	15-24	Ref			
	25-34	0.74 (0.35,1.57)	0.44		
	35-44	1.57 (0.80,3.06)	0.19		
	45-54	1.51 (0.78,2.95)	0.22		
	55-64	1.75 (0.89,3.44)	0.11		
	65 and above	2.65 (1.27,5.51)*	0.009*		
**Dandona 2004** (multi-state)[a,b] **[5]**		Values below are odds ratios			
	**Sex**				
	Female	Ref			
	Male	1.27 (0.91,1.78)	0.16		
	**Age (Years)**				
	16-30	Ref			
	31-50	0.88 (0.6,1.29)	0.51		
	Over 50	1.13 (0.75,1.71)	0.54		
	**Marital status**				
	Never married	Ref			
	Married	1.39 (0.82,2.36)	0.22		
	Others	0.94 (0.40,2.23)	0.90		

	**Literacy**				
	Literate	Ref			
	Illiterate	0.86 (0.62,1.18)	0.35		
	**Income in Indian rupees**				
	<3000 monthly	Ref			
	3001-5000 monthly	0.81 (0.42,1.56)	0.53		
	>5000 monthly	1.69 (0.96,2.99)	0.72		
	**Who informed patient of diagnosis**				
	Health personnel other than doctor	Ref			
	Doctor	1.68 (0.75,3.74)			
	Patient did not collect report	15697.8 (887.92,277524.8)*	<0.0001*		
	Family member	11.16 (4.79,26.01)*	<0.0001*		
**Das 2019 (Chhattisgarh)[a,b] [6]**		Values below are odds ratios			
	**Availability of GenXpert**				
	Post GenXpert	Ref			
	Pre GenXpert	1.94 (0.43,8.84)	0.39		
**Gopi 2005 (Tamil Nadu)[a,b] [8]**		Values below are odds ratios			
	**Sex**				
	Female	Ref			
	Male	2.17 (1.42,3.31)*	0.0003*		
	**Age (Years)**				
	15,44	Ref			
	45 and above	1.55 (1.15,2.7)*	0.004*		
	**Case finding approach**				
	Passive case finding (i.e., diagnosed in health facility)	Ref			
	Active case finding (i.e., diagnosed in community survey)	1.75 (1.25,2.47)*	0.001*		
**Ismail 2020 (Karnataka)[c] [9]**		Values below are relative risk ratios		Values below are adjusted relative risk ratios	
	**Sex**				
	Male	Ref		Ref	
	Female	1.34 (0.62,2.93)		1.38 (0.67,2.85)	0.374
	Transgender	N/A		N/A	
	**Age (Years)**				
	35-44	Ref		Ref	
	<15	N/A		N/A	
	15-24	0.8 (0.19,3.27)		0.74 (0.17,3.23)	0.691

	25-34	1.12 (0.33,3.80)		1.21 (0.32,4.58)	0.775
	45-54	0.87 (0.25,2.97)		1.05 (0.32,3.45)	0.94
	55-64	0.76 (0.18,3.11)		0.88 (0.20,3.73)	0.87
	65 and above	1.43 (0.42,4.83)		1.46 (0.36,5.81)	0.59
	**Type of TB**				
	New	Ref		Ref	
	Recurrent	3.04 (1.55,7.78)*		3.2 (1.38,7.46)*	0.007*
	**Contact of TB case**				
	No	Ref		Ref	
	Yes	1.33 (0.18,9.41)		0.83 (0.85,8.24)	0.88
	**Diabetes mellitus**				
	No	Ref		Ref	
	Yes	0.26 (0.03,1.93)		0.33 (0.04,2.7)	0.304
	**Type of enrollment center**				
	Peripheral health institute	Ref		Ref	
	District	Not reported		Not reported	Not reported
	Private chemist	Not reported		Not reported	Not reported
	Private health facility	3.01 (1.26,7.22)*		2.73 (1.08,6.9)*	0.034*
	Private lab	5.65 (1.40,22.70)*		5.12 (1.38,18.92)*	0.014*
	TB unit	Not reported		Not reported	Not reported
	Unknown	Not reported		Not reported	Not reported
**Khandekar 2013 (Delhi)[a,b] [10]**		Values below are odds ratios			
	**Sex**				
	Female	Ref			
	Male	1.03 (0.8,1.31)	0.83		
	**Age (Years)**				
	0-14	Ref			
	15-24	0.75 (0.54,1.03)	0.07		
	25-44	0.87 (0.64,1.2)	0.4		
	45-59	0.71 (0.43,1.2)	0.2		
	60 and above	1.04 (0.55,2.0)	0.9		
	**Type of TB**				
	Pulmonary TB	Ref			
	Extrapulmonary TB	0.82 (0.63,1.06)	0.14		
	Both pulmonary and extrapulmonary	0.90 (0.52,1.57)	0.71		
	**Type of patient**				

	New	Ref			
	Relapse	1.27 (0.51,3.16)	0.6		
	Treatment after default	1.36 (0.25,7.49)	0.72		
	Failure	0.99 (0.31,3.14)	0.99		
	Others	1.25 (0.83,1.88)	0.28		
**Kumar 2013 (Delhi)[a,b] [11]**		Values below are odds ratios			
	**Sex**				
	Female	Ref			
	Male	0.65 (0.31,1.34)	0.24		
	**Age (Years)**				
	0-14	Ref			
	15-24	1.00 (0.11,9.07)	1		
	25-34	2.49 (0.29,21.66)	0.41		
	35-44	2.21 (0.24,20.35)	0.49		
	45-54	2.11 (0.21,21.01)	0.53		
	55-64	1.14 (0.09,14.68)	0.92		
	65 and above	1.60 (0.08,31.77)	0.76		
**Majella 2021 (Puducherry)[c] [13]**		Values below are risk ratios		Values below are adjusted risk ratios	
	**Age**				
	0-14	1.6 (0.4,7.5)	0.53	2.1 (0.5,8.9)	0.32
	15-24	Ref		Ref	
	25-34	1.2 (0.4,3.6)	0.78	0.8 (0.3,2.0)	0.68
	35-44	1.9 (0.7,5.1)	0.21	1.0 (0.4,2.8)	0.94
	45-54	1.1 (0.4,3.1)	0.92	0.6 (0.2,2.0)	0.42
	55-64	2.2 (0.8,6.1)	0.12	1.3 (0.4,3.7)	0.68
	65+	1.4 (0.3,6.6)	0.66	0.7 (0.2,2.7)	0.59
	**Exposure to mobile intervention**				
	Phone calls reminding person to register for TB treatment	0.5 (0.3,0.9)	0.02*	0.5 (0.3,0.9)	0.03*
	No phone call	Ref		Ref	
	**Gender**				
	Male	2.7 (1.2,6.1)	0.02*	2.4 (1.0,6.0)	0.05*
	Female	Ref			
	**Education**				
	Primary or less	2.7 (0.9,7.3)	0.05		
	Secondary	1.0 (0.3,2.9)	0.98		
	Tertiary	Ref			

	**Occupation**				
	Employed	1.6 (0.7,3.8)	0.26		
	Unemployed	1.5 (0.6,4.1)	0.40		
	Student	Ref			
	Housewife	1.0 (0.3,3.4)	0.99		
	**Marital status**				
	Ever married	1.4 (0.7,3.1)	0.36	Ref	
	Never married	Ref		0.7 (1.3,1.8)	0.44
	**Residence**				
	Within the state	Ref		Ref	
	Neighboring districts	1.0 (0.4,2.6)	0.99	0.9 (0.3,2.3)	0.77
	Others	2.2 (0.9,5.5)	0.09	1.9 (0.7,5.1)	0.17
	**Current tobacco user**				
	Yes	2.1 (1.2,3.6)	0.01*	2.4 (1.2,4.8)	0.02*
	No	Ref		Ref	
	**HIV status**				
	Positive	1.7 (0.7,4.3)	0.25	1.4 (0.5,3.6)	0.54
	Negative	Ref		Ref	
	**Type of TB**				
	Smear-positive pulmonary TB	1.6 (0.4,6.7)	0.50	1.2 (0.2,5.6)	0.84
	Smear-negative pulmonary TB	Ref		Ref	
	Extrapulmonary TB	2.1 (0.5,8.4)	0.29	1.7 (0.4,7.2)	0.48
	**Category of TB treatment**				
	New (formerly Category 1)	Ref		Ref	
	Previously treatment (formerly Category 2)	1.5 (0.9,2.7)	0.14	1.4 (0.8,2.4)	0.30
**Mehra 2013 (Uttarakhand)[a,b] [14]**		Values below are odds ratios			
	**Sex**				
	Female	Ref			
	Male	1.00 (0.65,1.55)	0.99		
	**Age (Years)**				
	40 and above	Ref			
	18-40	1.27 (0.86,1.93)	0.26		
	**Type of residence**				
	Urban	Ref			
	Rural	2.72 (1.72,4.30)*	<0.0001*		
	**Education**				
	Literate	Ref			
	Illiterate	4.4 (2.55,7.61)*	<0.0001*		

	**Socioeconomic status**				
	Middle	Ref			
	Low	1.35 (0.86,2.10)	0.19		
	**Treatment category**				
	Category II	Ref			
	Category I	1.06 (0.71,1.59)	0.77		
	**Smoking status**				
	Non-smoker	Ref			
	Smoker	1.11 (0.71,1.74)	0.65		
	**Smokeless tobacco use status**				
	Non-user	Ref			
	User	1.96 (1.31,2.95)*	0.0012*		
	**Alcohol use**				
	Non-alcoholic	Ref			
	Alcoholic	1.00 (0.66,1.52)	0.99		
**Pardeshi 2018 (multi-state)[a] [15]**		Values below are odds ratios		Values below are adjusted odds ratios	
	**Sex**				
	Male	Ref		Ref	
	Female	1.38 (1.04,1.84)	0.03	1.24 (0.93,1.67)	0.15
	**Age (Years)**				
	21-30	Ref		Ref	
	0-10	3.2 (1.51,6.81)*	0.002*	3.43 (1.52,7.77)*	0.003*
	11 to 20	1.06 (0.47,2.36)	0.89	1.25 (0.56,2.79)	0.59
	31-40	1.77 (1.12,2.82)*	0.02	1.67 (1.05,2.65)*	0.03*
	41-50	1.68 (1.01,2.81)*	0.04*	1.50 (0.88,2.57)	0.14
	51-60	1.44 (0.89,2.35)	0.14	1.24 (0.70,2.19)	0.45
	60 and above	1.82 (1.15,2.87)*	0.01*	1.59 (0.96,2.63)	0.07
	**Type of residence**				
	Urban	Ref		Not included in the multivariable model	
	Rural	1.19 (0.82,1.72)	0.36	Not included in the multivariable model	
	**Education**				
	Secondary	Ref		Ref	
	No education or preschool	2.42 (1.57,3.69)*	0.00*	1.82 (1.10,3.01)*	0.02*
	Primary	1.10 (0.61,2.01)	0.75	0.89 (0.45,1.79)	0.76
	Higher than secondary	1.74 (0.88,3.44)	0.11	2.01 (0.91,4.45)	0.08
	**Wealth index**				

	Rich	Ref		Ref	
	Middle	1.37 (0.76,2.44)	0.29	1.38 (0.73,2.59)	0.32
	Poor	2.0 (1.19,3.37)*	0.009*	1.86 (1.01,3.34)*	0.04*
	**Household structure**				
	Non-nuclear	Ref		Not included in the multivariable model	
	Nuclear	0.87 (0.65,1.17)	0.37	Not included in the multivariable model	
	**Type of HCP where household members usually go for other illness**				
	Public health services	Ref		Ref	
	Other health services	1.75 (1.31,2.33)*	0.00*	1.69 (1.26,2.26)*	0.00*
**Selvaraju 2023 (Tamil Nadu)[a,b] [19]**		Values below are odds ratios			
	Did not receive WhatsApp outreach after TB diagnosis	Ref			
	Received WhatsApp (i.e., cellphone) outreach after diagnosis	0.025 (0.002,0.415)	0.01*		
**Singh 2020 (Uttarakhand)[a,b] [20]**		Values below are odds ratios			
	**Method of Case Finding**				
	Active Case Finding	125.3 (7.43,2112.52)	0.0008*		
	Passive Case Finding	Ref			
**Thomas 2018 (Tamil Nadu)[b] [1]** *Outcome of this analysis is non-registration in the TB program*		Values below are odds ratios		Values below are adjusted odds ratios	
	**Sex**				
	Male	Ref		Ref	
	Female	0.88 (0.45,1.72)	0.7	1.33 (0.59,2.88)	0.49
	**Age (Years)**				
	18-35	Ref		Ref	
	36-50	1.34 (0.65,2.77)	0.43	0.97 (0.44,2.20)	0.93
	51 and above	2.88 (1.42,5.85)*	0.003*	2.94 (1.40,6.49)*	0.04*
	**From Chennai**				
	Yes	Ref		Ref	
	No	1.25 (0.66,2.37)	0.48	1.18 (0.56,2.38)	0.66
	**Trackability**				
	Probably trackable	Ref		Ref	
	Possibly trackable	1.78 (1.05,3.04)*	0.03*	1.66 (0.91,3.05)	0.1
	Untrackable	3.16 (1.06,9.46)*	0.04*	4.49 (1.29,15.06)*	0.02*

	**Prior TB treatment history**				
	No prior TB treatment history	Ref		Ref	
	Prior TB treatment history	3.54 (2.08,6.05)*	<0.0001*	3.88 (2.15,7.09)*	<0.0001*
	**Site of initial microscopy test**				
	Moderate or low volume patient microscopy center	Ref		Ref	
	High volume patient microscopy center	2.91 (1.59,5.30)*	0.0005*	3.18 (1.69,6.32)*	0.0002*
**Thomas 2018 (Tamil Nadu)[b] [1]** *Outcome of this analysis is non-initiation of TB therapy*		Values below are odds ratios		Values below are adjusted odds ratios	
	**Sex**				
	Male	Ref		Ref	
	Female	0.59 (0.22,1.58)	0.3	0.82 (0.26,2.21)	0.7
	**Age (Years)**				
	18-35	Ref		Ref	
	36-50	1.42 (0.52,3.89)	0.49	0.99 (0.35,3.09)	0.99
	51 and above	3.20 (1.23,8.30)*	0.02*	2.70 (1.06,7.84)*	0.04*
	**From Chennai**				
	Yes	Ref		Ref	
	No	3.20 (1.57,6.51)*	0.0013*	3.01 (1.37,6.52)*	0.007*
	**Trackability**				
	Probably trackable	Ref		Ref	
	Possibly trackable	1.89 (0.94,3.78)	0.07	1.45 (0.68,3.08)	0.33
	Untrackable	3.94 (1.13,13.70)*	0.03	4.53 (1.08,16.52)*	0.04*
	**Prior TB treatment history**				
	No prior TB treatment history	Ref		Ref	
	Prior TB treatment history	1.62 (0.82,3.23)	0.17	1.79 (0.83,3.77)	0.13
	**Site of initial microscopy test**				
	Moderate or low volume patient microscopy center	Ref		Ref	
	High volume patient microscopy center	2.04 (0.96,4.32)	0.06	2.02 (0.94,4.68)	0.07
**Tripathy 2013 (Punjab)[a,b] [21]**		Values below are odds ratios			
	**Distance to DMC**				
	Distance less than 10 km	Ref			
	Distance more than 10 km	192.82 (40.96,907.59)*	<0.0001*		
Drug-resistant TB					
**Jain 2018 (Madhya Pradesh)[a,b] [23]**					

	**Distance from drug-resistant TB treatment center**	Study reported that residing a distance of >150 kilometers from the drug-resistant TB treatment center associated with increased risk of PTLFU (i.e., non-initiation of treatment >19 days after diagnosis), but data were not reported to allow calculation of odds ratio or other effect estimates.	0.009*		
**Shewade 2017 (Tamil Nadu)[c] [25]**		Values below are relative risk ratios			
	**Sex**				
	Female	Ref			
	Male	1.4 (0.6,3.1)			
	**Age (Years)**				
	45-64	Ref			
	<14	Not reported (no participants in this category)			
	14-44	1.3 (0.7,2.4)			
	65 and above	Not reported (no participants in this category)			
	**Presumptive drug-resistant TB criteria** (i.e., criteria for drug-susceptibility testing)				
	Follow-up smear positive	Ref			
	Retreatment	2.0 (0.5,7.1)			
	New patient with TB/HIV	Not reported			
	**Site of TB**				
	Pulmonary smear positive	Ref			
	Extrapulmonary TB	Not reported			
	Pulmonary smear negative	Not reported			
	Pulmonary missing	Not reported			
	**Type of health facility**				
	District level	Ref			
	Primary/Secondary	1.2 (0.5,2.6)			
	Medical college/others	Not reported (no participants in this category)			
	**Quarter**				
	Jan-March 2014	Ref			
	April-Jun 2014	14.5 (1.9,107.9)*	<0.05*		

	Jul-Sep 2014	18.5 (2.6,129.9)*	<0.05*		
	Oct-Dec 2014	18.5 (2.6,133.3)*	<0.05*		
**Shewade 2018 (Gujarat)[c] [26]**		Values below are relative risk ratios		Values below are adjusted relative risk ratios	
	**Sex**				
	Male	Ref		Ref	
	Female	1.2 (0.5,3.0)		1.2 (0.6,2.8)	
	**Age (Years)**				
	15-44	Ref		Ref	
	<15	Not reported (no participants in this category)		Not reported (no participants in this category)	
	45-64	1.0 (0.4,2.8)		1.5 (0.7,3.4)	
	65 and above	1.5 (0.2,9.9)		2.4 (0.3,17.4)	
	**Presumptive MDR TB criteria**				
	Retreatment	Ref		Ref	
	Follow-up smear positive	5.1 (2.1,12.2)*		6.0 (2.3,15.2)*	
	New patient with TB/HIV	Not reported (no participants in this category)		Not reported (no participants in this category)	
	Pulmonary TB with MDRTB contact	Not reported (no participants in this category)		Not reported (no participants in this category)	
	**Site of TB**				
	Pulmonary smear positive	Ref		Ref	
	Pulmonary smear missing	3.2 (1.2,8.3)*		17.1 (7.7,39.3)*	
	Extrapulmonary TB	Not reported (no participants in this category)		Not reported (no participants in this category)	
	Pulmonary smear negative	Not reported (few participants in this category)		Not reported (few participants in this category)	
	**Health facility**				
	Primary/Secondary	Ref		Ref	
	District level	Not reported (few participants in this category)		Not reported (few participants in this category)	
	Medical college/others	2.0 (0.5,7.8)		0.7 (0.3,1.5)	
**Singla 2014 (Delhi)[a,b] [27]**		Values below are odds ratios			
	**Line probe assay**				
	After line probe assay implementation	Ref			
	Before line probe assay implementation	4.69 (3.15,6.97)*	<0.0001*		
Pediatric TB – drug-susceptible					
**Raizada 2018** (multi-state)[a,b,d] **[28]**		Values below are odds ratios			
	**Sex**				
	Female	Ref			

	Male	1.11 (0.87,1.41)	0.41		
	**Age (Years)**				
	10 to 14	Ref			
	0-4	2.81 (2.14,3.68)*	<0.0001*		
	5 to 9	1.33 (0.97,1.82)	0.08		
	**Sector**				
	Public	Ref			
	Private	0.70 (0.44,1.11)	0.13		
Pediatric TB – drug-resistant					
**Raizada 2018 (**multi-state)[a,b,d] **[28]**		Values below are odds ratios			
	**Sex**				
	Female	Ref			
	Male	1.56 (0.79,3.10)	0.20		
	**Age (Years)**				
	10 to 14	Ref			
	0-4	3.33 (1.44,7.73)*	0.0051*		
	5 to 9	1.49 (0.64,3.49)	0.37		
	**Sector**				
	Public	Ref			
	Private	2.33 (0.87,6.23)	0.09		

PTLFU, pre-treatment loss to follow-up; N/A, not applicable; HCP, healthcare provider; km, kilometer; HIV, human immunodeficiency virus; MDR, multidrug-resistant
[a]Effect estimates comprise odds ratios or adjusted odds ratios.
[b]Unadjusted odds ratios and p-values were estimated by the systematic review team from the raw data, as these were not provided in the original study.
[c]Effect estimates comprise relative risk or adjusted relative risk ratios.
[d]Study reported starting or registering in treatment as the outcome, so effect estimates (odds ratio or relative risk and 95% confidence interval) were flipped to show effect estimates for the outcome of not starting or registering in treatment.
*Indicates statistical significance at the 5% level.

References

1. Thomas BE, Subbaraman R, Sellappan S, Suresh C, Lavanya J, Lincy S, et al. Pretreatment loss to follow-up of tuberculosis patients in Chennai, India: a cohort study with implications for health systems strengthening. BMC Infect Dis. 2018;18: 142. doi:10.1186/s12879-018-3039-3

2. Subbaraman R, Nathavitharana RR, Satyanarayana S, Pai M, Thomas BE, Chadha VK, et al. The Tuberculosis Cascade of Care in India's Public Sector: A Systematic Review and Meta-analysis. PLoS Med. 2016;13: e1002149. doi:10.1371/journal.pmed.1002149

3. Ahmed J, Chadha VK, Singh S, Venkatachalappa B, Kumar P. Utilization of RNTCP services in rural areas of Bellary District, Karnataka, by gender, age and distance from health centre. Indian J Tuberc. 2009;56: 62–68.

4. Balasubramanian R, Garg R, Santha T, Gopi PG, Subramani R, Chandrasekaran V, et al. Gender disparities in tuberculosis: report from a rural DOTS programme in south India. Int J Tuberc Lung Dis. 2004;8: 323–332.

5. Dandona R, Dandona L, Mishra A, Dhingra S, Venkatagopalakrishna K, Chauhan LS. Utilization of and barriers to public sector tuberculosis services in India. Natl Med J India. 2004;17: 292–299.

6. Das M, Pasupuleti D, Rao S, Sloan S, Mansoor H, Kalon S, et al. GeneXpert and Community Health Workers Supported Patient Tracing for Tuberculosis Diagnosis in Conflict-Affected Border Areas in India. Trop Med Infect Dis. 2019;5. doi:10.3390/tropicalmed5010001

7. Dave P, Nimavat P, Shah A, Pujara K, Patel P, Modi B. Knowing more about initial default among diagnosed sputum smear-positive pulmonary tuberculosis patients in Gujarat, India [Abstract PC-868-03]. Int J Tuberc Lung Dis. 2013;17: S469.

8. Gopi P, Chandrasekaran V, Subramani R, Narayanan P. Failure to initiate treatment for tuberculosis patients diagnosed in a community survey and at health facilities under a DOTS program in a district of south India. Indian J Tuberc. 2005;52: 153–156.

9. Ismail IM, Kibballi Madhukeshwar A, Naik PR, Nayarmoole BM, Satyanarayana S. Magnitude and Reasons for Gaps in Tuberculosis Diagnostic Testing and Treatment Initiation: An Operational Research Study from Dakshina Kannada, South India. J Epidemiol Glob Health. 2020;10: 326–336. doi:10.2991/jegh.k.200516.001

10. Khandekar J, Acharya AS, R TH, Sharma A. Do patients with tuberculosis referred from a tertiary care referral centre reach their peripheral health institution? Natl Med J India. 2013;26: 332–334.

11. Kumar S. A retrospective cohort study of the magnitude of initial default among sputum smear-positive TB patients diagnosed at NITRD New Delhi, 4th quarter 2012. New Delhi, India; 2013.

12. Mandal A, Basu M, Das P, Mukherjee S, Das S, Roy N. Magnitude and reasons of initial default among new sputum positive cases of pulmonary tuberculosis under RNTCP in a district of West Bengal, India. South East Asia Journal of Public Health. 2014;4: 41–47. doi:10.3329/seajph.v4i1.21839

13. Majella MG, Thekkur P, Kumar AM, Chinnakali P, Saka VK, Roy G. Effect of mobile voice calls on treatment initiation among patients diagnosed with tuberculosis in a tertiary care hospital of Puducherry: A randomized controlled trial. J Postgrad Med. 2021;67: 205–212. doi:10.4103/jpgm.JPGM_1105_20

14. Mehra D, Kaushik RM, Kaushik R, Rawat J, Kakkar R. Initial default among sputum-positive pulmonary TB patients at a referral hospital in Uttarakhand, India. Trans R Soc Trop Med Hyg. 2013;107: 558–565. doi:10.1093/trstmh/trt065

15. Pardeshi G, Deluca A, Agarwal S, Kishore J. Tuberculosis patients not covered by treatment in public health services: findings from India's National Family Health Survey 2015-16. Trop Med Int Health. 2018;23: 886–895. doi:10.1111/tmi.13086

16. Pillai D, Purty A, Prabakaran S, Singh Z, Soundappan G, Anandan V. Initial default among tuberculosis patients diagnosed in selected medical colleges of Puducherry: issues and possible interventions. 2015;4: 957-960. doi:10.5455/IJMSPH.2015.30012015196

17. Rawat J, Biswas D, Sindhwani G, Kesharwani V, Masih V, Chauhan BS. Diagnostic defaulters: an overlooked aspect in the Indian Revised National Tuberculosis Control Program. J Infect Dev Ctries. 2012;6: 20–22. doi:10.3855/jidc.1895

18. Sai Babu B, Satyanarayana AVV, Venkateshwaralu G, Ramakrishna U, Vikram P, Sahu S, et al. Initial default among diagnosed sputum smear-positive pulmonary tuberculosis patients in Andhra Pradesh, India. Int J Tuberc Lung Dis. 2008;12: 1055–1058.

19. Selvaraju S, Malaisamy M, Dolla CK, Murali L, Karikalan N, Saravanan B, et al. Application of mobile phone technology as intervention for the management of tuberculosis patients diagnosed through community survey. Indian J Med Res. 2022;155: 301–305. doi:10.4103/ijmr.IJMR_75_20

20. Singh M, Sagili KD, Tripathy JP, Kishore S, Bahurupi YA, Kumar A, et al. Are Treatment Outcomes of Patients with Tuberculosis Detected by Active Case Finding Different From Those Detected by Passive Case Finding? J Glob Infect Dis. 2020;12: 28–33. doi:10.4103/jgid.jgid_66_19

21. Tripathy JP, Srinath S, Naidoo P, Ananthakrishnan R, Bhaskar R. Is physical access an impediment to tuberculosis diagnosis and treatment? A study from a rural district in North India. Public Health Action. 2013;3: 235–239. doi:10.5588/pha.13.0044

22. Chadha SS, Sharath BN, Reddy K, Jaju J, Vishnu PH, Rao S, et al. Operational challenges in diagnosing multi-drug resistant TB and initiating treatment in Andhra Pradesh, India. PLoS One. 2011;6: e26659. doi:10.1371/journal.pone.0026659

23. Jain S, Varudkar HG, Julka A, Singapurwala M, Khosla S, Shah B. Socio-economical and Clinico-Radiological Profile of 474 MDR TB Cases of a Rural Medical College. J Assoc Physicians India. 2018;66: 14–18.

24. Natrajan S, Singh AR, Shewade HD, Verma M, Bali S. Pre-diagnosis attrition in patients with presumptive MDR-TB in Bhopal, India, 2015: a follow-up study. Public Health Action. 2018;8: 95–96. doi:10.5588/pha.18.0015

25. Shewade HD, Nair D, Klinton JS, Parmar M, Lavanya J, Murali L, et al. Low pre-diagnosis attrition but high pre-treatment attrition among patients with MDR-TB: An operational research from Chennai, India. J Epidemiol Glob Health. 2017;7: 227–233. doi:10.1016/j.jegh.2017.07.001

26. Shewade HD, Shringarpure KS, Parmar M, Patel N, Kuriya S, Shihora S, et al. Delay and attrition before treatment initiation among MDR-TB patients in five districts of Gujarat, India. Public Health Action. 2018;8: 59–65. doi:10.5588/pha.18.0003

27. Singla N, Satyanarayana S, Sachdeva KS, Van den Bergh R, Reid T, Tayler-Smith K, et al. Impact of introducing the line probe assay on time to treatment initiation of MDR-TB in Delhi, India. PLoS One. 2014;9: e102989. doi:10.1371/journal.pone.0102989

28. Raizada N, Khaparde SD, Salhotra VS, Rao R, Kalra A, Swaminathan S, et al. Accelerating access to quality TB care for pediatric TB cases through better diagnostic strategy in four major cities of India. PLoS One. 2018;13: e0193194. doi:10.1371/journal.pone.0193194
