# Supplementary material for: Barriers to engagement in the care cascade for tuberculosis disease in India: A systematic review of quantitative studies
Source: PLoS Med. 2024 May 28;21(5):e1004409. doi: 10.1371/journal.pmed.1004409 (PMC11166313; doi:10.1371/journal.pmed.1004409)
Supplement: S4 Appendix — (PDF) [file pmed.1004409.s004.pdf]

S4 Appendix. Methods and study characteristics for the systematic review of barriers to achieving treatment success in people with TB disease (Gap 4)

Supplement to:
Barriers to engagement in the care cascade for tuberculosis disease in India: a systematic review of quantitative studies

Authors:
Tulip A. Jhaveri, Disha Jhaveri, Amith Galivanche, Maya Lubeck-Schricker, Dominic Voehler, Mei Chung, Pruthu Thekkur, Vineet Chadha, Ruvandhi Nathavitharana, Ajay M.V. Kumar, Hemant Deepak Shewade, Katherine Powers, Kenneth H. Mayer, Jessica E. Haberer, Paul Bain, Madhukar Pai, Srinath Satyanarayana, Ramnath Subbaraman

Correspondence:
Ramnath Subbaraman, MD, MSc, FACP
Tufts University School of Medicine
Department of Public Health and Community Medicine
136 Harrison Ave., MV120
Boston, MA 02130, USA
Email: ramnath.subbaraman@tufts.edu

Table of Contents

Methods ***3***

Objectives 3

Search strategy 4

Table A. Search strategy to identify manuscripts regarding tuberculosis (TB) patients not achieving treatment success in India (Gap 4). This same search was also used to identify relevant articles for Gaps 2 and 3. 5

Inclusion and exclusion criteria 5

Study selection 6

Fig A. PRISMA flowchart: study selection for the systematic review of non-completion of the diagnostic workup, pretreatment loss to follow-up, and on-treatment loss to follow-up for TB patients in India (Gaps 2, 3 and 4) 7

Quality assessment of quantitative studies 8

Table B. Criteria for assessing quality of quantitative studies evaluating failure of diagnosed TB patients not getting successfully registered in treatment. 9

Data extraction and analysis 9

Table C. Characteristics of the included studies for patients who did not achieve treatment success (Gap 4) *11*

Table D. Factors associated with patients diagnosed with tuberculosis not achieving treatment success (Gap 4) *42*

References ***181***

Methods

Objectives

The objective of this systematic review was to understand why some people with tuberculosis (TB) who start and get registered for TB treatment do not achieve treatment success (Gap 4 in the care cascade). We included patients who started TB treatment either in India's National TB Elimination Programme (NTEP) or at private sector facilities. We included studies that evaluated patient outcomes starting from TB treatment initiation. Favorable outcomes in this analysis were assumed to include both cure and treatment completion, which together are referred to as "treatment success." Unfavorable outcomes in this analysis generally comprised the standard case definitions, as defined by India's NTEP and the World Health Organization (WHO), for the following outcomes during the treatment period: loss to follow-up, treatment failed, died, not evaluated, or a composite of more than one of these. In addition, situations in which studies reported loss to follow-up of less than two months or nonadherence to medications using other measures, we also analyzed these separately as unfavorable outcomes representing medication nonadherence. The protocol was registered in PROSPERO in April 2020 under ID CRD42020159361.

Given the large number of studies identified in this review, as well as the heterogeneity in treatment outcomes based on the type of TB, we report our data using subgroups related to drug-resistance profile or proxy measures previously used to presume risk of drug resistance in the NTEP (e.g., "new" patients versus patients with a prior TB treatment history; drug-susceptible versus drug-resistant patients). We also analyzed studies that only included people with human immunodeficiency virus (HIV) or pediatric patients as separate subgroups. We break down our findings as follows:

(a) *New TB patients*: These studies evaluated outcomes among patients with a new diagnosis of active TB disease at any site, including pulmonary or extrapulmonary TB with or without a bacteriological diagnosis. Up until the last few years, "new" patients were often presumed to have drug-susceptible TB and treated with a standardized regimen of isoniazid, rifampin, pyrazinamide, and ethambutol. Note that studies of sputum smear-positive patients treated using a "Category 1" regimen or smear-negative or extrapulmonary patients treated using a "Category 3" regimen, under prior nomenclature, are included in this subgroup.

(b) *Patients with a prior TB treatment history*: These studies evaluated outcomes among patients with a prior TB treatment history at any site, including pulmonary or extrapulmonary TB with or without a bacteriological diagnosis. These patients were categorized separately for a few reasons. Up until about 10 to 15 years ago, these patients were treated in the NTEP using a "Category 2" regimen, which included isoniazid, rifampin, pyrazinamide, and ethambutol, along with the addition of streptomycin by injection. Over the last decade or more, patients with a prior TB treatment history were supposed to undergo drug-susceptibility testing given their elevated risk for having drug-resistant TB. Notably, even with increased drug-susceptibility testing in recent years, these patients continue to have considerably poorer treatment outcomes in the NTEP than new patients.

(c) *Drug-resistant TB patients*: These studies evaluated outcomes among patients confirmed to have rifampin-resistant TB (generally diagnosed using Xpert MTB/Rif),

isoniazid-resistant TB, or multi-drug resistant (MDR) TB (generally diagnosed using culture or line probe assay).

(d) *Multiple populations*: These studies evaluated involved cohort studies that included more that one of the subgroups above and did not report outcomes separately for these subgroups.

(e) *People with HIV treated for active TB*: These studies evaluated outcomes among people with HIV who were being treated for active TB disease.

(f) *Pediatric TB patients*: These studies reported outcomes separately for children with TB.

We extracted two types of quantitative findings that help to understand reasons for unfavorable TB treatment outcomes:

(a) *Factors associated with unfavorable treatment outcomes in regression analyses*: For studies comparing individuals who had favorable versus unfavorable outcomes, we extracted effect estimates for independent variables (i.e., exposures or predictors) associated with experiencing an unfavorable treatment outcome. Effect estimates included odds ratios, risk ratios, hazard ratios, or beta-coefficients, depending on the approach to analysis.

(b) *Reasons reported by individuals for experiencing unfavorable treatment outcomes in quantitative surveys*: For studies that surveyed patients who experienced an unfavorable treatment outcome (or family members of patients who had died), we extracted the proportion of individuals who reported a given reason for an unfavorable outcome.

Search strategy

Three separate searches were conducted to identify articles. The first search was conducted as part of a previously published study quantifying gaps in India's TB care cascade [1]. We used articles identified for that review that evaluated Gap 4 in the TB care cascade but that also reported factors and reasons associated with not achieving treatment success. For that review, a medical librarian searched PubMed, Embase, and Web of Science for studies published between January 1, 2000 and February 26, 2015, without language restrictions, using search terms and related variants for "tuberculosis", "India", and "loss to follow-up", including "treatment success" and "treatment failure" to include losses after treatment initiation (Table A). We also carried out electronic searches of key Indian journals that were not indexed for that entire time window: the Indian Journal of Tuberculosis, Lung India, the Indian Journal of Chest and Allied Sciences, the India Journal of Public Health, and the Indian Journal of Community Medicine. Additional studies were identified by searching reference lists of the primary studies and review articles. Notably, given similarities in the search terms for identifying losses during the diagnostic workup (Gap 2), pretreatment loss to follow-up (Gap 3), and poor outcomes on treatment (Gap 4), this single search was used to identify studies related to all of these gaps. We screened all identified studies from this previous review for potential inclusion in our current review; however, studies met inclusion criteria for the current review only if they included information on reasons for not achieving TB treatment success.

To update our review, we conducted a second refresher search using the same search terms for October 2, 2015 to October 1, 2019. We did not repeat hand searches of the Indian journals

listed above, because all of these journals had been indexed in PubMed prior to the time period of this more recent search. Due to the extensive time required to extract data from the articles identified for this systematic review, we performed a third refresher search using the same search terms for October 2, 2019 to August 14, 2023. Finally, additional studies were identified by looking through the reference lists of the included primary studies and relevant review articles that were identified by the searches and by outreach to experts in the field.

Table A. Search strategy to identify manuscripts regarding people with tuberculosis (TB) not achieving treatment success in India (Gap 4). This same search was also used to identify relevant articles for Gaps 2 and 3.

Terms for tuberculosis:	"tuberculosis"[Mesh] OR *Mycobacterium tuberculosis*[tiab] OR TB[tiab] OR MDRTB[tiab] OR XDRTB[tiab]
Terms for India:	"India"[Mesh] OR India[tiab] OR India[ad] OR Indian[tiab] OR Indians[tiab]
Terms for loss to follow-up or other poor outcomes:	"patient dropouts"[tiab] OR "treatment refusal"[Mesh] OR "patient compliance"[Mesh] OR lost to follow up[tiab] OR loss to follow up[tiab] OR default*[tiab] OR compliance[tiab] OR adherence[tiab] OR noncompliance[tiab] OR nonadherence[tiab] OR patient cooperation[tiab] OR dropout*[tiab] OR linkage to care[tiab] OR retention[tiab] OR attrition[tiab] OR cascade of care[tiab] OR treatment cascade[tiab] OR treatment success*[tiab] OR treatment completion[tiab] OR cure[tiab] OR pretreatment loss to follow-up[tiab] OR initial default[tiab]; treatment failure [tiab]

Inclusion and exclusion criteria

We applied the following criteria for inclusion and exclusion of studies for this systematic review.

Inclusion criteria included the following:

(1) Studies that followed patients who were started on TB treatment to evaluate whether these patients achieved treatment success, or, conversely, whether they experienced suboptimal treatment outcomes, including loss to follow-up, treatment failed, not evaluated, or death. These studies had to have been conducted in programmatic settings—representing routine care in the public or private sector—and could have addressed any form of TB (i.e., TB at any body site, of any severity, or with any drug-resistance profile).
(2) Studies also had to have assessed reasons for individuals with TB in the study not achieving treatment success using a quantitative analysis. These studies could have compared characteristics of those who did or did not experience treatment success (e.g., regression analyses) or conducted structured interviews with patients who did not achieve treatment success to understand reasons for this outcome.

Exclusion criteria included the following:
(1) Studies that only described the proportion of patients who did or did not achieve treatment success without describing reasons why these outcomes occurred.

(2) Studies with data collected prior to the year 2000, as India's Revised National TB Control Programme (now called the NTEP) did not achieve nationwide coverage until the early 2000s.
(3) Studies only containing qualitative data evaluating inability to achieve treatment success. Findings from studies containing qualitative data will be reported in a separate paper.
(4) Studies that enrolled patients who would not be representative of the broader patient population, or in which study personnel were involved to help retain the patient in care, beyond what would have been provided in routine care. For example, we excluded randomized trials of new drug regimens, as these trials generally involve selection of patients who are more likely to adhere to treatment and mechanisms external to routine care for retaining these patients in care.

Study selection

Each citation identified by the search was independently assessed by at least two reviewers (among TJ, DJ, AG, DV, MLS and KP) for their eligibility at the title and abstract evaluation stage and again subsequently at the full text evaluation stage (Fig A). Disagreements between the two reviewers were resolved by discussion or, if necessary, through consultation of a third reviewer (RS). Independent selection of articles at the title and abstract and full text stages was conducted using Covidence software (Veritas Health Innovations, Melbourne, Australia); however, quality assessment and extraction of study findings was conducted using an Excel spreadsheet.

Fig A. PRISMA flowchart: study selection for the systematic review of non-completion of the diagnostic workup, pretreatment loss to follow-up, and on-treatment loss to follow-up for people with tuberculosis (TB) disease in India (Gaps 2, 3 and 4)

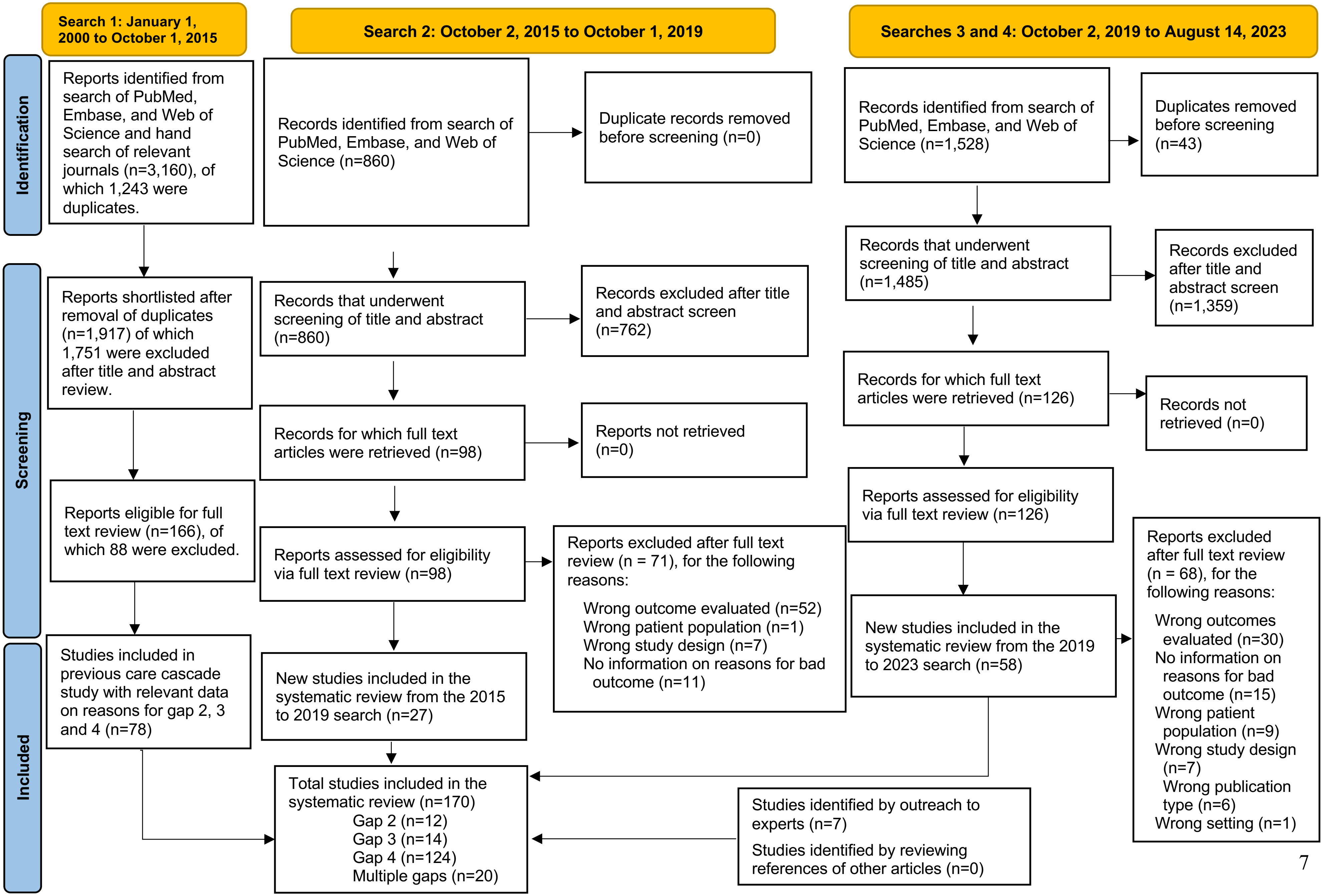

Quality assessment of quantitative studies

In our previous systematic review, we had developed quality criteria relevant to studies focused on identifying patients who did not complete the diagnostic workup for TB (Gap 2) or who were diagnosed with TB but did not get successfully started on, or registered in, TB treatment (Gap 3). Notably, in that prior review, we did not extract data from studies addressing patient outcomes during TB treatment (Gap 4), because district-, state-, and national-level treatment outcomes are routinely publicly reported by India's NTEP.

Given that the goal of the current review is to identify factors (or exposures) associated with unfavorable TB treatment outcomes, we could not rely on aggregate national-level reporting of TB treatment outcomes and had to review individual studies that evaluated exposures contributing to patient outcomes for Gap 4, as with other care cascade gaps. Notably, unlike with other gaps, studies contributing to Gap 4 were more likely to use cohort or case-control designs that may be more amenable to standardized quality evaluation approaches for non-randomized observational studies, such as the Newcastle-Ottawa scale.

However, we still used a more limited quality evaluation approach for Gap 4 for a few reasons. First, the overwhelming majority of studies included in this gap were not observational studies with specific inclusion and exclusion criteria designed to evaluate specific hypotheses; rather, most Gap 4 studies, whether conducted in the public or private sector, involved evaluation of exposures and outcomes under routine programmatic care. Second, given that our systematic review is focused on identifying exposures associated with unfavorable outcomes—rather than the outcomes themselves—we felt that study quality was often related to research approaches that were more likely to enrich the diversity of exposures characterized that might contribute to outcomes. For example, many Gap 4 studies retrospectively extracted data from routine programmatic records, which considerably limited the exposures available for analysis, especially given use of standardized records in the NTEP. In contrast, studies that prospectively collected baseline data directly from patients using structured surveys were able to explore the role of broader and more diverse exposures not captured in routine programmatic records. In addition, given challenges with recording of programmatic TB outcomes in many low- and middle-income country settings, studies that prospectively verify patient outcomes through triangulation with local TB clinic staff or patients themselves may be of higher quality.

As such, we systematically evaluated and reported findings on the following criteria that may provide further insights into the characteristics and quality of the included studies. We classified the health facility-based strategy for sampling patients in each study based on whether it was comprehensive, random, or convenience. Studies using convenience sampling were excluded from analysis. Notably, this was the only criterion that was used to exclude studies from this review, because we felt that non-random sampling was a major threat to the validity of study findings. For the remaining criteria below, we report them to allow readers to better understand factors that may shape the quality of individual studies but did not exclude studies from the review based on these criteria.

Studies that assessed more than 150 patients and at over one clinical site were rated as being higher in quality than studies that assessed less than 150 patients at a single clinical site. Studies that used prospective data collection were rates as being higher in quality than studies that only relied on retrospective data extraction from program records. Furthermore, for the reasons articulated above, we also report on whether studies more specifically used prospective data collection to assess exposures and/or outcomes.

Table B. Criteria for assessing quality of quantitative studies evaluating barriers to treatment success in people diagnosed with tuberculosis (TB) in India.

Criterion	Quality level
**Sampling strategy**	
Random or comprehensive sampling at selected facilities	High
Convenience sampling or not reported	Low (exclude findings from analysis)
**Sample size**	
>1 center and 150+ patients	High
Single center study with 150+ patients	Medium
<150 patients or not reported	Low
**Type of study design**	
Prospective data collection	High
Retrospective data collection	Medium to low
**Assessment of exposures**	
Patient interviews or tracking by a dedicated research team	High
Relying on extraction from medical records alone (limited exposures available)	Medium to low
**Assessment of outcomes**	
Patient interviews or tracking by a dedicated research team	High
Relying on extraction from medical records alone	Medium to low

Data extraction and analysis

Multiple reviewers (TJ, AG, DV, MLS, and DJ) independently extracted data from each included study into a structured form on an Excel spreadsheet; however, we ensured that every article had data independently extracted by at least two or more reviewers. Disagreements were resolved by discussion or, if necessary, by consulting a supervising reviewer (RS). From each study, we extracted information on the study design, location, setting (i.e., urban versus rural), sample size, quality measures (e.g., prospective or retrospective data collection), and variables of interest (Table C).

For studies that compared patients who did or did not achieve treatment success or experience medication nonadherence, we extracted adjusted and unadjusted effect estimates (odds ratios, risk ratios, hazard ratios, and beta-coefficients) from regression analyses. For studies that did not report effect estimates, we calculated unadjusted odds ratios from the data provided, if possible. For studies that reported reasons why patients experienced unfavorable treatment outcomes, we extracted the proportion of patients surveyed who reported a given reason for unfavorable outcomes. For effect estimates and proportions, we extracted information on 95% confidence intervals (95% CIs) where available; if 95% CIs were not reported, we calculated these from the data provided, if possible.

For some variables, we also changed the reference group as needed for consistency of reporting across studies. For example, because most studies compared men to the reference group of women, we “flipped” effect estimates and confidence intervals for studies that

presented men as the reference group. This allowed us to consistently present women as the reference group for findings regarding sex.

After “flipping” selected effect estimates from the regression analyses, we reported all unadjusted and adjusted effect estimates, regardless of statistical significance, organized by study (Table D). For the main manuscript and Forest plot, we restricted ourselves to presenting statistically significant adjusted effect estimates from multivariable analyses, as these may represent more meaningful associations from higher-quality analyses. After extracting this subset of findings, we organized findings into categories using our framework of demand- and supply-side factors (main manuscript, Table 1). For findings on reasons why patients did not achieve treatment success or experienced medication nonadherence, we presented all reported proportions in the Forest Plots in the main manuscript; we also organized these findings using our framework of demand- and supply-side categories.

To visualize quantitative findings, we generated Forest plots of effect estimates odds ratios, risk ratios, hazard ratios, beta-coefficients, and proportions using Stata version 16.1 (College Station, TX, USA). We did not conduct meta-analyses of data, because we extracted findings representing a diverse set of variables from every study.

Table C. Characteristics of the included studies for patients with tuberculosis (TB) who did not achieve treatment success (Gap 4)

**Citation** (year)	**Location** (state/union territory)	**Urban, rural, or both**	**Public or private sector**	**Type of population**	**Type of unfavorable outcomes assessed**	**Single or multiple designated microscopy centers (DMCs)**	**Sample size**	**Methodology for assessing exposures and outcomes**	**Type of findings included in the study** (sample size for each type of analysis)
People with new drug-susceptible TB									
Ahmed (2009) [2]	Karnataka	Rural	Public sector	New sputum smear positive pulmonary TB patients	Death, treatment failure, loss to follow-up, and transferred out as a composite outcome	Multiple healthcare facilities	186	Retrospective data collection from the government TB program*	Logistic regression (N=186)[a]
Babiarz (2014) [3]	Bihar	Rural	Public sector	New TB patients (sputum smear positive pulmonary, sputum smear negative pulmonary, and extrapulmonary)	Loss to follow-up as a single outcome (i.e., treatment discontinuation <25 weeks after initiation)	Multiple healthcare facilities	811	Retrospective data collection on outcomes from the government TB program with follow-up patient interview for more information on exposures*	Logistic regression (N=811)
Bagchi (2010) [4]	Maharashtra	Urban	Public sector	New sputum smear positive pulmonary TB patients in the first two months of therapy	Medication non-adherence as a single outcome (i.e., at least one week's worth of missed TB medication doses in any treatment month)	Multiple healthcare facilities	100	Case-control study using retrospective data collection from the government TB program with cross-sectional data collection from patient interviews by a dedicated research team	Logistic Regression (N=100)
Balasubramanian (2004) [5]	Tamil Nadu	Rural	Public sector	New sputum smear positive pulmonary TB patients	Loss to follow-up as a single outcome	Multiple healthcare facilities	1,015	Prospective data collection for both outcomes and exposures using a dedicated research team	Logistic regression (N=1,015)
Barathi (2022) [6]	Puducherry and Tamil Nadu	Urban and rural	Public sector	New sputum smear positive pulmonary TB patients	Death, treatment failure, and emerging resistance as a composite outcome	Multiple healthcare facilities	712	Prospective data collection for both outcomes and exposures using a dedicated research team	Logistic regression (N=712)

Bhatt (2017) [7]	Tamil Nadu	Urban and rural	Public sector	New sputum smear positive pulmonary TB patients	(1) Death, treatment failure, and loss to follow-up as single outcomes; and (2) unfavorable treatment outcomes (i.e., not achieving treatment success) as a composite outcome	Single healthcare facility*	191	Prospective data collection from the government TB program with longitudinal patient follow-up and interviews by a dedicated research team	Logistic regression (N=191)
Chakrabarti (2012) [8]	West Bengal	Rural (tribal and non-tribal populations)	Public sector	New pulmonary TB patients (sputum smear positive pulmonary, sputum smear negative pulmonary, and extrapulmonary)	Death, treatment failure, loss to follow-up, and transferred out as a composite outcome	Single healthcare facility*	399	Retrospective data collection from the government TB program*	Logistic regression (N=399)[a]
Chen (2023) [9]	Himachal Pradesh	Urban and rural	Public sector	New pulmonary TB patients (sputum smear positive pulmonary, sputum smear negative pulmonary, and extrapulmonary)	Death, treatment failure, loss to follow-up, and treatment regimen changed as a composite outcome	Multiple healthcare facilities	16,044	Retrospective data collection from the government TB program*	Logistic regression (N=16,044)
Gopi (2006) [10]	Tamil Nadu	Rural	Public sector	New sputum smear positive pulmonary TB patients	Not achieving microbiological cure assessed by sputum smear microscopy	Single healthcare facility*	1,463	Prospective data collection from the government TB program with baseline interview of patients by a dedicated research team	Logistic regression (N=1,463)
Gopalan (2021) [11]	Tamil Nadu	Urban	Public sector	New sputum smear positive pulmonary TB patients	Death, treatment failure, and recurrences as a composite outcome	Single healthcare facility*	667	Retrospective data collection from the government TB program*	Logistic regression (N=667)[a]
Gupta (2022) [12]	Punjab	Urban	Public sector	New sputum smear positive pulmonary TB patients	Death, loss to follow-up, and treatment regimen changed as a composite outcome	Multiple healthcare facilities	197	Prospective data collection from the government TB program with baseline patient interviews by a dedicated research team	Logistic regression (N=197)[a]
Joseph (2011) [13]	Karnataka	Rural	Public sector	New sputum smear positive pulmonary TB patients	Treatment failure and loss to follow-up as a composite outcome	Multiple healthcare facilities	212	Retrospective data collection from the government TB program*	Logistic regression (N=212)[a]

Kulkarni (2013) [14]	Maharashtra	Urban	Public Sector	New sputum smear positive pulmonary TB patients	Medication nonadherence (i.e., interruption of treatment for >=1 month)	Multiple healthcare facilities	150	Prospective data collection from the government TB program with longitudinal patient follow-up and interviews by a dedicated research team	Logistic regression (N=150); Reasons for medication nonadherence (N=78)
Mave (2021) [15]	Maharashtra	Urban and rural	Public sector	New bacteriologically confirmed pulmonary TB patients (i.e., by sputum smear microscopy, Xpert MTB/RIF testing, or mycobacterial culture)	(1) Death as a single outcome, and (2) treatment failure as a single outcome	Multiple healthcare facilities	832	Prospective data collection for both exposures and outcomes by a dedicated research team	Logistic Regression (N=832)
Mukherjee (2009) [16]	West Bengal	Urban and rural	Public sector	New pulmonary TB patients (sputum smear positive and sputum smear negative)	Death, treatment failure, and loss to follow-up as a composite outcome	Multiple healthcare facilities	2,870	Retrospective data collection from the government TB program*	Logistic Regression (N=2,870)[a]
Mukherjee (2012) [17]	West Bengal	Rural	Public sector	New sputum smear positive pulmonary TB patients	Death, treatment failure, and loss to follow-up as a composite outcome	Multiple healthcare facilities	1,393	Retrospective data collection from the government TB program*	Logistic regression (N=1,393)[a]
Prajapati (2023) [18]	Gujarat	Urban	Public sector	New TB patients (sputum smear positive pulmonary, sputum smear negative pulmonary, and extrapulmonary)	Death, treatment failure, modification of therapy, and loss to follow-up as a composite outcome	Multiple healthcare facilities	7,314	Retrospective data collection from the government TB program*	Logistic regression (N=7,314)
Ramachandran (2020) [19]	Tamil Nadu and Maharashtra	Urban	Public sector	New pulmonary TB patients (sputum smear positive and sputum smear negative)	Treatment failure and death as single outcomes	Multiple healthcare facilities	404	Prospective data collection from the government TB program with baseline evaluation and then longitudinal follow-up of patients by a dedicated research team	Poisson regression (N=404)
Rouf (2021) [20]	Jammu and Kashmir	Urban	Public sector	New TB patients (sputum smear positive pulmonary, sputum smear	Death, treatment failure, loss to follow-up, and not evaluated as a composite outcome	Multiple healthcare facilities	202	Prospective data collection from the government TB program with baseline and follow-up patient interviews by a dedicated research team	Logistic regression (N=202)

				negative pulmonary, and extrapulmonary)					
Shameer (2016) [21]	Kerala	Not reporte d	Public sector	New TB patients (sputum smear positive pulmonary, sputum smear negative pulmonary, and extrapulmonary)	Medication nonadherence (i.e., missing 3 or more consecutive doses)	Multiple healthcare facilities	141*	Case-control study using retrospective data collection from the government TB program with cross-sectional data collection from patient interviews by a dedicated research team	Logistic regression (N=141)
Shewade (2019) [22]	Tamil Nadu, Kerala, Maharashtra , Madhya Pradesh, Chhattisgarh , Bihar, Jharkhand, West Bengal, Punjab	Urban and rural	Public sector	New sputum smear positive pulmonary TB patients	Death, treatment failure, loss to follow-up, and not evaluated as a composite outcome	Multiple healthcare facilities	572	Prospective data collection from the government TB program with baseline patient interviews by a dedicated research team	Logistic regression (N=572)
Shivam (2014) [23]	West Bengal	Rural	Public sector	New TB patients (sputum smear positive pulmonary, sputum smear negative pulmonary, and extrapulmonary)	Death, treatment failure, and loss to follow-up as a composite outcome	Multiple healthcare facilities	758	Retrospective data collection from the government TB program*	Logistic regression (N=758)[a]
Singla (2009) [24]	Delhi	Urban	Public sector	New sputum smear positive pulmonary TB patients	Treatment failure as a single outcome as compared to patients who achieved cure	Single healthcare facility*	118*	Prospective data collection from the government TB program with baseline patient interviews by a dedicated research team	Logistic regression (N=118)
Singla (2013) [25]	Delhi	Urban	Public sector	New sputum smear positive pulmonary TB patients	(1) Sputum smear positive at 2 months as a single outcome; (2) sputum culture positive at 2 months as a single outcome; and (3) death, treatment failure, and loss to follow-up as a composite outcome	Single healthcare facility*	148*	Prospective data collection from the government TB program with baseline patient interviews by a dedicated research team	Logistic regression (N=148)

Sinha (2023) [26]	Maharashtra, Tamil Nadu, and Puducherry	Urban	Public sector	New pulmonary TB patients (sputum smear positive and sputum smear negative)	(1) Death, treatment failure, loss to follow-up, and transferred out as a composite outcome; (2) Death as a single outcome; (3) treatment failure as a single outcome; and (4) loss to follow-up as a single outcome.	Multiple healthcare facilities	2,931	Prospective data collection for both exposures and outcomes by a dedicated research team	Logistic regression (N=2,931)
Tiwari (2012) [27]	Delhi	Urban	Public sector	New sputum smear positive pulmonary TB patients	Death, treatment failure, loss to follow-up, and transferred out as a composite outcome	Multiple healthcare facilities	338	Prospective data collection from the government TB program with baseline patient interviews by a dedicated research team	Logistic regression (N=338)[a]
Trivedi (2019) [28]	Gujarat	Urban and rural	Public sector	New sputum smear positive pulmonary TB patients	Not achieving cure (based on follow-up sputum microscopy) as a single outcome	Single healthcare facility*	76*	Prospective data collection from the government TB program with baseline patient interviews by a dedicated research team	Logistic regression (N=76)[a]
Umayorubhagom (2023) [29]	Karnataka	Urban	Public sector	New TB patients	Death, treatment failure, modification of therapy, and loss to follow-up as a composite outcome	Single healthcare facility*	261	Prospective data collection for both exposures and outcomes by a dedicated research team	Logistic regression (N=261)
Vashishtha (2013) [30]	Delhi	Urban	Public sector	New TB patients (sputum smear positive pulmonary, sputum smear negative pulmonary, and extrapulmonary) with and without HIV	Death, treatment failure, loss to follow-up, and treatment modified as a composite outcome	Single healthcare facility*	305 (150 with HIV and 155 without HIV)	Prospective data collection from the government TB program by a dedicated research team	Logistic regression (N=305)[a]
Velayutham (2014) [31]	Tamil Nadu	Urban and rural	Public sector	New TB patients (sputum smear positive pulmonary, sputum smear negative pulmonary, and extrapulmonary)	(1) Death as a single outcome; (2) treatment failure as a single outcome; and (3) loss to follow-up as a single outcome.	Multiple healthcare facilities	2,602	Retrospective data collection from the government TB program*	Logistic regression (N=2,602)
Velayutham (2014) [31]	Tamil Nadu	Urban and rural	Public sector	New sputum smear positive pulmonary TB patients	(1) Death as a single outcome; (2) treatment failure as a single outcome; and (3) loss to follow-up as a single outcome.	Multiple healthcare facilities	2,285	Retrospective data collection from the government TB program*	Logistic regression (N=2,285)

Velayutham (2018) [32]	Madhya Pradesh, Kerala, Delhi, Tamil Nadu, Maharashtra, Karnataka	Urban and rural	Public sector	New sputum smear positive pulmonary TB patients	Death, treatment failure, loss to follow-up, treatment modified, and not evaluated as a composite outcome	Multiple healthcare facilities	1,543	Prospective data collection from the government TB program with baseline and follow-up patient interviews by a dedicated research team	Logistic Regression (N=1,543)
Vijay (2010) [33]	Tamil Nadu, Kerala, Rajashan, Himachal Pradesh, Delhi, Manipur	Urban and rural	Public sector	New sputum smear positive pulmonary TB patients	Loss to follow-up as a single outcome	Multiple healthcare facilities	929	Case-control study using retrospective data collection from the government TB program with cross-sectional data collection from patient interviews by a dedicated research team	Logistic Regression (N=929)
Viswanathan (2014) [34]	Tamil Nadu	Urban and rural	Public sector	New sputum smear positive pulmonary TB patients	(1) Death, treatment failure, treatment modified, and loss to follow-up as a composite outcome; and (2) treatment failure and treatment modified as a composite outcome	Multiple healthcare facilities	245	Prospective data collection from the government TB program with baseline patient interviews by a dedicated research team	Logistic regression (N=245)[a] including diabetics and non-diabetes; Logistic regression (N=78)[a] including diabetics only
Viswanathan (2023) [35]	Tamil Nadu	Urban	Public sector	New sputum smear positive pulmonary TB patients	Death, treatment failure, relapse, and loss to follow-up as a composite outcome	Multiple healthcare facilities	569	Prospective data collection from the government TB program with baseline patient interviews by a dedicated research team	Logistic Regression (N=569)[a]
Zaman (2014) [36]	Assam	Not reported	Public sector	New sputum smear positive pulmonary TB patients	Death, treatment failure, and loss to follow-up as a composite outcome	Multiple healthcare facilities	54*	Prospective data collection from the government TB program with baseline and follow-up patient interviews by a dedicated research team	Logistic regression (N=54)[a] for death, treatment failure, or loss to follow-up as a composite outcome; Logistic regression (N=28)[a] for loss to follow-up as a single outcome among patients with irregular adherence; Reasons for medication nonadherence

									including loss to follow-up (N=28)
Zhou (2020) [37]	Puducherry, Tamil Nadu	Urban and rural	Public sector	New sputum smear positive pulmonary TB patients (confirmed by positive mycobacterial culture)	Loss to follow-up as a single outcome	Multiple healthcare facilities	425	Prospective data collection from the government TB program with baseline interviews by a dedicated research team	Logistic Regression (N=425)
People with drug-susceptible TB who have a prior TB treatment history									
Ahmed (2022) [38]	Karnataka	Urban and rural	Public sector	Previously treated smear positive pulmonary TB patients	Loss to follow-up as a single outcome	Multiple healthcare facilities	204	Retrospective data collection on outcomes from the government TB program with follow-up patient interview for more information on exposures*	Logistic Regression (N=204)
Babiarz (2014) [3]	Bihar	Rural	Public sector	Previously treated TB patients (sputum smear positive pulmonary, sputum smear negative pulmonary, and extrapulmonary)	Loss to follow-up as a single outcome (i.e., treatment discontinuation <25 weeks after initiation)	Multiple healthcare facilities	196	Retrospective data collection on outcomes from the government TB program with follow-up patient interview for more information on exposures*	Logistic Regression (N=196)
Bhagat (2010) [39]	Maharashtra	Urban	Public sector	Previously treated TB patients (sputum smear positive pulmonary, sputum smear negative pulmonary, and extrapulmonary)	Loss to follow-up as a single outcome	Multiple healthcare facilities	112*	Retrospective data collection from the government TB program*	Logistic Regression (N=97)
Chakrabarti (2012) [8]	West Bengal	Rural (tribal and non-	Public sector	Previously treated TB patients (sputum smear positive pulmonary, sputum	Death, treatment failure, loss to follow-up, and transferred out	Single healthcare facility*	75	Retrospective data collection from the government TB program*	Logistic Regression (N=75)[a]

		tribal populations)		smear negative pulmonary, and extrapulmonary)	as a composite outcome				
Chandrasekaran (2006) [40]	Tamil Nadu	Rural	Public sector	Previously treated smear positive pulmonary TB patients	Death, treatment failure, loss to follow-up, and others (e.g., transferred out) as a composite outcome	Multiple healthcare facilities	696	Retrospective data collection from the government TB program*	Logistic regression (N=696)[a]
Deepa (2013) [41]	Andhra Pradesh	Urban	Public sector	Previously treated sputum smear positive pulmonary TB patients	Death, treatment failure, loss to follow-up, and transferred out as a composite outcome	Multiple healthcare facilities	1,077	Retrospective data collection from the government TB program*	Relative risk regression (N=1,077)
Joseph (2011) [13]	Karnataka	Rural	Public sector	Previously sputum smear positive pulmonary TB patients	Treatment failure and loss to follow-up as a composite outcome	Multiple healthcare facilities	74*	Retrospective data collection from the government TB program*	Logistic Regression (N=74)[a]
Jha (2010) [42]	Nationally representative sample	Urban and rural	Public sector	Previously treated TB patients (sputum smear positive pulmonary, sputum smear negative pulmonary, and extrapulmonary)	Loss to follow-up as a single outcome compared to patients with treatment success or treatment failure	Multiple healthcare facilities	8,790	Case-control study using retrospective data from the government TB program*	Logistic Regression (N=2,330 for univariable analysis, N=1,160 for multivariable analysis)
Mukherjee (2009) [16]	West Bengal	Rural	Public sector	Previously sputum smear positive pulmonary TB patients	Death, treatment failure, loss to follow-up, and transferred out as a composite outcome	Multiple healthcare facilities	234	Retrospective data collection from the government TB program*	Logistic Regression (N=234)[a]
Nagaraja (2011) [43]	Andhra Pradesh	Not reported	Public sector	Previously treated patients who experienced treatment failure and were not placed on regimens to treat drug-resistant TB	Death, treatment failure, loss to follow-up, and transferred out as a composite outcome	Multiple healthcare facilities	202	Retrospective data collection from the government TB program*	Logistic Regression (N=202)[a]
Pardeshi (2010) [44]	Maharashtra	Urban	Public sector	Previously treated sputum smear positive pulmonary TB patients	Loss to follow-up as a single outcome	Multiple healthcare facilities	716	Retrospective data collection from the government TB program*	Logistic Regression (N=95)[a]

Prajapati (2023) [18]	Gujarat	Urban	Public sector	Previously treated TB patients (sputum smear positive pulmonary, sputum smear negative pulmonary, and extrapulmonary)	Death, treatment failure, modification of therapy, and loss to follow-up as a composite outcome	Multiple healthcare facilities	688	Retrospective data collection from the government TB program*	Logistic regression (N=688)
Sarpal (2014) [45]	Chandigarh	Urban	Public sector	Previously treated TB patients (sputum smear positive pulmonary, sputum smear negative pulmonary, and extrapulmonary)	Death, treatment failure, and loss to follow-up as a composite outcome	Multiple healthcare facilities	545	Prospective data collection from the government TB program with longitudinal patient follow-up and interviews by a dedicated research team	Logistic Regression (N=545)[a]
Sarpal (2014) [45]	Chandigarh	Urban	Public sector	Previously treated TB patients (sputum smear positive pulmonary, sputum smear negative pulmonary, and extrapulmonary)	Loss to follow-up as a single outcome	Multiple healthcare facilities	545	Prospective data collection from the government TB program with longitudinal patient follow-up and interviews by a dedicated research team	Logistic Regression (N=545);[a] Reasons for loss to follow-up (N=32)
Shivam (2014) [23]	West Bengal	Rural	Public sector	Previously treated TB patients (sputum smear positive pulmonary, sputum smear negative pulmonary, and extrapulmonary)	Death, treatment failure, and loss to follow-up as a composite outcome	Multiple healthcare facilities	149	Retrospective data collection from the government TB program*	Logistic Regression (N=149)[a]
Singla (2009) [24]	New Delhi	Urban	Public sector	Previously treated sputum smear positive pulmonary TB patients	Death, treatment failure, loss to follow-up, and transferred out as a composite outcome	Single healthcare facility*	38*	Prospective data collection from the government TB program with baseline patient interviews by a dedicated research team	Logistic Regression (N=38)[a] for all previously treated patients; logistic regression (N=19)[a] for previously treated patients with any drug resistance detected
Sisodia (2006) [46]	Rajasthan	Not reported		Previously treated sputum smear positive pulmonary TB patients	Death, treatment failure, loss to follow-up, and transferred out as a composite outcome	Multiple healthcare facilities	2,215	Prospective data collection from the government TB program by a dedicated research team	Logistic Regression (N=2,215)[a]

Srinath (2010) [47]	Andhra Pradesh	Not reported	Public sector	Previously treated TB patients, specifically focusing on "other" TB previously treated patients (generally sputum smear negative pulmonary or extrapulmonary patients)	Death, treatment failure, loss to follow-up, and transferred out as a composite outcome	Multiple healthcare facilities	4,067 previously treated TB patients, including 1,029 "other" previously treated patients	Retrospective data collection from the government TB program*	Logistic Regression (N=4,067)[a] for all previously treated patients; logistic regression (N=1,009) for "other" previously treated patients
Velavan (2018) [48]	Puducherry	Urban and rural	Public sector	Previously treated TB patients (sputum smear positive pulmonary, sputum smear negative pulmonary, and extrapulmonary)	Death, treatment failure, and loss to follow-up as a composite outcome	Multiple healthcare facilities	392	Retrospective data collection from the government TB program*	Logistic Regression (N=392)[a]
Velayutham (2014) [31]	Tamil Nadu	Urban and rural	Public sector	(1) All previously treated TB patients; and (2) previously treated sputum smear positive pulmonary TB patients	(1) Death as a single outcome; (2) treatment failure as a single outcome; and (3) loss to follow-up as a single outcome	Multiple healthcare facilities	803 previously treated TB patients, including 699 previously treated smear positive pulmonary TB patients	Retrospective data collection from the government TB program*	Logistic Regression (N=803) previously treated patients; logistic regression (N=699) previously treated smear positive pulmonary TB patients
People with drug resistant TB									

Bhatt (2018) [49]	New Delhi	Urban	Public sector	Rifampin-resistant TB patients confirmed by Xpert MTB/RIF or culture	(1) Death as a single outcome; (2) treatment failure as a single outcome; and (3) loss to follow-up as a single outcome	Single healthcare facility*	123*	Retrospective data collection from the government TB program*	Logistic Regression (N=123); Cox regression (N=123)
Dela (2017) [50]	Gujarat	Not reporte d	Public sector	Multidrug-resistant TB patients registered in the government program	Death, progression to extensively drug-resistant TB, loss to follow-up, and transfer out as a composite outcome	Single healthcare facility*	125*	Retrospective data collection from the government TB program*	Logistic Regression (N=125)[a]
Dash (2022) [51]	Odisha	Rural	Public sector	Multidrug-resistant TB patients registered in the government program	Death, loss to follow-up, failure to complete treatment regimen, and transferred out as a composite outcome	Single healthcare facility*	40*	Retrospective data collection from the government TB program*	Logistic Regression (N=40)[a]
Dole (2017) [52]	Maharashtra	Urban	Public sector	Rifampin-resistant TB patients confirmed by line probe assay or Xpert MTB/RIF	Death, treatment failure, and loss to follow-up as a composite outcome	Single healthcare facility*	146*	Retrospective data collection from the government TB program*	Logistic regression (N=146); Reasons for loss to follow-up (N=28)
Duraisamy (2014) [53]	Kerala	Urban and rural	Public sector	Multidrug-resistant TB patients confirmed by culture	(1) Death, treatment failure, progression to extensively drug-resistant TB, loss to follow-up, treatment interruption due to adverse drug reaction, and transferred out as a composite outcome; and (2) medication non-adherence (mean number of missed doses) as a single outcome	Multiple healthcare facilities	179	Retrospective data collection from the government TB program*	Cox regression (N=179)
Giri (2022) [54]	Bihar	Urban	Public sector	Multidrug-resistant pulmonary TB patients diagnosed by cartridge-based nucleic acid	Death, treatment failure, loss to follow-up, and transfer out as a composite outcome	Single healthcare facility*	268	Prospective data collection by clinical staff and a dedicated research team with data entered into an electronic medical record and separate clinical research database	Logistic Regression (N=268)[a]

				amplification testing and culture					
Isaakidis (2012) [55]	Maharashtra	Urban	Private sector (non-profit non-governmental organization)	Multidrug-resistant TB patients, confirmed by culture or diagnosed empirically, with HIV	Death, treatment failure, and loss to follow-up as a composite outcome	Single healthcare facility*	67*	Prospective data collection by clinical staff and a dedicated research team with data entered into an electronic medical record and separate clinical research database	Logistic Regression (N=67)
Jain (2014) [56]	Gujarat	Urban	Public sector	Multidrug-resistant TB patients registered in the government program and treated with a standardized regimen	Death, treatment failure, and loss to follow-up as a composite outcome	Single healthcare facility*	130*	Prospective data collection from the government TB program with baseline patient interview by dedicated research staff	Logistic Regression (N=130)[a]
Janmeja (2017) [57]	Chandigarh	Urban	Public sector	Rifampin resistant or multidrug-resistant TB diagnosed by cartridge-based nucleic acid amplification testing or culture, respectively	Death, treatment failure, modification of therapy, and loss to follow-up as a composite outcome	Single healthcare facility*	256	Retrospective data collection from the government TB program*	Logistic Regression (N=256)
Johnson (2022) [58]	Karnataka	Urban	Public sector	Multidrug-resistant TB patients diagnosed by cartridge-based nucleic acid amplification testing and culture	Death, treatment failure, loss to follow-up, and TB relapse as a composite outcome	Single healthcare facility*	462	Retrospective data collection from the government TB program*	Logistic Regression (N=462)
Kalagani (2022) [59]	Telangana	Urban	Public sector	Rifampin resistant or multidrug-resistant TB diagnosed by cartridge-based nucleic acid amplification testing or culture, respectively	(1) Death as a single outcome; (2) loss to follow-up as a single outcome	Single healthcare facility*	100*	Prospective data collection for both exposures and outcomes by a dedicated research team	Logistic Regression (N=100)

Kandi (2021) [60]	Telangana	Urban	Public sector	Multidrug-resistant TB patients registered in the government program	Death, treatment failure, switch to extensively drug-resistant TB treatment, and loss to follow-up as a composite outcome	Single healthcare facility*	377	Retrospective data collection from the government TB program*	Logistic Regression (N=377)[a]
Keshari (2023) [61]	New Delhi	Urban	Public sector	New isoniazid-resistant pulmonary TB diagnosed by line probe assay or culture	Death, treatment failure, modification of therapy, and loss to follow-up as a composite outcome	Single healthcare facility*	85*	Prospective data collection by clinical staff and a dedicated research team with data entered into an electronic medical record and separate clinical research database	Logistic Regression (N=85)[a]
Kiran B (2022) [62]	New Delhi	Urban	Public sector	Multidrug-resistant or rifampin-resistant TB registered in the government program who had completed a shorter muti-drug resistant (MDR) regimen or are already under/yet to be on a shorter MDR TB regimen in 1 year	(1) Death, modification of therapy, and loss to follow-up as a composite outcome; (2) Death as a single outcome	Single healthcare facility*	55*	Retrospective data collection on outcomes from the government TB program with follow-up patient interview for more information on exposures	Logistic Regression (N=55)[a]
Kumar R (2023) [63]	Tamil Nadu	Urban	Public sector	Multidrug-resistant or rifampin-resistant TB diagnosed by cartridge-based nucleic acid amplification testing and line probe assay	Death and loss to follow-up as a composite outcome	Single healthcare facility*	67*	Prospective data collection by clinical staff and a dedicated research team with data entered into an electronic medical record and separate clinical research database	Logistic Regression (N=67)[a]
Kumari (2022) [64]	Andhra Pradesh	Urban	Public sector	Multidrug-resistant TB diagnosed by cartridge-based nucleic acid amplification testing and line probe assay	Death, treatment failure, and loss to follow-up as a composite outcome	Single healthcare facility*	360	Prospective data collection by clinical staff and a dedicated research team with data entered into an electronic medical record and separate clinical research database	Logistic Regression (N=360)[a]
Lohiya (2020) [65]	New Delhi	Urban	Public sector	Extrapulmonary rifampin-resistant or multidrug-resistant TB diagnosed by cartridge-based nucleic acid amplification testing,	Death, treatment failure, modification of therapy, treatment discontinuation for reasons other than adverse drug reactions, and loss to	Multiple healthcare facilities	203	Retrospective data collection from the government TB program*	Relative Risk Regression (N=203)

				culture, or empiric diagnosis	follow-up as a composite outcome				
Nair (2016) [66]	Tamil Nadu	Urban and rural	Public sector	Multidrug-resistant or rifampin-resistant TB diagnosed by culture vs. line probe assay or Xpert MTB/RIF	Death, treatment failure, switched to extensively drug resistant TB treatment, interrupted treatment due to reasons other than adverse drug reaction, transfer out, and loss to follow-up as a composite outcome	Multiple healthcare facilities	524	Retrospective data collection from the government TB program*	Relative risk regression (N=524)
Natarajan (2020) [67]	Delhi	Urban and rural	Public sector	Multidrug-resistant or rifampin-resistant pulmonary TB who required the addition of bedaquiline with/without other newer/repurposed drugs	Medication non-adherence as a single outcome	Single healthcare facility*	275	Prospective data collection by clinical staff and a dedicated research team with data entered into an electronic medical record and separate clinical research database	Reasons for treatment interruption (N=275)
Parmar (2018) [68]	7 Indian states	Urban and rural	Public sector	Multidrug-resistant TB patients diagnosed by culture or line probe assay	(1) Death, treatment failure, and loss to follow-up as a composite outcome; (2) death as a single outcome; (3) treatment failure as a single outcome; and (4) loss to follow-up as a single outcome.	Multiple healthcare facilities	2,264	Retrospective data collection from the government TB program*	Logistic Regression (N=2,264)
Patel (2018) [69]	Gujarat	Urban	Public sector	Multidrug-resistant TB patients registered in the government program	Death, treatment failure (i.e., smear positivity, culture positivity, or smear/culture unavailability), and loss to follow-up as a composite outcome	Single healthcare facility*	145	Prospective data collection from the government TB program with follow-up interviews by dedicated research staff	Reasons for loss to follow-up (N=32)

Rupani (2020) [70]	Gujarat	Urban	Public sector	Multidrug-resistant TB patients diagnosed by cartridge-based nucleic acid amplification testing	Loss to follow-up (i.e., discontinuation of MDR TB treatment)	Single healthcare facility*	94*	Prospective data collection from the government TB program with follow-up interviews by dedicated research staff	Logistic Regression (N=94)
Saha (2017) [71]	Maharashtra	Urban	Private sector (for-profit hospital)	Multidrug-resistant TB, pre-extensively drug-resistant TB, or extensively drug-resistant TB diagnosed by Xpert MTB/RIF and culture	Death, treatment failure, and loss to follow-up as a composite outcome	Single healthcare facility*	59*	Retrospective data collection from medical record of private hospital*	Logistic regression (N=59)[a]
Sharma (2020) [72]	Delhi	Urban	Public sector	Multidrug-resistant TB patients registered in the government program	Death, treatment failure, switched to extensively drug-resistant TB therapy, treatment stopped due to adverse drug reaction, and loss to follow-up as a composite outcome	Multiple healthcare facilities	2,958	Retrospective data collection from the government TB program*	Logistic regression (N=2,958)
Shringarpure (2015) [72]	Gujarat	Urban and rural	Public sector	Multidrug-resistant TB patients registered in the government program	Loss to follow-up as a single outcome	Single healthcare facility*	322	Retrospective data collection from the government TB program*	Cox regression (N=322)
Velayutham (2022) [73]	Over 18 states	Urban and rural	Public sector	New isoniazid-resistant pulmonary TB diagnosed by line probe assay or culture	Death, treatment failure, modification of therapy, and loss to follow-up as a composite outcome	Multiple healthcare facilities	11,341	Retrospective data collection from the National TB Elimination program* followed by prospective data collection with follow-up interviews by dedicated research staff	Logistic regression (N=11,341)
Multiple populations of people with drug-susceptible TB (who have new TB or a prior TB									

treatment history)									
Ahmed (2021) [74]	Assam	Urban and rural	Public sector	New and previously treated TB patients (sputum smear positive pulmonary, sputum smear negative pulmonary, and extrapulmonary) as a combined population	Loss to follow-up as a single outcome	Multiple healthcare facilities	70	Prospective data collection by clinical staff and a dedicated research team with data entered into an electronic medical record and separate clinical research database	Reasons for treatment interruption (N=70)
Babiarz (2014) [3]	Bihar	Rural	Public sector	New and previously treated TB patients (sputum smear positive pulmonary, sputum smear negative pulmonary, and extrapulmonary) as a combined population	Loss to follow-up as a single outcome (i.e., treatment discontinuation <25 weeks after initiation)	Multiple healthcare facilities	1,007	Retrospective data collection on outcomes from the government TB program with follow-up patient interview for more information on exposures*	Logistic regression (N=1,007)
Bagchi (2010) [4]	Maharashtra	Urban	Public sector	New and previously treated sputum smear positive pulmonary TB patients, excluding new patients in the first two months of therapy	Medication non-adherence as a single outcome (i.e., at least one week's worth of missed TB medication doses in any treatment month)	Multiple healthcare facilities	438	Case-control study using retrospective data collection from the government TB program with cross-sectional data collection from patient interviews by a dedicated research team	Logistic regression (N=438)
Balasubramanian (2004) [5]	Tamil Nadu	Rural	Public sector	New and previously treated TB patients (sputum smear positive pulmonary, sputum smear negative pulmonary, and extrapulmonary) as a combined population	Loss to follow-up as a single outcome	Multiple healthcare facilities	2,371	Prospective data collection for both exposures and outcomes by a dedicated research team	Logistic regression (N=2,371); Reasons for loss to follow-up (N=1,086)

Banerjee (2020) [75]	West Bengal	Urban	Public sector	New and previously treated TB patients (pulmonary and extrapulmonary) as a combined population	Death, treatment failure, and loss to follow-up as a composite outcome	Multiple healthcare facilities	140*	Prospective data collection for both exposures and outcomes by a dedicated research team	Logistic regression (N=140)
Bhagyalaxmi (2010) [76]	Gujarat	Urban	Public sector	New and previously treated TB patients (pulmonary and extrapulmonary) as a combined population	Death, treatment failure, and loss to follow-up as a composite outcome	Multiple healthcare facilities	200	Prospective data collection for both exposures and outcomes by a dedicated research team	Logistic regression (N=200)[a]
Bhargava (2013) [77]	Chhattisgarh	Rural	Public sector	New and previously treated TB patients (sputum smear positive pulmonary, sputum smear negative pulmonary, and extrapulmonary) as a combined population	Death as a single outcome	Multiple healthcare facilities	1,695	Retrospective data collection from the government TB program with cross-sectional data collection from patient interviews by a dedicated research team	Logistic regression (N=1,695)
Brahmapurkar (2017) [78]	Chhattisgarh	Urban	Public sector	New and previously treated TB patients (sputum smear positive pulmonary, sputum smear negative pulmonary, and extrapulmonary) as a combined population	Death, treatment failure, loss to follow-up, and transferred out as a composite outcome	Multiple healthcare facilities	496	Retrospective data collection from the government TB program*	Logistic regression (N=496)[a]
Cox (2021) [79]	Maharashtra and Tamil Nadu	Urban and rural	Public sector	Men with drug-susceptible or presumed drug-susceptible pulmonary TB confirmed by Xpert MTB/RIF or culture or through empirical diagnosis for those with test-negative TB	Treatment failure as a single outcome	Multiple healthcare facilities	751	Prospective data collection for both exposures and outcomes by a dedicated research team	Logistic regression (N=751)

Dandona (2004) [80]	Andhra Pradesh, Maharastra, Rajasthan, Tamil Nadu	Urban and rural	Public sector	New and previously treated pulmonary TB patients as a combined population	Loss to follow-up as a single outcome	Multiple healthcare facilities	744	Prospective data collection for both exposures and outcomes by a dedicated research team	Logistic regression (N=744); Reported barriers to treatment completion among those who did and did not complete treatment (N=729)
Das (2014) [81]	Nagaland	Rural	Public sector with private sector support from a non-profit philanthro pic organizati on	New and previously treated TB patients (sputum smear positive pulmonary, sputum smear negative pulmonary, and extrapulmonary) as a combined population	Death, treatment failure, and loss to follow-up as a composite outcome	Single healthcare facility*	238	Retrospective data collection from the government TB program*	Logistic regression (N=238)[a]
Dey (2021) [82]	West Bengal	Urban	Private sector	New and previously treated TB patients as a combined population	Loss to follow-up as a single outcome	Multiple healthcare facilities	7,505	Retrospective data collection from the government TB program with cross-sectional data collection from patient interviews by a dedicated research team*	Logistic regression (N=7,505)[a] Reasons for loss to follow-up (N=377)
Gopi (2007) [83]	Tamil Nadu	Rural	Public sector	New and previously treated pulmonary TB patients (sputum smear positive and sputum smear negative) during the intensive phase of therapy as a combined population	Medication non-adherence as a single outcome (i.e., partial or complete non-observation of doses by healthcare providers during the intensive phase of therapy)	Multiple healthcare facilities	1,666	Prospective data collection for both exposures and outcomes by a dedicated research team	Logistic regression (N=1,666)
Gupta (2011) [84]	Delhi	Urban	Public sector	New and previously treated TB patients (including smear-positive pulmonary, smear-negative pulmonary, and extrapulmonary disease) with history of treatment	Medication non-adherence as a single outcome	Single healthcare facility*	201	Retrospective cohort-based analysis	Reasons for treatment interruption (N=201)

				interruption as a combined population					
Huddart (2021) [85]	Bihar	Both	Private sector	New and previously treated TB patients as a combined population	Death as a single outcome	Multiple healthcare facilities	4,000	Retrospective assessment of outcomes using phone surveys by a dedicated research team*	Logistic regression (N=4,000, with n=2,240 observed [i.e., answered the survey] and n=1,760 unobserved [i.e., did not answer the survey but accounted for using inverse probability weighting])
Islam (2023) [86]	West Bengal	Rural	Public sector	New and previously treated TB patients as a combined population	Medication non-adherence as a single outcome	Single healthcare facility*	82*	Retrospective data collection from the government TB program with cross-sectional data collection from patient interviews by a dedicated research team*	Logistic regression (N=82)
Jaggarajamma (2007) [87]	Tamil Nadu	Rural	Public sector	New and previously treated TB patients (sputum smear positive pulmonary, sputum smear negative pulmonary, and extrapulmonary) as a combined population	Loss to follow-up as a single outcome	Multiple healthcare facilities	1,124	Prospective data collection for both exposures and outcomes by a dedicated research team	Logistic regression (N=1,124);[a] Reasons reported by patients for loss to follow-up (N=141)
Jaiswal (2021) [88]	Chattisgharh	Urban	Public sector	New and previously treated TB patients who completed at least 1 month of TB therapy as a combined population	Medication non-adherence as a single outcome	Multiple healthcare facilities	55*	Cross-sectional data collection from patient interviews by a dedicated research team	Reasons for non-adherence (N=55)
Jan Swasthya Sahyog (2018) [89]	Chattisgharh	Rural	Private sector (non-profit community-based organization)	New and previously treated TB patients (sputum smear positive pulmonary, sputum smear negative pulmonary, and extrapulmonary) as a combined population	Death, treatment failure, and loss to follow-up as a composite outcome	Multiple healthcare facilities	4,979	Retrospective data collection from health system records of a community-based program*	Relative risk regression and logistic regression (N=2,607)[a]

Jonnalagada (2011) [90]	Andra Pradesh	Urban and rural	Public sector	New and previously treated TB patients (sputum smear positive pulmonary, sputum smear negative pulmonary, and extrapulmonary) as a combined population	Death, treatment failure, loss to follow-up, and transferred out as a composite outcome	Multiple healthcare facilities	8,240	Retrospective data collection from the government TB program*	Logistic regression (N=8,240)[a]
Joseph (2011) [13]	Karnataka	Rural	Public sector	New and previously treated sputum smear positive pulmonary TB patients as a combined population	Treatment failure and loss to follow-up as a composite outcome	Single healthcare facility*	286	Retrospective data collection from the government TB program*	Logistic regression (N=286)[a]
Kamble (2022) [91]	Haryana	Urban	Public sector	New and previously treated TB patients (sputum smear positive pulmonary, sputum smear negative pulmonary, and extrapulmonary) as a combined population	Death, treatment failure, loss to follow-up, shift to category IV, and transferred out as a composite outcome	Multiple healthcare facilities	5,257	Retrospective data collection from the government TB program*	Logistic regression (N=5,243)
Karanjekar (2014) [92]	Maharashtra	Rural	Public sector	New and previously treated TB patients (sputum smear positive pulmonary, sputum smear negative pulmonary, and extrapulmonary) as a combined population	Death, loss to follow-up, and transferred out as a composite outcome	Single healthcare facility*	125*	Prospective data collection for both exposures and outcomes by a dedicated research team	Logistic regression (N=125)[a]
Kumar (2018) [93]	Madhya Pradesh	Urban	Public sector	New and previously treated TB patients (sputum smear positive pulmonary, sputum smear negative pulmonary, and extrapulmonary) as a combined population	Death, treatment failure, loss to follow-up, and transferred out as a composite outcome	Multiple healthcare facilities	454	Retrospective data collection from the government TB program*	Logistic regression (N=454)

Kuruva (2020) [94]	Telangana	Urban	Public sector	New and previously treated TB patients (sputum smear positive pulmonary and sputum smear negative pulmonary) as a combined population	Death, treatment failure, and loss to follow-up as a composite outcome	Single healthcare facility*	126*	Prospective case-control data collection for both exposures and outcomes by a dedicated research team	Logistic Regression (N=126)[a]
Lata (2021) [95]	Jammu and Kashmir	Rural	Public sector	New and previously treated TB patients who completed at least 2 months of TB therapy as a combined population	Medication non-adherence as a single outcome (i.e., self-reported non-ingestion of at least one medication dose as measured by the Morisky adherence scale)	Single healthcare facility*	72*	Prospective data collection for both exposures and outcomes by a dedicated research team	Logistic regression (N=72);[a] Reasons for medication nonadherence (N=14)
Maroof (2022) [96]	Uttarakhand	Urban	Public sector	New and previously treated TB patients as a combined population who could be interviewed	Loss to follow-up as a single outcome	Single healthcare facility*	284	Retrospective data collection from the government TB program* prospective interview of these patients	Logistic regression (N=284)
Mittal (2011) [97]	Uttar Pradesh	Urban	Public sector	New and previously treated TB patients (sputum smear positive pulmonary, sputum smear negative pulmonary, and extrapulmonary) as a combined population	Death, treatment failure, loss to follow-up, and transferred out as a composite outcome	Multiple healthcare facilities	900	Retrospective data collection from the government TB program*	Logistic regression (N=900)[a]
Mittal (2011) [98]	Uttar Pradesh	Urban	Public sector	New and previously treated TB patients (sputum smear positive pulmonary, sputum smear negative pulmonary, and extrapulmonary) as a combined population	Loss to follow-up as a single outcome	Multiple healthcare facilities	900	Retrospective data collection on outcomes from the government TB program;* prospective interview of patients who became lost to follow-up	Logistic regression (N=900);[a] Reasons for loss to follow-up (N=111)

Motappa (2022) [99]	Karnataka	Urban	Public sector	New and previously treated TB patients (sputum smear positive pulmonary, sputum smear negative pulmonary, and extrapulmonary) as a combined population	Medication non-adherence as a single outcome (i.e., self-reported non-ingestion of at least one medication dose)	Single healthcare facility*	200	Retrospective data collection from the government TB program with cross-sectional data collection from patient interviews by a dedicated research team	Logistic regression (N=200)[a]
Mukhopadhyay (2011) [100]	West Bengal	Urban and rural	Public sector	New and previously treated TB patients (sputum smear positive pulmonary, sputum smear negative pulmonary, and extrapulmonary) as a combined population	Death, treatment failure, and loss to follow-up as a composite outcome	Multiple healthcare facilities	898	Retrospective data collection from the government TB program*	Logistic regression (N=898)[a]
Mundra (2017) [101]	Maharashtra	Urban and rural	Public sector	New and previously treated TB patients (sputum smear positive pulmonary, sputum smear negative pulmonary, and extrapulmonary) as a combined population	(1) Death, treatment failure, treatment modification, and loss to follow-up as a composite outcome; (2) death as a single outcome; (3) treatment failure as a single outcome; (4) treatment modification as a single outcome; and (5) loss to follow-up as a single outcome	Multiple healthcare facilities	503	Retrospective data collection from the government TB program*	Cox regression (N=503)
Mundra (2018) [102]	Maharashtra	Urban and rural	Public sector	New and previously treated TB patients (sputum smear positive pulmonary, sputum smear negative pulmonary, and extrapulmonary) as a combined population	Death, treatment failure, treatment modification, and loss to follow-up as a composite outcome	Single healthcare facility*	275	Case-control study using retrospective data collection from the government TB program with cross-sectional data collection from patient interviews by a dedicated research team	Logistic regression (N=275)
Nahar (2014) [103]	Madhya Pradesh	Urban	Public sector	TB patients being treated in the government TB program (no further	Loss to follow-up as a single outcome	Multiple healthcare facilities	Not fully clarified, presum	Case-control study using retrospective data collection from the government TB program with cross-sectional	Logistic regression (Sample size not fully clarified,

				description, but presumed to comprise new and previously treated patients)			ed to be 386	data collection from patient interviews by a dedicated research team	presumed to be N=386)
Nandakumar (2013) [104]	Kerala	Urban and rural	Public sector	New and previously treated TB patients (sputum smear positive pulmonary, sputum smear negative pulmonary, and extrapulmonary) as a combined population	Death, treatment failure, loss to follow-up, and transferred out as a composite outcome	Multiple healthcare facilities	3,116	Retrospective data collection from the government TB program*	Relative risk regression (N=3,116)
Nandi (2022) [105]	West Bengal	Urban	Public sector	New and previously treated sputum smear positive pulmonary TB patients as a combined population	Death, treatment failure, and loss to follow-up as a composite outcome	Multiple healthcare facilities	295	Retrospective data collection on outcomes from the government TB program; prospective interview of some patients registered during reference period	Logistic regression (N=295)[a]
Panati (2023) [106]	Andhra Pradesh	Urban	Public sector	New and previously treated TB patients as a combined population	Low to medium adherence as a single outcome	Single healthcare facility*	100*	Prospective data collection for both exposures collected by a dedicated research team with retrospective data on outcomes collected from the government TB program	Logistic regression (N=100)
Pardeshi (2007) [107]	Maharashtra	Urban	Public sector	New and previously treated sputum smear positive pulmonary TB patients as a combined population	Death, treatment failure, and loss to follow-up as a composite outcome	Single healthcare facility*	1,646	Retrospective data collection from the government TB program*	Logistic regression (N=1,646)[a]
Pardeshi (2010) [44]	Maharashtra	Urban	Public sector	New and previously treated sputum smear positive pulmonary TB patients as a combined population	Death, treatment failure, loss to follow-up, and transferred out as a composite outcome	Single healthcare facility*	1,925	Retrospective data collection from the government TB program*	Logistic regression (N=1,925)[a]
Patra (2013) [108]	Delhi	Urban	Public sector	New and previously treated TB patients (sputum smear positive pulmonary, sputum smear negative pulmonary,	Death, treatment failure, loss to follow-up, and transferred out as a composite outcome	Single healthcare facility*	2,401	Retrospective data collection from the government TB program*	Logistic regression (N=2,401)

				and extrapulmonary) as a combined population among individuals >=60 years old					
Pauniikar (2019) [109]	Maharashtra	Both	Public sector	New and previously treated TB patients (sputum smear positive pulmonary, sputum smear negative pulmonary, and extrapulmonary) as a combined population	Loss to follow-up as a single outcome	Single healthcare facility*	440	Prospective data collection for both exposures collected by a dedicated research team with retrospective data on outcomes collected from the government TB program	Cox regression (N=440)
Pore (2020) [110]	Maharashtra	Rural	Public sector	Presumed drug-susceptible new and previously treated TB patients and multidrug-resistant TB patients (sputum smear positive pulmonary, sputum smear negative pulmonary, and extrapulmonary) as a combined population	Medication non-adherence as a single outcome (i.e., missing one or more medications for 7 consecutive days anytime during the treatment period)	Single healthcare facility*	88*	Prospective data collection for both exposures and outcomes by a dedicated research team	Logistic regression (N=88);[a] Reasons for medication nonadherence (N=34)
Potty (2021) [111]	Karnataka and Telangana	Urban	Public sector	New and previously treated TB patients (sputum smear positive pulmonary, sputum smear negative pulmonary, and extrapulmonary) as a combined population	Death, treatment failure, loss to follow-up, and transferred out as a composite outcome	Multiple healthcare facilities	31,617	Retrospective data collection from the government TB program*	Logistic regression (N=2,760)
Potty (2023) [112]	Karnataka and Telangana	Urban	Public sector	New and previously treated TB patients (sputum smear positive pulmonary, sputum smear negative pulmonary, and extrapulmonary)	Death, treatment failure, modification of therapy, and loss to follow-up, as a composite outcome	Multiple healthcare facilities	30,706	Prospective data collection for both exposures and outcomes by a dedicated research team	Logistic regression (N=30,561)

				as a combined population					
Prudhivi (2019) [113]	Andhra Pradesh	Urban and rural	Public sector	New and previously treated pulmonary TB patients (sputum smear positive and sputum smear negative) as a combined population	Death, treatment failure, loss to follow-up, and transferred out as a composite outcome	Single healthcare facility*	1,113	Retrospective data collection from the government TB program*	Logistic regression (N=1,113)
Ratnesh (2020) [114]	Uttar Pradesh	Urban	Public sector	New and previously treated TB patients (sputum smear positive pulmonary, sputum smear negative pulmonary, and extrapulmonary) as a combined population	Loss to follow-up as a single outcome	Multiple healthcare facilities	2,010	Prospective data collection for both exposures and outcomes by a dedicated research team	Logistic regression (N=2,010)
Saini (2016) [115]	Chandigarh	Urban	Public sector	New and previously treated TB patients (sputum smear positive pulmonary, sputum smear negative pulmonary, and extrapulmonary) as a combined population	Death, treatment failure, loss to follow-up, and treatment regimen changed to Cat IV as a composite outcome	Multiple healthcare facilities	3,551	Retrospective data collection from the government TB program*	Logistic regression (N=3,551)[a]
Shabil (2019) [116]	Karnataka	Urban and rural	Public sector	New and previously treated TB patients as a combined population, without further description of the population	Loss to follow-up as a single outcome	Single healthcare facility*	90*	Prospective data collection for both exposures and outcomes by a dedicated research team	Logistic regression (N=90);[a] Reasons for loss to follow-up (N=12); Reasons for medication nonadherence (i.e., treatment interruption) (N=9)

Sharma (2003) [117]	Delhi	Urban	Public sector	New and previously treated TB patients (sputum smear positive pulmonary, sputum smear negative pulmonary, and extrapulmonary) as a combined population	Death, treatment failure, loss to follow-up, and transferred out as a composite outcome	Single healthcare facility*	67*	Retrospective data collection from the government TB program*	Logistic regression (N=67)[a]
Sharma (2021) [118]	West Bengal	Urban	Private sector	New and previously treated TB patients (sputum smear positive pulmonary, sputum smear negative pulmonary, and extrapulmonary) as a combined population	Death, treatment failure, treatment modified to drug-resistant TB therapy, loss to follow-up, and not evaluated as a composite outcome	Multiple healthcare facilities	2,347	Retrospective data collection from an electronic database created to capture outcomes on patients treated in the private sector*	Logistic regression (N=2,347)[a]
Shivam (2014) [23]	West Bengal	Rural	Public sector	New and previously treated TB patients (sputum smear positive pulmonary, sputum smear negative pulmonary, and extrapulmonary) as a combined population	Death, treatment failure, and loss to follow-up as a composite outcome	Single healthcare facility*	758	Retrospective data collection from the government TB program*	Logistic regression (N=758)
Siddiqui (2016) [119]	Delhi	Urban	Public sector	New and previously treated TB patients (sputum smear positive pulmonary, sputum smear negative pulmonary, and extrapulmonary) as a combined population	Death, treatment failure, treatment modified to drug-resistant TB therapy, and loss to follow-up as a composite outcome	Multiple healthcare facilities	316	Retrospective data collection from the government TB program*	Logistic regression (316)
Singh (2020) [120]	Uttarakhand	Urban	Public sector	New and previously treated TB patients (sputum smear positive pulmonary, sputum smear negative pulmonary, and extrapulmonary)	Death, treatment failure, treatment regimen modified, loss to follow-up, transferred out and not evaluated as a composite outcome	Multiple healthcare facilities	433	Retrospective data collection from the government TB program*	Logistic regression (N=238)

				as a combined population					
Sodhi (2023) [121]	Gujarat, Madhya Pradesh, Bihar, New Delhi, and Haryana	Urban	Private sector	New and previously treated TB patients (sputum smear positive pulmonary, sputum smear negative pulmonary, and extrapulmonary) as a combined population	Death, treatment failure, and loss to follow-up as a composite outcome	Multiple healthcare facilities	42,562	Retrospective data collection from Project JEET (a non-governmental organization engaging private sector TB physicians)*	Logistic regression (N=42,562)[a]
Subbaraman (2021) [122]	Tamil Nadu and Maharashtra	Urban	Public sector	New and previously treated TB patients (sputum smear positive pulmonary, sputum smear negative pulmonary, and extrapulmonary) as a combined population	Medication non-adherence as a single outcome	Multiple healthcare facilities	650	Prospective data collection for both exposures and outcomes by a dedicated research team	Logistic regression (N=650)<br>Reasons for medication nonadherence (N=167)
Vasantha (2008) [123]	Tamil Nadu	Rural	Public sector	New and previously treated TB patients (sputum smear positive pulmonary, sputum smear negative pulmonary, and extrapulmonary) as a combined population	Death as a single outcome	Multiple healthcare facilities	3,513	Prospective data collection for both exposures and outcomes by a dedicated research team	Cox regression (N=3,513)
Vashishtha (2013) [30]	Delhi	Urban	Public sector	New and previously treated TB patients (sputum smear positive pulmonary, sputum smear negative pulmonary, and extrapulmonary) as a combined population among people with and without HIV	Death, treatment failure, treatment regimen modified, and loss to follow-up as a composite outcome	Multiple healthcare facilities	305	Prospective data collection for both exposures and outcomes by a dedicated research team	Logistic regression (N=305)[a]

Vasudevan (2014) [124]	Puducherry	Urban	Public sector	New and previously treated TB patients (sputum smear positive pulmonary, sputum smear negative pulmonary, and extrapulmonary) as a combined population	Loss to follow-up as a single outcome	Multiple healthcare facilities	4,421	Retrospective data collection from the government TB program*	Logistic regression (N=4,421)[a]
Viswanathan (2014) [34]	Tamil Nadu	Urban	Public sector	New and previously treated TB patients (sputum smear positive pulmonary, sputum smear negative pulmonary, and extrapulmonary) as a combined population	Death, treatment failure, and loss to follow-up as a composite outcome	Multiple healthcare facilities	209	Retrospective data collection from the government TB program*	Logistic regression (N=209)[a]
Washington (2020) [125]	Karnataka and Telangana	Rural	Public sector with private sector support from a non-profit philanthro pic organizati on	Presumed drug-susceptible new and previously treated TB patients and multidrug-resistant TB patients (sputum smear positive pulmonary, sputum smear negative pulmonary, and extrapulmonary) as a combined population	(1) Death, treatment failure, and loss to follow-up as a composite outcome; and (2) death as a single outcome	Multiple healthcare facilities	4,749	Prospective data collection for both exposures and outcomes by a dedicated research team	Logistic regression (N=4,749)
Yadav (2019) [126]	Rajasthan	Urban and rural	Public sector	New and previously treated TB patients (including smear-positive pulmonary, smear-negative pulmonary, and extrapulmonary disease) with history of treatment interruption as a combined population	Medication non-adherence as a single outcome	Single healthcare facility*	150	Descriptive cross-sectional questionnaire-based	Reasons for medication nonadherence (i.e., treatment interruption) (N=9)

People with HIV treated for active TB									
Ambadekar (2015) [127]	Maharashtra	Urban and rural	Public sector	New and previously treated TB patients (including smear-positive pulmonary, smear-negative pulmonary, and extrapulmonary disease) with HIV	(1) Death, treatment failure, loss to follow-up, and transfer out as a composite outcome; and (2) death as a single outcome	Multiple healthcare facilities	886	Retrospective data collection from the government TB program*	Logistic Regression (N=886)
Madan (2018) [128]	New Delhi	Urban	Public sector	New and previously treated TB patients (including smear-positive pulmonary, smear-negative pulmonary, and extrapulmonary disease) with HIV	Death, treatment failure, loss to follow-up, transfer out, and switched to a MDR-TB treatment regimen as a composite outcome	Multiple healthcare facilities	816	Retrospective data collection from the government TB program*	Logistic Regression (N=816)
Maji (2022) [129]	New Delhi	Urban	Public sector	New and previously treated TB patients (including smear-positive pulmonary, smear-negative pulmonary, and extrapulmonary disease) with HIV	Death and loss to follow-up as a composite outcome	Single healthcare facility*	53*	Cross-sectional data collection from patient interviews by a dedicated research team	Logistic Regression (N=53)[a]
Ranganath (2021) [130]	Karnataka	Urban and rural	Public sector	New and previously treated TB patients (including smear-positive pulmonary, smear-negative pulmonary, and extrapulmonary disease) with HIV	Non-adherence as a single outcome	Multiple healthcare facilities	500	Case-control study using retrospective data collection from the government TB program with cross-sectional data collection from patient interviews by a dedicated research team	Logistic Regression (N=500)
Sharma (2014) [131]	New Delhi	Urban	Public sector	New and previously treated TB patients (including smear-positive pulmonary, smear-negative pulmonary, and	Death, treatment failure, and loss to follow-up as a composite outcome	Single healthcare facility*	431	Retrospective data collection from the government TB program*	Logistic Regression (N=431)

				extrapulmonary disease) with HIV					
Shastri (2013) [132]	Karnataka	Urban and rural	Public sector	New and previously treated TB patients (presumably including smear-positive pulmonary, smear-negative pulmonary, and extrapulmonary disease) with HIV	Death, treatment failure, and loss to follow-up as a composite outcome	Multiple healthcare facilities	5,079	Retrospective data collection from the government TB program*	Logistic Regression (N=5,040)[a]
Vijay (2011) [137]	Karnataka	Urban and rural	Public sector	New and previously treated TB patients (including smear-positive pulmonary, smear-negative pulmonary, and extrapulmonary disease) with HIV	(1) Death, treatment failure, and loss to follow-up as a composite outcome; and (2) death as a single outcome	Multiple healthcare facilities	281	Retrospective data collection from the government TB program* (a dedicated study team collected interviewed patients, but these data did not inform the regression analyses)	Logistic Regression (N=281)
Children with TB									
Dhakulkar (2021) [133]	Maharashtra	Urban	Public sector with collaborative support from the private sector (non-profit non-governmental organization)	Children (0-9 years old) and adolescents (10-19 years old) with multidrug-resistant TB, pre-extensively drug-resistant TB, and extensively drug-resistant TB	Death, treatment failure, and loss to follow-up as a composite outcome	Single healthcare facility*	268	Retrospective data collection from the government TB program*	Logistic Regression (N=268)

Raizada (2018) [134]	New Delhi, Tamil Nadu, Telangana, West Bengal	Urban	Public sector and private sector	Children (0-14 years old) with drug-susceptible TB diagnosed by Xpert MTB/RIF testing	Death as a single outcome	Multiple healthcare facilities	1,164	Retrospective data collection from the government TB program or private sector*	Logistic Regression (N=1,164)[a]
Sadana (2020) [135]	Punjab	Urban and rural	Public sector	Children aged 0-14 years old	Death, treatment modified, and loss to follow-up as a composite outcome in children <15 years age	Single healthcare facility*	62*	Evaluation of exposures through baseline interview by a dedicated research team and assessment of outcomes through retrospective data collection from the government TB program*	Logistic Regression (N=62)[a]
Satyanarayana (2010) [136]	New Delhi	Urban	Public sector	Children 0-14 years old	Death, treatment failure, loss to follow-up, amd transferred out as a composite outcome	Multiple healthcare facilities	1,074	Retrospective data collection from the government TB program*	Logistic Regression (N=1,074)[a]

TB, tuberculosis; HIV, human immunodeficiency virus
*Medium or low quality for this indicator
[a]Unadjusted odds ratios and/or p-values were estimated by the systematic review team from the raw data, as these were not provided in the original study.

Table D. Factors associated with patients diagnosed with tuberculosis (TB) not achieving treatment success (Gap 4)

Study and specific outcome	Exposure / Independent variable	Unadjusted Effect Estimate (95% Confidence Interval)	P-value	Adjusted Effect Estimate (95% Confidence Interval)	P-value
Studies of people with new drug-susceptible					
Ahmed, 2009[a] (Karnataka) *Outcome: Death, treatment failure, loss to follow-up, and transferred out as a composite outcome* [2]		Values below are odds ratios			
	**Sex**				
	Female	Ref			
	Male	2.16 (0.89,5.28)	0.09		
	**Distance from village of residence to treating health center**				
	<4 km	Ref			
	5-9 km	1.45 (0.6,3.5)	0.41		
	10-14 km	0.61 (0.13,2.92)	0.53		
	15-19 km	0.47 (0.06,3.92)	0.48		
	>20 km	4.5 (1.40,14.42)*	0.01*		
Babiarz, 2014 (Bihar) *Outcome: Loss to follow-up as a single outcome (i.e., treatment discontinuation <25 weeks after initiation)* [3]				Values below are adjusted odds ratios	
	**Sex**				
	Female			Ref	
	Male			1.34 (0.81,2.22)	
	**Age[c]**				
	Per each year increase in age			0.98 (0.93,1.03)	
	**Religion**				
	Non-Hindu			Ref	
	Hindu			0.97 (0.51,1.84)	
	**Caste/tribe**				
	Other			Ref	
	Scheduled caste/tribe/OBC			1.02 (0.51,2.02)	
	**Number of children in household[c]**				
	Per each increase in number of children			1.08 (0.89,1.31)	
	**Education**				

	Yes			Ref	
	No			1.01 (0.95,1.07)	
	**Poor**				
	No			Ref	
	Yes			1.27 (0.73,2.19)	
	**Middle income**				
	No			Ref	
	Yes			0.95 (0.56,1.61)	
	**Household size**[c]				
	Per each person increase in household			0.92 (0.81,1.04)	
	**Total weeks from symptom onset to treatment initiation**[c]				
	Per each week increase in symptoms			1.02 (0.97,1.08)	
	**Number of symptoms at treatment initiation**				
	>=5			Ref	
	2 or fewer			1.29 (0.76,2.21)	
	3 to 4			1.16 (0.65,2.09)	
	**Travel cost as a barrier**				
	No			Ref	
	Yes			4.39 (1.36,14.13)*	
	**Number of providers visited**[d]				
	Per each provider increased			5.62 (2.32,13.66)*	
	**Treatment or medication fees**				
	No			Ref	
	Yes			6.32 (1.43,28.0)*	
Bagchi, 2010[a] (Maharashtra) *Population: New sputum smear positive pulmonary TB patients in the first two months of therapy* *Outcome: Medication non-adherence as a single outcome (i.e., at least one week's worth of missed TB medication doses in any treatment month)* [4]		Values below are odds ratios		Values below are adjusted odds ratios	
	**Sex**				
	Female	Ref			
	Male	0.7 (0.2,2.4)	Not reported		
	**Household members**				

	0-3	Ref			
	>3	0.3 (0.0,2.7)	Not reported	0.3 (0.03,2.9)	Not reported
	**Smoking status**				
	Never smoked	Ref		Ref	
	Ever smoked	8.2 (1.4,46)*	Not reported	7.8 (1.2,49)*	Not reported
	**Tobacco chewing**				
	Never used	Ref		Ref	
	Ever used	2.0 (0.,6.9)	Not reported	1.6 (0.3,2.8)	Not reported
	**Alcohol use**				
	Never used	Ref			
	Ever used	1.3 (0.1,12.4)	Not reported		
	**Hid TB disease from family**				
	No	Ref			
	Yes	0.6 (0.1,3.1)	Not reported		
	**Treatment duration perceived by patient as being too long**	`			
	No	Ref		Ref	
	Yes	2.9 (0.9,9.9)	Not reported	1.4 (0.3,7.4)	Not reported
	**Treatment discontinued once symptoms resolved**				
	No	Ref		Ref	
	Yes	2.9 (0.8,11)	Not reported	0.9 (0.1,5.8)	Not reported
	**Knows about problems with stopping treatment early**				
	Yes	Ref		Ref	
	No	2.1 (0.6,6.7)	Not reported	0.8 (0.2,3.9)	Not reported
	**Feels confident about completing treatment**				
	Somewhat or very sure	Ref		Ref	
	Not at all	0.5 (0.1,1.8)	Not reported	0.6 (0.0,11)	Not reported
	**Travel mode to health center**				
	Walking	Ref			
	Other	4.7 (1.7,12)*	Not reported		
	**Travel to health center is a problem**				
	No	Ref			

	Yes	7.1 (1.6,31)*	Not reported		
	**Has concerns about transportation to the health center**				
	No	Ref		Ref	
	Somewhat or very concerned	4.3 (1.1,17)*	Not reported	0.9 (0.7,1.9)	Not reported
	**Concerned about distance to the health center**				
	No	Ref			
	Somewhat or very concerned	4.3 (1.1,17)*	Not reported		
	**Concerned about the time to reach the health center**				
	No	Ref			
	Somewhat or very concerned	4.2 (1.2,15)*	Not reported		
	**Costs-related barriers to travel to health center**				
	No	Not reported		Ref	
	Yes	Not reported	Not reported	5.1 (1.4,19)*	Not reported
	**Doctor communicated problems related to stopping medication early**				
	Yes	Ref		Ref	
	No	2.2 (0.8,5.9)	Not reported	2.8 (0.5,14)	Not reported
	**Where patient gets most TB information**				
	DOTS center	Ref		Ref	
	Other sources	2.0 (0.7,5.8)	Not reported	0.8 (0.1,6.9)	Not reported
Balasubramanian, 2004 (Tamil Nadu) *Outcome: Loss to follow-up as a single outcome* [5]		Values below are odds ratios		Values below are adjusted odds ratios	
	**Sex**				
	Female	Ref			
	Male	2.5 (1.4,4.3)*	<0.001*		
	**Age (years)**				
	<45	Ref			
	>=45	1.6 (1.1,2.3)*	<0.001*		
Barathi, 2022 (Puducherry and Tamil Nadu) *Outcome: Death, treatment failure, and emerging resistance as a composite outcome* [6]				Values below are adjusted odds ratios	

	**Sex**				
	Females			Ref	
	Males			2.48 (1.11,5.55)*	0.03*
	**Treatment adherence**				
	Compliant			Ref	
	Non-compliant			11.51 (6.68,19.84)*	<0.001*
Bhatt, 2017 (Tamil Nadu) *Outcome: Death as a single outcome* [7]		Values below are odds ratios			
	**Type of treatment**				
	DOTS	Ref			
	SAT	1.30 (0.37,4.62)			
Bhatt, 2017 (Tamil Nadu) *Outcome: Treatment failure as a single outcome* [7]		Values below are odds ratios			
	**Type of treatment**				
	DOTS	Ref			
	SAT	1.13 (0.27,4.66)			
Bhatt, 2017 (Tamil Nadu) *Outcome: Loss to follow-up as a single outcome* [7]		Values below are odds ratios			
	**Type of treatment**				
	DOTS	Ref			
	SAT	0.61 (0.25,1.50)			
Bhatt, 2017[b] (Tamil Nadu) *Outcome: Not achieving treatment success as a composite outcome* [7]		Values below are odds ratios			
	**Type of treatment**				
	DOTS	Ref			
	SAT	0.74 (0.36,1.51)			
Chakrabarti, 2012[a] (West Bengal) *Outcome: Death, treatment failure, loss to follow-up, and transferred out as a composite outcome* [8]		Values below are odds ratios			
	**Population**				
	Non-tribal	Ref			
	Tribal	1.14 (0.71,1.84)	0.58		
Chen, 2023 (Himachal Pradesh) *Outcome: Death, treatment failure, loss to follow-up, and treatment regimen changed as a composite outcome* [9]		Values below are odds ratios		Values below are adjusted odds ratios	
	**Age**				

	Per each year increase in age			1.04 (1.04,1.04)*	<0.001*
	**Sex**				
	Female or transgender			Ref	
	Male			1.29 (1.12,1.48)*	<0.001*
	**Site of disease**				
	Pulmonary			Ref	
	Extrapulmonary			0.88 (0.77,1.02)	0.08
	**District**				
	District 1			Ref	
	District 2			1.61 (0.98,2.62)	0.06
	District 3			0.96 (0.30,3.12)	0.95
	District 4			0.95 (0.57,1.61)	0.86
	District 5			0.83 (0.53,1.30)	0.41
	District 6			0.80 (0.36,1.77)	0.58
	District 7			0.77 (0.46,1.30)	0.33
	District 8			0.76 (0.47,1.24)	0.28
	District 9			0.75 (0.44,1.27)	0.28
	District 10			0.63 (0.37,1.09)	0.10
	District 11			0.61 (0.34,1.09)	0.10
	District 12			0.60 (0.38,0.96)*	0.03*
	**Pre/Post 99DOTS launch**				
	Pre-intervention	Ref			
	Post-intervention	1.02 (0.90,1.15)	0.76	1.03 (0.91,1.17)	0.64
Gopi, 2006[b] (Tamil Nadu) *Outcome: Not achieving microbiological cure as assessed by sputum microscopy* [10]		Values below are odds ratios		Values below are adjusted odds ratios	
	**Age (years)**				
	<45	Ref		Ref	
	>=45	1.8 (1.4,2.3)*	<0.001*	1.5 (1.1,2.1)*	<0.05*
	**Sex**				
	Male	Ref		Ref	
	Female	0.42 (0.29,0.59)*	<0.001*	0.71 (0.45,1.14)	
	**Education**				
	Illiterate	Ref		Ref	
	Literate	0.77 (0.59,1)*	<0.05*	0.83 (0.59,1.18)	
	**Cough**				
	Cough >=4 weeks	Ref		Ref	

	Cough <4 weeks	0.63 (0.45,0.86)*	<0.01*	0.77 (0.53,1.12)	
	**Smear grade**				
	High	Ref		Ref	
	Low	0.71 (0.56,0.91)*	<0.01*	0.91 (0.66,1.24)	
	**Conversion**				
	Yes	Ref		Ref	
	No	4.1 (3.1,5.5)*	<0.001*	3.5 (2.6,4.8)*	<0.05*
	**Body weight**				
	>40 kg	Ref			
	<=40 kg	0.83 (0.63,1.11)	0.2		
	**Smoking**				
	Yes	Ref		Ref	
	No	0.42 (0.32,0.55)*	<0.001*	0.91 (0.62,1.33)	
	**Alcoholism**				
	No	Ref		Ref	
	Yes	2.7 (2.1,3.6)*	<0.001*	1.7 (1.2,2.4)*	<0.05*
	**Patient delay**				
	<4 weeks	Ref			
	>=4 weeks	0.83 (0.65,1.08)	0.3		
	**Diagnosis**				
	Community survey	Ref			
	Health facility	0.83 (0.63,1.11)	0.3		
Gopalan, 2021 (Tamil Nadu) *Outcome: Death, treatment failure, and recurrences as a composite outcome cure as assessed by sputum microscopy* [11]		Values below are hazard ratios			
	**Baseline Radiological Parameter (Cavity and Smear)**				
	No Cavity, Smear 1+	Ref			
	No Cavity, Smear 2+	1.05 (0.46,2.42)	0.91		
	No Cavity, Smear 3+	1.94 (0.81,4.64)	0.14		
	Cavity, Smear 1+	1.30 (0.28,6.18)	0.74		
	Cavity, Smear 2+	1.92 (0.80,4.60)	0.14		
	Cavity, Smear 3+	3.26 (0.133,8.00)*	0.01*		
	**Baseline Radiological Parameter (Number of Zones and Smear)**				
	≤2 zones, Smear 1+	Ref			
	≤2 zones, Smear 2+	1.04 (0.35,3.03)	0.95		

	≤2 zones, Smear 3+	2.26 (0.74,6.95)	0.15		
	>2 zones, Smear 1+	1.55 (0.45,5.37)	0.49		
	>2 zones, Smear 2+	1.92 (0.73,5.08)	0.19		
	>2 zones, Smear 3+	3.05 (1.13,8.24)*	0.03*		
	**Radiological Clearance and Smear Conversion at the end of IP**				
	Reduction <50% without smear conversion	Ref			
	Reduction <50% with smear conversion	0.30 (0.15,0.59)*	<0.001*		
	Reduction ≥50% without smear conversion	0.44 (0.22,0.86)*	0.02*		
	Reduction ≥50% with smear conversion	0.26 (0.14,0.49)*	<0.001*		
	**Radiological Clearance and Culture Conversion at the end of IP**				
	Reduction <50% without culture conversion	Ref			
	Reduction <50% with culture conversion	0.10 (0.05,0.19)*	<0.001*		
	Reduction ≥50% without culture conversion	0.63 (0.30,1.30)	0.22		
	Reduction ≥50% with culture conversion	0.05 (0.02,0.10)*	<0.001*		
Gupta, 2022 (Punjab) *Outcome: Death, loss to follow-up, and treatment regimen changed as a composite outcome* [12]		Values below are odds ratios			
	**Tobacco Consumption**				
	Non-Consumer	Ref			
	Consumer	3.11 (1.13,8.59)*	0.03*		
	**Smoking**				
	Non-Smoker	Ref			
	Smoker	3.68 (1.33,10.23)*	0.01*		
	**Exposure to Environmental Tobacco Smoke**				
	Absent	Ref			
	Present	1.44 (0.28,7.36)	0.66		
	**Number of packs smoked**				
	<1 pack	Ref			
	≥1 pack	3.65 (0.40,33.24)	0.25		
	**Age of start of smoking**				
	≥18 years	Ref			
	<18 years	2.89 (0.60,14.02)	0.19		
	**Years of Smoking**				

	0-5	Ref			
	5-10	0.16 (0.005,4.87)	0.29		
	10-15	0.16 (0.005,4.87)	0.29		
	15-20	0.33 (0.010,11.34)	0.54		
	>20	1.40 (0.12,15.98)	0.79		
Joseph, 2011[a] (Karnataka) *Outcome: Treatment failure and loss to follow-up as a composite outcome* [13]		Values below are odds ratios			
	**Sex**				
	Female	Ref			
	Male	1.31 (0.60,2.85)	0.5		
	**Age (years)**				
	<=30	Ref			
	30-60	1.71 (0.77,3.78)	0.18		
	>60	5.27 (1.78,15.65)*	0.003*		
	**Residence**				
	Urban	Ref			
	Rural	1.25 (0.63,2.46)	0.52		
Kulkarni, 2013 (Maharashtra) *Outcome: Medication nonadherence as a single outcome* [14]		Values below are odds ratios			
	**Age (years)**				
	Other than 15-49	Ref			
	15-49	1.56 (0.93,2.63)	0.06		
	**Sex**				
	Female	Ref			
	Male	2.51 (1.51,4.18)*	<0.001*		
	**Migrant**				
	No	Ref			
	Yes	1.97 (1.38,2.82)*	<0.001*		
	**Education**				
	Post-high school diploma or more	Ref			
	Literate with any schooling until high school	18.33 (1.02,328.99)*	0.05*		
	Illiterate	16.82 (0.89,317.71)	0.06		
	**Employment**				
	Unemployed	Ref			
	Employed	1.63 (1.17,2.27)*	0.004*		

	**Social class**				
	II	Ref			
	III	2.62 (0.82,8.39)	0.1		
	IV and V	6.32 (2.53,15.84)*	0.0001*		
	**Female sex worker**				
	Not female sex worker	Ref			
	Female sex worker	4.89 (2.73,8.76)*	0.001*		
	**Smoking**				
	No	Ref			
	Yes	1.88 (1.41,2.50)*	<0.001*		
	**Alcohol consumption**				
	No	Ref			
	Yes	1.85 (1.38,2.48)*	<0.001*		
	**Knowledge about importance of regular treatment**				
	Satisfactory	Ref			
	Unsatisfactory	2.20 (1.64,2.95)*	<0.001*		
	**Living with own family**				
	Yes	Ref			
	No	3.20 (1.93,5.30)*	<0.001*		
Mave, 2021 (Maharashtra) *Outcome: Death as a single outcome* [15]		Values below are hazard ratios		Values below are adjusted hazard ratios	
	**Diabetes**				
	TB only without diabetes mellitus	Ref		Ref	
	Any diabetes mellitus	5.06 (2.26,11.35)*	<0.001*	4.36 (1.62,11.76)*	0.004*
	Newly diagnosed diabetes mellitus	7.17 (2.67,19.27)*	<0.001*	6.56 (2.18,19.71)*	0.001*
	Known diabetes mellitus before TB diagnosis	4.20 (1.70,10.33)*	0.002*	3.14 (1.03,9.61)*	0.045*
	Diabetes mellitus being treated with metformin	3.30 (1.18,9.28) *	0.02*	2.32 (0.67,8.08)	0.2
	Diabetes mellitus not being treated with metformin	7.13 (2.96,17.21)*	<0.001*	6.17 (2.24,17.04)*	<0.001*
Mave, 2021 (Maharashtra) *Outcome: Treatment failure as a single outcome* [15]		Values below are odds ratios		Values below are adjusted odds ratios	
	**Diabetes**				
	TB only without diabetes mellitus	Ref		Ref	
	Any diabetes mellitus	0.56 (0.30,1.06)	0.08	0.75 (0.36,1.58)	0.46
Mukherjee, 2009[a] (West Bengal)		Values below are odds ratios			

Outcome: Death, treatment failure, loss to follow-up, and transferred out as a composite outcome [16]					
	**Sputum smear status**				
	New sputum smear negative pulmonary TB patients	Ref			
	New sputum smear positive pulmonary TB patients	0.72 (0.58,0.89)*	0.002*		
Mukherjee, 2012[a] (West Bengal) *Outcome: Death, treatment failure, and loss to follow-up as a composite outcome* [17]		Values below are odds ratios			
	**Sex**				
	Female	Ref			
	Male	1.44 (0.95,2.22)	0.09		
Prajapati, 2023 (Gujarat) *Outcome: Death, treatment failure, modification of therapy, and loss to follow-up as a composite outcome* [18]		Values below are relative risk ratios		Values below are adjusted relative risk ratios	
	**Age (years)**				
	<15	1.21 (0.88,1.65)		1.32 (1.00,1.76)*	
	15-24	Ref		Ref	
	25-34	1.37 (1.13,1.67)*		1.26 (1.07,1.50)*	
	35-44	1.30 (1.04,1.63)*		1.12 (0.92,1.37)	
	45-54	1.87 (1.51,2.31)*		1.59 (1.31,1.93)*	
	55-64	1.91 (1.52,2.39)*		1.67 (1.36,2.05)*	
	≥65	2.74 (2.20,3.40)*		2.05 (1.67,2.53)*	
	**Sex**				
	Female	Ref		Ref	
	Male	1.47 (1.29,1.68)*		1.15 (1.02,1.30)*	
	Others	3.53 (0.71,17.58)		3.52 (1.20,10.31)*	
	**HIV status**				
	Nonreactive	Ref		Ref	
	Reactive	2.59 (2.00,3.38)*		1.73 (1.16,2.57)*	
	Unknown	1.47 (1.30,1.67)*		1.46 (1.24,1.72)*	
	**Site of TB**				
	Pulmonary	1.53 (1.29,1.82)*		1.68 (1.41,2.01)*	
	Extrapulmonary	Ref		Ref	
	Not record	12.62 (10.78,14.79)*		13.11 (10.90,15.78)*	
	**Treatment place**				

	Public	1.13 (0.99,1.30)		2.16 (1.85,2.54)*	
	Private	Ref		Ref	
	**Adherence method**				
	Conventional	2.35 (1.76,3.14)*		2.11 (1.33,3.34)*	
	99DOTS	Ref		Ref	
Ramachandran, 2020 (Maharashtra and Tamil Nadu) *Outcome: Treatment failure as a single outcome* [19]		Values below are incident rate ratios		Values below are adjusted incident rate ratios	
	**Rifampicin**[c]				
	1 unit decrease in drug concentration	1.36 (1.04,1.26)	0.005*	1.16 (1.05,1.28)*	0.003*
	**Isoniazid**[c]				
	1 unit decrease in drug concentration	1.06 (0.00,1.13)	0.076	1.06 (0.99,1.14)	0.1
	**Pyrazinamide**[c]				
	1 unit decrease in drug concentration	1.01 (0.99,1.04)	0.255	1.02 (0.99,1.04)	0.13
Ramachandran, 2020 (Maharashtra and Tamil Nadu) *Outcome: Death as a single outcome* [19]				Values below are adjusted incident rate ratios	
	**Rifampicin**[c]				
	1 unit decrease in drug concentration	1.07 (0.96,1.18)	0.205	1.04 (0.94,1.15)	0.47
	**Isoniazid**[c]				
	1 unit decrease in drug concentration	1.05 (0.97,1.14)	0.212	1.04 (0.72,1.13)	0.28
	**Pyrazinamide**[c]				
	1 unit decrease in drug concentration	1.01 (0.98,1.03)	0.821	1.01 (0.98,1.04)	0.62
Rouf, 2021 (Jammu and Kashmir) *Outcome: Death, treatment failure, loss to follow-up, and not evaluated as a composite outcome* [20]		Values below are odds ratios		Values below are adjusted odds ratios	
	**Age (years)**				
	Reference group is not clearly denoted to understand findings			0.99 (0.95,1.03)	<0.54
	**Type of TB**				
	Reference group is not clearly denoted to understand findings			0.11 (0.02,0.75)*	<0.02*
	**TB related stigma**				
	Absent			Ref	
	Present			0.47 (0.79,2.39)	<0.36
	**Depression at Baseline**				

	Absent	Ref		Ref	
	Present	7.80 (1.72,35.26)*	0.008	6.70 (1.01,35.15)*	<0.05*
	**Depression after 2 Months**				
	Absent	Ref		Ref	
	Present	48.95 (13.71,174.75)*	<0.0001*	58.91 (12.56,166.09)*	<0.001*
	**Depression after 6 Months**				
	Absent	Ref			
	Present	21.23 (3.25,138.52)*	0.001*		
Shameer, 2016 (Kerala) *Outcome: Medication nonadherence (i.e., missing 3 or more consecutive doses)* [21]		Values below are odds ratios		Values below are adjusted odds ratios	
	**Age (years)**				
	>45	Ref			
	<45	1.69 (0.83,3.4)	0.15		
	**Sex**				
	Female	Ref			
	Male	1.53 (0.67,3.70)	0.33		
	**Education**				
	Above primary school	Ref		Ref	
	Up to primary school	3.5 (1.56,8.03)*	0.003*	2.25 (0.83,6.13)	
	**Occupation**				
	Others	Ref			
	Nil/unskilled	0.82 (0.34,1.97)	0.82		
	**SES**				
	Middle	Ref		Ref	
	Lower	2.72 (0.87,8.53)	0.09	1.02 (0.25,4.07)	
	**Type of TB**				
	Extrapulmonary	Ref			
	Pulmonary	1.36 (0.57,3.25)	0.52		
	**Adverse drug reactions**				
	Absent	Ref		Ref	
	Present	2.91 (1.41,6.02)*	0.006*	2.46 (1.07,6.14)*	<0.05*
	**Initial counseling**				
	Not received	Ref			
	Received	1 (0.23,4.18)	1		
	**Selection of DOT center after consulting with patient**				
	Yes	Ref			

	No	2.67 (0.68,10.48)	0.16		
	**Current smoker**				
	No	Ref		Ref	
	Yes	8.34 (2.8,24.81)*	<0.001*	3.84 (0.92,16.06)	
	**Alcohol**				
	Never used	Ref		Ref	
	Non-hazardous use	1.84 (0.75,4.01)	0.21		
	Hazardous use	22.67 (5.76,89.16)*	<0.001*	16.67 (3.22,61.42)*	<0.05*
	**TB stigma perceived by the patient**				
	Lower degree	Ref			
	Higher degree	3.04 (1.40,6.51)*	0.004*		
	**Shared TB status with family**				
	No	Ref		Ref	
	Yes	2.90 (1.06,7.94)*	0.058*	2.22 (0.52,9.50)	
	**Family support**				
	No	Ref			
	Yes	0.64 (0.24,1.73)	0.43		
	**Distance to nearest PHI**				
	<=2 km	Ref		Ref	
	>2 km	2.76 (1.15,6.58)*	0.02*	2.99 (0.99,8.99)	
	**Any conflict with DOT provider**				
	No	Ref			
	Yes	2.2 (0.77,6.30)	0.17		
Shewade, 2019 (multi-state) *Outcome: Death, treatment failure, loss to follow-up, and not evaluated as a composite outcome* [22]				Values below are adjusted relative risk ratios	
	**Cohort including 572 patients**				
	Non-Axshya SAMVAD (passive case finding)			Ref	
	Axshya SAMVAD (active case finding)			0.83 (0.56,1.21)	
	**Cohort including 465 patients**				
	Non-Axshya SAMVAD (passive case finding)			Ref	
Shivam, 2014[a] (West Bengal) *Outcome: Death, treatment failure, and loss to follow-up as a composite outcome* [23]		Values below are odds ratios			
	**Sex**				
	Female	Ref			
	Male	0.81 (0.51,1.30)	0.38		

Singla, 2009 (Delhi)<br>*Outcome: Treatment failure as a single outcome as compared to patients who achieved cure* [24]		Values below are odds ratios			
	**Sex**				
	Female	Ref			
	Male	1.13 (0.39,3.25)	0.83		
	**Age[c]**				
	Per each year increase in age	1.02 (0.98,1.05)	0.41		
	**SES**				
	Upper	Ref			
	Upper-middle	9.00 (0.10,831.85)	0.34		
	Lower-middle	3.86 (0.12,126.74)	0.45		
	Upper-lower	1.49 (0.06,37.50)	0.81		
	Lower	5.00 (0.11,220.64)	0.4		
	**Duration of illness**				
	<2 months	Ref			
	>2 months	14.8 (3.12,70.14)*	<0.001*		
	**Cavity**				
	Absent	Ref		Ref	
	Present	16.02 (5.92,43.97)*	<0.001*	18.35 (2.52,133)*	0.004*
	**Radiological extent of disease**				
	Less advanced	Ref			
	Far advanced	4.63 (2.05,10.46)*	<0.001*		
	**Initial sputum grade**				
	Non-3+	Ref			
	3+	2.89 (1.33,6.23)*	0.01*		
	**Interruptions in treatment (i.e., missing at least one medication dose)**				
	No	Ref		Ref	
	Yes	1.76 (1.09,2.82)*	0.03*	2.018 (1.03,3.94)*	0.04*
	**Culture-positive at 5 months**				
	No	Ref			
	Yes	Infinite (0,infinity)*	<0.001*		
	**Smear-positive at 2 months**				
	No	Ref		Ref	
	Yes	275.00 (33.47,2250)*	<0.001*	219.9 (17.6,2603.2)*	<0.001*
	**BMI[d]**				
	Per each unit increase in BMI	0.91 (0.78,1.05)	0.18		
	**Alcohol use**				

	Non-drinker	Ref			
	Alcohol consumption	1.42 (0.56,3.59)	0.5		
	**Smoking**				
	Non-smoker	Ref			
	Smoker	1.24 (0.57,2.70)	0.69		
Singla, 2013 (Delhi) *Outcome: Smear positive at 2 months as a single outcome* [25]				Values below are adjusted odds ratios	
	**Duration of illness**				
	<=2 months			Ref	
	>2 months			8.29 (2.10,32.70)*	0.003*
	**Cavity**				
	Absent			Ref	
	Present			10.81 (2.42,48.22)*	0.002*
	**Radiological extent of disease**				
	Less advanced			Ref	
	Far advanced			10.80 (3.12,37.39)*	<0.001*
	**Number of interruptions (missed doses) in treatment in IP**				
	No interruptions			Ref	
	1 to 2			4.74 (1.21,18.49)*	0.03*
	3 or more			15.56 (2.15,112.88)*	0.007*
Singla, 2013 (Delhi) *Outcome: Culture positive at 2 months as a single outcome* [25]		Values below are odds ratios			
	**Duration of illness**				
	<=2 months	Ref			
	>2 months	2.38 (1.03,5.50)*	<0.001*		
	**Cavity**				
	Absent	Ref			
	Present	10.81 (2.42,48.22)*	0.002*		
	**Extensive disease**				
	No	Ref			
	Yes	12.19 (3.98,37.37)*	<0.001*		
	**Sputum smear**				
	1 or 2 +	Ref			
	3+	4.85 (1.84,12.75)*	<0.001*		
	**Interruptions**				
	No interruptions	Ref			
	Occasional interrupter	1.39 (0.54,3.56)			
	Frequent interrupter	4.31 (1.43,12.97) *	0.02*		

Singla, 2013[a] (Delhi) *Outcome: Death, treatment failure, and loss to follow-up as a composite outcome* [25]		Values below are odds ratios			
	**Smear status at 2 months**				
	Smear-negative at 2 months	Ref			
	Smear-positive at 2 months	86.26 (5.14,1447.98)*	0.002*		
	**Smear status at 3 months**				
	Smear-negative at 3 months	Ref			
	Smear-positive at 3 months	19.05 (5.60,64.20)*	<0.001*		
	**IP interruption**				
	No IP interruption	Ref			
	Any IP interruption	1.98 (0.84,4.66)	0.12		
	**IP interruption (more categories)**				
	Non-interrupter	Ref			
	Occasional interrupter	1.12 (0.41,3.08)	0.83		
	Frequent interrupter	4.30 (1.40,12.90)*	0.009*		
Sinha, 2023 (Maharashtra, Tamil Nadu, and Puducherry) [26] *Outcome: (1) Death, treatment failure, loss to follow-up, and transferred out as a composite outcome*		Values below are incident rate ratios		Values below are adjusted incident rate ratios	
	**Treatment initiation BMI (kg/m^2)**				
	18.5-22.99	Ref		Ref	
	<16	2.46 (1.79,3.40)*	<0.01*	2.05 (1.42,2.98)*	
	16-16.99	1.70 (1.13,2.51)*	<0.01*	1.50 (0.97,2.31)	
	17-18.49	1.08 (0.69,1.64)		0.99 (0.62,1.54)	
	>23	0.91 (0.56,1.44)		0.76 (0.42,1.29)	
	**Premorbid BMI (kg/m^2)**				
	<18.5-22.99	Ref		Ref	
	<16	1.68 (0.93,2.86)	0.07	2.20 (1.16,3.94)*	
	16-16.99	1.41 (0.77,2.42)	0.07	1.67 (0.89,2.95)	
	17-18.49	0.93 (0.53,1.57)	0.07	1.01 (0.56,1.73)	
	>23	0.96 (0.62,1.45)	0.07	1.16 (0.71,1.85)	
	**Unchanged or decreased BMI**				
	Per unit decrease in BMI	1.81 (1.33,2.48)*	<0.001*	1.81 (1.27,2.61)*	
	**Stunting**				
	Not stunted (HAZ >-2)	Ref		Ref	
	Moderately stunted (-3>HAZ≤-2)	1.13 (0.85,1.48)		1.11 (0.82,1.49)	

	Severely stunted (HAZ ≤3)	1.65 (1.13,2.34)*	<0.01*	1.52 (1.00,2.24)*	
	**Age (years)**				
	18-29	Ref			
	30-39	1.49 (1.02,2.18)*	<0.001*		
	40-49	1.87 (1.33,2.67)*	<0.001*		
	50-59	1.86 (1.27,2.74)*	<0.001*		
	60-69	1.36 (0.72,2.39)			
	70-82	3.73 (1.43,8.01)*	<0.001		
	**Age (years), Baseline BMI**				
	18-29			Ref	
	30-39			1.49 (0.96,2.32)	
	40-49			1.65 (1.08,2.56)*	
	50-59			1.82 (1.14,2.91)*	
	60-69			1.30 (0.65,2.44)	
	70-82			3.56 (1.20,8.55)*	
	**Age (years), Premorbid BMI**				
	18-29			Ref	
	30-39			1.30 (0.76,2.24)	
	40-49			1.35 (0.78,2.36)	
	50-59			1.64 (0.90,2.98)	
	60-69			0.89 (0.32,2.10)	
	70-82			5.44 (1.25,16.53)*	
	**Age (years), Unchanged or Decreased BMI**				
	18-29			Ref	
	30-39			1.22 (0.69,2.15)	
	40-49			1.61 (0.92,2.80)	
	50-59			2.41 (1.36,4.26)*	
	60-69			0.98 (0.33,2.91)	
	70-82			0.00 (0,∞)	
	**Age (years), Stunting BMI**				
	18-29			Ref	
	30-39			1.42 (0.91,2.22)	
	40-49			1.58 (1.03,2.45)*	
	50-59			1.73 (1.08,2.79)*	
	60-69			1.20 (0.60,2.28)	
	70-82			3.13 (1.05,7.50)*	
	**Sex**				
	Female	Ref			
	Male	2.32 (1.70,3.23)*	<0.001		
	**Sex, Baseline BMI**				

	Female			Ref	
	Male			1.58 (1.06,2.40)*	
	**Sex, Premorbid BMI**				
	Female			Ref	
	Male			1.56 (0.92,2.70)	
	**Sex, Unchanged or Decreased BMI**				
	Female			Ref	
	Male			1.56 (0.94,2.61)	
	**Sex, Stunting BMI**				
	Female			Ref	
	Male			1.58 (1.05,2.39)*	
	**Monthly household income**				
	<3000 (<USD 37.5)	Ref			
	3001-500 (USD 37.5-62.5)	0.94 (0.55,1.66)	0.99		
	5001-10000 (USD 62.5-125)	1.00 (0.63,1.69)	0.99		
	>10000 (>USD 125)	0.78 (0.48,1.32)	0.99		
	**Symptom duration (months)**				
	Per month increase in symptom duration	1.01 (0.98,1.03)	0.41		
	**Liquid culture time to positivity (weeks)**				
	Per week increase in culture time	0.96 (0.77,1.18)	0.73		
	**Sputum smear at baseline**				
	Negative	Ref			
	1+	1.51 (1.00,2.35)*	<0.01*		
	2+	1.91 (1.27,2.96)*	<0.01*		
	3+	1.48 (0.91,2.43)			
	Scanty	1.73 (0.77,3.52)			
	**Sputum smear at baseline, Baseline BMI**				
	Negative			Ref	
	1+			1.24 (0.76,2.09)	
	2+			1.42 (0.87,2.40)	
	3+			1.00 (0.56,1.78)	
	Scanty			1.46 (0.61,3.12)	
	**Sputum smear at baseline, Premorbid BMI**				
	Negative			Ref	
	1+			1.30 (0.73,2.39)	
	2+			1.70 (0.95,3.15)	
	3+			1.06 (0.53,2.11)	
	Scanty			1.55 (0.52,3.81)	

	**Sputum smear at baseline, Unchanged or Decreased BMI**				
	Negative			Ref	
	1+			1.19 (0.67,2.09)	
	2+			1.54 (0.87,2.71)	
	3+			1.54 (0.75,3.18)	
	Scanty			1.69 (0.68,4.23)	
	**Sputum smear at baseline, Stunting BMI**				
	Negative			Ref	
	1+			1.29 (0.79,2.17)	
	2+			1.44 (0.88,2.44)	
	3+			1.02 (0.58,1.82)	
	Scanty			1.49 (0.62,3.18)	
	**Cavitation**				
	Per unit increase in cavitation	1.36 (1.01,1.82)*	0.04*		
	**Pretreatment weight loss (kg)**				
	Per kg lost	1.06 (1.03,1.08)*	<0.001*		
	**Smoking**				
	Never	Ref			
	Former	1.85 (1.37,2.48)*	<0.001		
	Current	1.82 (1.35,2.43)*	<0.001*		
	**Smoking, Baseline BMI**				
	Never			Ref	
	Former			1.21 (0.84,1.74)	
	Current			1.16 (0.80,1.65)	
	**Smoking, Premorbid BMI**				
	Never			Ref	
	Former			1.73 (1.07,2.78)*	
	Current			1.51 (0.90,2.51)	
	**Smoking, Unchanged or Decreased BMI**				
	Never			Ref	
	Former			1.52 (0.93,2.50)	
	Current			1.52 (0.94,2.46)	
	**Smoking, Stunting BMI**				
	Never			Ref	
	Former			1.24 (0.86,1.78)	
	Current			1.26 (0.88,1.81)	
	**Alcohol Use Disorder**				
	No	Ref			
	Yes	1.62 (1.22,2.13)*	<0.001		

	**Alcohol Use Disorder, Baseline BMI**				
	No			Ref	
	Yes			1.13 (0.81,1.55)	
	**Alcohol Use Disorder, Premorbid BMI**				
	No			Ref	
	Yes			1.23 (0.90,2.51)	
	**Alcohol Use Disorder, Unchanged or Decreased BMI**				
	No			Ref	
	Yes			1.11 (0.74,1.66)	
	**Alcohol Use Disorder, Stunting BMI**				
	No			Ref	
	Yes			1.18 (0.82,1.69)	
	**Diabetes**				
	No	Ref			
	Yes	0.83 (0.63,1.08)	0.18		
	**Diabetes, Baseline BMI**				
	No			Ref	
	Yes			0.84 (0.60,1.17)	
	**Diabetes, Premorbid BMI**				
	No			Ref	
	Yes			0.76 (0.47,1.21)	
	**Diabetes, Unchanged or Decreased BMI**				
	No			Ref	
	Yes			0.59 (0.40,0.89)*	
	**Diabetes, Stunting BMI**				
	No			Ref	
	Yes			0.87 (0.62,1.21)	
	**Human immunodeficiency virus**				
	No	Ref			
	Yes	1.45 (0.77,2.47)	0.21		
	**Human immunodefiency virus, Baseline BMI**				
	No			Ref	
	Yes			2.02 (0.93,3.97)	
	**Human immunodefiency virus, Premorbid BMI**				
	No			Ref	
	Yes			2.58 (1.15,5.36)*	
	**Human immunodefiency virus, Unchanged or Decreased BMI**				

	No			Ref	
	Yes			1.02 (0.42,2.48)	
	**Human immunodefiency virus, Stunting BMI**				
	No			Ref	
	Yes			1.83 (0.85,3.62)	
	**Antiretroviral therapy status**				
	No	Ref			
	Yes	0.31 (0.07,1.04)	0.08		
Sinha, 2023 (Maharashtra, Tamil Nadu, and Puducherry) *Outcome: Death as a single outcome* [26]				Values below are adjusted incident rate ratios	
	**Treatment initiation BMI (kg/m²)**				
	18.5-22.9			Ref	
	<16			4.17 (1.97,9.53)*	
	16-16.99			1.23 (0.43,3.36)	
	17-18.49			0.53 (0.12,1.78)	
	>23			0.82 (0.18,2.77)	
	**Premorbid BMI (kg/m²)**				
	18.5-22.9			Ref	
	<16			1.18 (0.06,6.41)	
	16-16.99			2.62 (0.81,7.49)	
	17-18.49			2.14 (0.77,5.58)	
	>23			1.70 (0.70,4.12)	
	Unchanged or decreased BMI			5.16 (1.51,17.65)*	
	**Stunted**				
	Not stunted			Ref	
	Moderate stunting			1.08 (0.56,1.98)	
	Severe stunting			1.30 (0.48,2.97)	
Sinha, 2023 (Maharashtra, Tamil Nadu, and Puducherry) *Outcome: Loss to follow-up as a single outcome.* [26]				Values below are adjusted incident rate ratios	
	**Treatment initiation BMI (kg/m²)**				
	18.5-22.9			Ref	
	<16			1.81 (1.46,2.25)*	
	16-16.99			1.37 (1.05,1.78)*	
	17-18.49			1.14 (0.86,1.48)	
	>23			1.02 (0.76,1.35)	
	**Premorbid BMI (kg/m²)**				

	18.5-22.9			Ref	
	<16			0.76 (0.28,1.69)	
	16-16.99			1.19 (0.54,2.35)	
	17-18.49			1.36 (0.82,2.20)	
	>23			1.65 (1.09,2.50)*	
	Unchanged or decreased BMI			6.24 (3.78,10.87)*	
	**Stunted**				
	Not stunted			Ref	
	Moderate stunting			0.56 (0.41,0.77)*	
	Severe stunting			0.82 (0.52,1.25)	
Tiwari, 2012[a] (Delhi) *Outcome: Treatment failure, LTFU, transferred out and death as a composite outcome* [27]		Values below are odds ratios			
	**High/low positive**				
	High positive	Ref			
	Low positive	0.87 (0.49,1.57)	0.65		
	**Conversion at 2 months**				
	Converted at 2 months	Ref			
	Not converted at 2 months	3.86 (1.03,7.32)*	<0.0001*		
	**Conversion at 3 months**				
	Converted at 3 months	Ref			
	Not converted at 3 months	4.25 (1.66,10.90)*	0.003*		
	Not converted at 2 months	3.86 (1.03,7.32)*	<0.0001*		
	**Conversion at 3 months**				
	Converted at 3 months	Ref			
	Not converted at 3 months	4.25 (1.66,10.90)*	0.003*		
Trivedi, 2019[a] (Gujarat) *Outcome: Unable to achieve cure as a single outcome* [28]		Values below are odds ratios			
	**Sex**				
	Female	Ref			
	Male	1.09 (0.26,4.49)	0.91		
	**Age (years)**				
	<=40	Ref			
	>40	1.92 (0.55,6.70)	0.31		
	**Residence**				
	Urban	Ref			
	Rural	2.05 (0.51,8.31)	0.31		
	**Initial sputum colony**				
	1+ or scanty	Ref			
	2+ or 3+	0.58 (0.17,1.97)	0.38		

	**TB/HIV coinfected**				
	No	Ref			
	Yes	1.54 (0.06,39.97)	0.79		
	**Personal habit (i.e., smoking, tobacco, or alcohol use)**				
	No	Ref			
	Yes	3.57 (0.97,13.14)	0.06		
Umayorubhagom, 2023 (Karnataka) *Outcome: Death, treatment failure, modification of therapy, and loss to follow-up as a composite outcome* [29]		Values below are odds ratios		Values below are adjusted odds ratios	
	**Age (years)**				
	<u><</u>20	Ref		Ref	
	21-40	0.94 (0.38,2.33)	0.90	1.08 (0.34,3.49)	0.9
	41-60	1.40 (0.55,3.54)	0.48	1.97 (0.59,6.52)	0.27
	>60	2.33 (0.82,6.58)	0.11	2.38 (0.62,9.1)	0.2
	**Type of house**				
	Kacha	Ref		Ref	
	Pucca	0.34 (0.15,0.76)*	0.008*	0.34 (0.12,0.94)*	0.04*
	Semi-pucca	0.94 (0.45,1.95)	0.86	0.81 (0.34,1.94)	0.64
	**Cough**				
	Absent	Ref		Ref	
	Present	2.84 (1.43,5.65)*	0.003*	4.08 (1.71,9.75)*	0.001*
	**Past history of TB**				
	Absent	Ref		Ref	
	Present	1.87 (1.01,3.47)*	0.05*	0.47 (0.17,1.27)	0.13
	**Family support**				
	Absent	Ref		Ref	
	Present	0.13 (0.07,0.25)*	0.00*	0.17 (0.06,0.43)*	0.0002*
	**Supervision by family**				
	Absent	Ref		Ref	
	Present	0.19 (0.09,0.37)*	0.00*	0.26 (0.1,0.67)*	0.005*
	**Support of supervisor**				
	No	Ref		Ref	
	Yes	0.38 (0.21,0.68)*	0.00*	0.39 (0.19,0.79)*	0.009*
Vashishtha, 2013[a] (Delhi) *Outcome: Death, treatment failure, loss to follow-up, and treatment modified as a composite outcome* [30]		Values below are odds ratios			
	**Outcome at end of initial ATT**				
	HIV-negative	Ref			

	HIV-positive	8.89 (3.04,26.02)*	0.0001*		
Velayutham, 2014 (Tamil Nadu)<br>*Outcome: Death as a single outcome* [31]		Values below are odds ratios			
	**Age**				
	Younger	Ref			
	Elderly	2.60 (1.70,3.90)*	<0.001*		
Velayutham, 2014 (Tamil Nadu)<br>*Outcome: Treatment failure as a single outcome* [31]		Values below are odds ratios			
	**Age**				
	Younger	Ref			
	Elderly	1.97 (0.54,1.66)	0.92		
Velayutham, 2014 (Tamil Nadu)<br>*Outcome: Loss to follow-up as a single outcome* [31]		Values below are odds ratios			
	**Age**				
	Younger	Ref			
	Elderly	1.30 (0.90,1.80)	0.09		
Velayutham, 2014 (Tamil Nadu)<br>*Outcome: Death as a single outcome* [31]		Values below are odds ratios			
	**Age**				
	Younger	Ref			
	Elderly	2.60 (1.6,4.20)*	<0.001*		
Velayutham, 2014 (Tamil Nadu)<br>*Outcome: Treatment failure as a single outcome* [31]		Values below are odds ratios			
	**Age**				
	Younger	Ref			
	Elderly	0.91 (0.49,1.58)	0.74		
		Values below are odds ratios			
Velayutham, 2014 (Tamil Nadu)<br>*Outcome: Loss to follow-up as a single outcome* [31]	**Age**				
	Younger	Ref			
	Elderly	1.40 (1.0,1.97)*	0.03*		
Velayutham, 2018 (6 Indian States)<br>*Outcome: Death, treatment failure, loss to follow-up, treatment modified, and not evaluated as a composite outcome* [32]		Values below are relative risk ratios		Values below are adjusted relative risk ratios	
	**Sex**				

	Female	Ref			
	Male	1.08 (0.98,1.20)	0.12		
	**Age (years)**				
	18-24	Ref			
	25-34	1.03 (0.89,1.19)	0.69		
	35-44	1.04 (0.89,1.21)	0.61		
	45-54	1.03 (0.89,1.20)	0.66		
	55-64	1.06 (0.90,1.24)	0.48		
	>65	1.01 (0.84,1.23)	0.86		
	**Baseline sputum smear grade**				
	Scanty/1+	Ref			
	2+/3+	1.02 (0.93,1.12)	0.66		
	**Baseline sputum culture grade**				
	Cols/1+	Ref			
	2+/3+	0.98 (0.89,1.08)	0.64		
	**Baseline drug susceptibility test**				
	Sensitive	Ref		Ref	
	Resistant to one or more drugs	1.15 (0.98,1.34)	0.08	1.14 (0.96,1.35)	0.14
	**Body mass index**				
	18.5-22.9	Ref			
	>23	0.89 (0.72,1.09)	0.28		
	16-18.4	1.04 (0.92,1.17)	0.54		
	<16	1.06 (0.94,1.19)	0.35		
	**Diabetes**				
	No diabetes	Ref			
	Not on anti-diabetic treatment	0.92 (0.77,1.11)	0.39		
	On anti-diabetic treatment	0.92 (0.79,1.06)	0.25		
	**HIV status**				
	Non-reactive	Ref		Ref	
	Reactive, not on ART	1.51 (0.97,2.34)	0.07	1.44 (0.68,3.03)	0.34
	Reactive, on ART	1.11 (0.84,1.47)	0.45	1.04 (0.73,1.46)	0.84
	**Smoker**				
	Non-smoker	Ref			
	Past smoker	1.08 (0.98,1.18)	0.12		
	Current smoker	1.16 (0.96,1.41)	0.12		
	**Alcohol use**				
	None	Ref		Ref	
	Past	1.09 (1.00,1.20)*	0.04*	1.08 (0.97,1.20)	0.17
	Current	1.02 (0.72,1.46)	0.9	1.00 (0.68,1.47)	1
	**Duration of IP**				

	2 months	Ref			
	3 months	1.09 (0.95,1.24)	0.24		
	**Type of DOT**				
	Health center-based	Ref			
	Community-based	1.03 (0.94,1.34)	0.53		
	**Duration of symptoms to treatment initiation**				
	<3 weeks	Ref			
	3-6 weeks	0.98 (0.87,1.12)	0.79		
	6-9 weeks	1.00 (0.87,1.15)	0.99		
	9-12 weeks	0.98 (0.84,1.14)	0.79		
	>12 weeks	1.03 (0.90,1.18)	0.63		
	**Missed doses in IP of treatment**				
	None	Ref		Ref	
	1 to 6	1.10 (0.96,1.27)	0.18	1.10 (0.95,1.29)	0.2
	7 to 12	1.28 (0.99,1.65)	0.06	1.21 (0.91,1.59)	0.19
	>12	1.31 (0.93,1.84)	0.12	1.29 (0.91,1.81)	0.15
Vijay, 2010 (6 Indian States) *Outcome: Loss to follow-up as a single outcome* [33]		Values below are odds ratios		Values below are adjusted odds ratios	
	**Age (years)**				
	41 and over	Ref			
	<41	1.02 (0.79,1.32)	0.88		
	**Residency time**				
	1 year or more	Ref			
	<1 year	0.94 (0.53,1.66)	0.82		
	**Marital status**				
	Not married	Ref			
	Married	0.87 (0.63,1.19)	0.39		
	**Literacy**				
	Literate	Ref		Ref	
	Not literate	1.47 (1.12,1.92)*	0.004*	1.41 (1.03,1.92)*	0.03*
	**Employment**				
	Not employed	Ref			
	Employed	0.95 (0.73,1.24)	0.73		
	**Number of earners**				
	Patient not sole earner	Ref			
	Patient sole earner	0.97 (0.72,1.33)	0.88		
	**Drug side effects**				
	No side effects to drugs	Ref		Ref	
	Side effects to drugs	3.14 (2.39,4.14)*	<0.001*	2.55 (1.87,3.47)*	<0.001*
	**Associated illness**				
	Did not have associated illness	Ref			

	Had associated illness	0.77 (0.54,1.09)	0.14		
	**Satisfaction**				
	Satisfied with services	Ref		Ref	
	Unsatisfied with services	12.04 (5.92,25.2)*	<0.001*	1.73 (1.14,2.60)*	0.009*
	**Alcoholism**				
	Not alcoholic	Ref		Ref	
	Alcoholic	1.93 (1.48,2.52)*	0.01*	1.72 (1.23,2.44)*	0.002*
	**Smoking**				
	Not a smoker	Ref		Ref	
	Smoker	1.48 (1.14,1.92)*	0.003*	1.12 (0.77,1.64)	0.55
	**Knowledge of TB and treatment**				
	Adequate	Ref		Ref	
	Inadequate or poor	2.22 (1.70,3.00)*	<0.001*	1.88 (1.35,2.63)*	<0.001*
	**Nuclear family**				
	Not having a nuclear family	Ref			
	Having a nuclear family	1.04 (0.79,1.37)	0.77		
	**Family support**				
	Not having family support	Ref			
	Having family support	0.59 (0.23,1.49)	0.22		
	**Other commitments**				
	Did not have other commitments	Ref		Ref	
	Had other commitments	2.44 (1.72,3.45)*	<0.01*	3.22 (1.12,9.09)*	0.03*
	**Distance to DOT center**				
	>2 km	Ref			
	<=2 km	0.91 (0.66,1.26)	0.57		
	**DOT timing**				
	No overlapping work hours and DOT timing	Ref			
	Overlapping work hours and DOT timing	0.93 (0.61,1.42)	0.71		
	**Out station duties**				
	No out station duties during treatment	Ref			
	Out station duties during treatment	2.23 (1.05,4.72)*	0.03*		
	**Patient-provider interaction**				
	Adequate	Ref		Ref	
	Inadequate	2.0 (1.48,2.71)*	<0.001*	1.72 (1.23,2.44)*	<0.001*
	**Health staff support**				
	Adequate	Ref		Ref	
	Inadequate	8.52 (3.40,22.83)*	<0.001*	1.93 (1.41,2.64)*	<0.001*
	**Address verification**				
	Address verification done	Ref		Ref	

	Address verification not done	1.34 (1.01,1.77)*	0.03*	1.37 (1.00,1.88)	0.053
	**DOT location**				
	DOT not at health/sub center	Ref			
	DOT at health/sub center	0.84 (0.59,1.19)	0.32		
	**DOT used**				
	DOT done	Ref		Ref	
	DOT not done	1.35 (1.03,1.80)*	0.03*	1.101 (0.73,1.39)	0.93
	**Instances of missed doses**				
	Had not missed any doses	Ref		Ref	
	Instances of missed doses	3.25 (2.42,3.47)*	<0.001*	2.56 (1.82,3.57)*	<0.001*
Viswanathan, 2014[a] (Tamil Nadu) *Outcome: Death, treatment failure, treatment modified, and loss to follow-up as a composite outcome* [34]		Values below are odds ratios			
	**Diabetic status**				
	Non-diabetic	Ref			
	Diabetic	0.89 (0.25,3.13)	0.86		
Viswanathan, 2014[a] (Tamil Nadu) *Outcome: Treatment failure or treatment modified as a composite outcome* [34]		Values below are odds ratios			
	**Diabetes treatment status**				
	Diabetes not treated	Ref			
	Diabetes treated with oral medications or insulin	36.0 (3.23,401.5)	0.004*		
Viswanathan, 2023 (Tamil Nadu) *Outcome: Death, treatment failure, relapse, and loss to follow-up as a composite outcome* [35]					
	**Normoglycaemia vs. Prediabetes**				
	Normoglycaemia	Ref			
	Prediabetes	1.31 (0.71,2.45)	0.38		
Zaman, 2014[a] (Assam) *Outcome: Death, treatment failure, and loss to follow-up as a composite outcome* [36]		Values below are odds ratios			
	**Time lag between onset of systems and start of treatment**				
	Began treatment within 6 weeks after onset of symptoms	Ref			

	Began treatment between 6 weeks and 6 months after onset of symptoms	16.88 (2.15,132.51)*	0.007*		
Zaman, 2014[a] (Assam) *Outcome: Loss to follow-up as a single outcome* [36]		Values below are odds ratios			
	**Distance from house to DOTS center and association with loss to follow-up among patients with irregular adherence**				
	Within 1 km	Ref			
	>1 km	114.33 (3.8,3395.10)*	0.006*		
Zhou, 2020 (Tamil Nadu) *Outcome: Loss to follow-up as a single outcome* [37]		Values below are odds ratios		Values below are adjusted odds ratios	
	**Age[c]**				
	Increase of 1 year	1.01 (0.99,1.03)	Not reported	1.01 (0.99,1.03)	Not reported
	**Marital status**				
	Married/single/widowed	Ref		Ref	
	Separated/divorced	4.04 (1.51,10.83)*	Not reported	3.80 (1.39,10.38)*	Not reported
	**Religion**				
	Hindu	Ref			
	Christian/Muslim	0.57 (0.23,1.39)	Not reported		
	**Monthly income (Indian rupees)**				
	5500 or less	Ref			
	More than 5500	1.53 (0.94,2.50)	Not reported		
	**Weight**				
	Normal weight or overweight	Ref			
	Underweight or severely underweight	1.77 (1.03,3.05)*	Not reported		
	**Diabetic status**				
	Non-diabetic	Ref		Ref	
	Diabetic	0.50 (0.29,0.87)*	Not reported	0.52 (0.29,0.92)*	Not reported
	**Smoking tobacco use**				
	Nonsmoker	Ref			
	Current smoker or former smoker	1.92 (1.12,3.30)*	Not reported		
	**At risk alcohol use**				
	Not at risk	Ref		Ref	
	At risk	2.20 (1.31,3.69)*	Not reported	1.92 (1.12,3.27)*	Not reported
	**Knowledge TB transmitted by cough**				
	No	Ref			

	Yes	0.61 (0.36,1.06)	Not reported		
Studies in people with drug-susceptible TB who have a prior TB treatment history					
Ahmed, 2022 (Karnataka) *Outcome: Loss to follow-up as a single outcome* [38]				Values below are adjusted odds ratios	
	**Continuation of treatment after symptom relief**				
	Agree			Ref	
	Disagree			2.74 (0.17,45.30)	0.48
	**TB can be cured if treatment is fully completed**				
	Agree			Ref	
	Disagree			4.20 (0.41,42.98)	0.23
	**Incomplete treatment leads to TB spread among family**				
	Agree			Ref	
	Disagree			86.92 (7.24,1041.57)*	<0.001*
	**Coughing in open leads to spread of TB**				
	Yes			Ref	
	No			8.68 (0.85,88.93)	0.07
	**Sharing food will lead to TB**				
	Disagree			Ref	
	Agree			11.19 (1.32,94.77)*	0.02*
	**Overcrowding spreads TB**				
	Yes			Ref	
	No			4.67 (0.31,70.38)	0.27
	**Symptoms of TB**				
	Poor/average			Ref	
	Good			121.06 (3.96,3704.21)*	0.006*
	**Treatment duration**				
	Correct			Ref	
	Incorrect			66.91 (4.86,921.57)*	0.002*
	**Discrimination by family/friends/relatives**				
	Absent			Ref	
	Present			2.08 (0.12,37.42)	0.62
	**Lack of motivation to complete treatment**				
	Absent			Ref	
	Present			276.64 (14.29,5355.96)*	<0.001*

	**Fear of side effects**				
	Absent			Ref	
	Present			0.655 (0.07,5.88)	0.71
	**Number of tablets to be taken**				
	Little			Ref	
	Many			11.72 (0.96,142.80)	0.05
	**Smoking**				
	Never use			Ref	
	Current use			4.25 (2.13,8.51)*	<0.001*
	**Tobacco (smokeless)**				
	Never use			Ref	
	Current use			4.54 (2.00,10.35)*	<0.001*
	**Alcohol**				
	Never use			Ref	
	Current use			3.26 (1.62,6.58)*	<0.001*
	**Attitude/Behavior of treatment provider**				
	Satisfactory			Ref	
	Unsatisfactory			4.46 (2.08,9.59)*	<0.001*
	**Collection medication by family**				
	Not allowed			Ref	
	Allowed			2.11 (1.07,4.14)*	0.03*
	**Distance from treatment centre**				
	<5 km			Ref	
	>5 km			3.38 (1.32,8.65)*	0.01*
	**Disease explained by provider**				
	Yes			Ref	
	No			2.99 (1.41,6.34)*	0.004*
	**Swallow medicines in front of provider**				
	Yes			Ref	
	No			2.34 (0.65,8.46)	0.20
Babiarz, 2014 (Bihar) *Outcome: Loss to follow-up as a single outcome (i.e., treatment discontinuation <25 weeks after initiation)* [3]				Values below are adjusted odds ratios	
	**Sex**				
	Female			Ref	
	Male			1.02 (0.39,2.69)	
	**Age**[c]				
	Per each year increase in age			0.84 (0.70,1.01)	

	**Religion**				
	Non-Hindu			Ref	
	Hindu			0.25 (0.08,0.78)*	
	**Caste/tribe**				
	Other			Ref	
	Scheduled caste, scheduled tribe, or other backward class			0.36 (0.08,1.71)	
	**Number of children in household**[c]				
	Per each increase in number of children			0.80 (0.53,1.20)	
	**Education**				
	Per each year increase in education			0.75 (0.58,0.98)*	
	**Poor**				
	No			Ref	
	Yes			0.76 (0.14,4.11)	
	**Middle income**				
	No			Ref	
	Yes			0.99 (0.30,3.20)	
	**Household size**[c]				
	Per each person increase in household			1.35 (0.97,1.88)	
	**Completed previous TB treatment**				
	No			Ref	
	Yes			0.15 (0.03,0.73)*	
	**Total weeks from symptom onset to treatment initiation**[c]				
	Per each week increase in symptoms			1.04 (0.90,1.20)	
	**Number of symptoms at treatment initiation**				
	>=5			Ref	
	2 or fewer			4.20 (1.26,13.92)*	
	3 to 4			2.55 (0.66,9.95)	
	**Travel cost as a barrier**				
	No			Ref	
	Yes			1.43 (0.13,16.08)	
	**Number of providers visited**[c]				
	Per each provider increased			3.08 (0.46,20.45)	
	**Treatment or medication fees**				
	No			Ref	
	Yes			1.13 (0.08,16.48)	

Bhagat, 2010[a] (Maharashtra) *Outcome: Loss to follow-up as a single outcome* [39]		Values below are odds ratios		Values below are adjusted odds ratios	
	**Age (years)**				
	<24	Ref			
	25-34	1.74 (0.4,7.62)	0.46		
	35-44	2.69 (0.58,12.6)	0.21		
	45-54	1.39 (0.28,6.8)	0.68		
	**Sex**				
	Female	Ref		Ref	
	Male	3.34 (0.71,15.67)	0.13	3.96 (0.61,25.77)	0.15
	**Religion**				
	Hindu	Ref			
	Muslim	2.25 (0.79,6.43)	0.13		
	Others	1.23 (0.33,4.52)	0.75		
	**Marital status**				
	Unmarried	Ref			
	Married	2.02 (0.61,6.62)	0.25		
	**Literacy**				
	Literate	Ref		Ref	
	Illiterate	5.28 (1.92,14.48)*	0.001*	3.51 (1.10,11.24)*	0.03*
	**Employment**				
	Unemployed	Ref		Ref	
	Employed	3.86 (1.47,10.15)*	0.006*	3.52 (1.09,11.33)*	0.04*
	**Overcrowding**				
	Absent	Ref		Ref	
	Present	1.79 (0.66,4.85)	0.25	1.26 (0.37,4.25)	0.71
	**History of TB**				
	Absent	Ref		Ref	
	Present	5.07 (0.79,32.39)	0.09	1.15 (0.11,11.98)	0.91
	**Alcohol use**				
	Not alcoholic	Ref		Ref	
	Alcoholic	4.66 (1.71,12.73)*	0.003*	3.41 (1.04,11.22)*	0.04*
	**Type of family**				
	Joint family	Ref		Ref	
	Nuclear family	0.68 (0.24,1.94)	0.47	0.67 (0.19,2.37)	0.53
Chakrabarti, 2012[a] (West Bengal) *Outcome: Death, treatment failure, loss to follow-up, and transferred out as a composite outcome* [8]		Values below are odds ratios			
	**Population**				
	Non-tribal	Ref			

	Tribal	1.23 (0.41,3.72)	0.71		
Chandrasekaran[a], 2006 (Tamil Nadu) *Outcome: Death, treatment failure, loss to follow-up, and others (e.g., transferred out) as a composite outcome* [40]		Values below are odds ratios			
	**Category of previously treated patient based on prior treatment outcome**				
	Relapse (completion of prior treatment)	Ref			
	Loss to follow-up during previous treatment	2.65 (1.55,4.52)*	0.0004*		
	Treatment failure	1.73 (1.02,2.93)*	0.04*		
Deepa, 2013 (Andhra Pradesh) *Outcome: Death, treatment failure, loss to follow-up, and transferred out as a composite outcome* [41]		Values below are relative risk ratios		Values below are adjusted relative risk ratios	
	**Sex**				
	Female	Ref		Ref	
	Male	1.36 (1.1,1.69)*	Not reported	1.36 (1.09,1.68)*	Not reported
	**Age (years)**				
	<40	Ref			
	>=40	1.2 (0.94,1.33)	Not reported		
	**INH-resistance**				
	No	Ref		Ref	
	Yes	1.44 (1.18,1.78)*	Not reported	1.46 (1.19,1.78)*	Not reported
	**Type**				
	Relapse	Ref		Ref	
	TAD	1.2 (0.99,1.44)	Not reported	1.18 (0.98,1.42)	Not reported
	Failure	1.71 (1.35,2.16)*	Not reported	1.62 (1.28,2.04)*	Not reported
	**HIV status**				
	Negative	Ref		Ref	
	Positive	1.34 (1.0,1.79)	Not reported	1.34 (1.0,1.77)	Not reported
	Unknown	1.66 (1.11,2.48)*	Not reported	1.68 (1.13,2.51)*	Not reported
	**ART**				
	Received	Ref			
	Not received	1.93 (1.14,3.29)*	Not reported		
Joseph, 2011[a] (Karnataka) *Outcome: Treatment failure and loss to follow-up as a composite outcome* [13]		Values below are odds ratios			
	**Sex**				

	Female	Ref			
	Male	0.71 (0.18,2.76)	0.62		
	**Age (years)**				
	<=30	Ref			
	30-60	1.05 (0.37,2.97)	0.93		
	>60	0.91 (0.20,4.10)	0.9		
	**Residence**				
	Urban	Ref			
	Rural	1.48 (0.58,3.80)	0.41		
Jha, 2010 (Nationally Representative Sample) *Outcome: Loss to follow-up as a single outcome as compared to treatment success or treatment failure* [42]		Values below are odds ratios		Values below are adjusted odds ratios	
	**Sex**				
	Female	Ref		Ref	
	Male	1.56 (1.28,1.89)*	<0.01*	1.42 (1.16,1.73)*	<0.01*
	**Age (years)**				
	25-34	Ref		0.87 (0.68,1.09)	0.23
	<15	0.44 (0.17,1.07)	0.05	0.49 (0.19,1.22)	0.12
	15-24	0.85 (0.63,1.14)	0.26	0.79 (0.59,1.04)	0.1
	35-44	1.19 (0.94,1.51)	0.13	Ref	
	45-54	0.98 (0.76,1.26)	0.84	0.81 (0.63,1.03)	0.09
	55-64	1.39 (1.03,1.89)	0.26	1.12 (0.84,1.51)	0.43
	>=65	0.72 (0.47,1.10)	0.11	0.60 (0.40,0.91)*	0.02*
	**Classification**				
	Smear-positive	Ref			
	Smear-negative	0.92 (0.74,1.14)	0.42		
	Smear-unknown	0.65 (0.26,1.6)	0.31		
	Extrapulmonary	0.63 (0.37,1.08)	0.07		
	**Outcome of previous TB treatment**				
	Relapse (previous treatment completed)	Ref		Ref	
	Previous treatment failure	1.16 (0.85,1.60)	0.33	1.14 (0.84,1.56)	0.39
	Loss to follow-up during previous treatment	1.41 (1.15,1.72)*	<0.01*	1.31 (1.07,1.61)*	<0.01*
	"Other" previously treated patient**	1.04 (0.83,1.30)	0.74	0.98 (0.77,1.24)	0.86
	**Adverse Reaction**				
	Not documented defaulter	Ref			
	Documented defaulter	27.6 (10.8,76.7)*	<0.01*		
	**Source of previous treatment**				
	RNTCP (i.e., public sector)	Ref		Ref	

	Non-RNTCP (i.e., private sector)	1.31 (1.07,1.6)*	<0.01*	1.28 (1.04,1.57)*	<0.01*
	Data missing	1.18 (0.96,1.44)	0.1	1.14 (0.92,1.40)	0.21
	**Nature of DOT provider**				
	Public health facility	Ref			
	Community provider	0.92 (0.72,1.19)	0.52		
	Medical college	0.44 (0.28,0.71)*	<0.01*		
	Private provider	0.66 (0.49,0.88)*	<0.01*		
	NGO	0.66 (0.41,1.05)	0.06		
	Data missing	1.26 (0.24,7.11)	0.75		
	**Nature of DOT provider**				
	Other facility (e.g., community providers, medical providers, private practitioners, or non-governmental organizations)			Ref	
	Public health facility			1.33 (1.11,1.60)*	<0.01*
	**Missed doses during IP**				
	None	Ref			
	1 or more	1.66 (1.39,1.99)*	<0.01*		
	2 or more	1.69 (1.40,2.04)*	<0.01*		
	3 or more	1.68 (1.37,2.05)*	<0.01*		
	4 or more	1.86 (1.50,2.30)*	<0.01*		
	5 or more	1.93 (1.54,2.43)*	<0.01*		
	6 or more	1.9 (1.49,2.42)*	<0.01*		
	10 or more	1.93 (1.41,2.63)*	<0.01*		
Mukherjee, 2009[a] (West Bengal) *Outcome: Death, treatment failure, loss to follow-up, and transferred as a composite outcome* [16]		Values below are odds ratios			
	**Outcome of previous TB treatment**				
	Relapse (previous treatment completed)	Ref			
	Previous treatment failure	2.77 (1.42,5.38)*	0.003*		
	Loss to follow-up during previous treatment	2.55 (1.17,5.54)*	0.02*		
	**Sputum grade (initial)**				
	Low grade (1+)	Ref			
	High grade (2+, 3+)	1.73 (0.94,3.2)	0.08		
Nagaraja, 2011[a] (Andhra Pradesh) *Outcome: Death, treatment failure, loss to follow-up, and transferred as a composite outcome* [43]		Values below are odds ratios			

	**Treatment category during prior treatment episode**				
	Category I	Ref			
	Category II	2.31 (1.22,4.38)*	0.009*		
	Category III	0.58 (0.09,3.74)	0.57		
	**Sensitivity pattern**				
	Pan sensitive	Ref			
	Any resistance	3.22 (1.39,7.5)*	0.007*		
	Resistance to 'S' only	0.29 (0.07,1.25)	0.09		
	Resistance to 'H' only	3.29 (0.89,12.18)	0.08		
	Resistance to 'H' and 'S'	16.94 (0.97,294.51)	0.05		
	Resistance to 'H' and 'E'	5.26 (0.27,101.14)	0.27		
	Resistance to 'S' and 'E'	1.74 (0.17,17.51)	0.64		
	Resistance to 'S,' 'H,' and 'E'	1.74 (0.33,9.19)	0.51		
	Negative culture	0.64 (0.32,1.29)	0.21		
	Non-tuberculous mycobacteria	2.92 (0.14,162.93)	0.49		
Pardeshi, 2010[a] (Maharashtra) *Outcome: Loss to follow-up as a single outcome* [44]		Values below are odds ratios			
	**Outcome of previous TB treatment**				
	Relapse (previous treatment completed)	Ref			
	Previous treatment failure	0.95 (0.14,6.46)	0.96		
	Loss to follow-up during previous treatment	2.91 (0.76,11.10)	0.12		
Prajapati, 2023 (Gujarat) *Outcome: Death, treatment failure, modification of therapy, and loss to follow-up as a composite outcome* [18]		Values below are relative risk ratios			
	**Age (years)**				
	<15	Ref			
	15-24	1.32 (0.20,8.61)			
	25-34	1.68 (0.26,10.74)			
	35-44	1.95 (0.30,12.53)			
	45-54	1.99 (0.31,12.71)			
	55-64	1.46 (0.22,9.77)			
	≥65	2.46 (0.37,16.34)			
	**Sex**				
	Female	Ref			
	Male	1.27 (0.92,1.77)			
	**HIV status**				
	Nonreactive	Ref			

	Reactive	1.73 (0.85,3.53)			
	Unknown	0.89 (0.66,1.22)			
	**Site of TB**				
	Pulmonary	2.80 (1.56,5.01)*			
	Extrapulmonary	Ref			
	**Treatment place**				
	Public	1.31 (0.58,2.96)			
	Private	Ref			
	**Adherence method**				
	Conventional	0.95 (0.43,2.08)			
	99DOTS	Ref			
Sarpal, 2014[a] (Punjab) *Outcome: Death, treatment failure, and loss to follow-up as a composite outcome* [45]		Values below are odds ratios			
	**Outcome of previous TB treatment**				
	Relapse (previous treatment completed)	Ref			
	Previous treatment failure	3.58 (1.78,7.21)*	0.0004*		
	Loss to follow-up during previous treatment	2.09 (1.18,3.69)*	0.01*		
	"Other" previously treated patient**	0.18 (0.08,0.41)*	<0.0001*		
Sarpal 2014[a] (Punjab) *Outcome: Loss to follow-up as a single outcome* [45]		Values below are odds ratios		Values below are adjusted odds ratios	
	**Age (years)**				
	<=35	Ref		Ref	
	>35	1.41 (0.65,3.04)	0.44	0.74 (0.32,1.73)	0.49
	**Sex**				
	Female	Ref		Ref	
	Male	19.17 (2.71,380.6)*	<0.001*	6.84 (0.73,64.41)	0.09
	**Religion**				
	Other than Hindu	Ref		Ref	
	Hindu	1.73 (0.49,7.32)	0.52	1.63 (0.46,5.72)	0.45
	**Marital status**				
	Single (unmarried)	0.57 (0.21,1.51)	0.31	1.01 (0.34,2.98)	0.99
	Not single	Ref		Ref	
	**Type of family**				
	Nuclear	0.91 (0.36,0.38)	0.99	0.85 (0.33,2.18)	0.74
	Other than nuclear	Ref		Ref	
	**Socioeconomic status**				
	Low	Ref			
	Others	1.63 (0.75,3.53)	0.24		
	**Place of residence**				

	Slum Urban	Ref		Ref	
	Rural	1.13 (0.47,2.79)	0.93	0.87 (0.35,2.18)	0.76
	**Education**				
	Illiterate	Ref		0.941 (0.32,2.76)	0.91
	Literate	1.21 (0.39,3.46)	0.7	Ref	
	**Substance use**				
	No substance use disorder	Not reported		Ref	
	Substance use disorder ("addicted")	Not reported	Not reported	4.45 (1.28,15.48)*	0.019*
Shivam, 2014[a] (West Bengal) *Outcome: Death, treatment failure, and loss to follow-up as a composite outcome* [23]		Values below are odds ratios			
	**Sex**				
	Female	Ref			
	Male	1.55 (0.58,4.16)	0.39		
Singla, 2009[a] (Delhi) *Outcome: Death, treatment failure, loss to follow-up, and transferred out as a composite outcome* [24]		Values below are odds ratios			
	**Culture status among sputum smear positive patients**				
	Smear-positive, culture negative	Ref			
	Smear-positive, culture positive	18.75 (3.25,108.23)*	0.001*		
	**Resistance**				
	Drug resistance but not multidrug resistance	Ref			
	Multidrug resistance	0.25 (0.02,2.58)	0.24		
Sisodia, 2006[a] (Rajasthan) *Outcome: Death, treatment failure, loss to follow-up, and transferred out as a composite outcome* [46]					
	**Outcome of previous TB treatment**	Values below are odds ratios			
	Relapse (previous treatment completed)	Ref			
	Loss to follow-up during previous treatment	1.77 (1.28,2.45)*	0.0006*		
	Previous treatment failure	1.75 (0.80,3.86)	0.16		
	**Source of previous TB treatment**				
	Government (TB program or other government sector facility)	Ref			
	Private sector	16.91 (7.02,40.72)*	<0.0001*		
Srinath, 2010[a] (Andhra Pradesh) *Population: all previously*		Values below are odds ratios			

treated TB patients; Outcome: Death, treatment failure, loss to follow-up, transferred out, and not recorded as a composite outcome [47]					
	**Type of retreatment**				
	Others treated with Cat II	Ref			
	Smear-positive relapse	1.43 (1.19,1.71)*	0.0001*		
	Smear-positive failures	3.39 (2.68,4.29)*	<0.0001*		
	Smear-positive treatment after default	1.87 (1.50,2.20)*	<0.0001*		
Srinath, 2010[a] (Andhra Pradesh) *Population: previously treated "other" TB patients; Outcome: Death, treatment failure, loss to follow-up, transferred out, and not recorded as a composite outcome* [47]		Values below are odds ratios			
	**Sex**				
	Female	Ref			
	Male	1.43 (1.02,2.02)*	0.04*		
	**Age (years)**				
	<15	Ref			
	15-64	1.24 (0.27,5.70)	0.78		
	**Site of TB**				
	Pulmonary	Ref			
	Extra-pulmonary	0.56 (0.34,0.90)*	0.02*		
	**HIV status**				
	HIV-negative	Ref			
	HIV-positive	3.10 (1.39,6.90)*	0.006*		
	HIV-unknown	1.74 (0.97,3.12)	0.06		
Velavan, 2018[a] (Puducherry) *Outcome: Death, treatment failure, and loss to follow-up as a composite outcome* [48]		Values below are relative risk ratios		Values below are adjusted relative risk ratios	
	**Sex**				
	Female	Ref		Ref	
	Male	1.7 (0.9,3.1)		1.5 (0.8,2.5)	0.17
	**Age (years)**				
	<15	Ref			
	15-29	1.8 (0.4,6.6)	Not reported		
	30-44	2.3 (0.6,8.5)	Not reported		
	45-59	1.5 (0.4,6.5)	Not reported		

	>60	1.6 (0.2,14.2)	Not reported		
	**Place of residence**				
	Urban	Ref			
	Rural	0.8 (0.6,1.3)	Not reported		
	Peri-urban	1.2 (0.7,2.2)	Not reported		
	**Outcome of previous TB treatment**				
	Relapse (previous treatment completed)	Ref		Ref	
	Previous treatment failure	1.7 (1.1,2.4)*	Not reported	1.7 (1.04,2.8)*	0.03*
	Loss to follow-up during previous treatment	1.7 (1.1,2.5)*	Not reported	1.6 (1.1,2.4)*	0.01*
	"Other" previously treated patient**	0.4 (0.1,0.9)*	Not reported	0.7 (0.3,1.8)	0.4
	**Site of TB**				
	Pulmonary	Ref		Ref	
	Extrapulmonary	0.1 (0.02,0.9)*	Not reported	0.3 (0.03,2.3)	0.22
	**HIV status**				
	Negative	Ref			
	Positive	0.9 (0.2,5.3)	Not reported		
	**Pre-treatment weight**				
	>=40 kg	Ref		Ref	
	<40 kg	1.9 (1.3,2.6)*	Not reported	1.8 (1.3,2.5)*	0.001*
Velayutham, 2014 (Tamil Nadu) *Patient population: all previously treated TB patients; Outcome: Loss to follow-up as a single outcome* [31]		Values below are odds ratios			
	**Age**				
	Younger	Ref			
	Elderly	0.90 (0.60,1.40)	0.61		
Velayutham, 2014 (Tamil Nadu) *Patient population: all previously treated TB patients; Outcome: Death as a single outcome* [31]		Values below are odds ratios			
	**Age**				
	Younger	Ref			
	Elderly	1.50 (0.70,3.00)	0.25		
Velayutham, 2014 (Tamil Nadu) *Patient population: all previously treated TB patients; Outcome:*		Values below are odds ratios			

Treatment failure as a single outcome [31]					
	**Age**				
	Younger	Ref			
	Elderly	0.41 (0.14,1.18)	0.1		
Velayutham, 2014 (Tamil Nadu)<br>*Patient population: previously treated smear-positive pulmonary TB patients; Outcome: Loss to follow-up as a single outcome* [31]		Values below are odds ratios			
	**Age**				
	Younger	Ref			
	Elderly	0.90 (0.50,1.40)	0.5		
Velayutham, 2014 (Tamil Nadu)<br>*Patient population: previously treated smear-positive pulmonary TB patients; Outcome: Treatment failure as a single outcome* [31]		Values below are odds ratios			
	**Age**				
	Younger	Ref			
	Elderly	0.42 (0.11,1.18)	0.09		
Velayutham, 2014 (Tamil Nadu)<br>*Patient population: previously treated smear-positive pulmonary TB patients; Outcome: Death as a single outcome* [31]		Values below are odds ratios			
	**Age**				
	Younger	Ref			
	Elderly	1.60 (0.60,3.30)	0.28		
Studies in people with drug-resistant TB					
Bhatt, 2018 (Delhi)<br>*Outcome: Death as a single outcome* [49]		Values below are hazard ratios		Values below are adjusted hazard ratios	
	**Age**[c]				
	Per each year increase in age	Not reported		1.007 (0.98,1.04)	0.63
	**Sex**				
	Female	Not reported		Ref	
	Male	Not reported		4.07 (1.47,11.24)*	0.007*
	**Support duration**[c]				
	Per each month increase in duration of a support package including counseling, nutritional	Not reported		0.88 (0.81,0.95)*	0.0009*

	supplementation, and cash transfer				
	**Initial BMI[c,***]**				
	Per each unit decrease in BMI	Not reported		1.22 (1.07,1.39)*	0.005*
	**Family size[c]**				
	Per each increase in member of family	Not reported		0.91 (0.72,1.14)	0.4
Bhatt, 2018 (Delhi) *Outcome: Treatment failure as a single outcome* [49]				Values below are adjusted odds ratios	
	**Age[c]**				
	Per each year increase in age	Not reported		1.02 (0.96,1.10)	0.56
	**Sex**				
	Female	Not reported		Ref	
	Male	Not reported		2.56 (0.64,12.24)	0.2
	**Support duration[c]**				
	Per each month increase in duration of a support package including counseling, nutritional supplementation, and cash transfer	Not reported		0.93 (0.85,0.998)	0.051
	**Initial BMI[c,***]**				
	Per each unit decrease in BMI	Not reported		1.06 (0.88,1.29)	0.55
	**Family size[c]**				
	Per each increase in member of family	Not reported		0.85 (0.58,1.20)	0.35
Bhatt, 2018 (Delhi) *Outcome: Loss to follow-up as a single outcome* [49]				Values below are adjusted odds ratios	
	**Age[c]**				
	Per each year increase in age	Not reported		1.07 (1.02,1.13)*	0.01*
	**Sex**				
	Female	Not reported		Ref	
	Male	Not reported		6.48 (1.40,40.27)*	0.03*
	**Support duration[c]**				
	Per each month increase in duration of a support package including counseling, nutritional supplementation, and cash transfer	Not reported		0.75 (0.60,0.87)*	0.002*
	**Initial BMI[c,***]**				
	Per each unit decrease in BMI	Not reported		1.26 (1.004,1.60)*	0.04*
	**Family size[c]**				
	Per each increase in member of family	Not reported		1.19 (0.87,1.68)	0.29
Dash, 2022 (Odisha)		Values below are odds ratios			

Outcome: Death, loss to follow-up, failure to complete treatment regimen, and transferred out as a composite outcome [51]					
	**Age (years)**				
	15-30	Ref			
	31-45	2.19 (0.32,15.04)	0.43		
	46-60	8.17 (1.03,64.94)*	0.0471*		
	**Sex**				
	Female	Ref			
	Male	1.05 (0.19,5.69)	0.96		
	**BMI**				
	Normal weight or more (BMI >18.5)	Ref			
	Underweight (BMI <18.5)	1.59 (0.36,7.11)	0.54		
	**Sputum Positive**				
	No	Ref			
	Yes	0.76 (0.04,13.41)	0.85		
	**Retreatment Case**				
	No	Ref			
	Yes	0.27 (0.05,1.36)	0.11		
	**Drug Resistance**				
	Only R resistance	Ref			
	R+H resistance	0.27 (0.03,2.73)	0.27		
	**Anemia**				
	Mild anemia	Ref			
	Moderate anemia	0.37 (0.07,1.89)	0.23		
	Severe anemia	2.40 (0.18,32.88)	0.51		
	**Leucopenia and Leukocytosis**				
	No	Ref			
	Yes	3.50 (0.76,16.12)	0.11		
	**Co morbidity**				
	No	Ref			
	Yes	3.20 (0.49,20.81)	0.22		
	**Thyroid dysfunction (n=28)**				
	Euthyroid	Ref			
	Sickeuthyroid	0.70 (0.13,3.68)	0.67		
	Subclinical hypothyroid/Subclinical hyperthyroid	1.40 (0.14,13.57)	0.77		
Dela, 2017[a] (Gujarat) *Outcome: Death, progression to extensively drug-resistant TB, loss to*		Values below are odds ratios			

follow-up, and transfer out as a composite outcome					
	**Adverse drug reaction**				
	Yes	Ref			
	No	3.43 (1.61,7.27)*	0.0013*		
Dela, 2017[a] (Gujarat) *Outcome: Medication non-adherence as a single outcome*		Values below are odds ratios			
	**Adverse drug reaction**				
	Yes	Ref			
	No	5.35 (2.43,11.78)*	<0.0001*		
Dole, 2017[b] (Maharashtra) *Outcome: Death, treatment failure, and loss to follow-up as a composite outcome* [50]		Values below are odds ratios			
	**Age (years)**				
	>50	Ref			
	18-50	0.70 (0.28,1.75)	0.59		
	**Sex**				
	Female	Ref			
	Male	1.23 (0.61,2.48)	0.68		
	**Residence**				
	Urban	Ref			
	Rural	2.44 (1.22,4.90)*	0.02*		
	**X-ray findings**				
	Moderate	Ref			
	Advanced	3.31 (1.51,7.24)*	0.003*		
	**Weight (kg)**				
	45-70	Ref			
	26-45	1.38 (0.68,2.78)	0.48		
	**Test used for diagnosis**				
	GeneXpert	Ref			
	Line probe assay	2.51 (1.14,5.52)*	0.03*		
Duraisamy, 2014 (Kerala) *Outcome: Death, treatment failure, progression to extensively drug-resistant TB, loss to follow-up, treatment interruption due to adverse drug reaction, and transfer out as a composite outcome* [53]		Values below are hazard ratios		Values below are adjusted hazard ratios	
	**Sex**				
	Female	Ref			
	Male	0.9 (0.6,1.4)			
	**Age (years)**				

	25-44	Ref			
	15-24	1.0 (0.5,1.9)			
	>44	1 (0.7,1.5)			
	**Living below poverty line**				
	No	Ref			
	Yes	1.1 (0.7,1.7)			
	**Cavitary chest radiograph**				
	No	Ref			
	Yes	0.7 (0.3,1.9)			
	**Number of previous TB episodes**				
	>=3	Ref			
	1 to 2	0.9 (0.6,1.5)			
	**At least 1 adverse drug event**				
	No	Ref			
	Yes	1 (0.7,1.5)			
	**Hospitalization during treatment**				
	No	Ref		Ref	
	Yes	1.7 (1.1,2.7)*		1.5 (1.0,2.5)*	
	**HIV seropositive**				
	No	Ref			
	Yes	20.3 (0 to infinity)			
	**Diabetes**				
	No	Ref			
	Yes	0.9 (0.6,1.5)			
	**Alcohol before treatment**				
	No	Ref			
	Yes	0.9 (0.6,1.5)			
	**Alcohol during treatment**				
	No	Ref		Ref	
	Yes	4.9 (1.2,20.3)*		4.3 (1.1,17.6)*	
	**Tobacco before treatment**				
	No	Ref		Ref	
	Yes	0.9 (0.6,1.4)		0.6 (0.2,1.7)	
	**Tobacco during treatment**				
	No	Ref		Ref	
	Yes	1.7 (0.9,3.5)		1.2 (0.3,5.0)	
Duraisamy, 2014 (Kerala) *Outcome: Medication non-adherence (mean number of doses missed in the*		Values below represent the mean number of missed doses (standard deviation)		Value below represents the mean difference (95% confidence	

intensive phase of therapy) as a single outcome [53]				interval) in missed doses	
	**Alcohol consumption during treatment**				
	No	0.6 (3.3)			
	Yes	7.2 (14.2)		6.6 (3.8,9.3)	<0.0001
Duraisamy, 2014 (Kerala) *Outcome: Medication non-adherence (mean number of doses missed in the continuation phase of therapy) as a single outcome* [53]		Values below represent the mean number of missed doses (standard deviation)		Value below represents the mean difference (95% confidence interval) in missed doses	
	**Alcohol consumption during treatment**				
	No	4.4 (14.5)			
	Yes	12.5 (28.5)		8.1 (,0.3,16.5)	0.06
Giri, 2022 (Bihar) *Outcome: Death, treatment failure, loss to follow-up, and transfer out as a composite outcome* [54]		Values below are odds ratios			
	**Sex**				
	Female	Ref			
	Male	2.46 (1.45,4.19)*	0.0009*		
	**Education**				
	Literate and above	Ref			
	Illiterate	2.84 (1.56,5.14)*	0.0006*		
	**Income**				
	Above Poverty Line	Ref			
	Below Poverty Line	3.89 (1.95,7.76)*	0.0001*		
	**Diabetes**				
	No	Ref			
	Yes	2.38 (1.06,5.34)*	0.03*		
	**BMI**				
	Normal	Ref			
	Low (<18.5)	2.63 (1.45,4.79)*	0.002*		
	**Alcohol Use**				
	No	Ref			
	Yes	4.38 (1.40,13.69)*	0.01*		
	**Tobacco Use**				
	No	Ref			
	Yes	2.15 (1.15,4.04)*	0.02*		
Isaakidis, 2012 (Maharashtra) *Outcome: Treatment failure, LTFU and death as a composite outcome in MDR-TB/HIV coi-infected patients* [55]		Values below are odds ratios			

	**Occurrence of severe medication adverse event**				
	No	Ref			
	Yes	1.12 (0.41,3.07)			
Jain, 2014[a] (Municipal Corporation Area, Western India)<br>*Outcome: Death, treatment failure, and loss to follow-up as a composite outcome* [56]		Values below are odds ratios			
	**Sex**				
	Female	Ref			
	Male	2.27 (1.1,4.7)*	0.03*		
	**Age (years)**				
	<=40	Ref			
	>40	1.56 (0.63,3.83)	0.34		
	**Radiological extent**				
	No bilateral cavity	Ref			
	Bilateral cavity	2.09 (0.87,5.04)	0.09		
	**Radiological improvement**				
	No	Ref			
	Yes	0.006 (0.0004,0.1)*	0.0004*		
	**Initial culture colony count**				
	1+/scanty	Ref			
	2+/3+	0.83 (0.41,1.67)	0.6		
	**Culture conversion within 3 months**				
	No	Ref			
	Yes	0.098 (0.042,0.23)*	<0.0001*		
	**Drug resistance pattern**				
	<3	Ref			
	>=3	0.87 (0.43,1.76)	0.7		
	**Concomitant disease**				
	No	Ref			
	Yes	1.45 (0.4,5.2)	0.57		
	**Smoking**				
	No	Ref			
	Yes	2.51 (1.18,5.38)*	0.02*		
	**Alcohol**				
	No	Ref			
	Yes	4.66 (1.64,13.26)*	0.004*		
	**Tobacco chewing**				
	No	Ref			

	Yes	1.49 (0.73,3.03)	0.27		
Janmeja, 2017[b] (Chandigarh) *Outcome: Death, treatment failure, modification of therapy, and loss to follow-up as a composite outcome* [57]		Values below are odds ratios		Values below are adjusted odds ratios	
	**Age[c,***]**				
	Per each year increase in age	1.03 (1.0,1.05)*	0.002*	1.05 (1.0,1.09)*	0.01*
	**Sex[***]**				
	Female	Ref			
	Male	1.26 (0.75,2.12)	0.38		
	**Unsuccessful outcome in previous anti-TB therapy[***]**				
	No	Ref			
	Yes	2.08 (1.17,3.67)*	0.01*		
	**Number of previous ATT courses >=1[***]**				
	No	Ref			
	Yes	2.05 (1.15,3.67)*	0.01*		
	**3 month sputum culture conversion[***]**				
	Yes	Ref			
	No	1.79 (0.85,3.78)	0.12		
	**Treatment adherence[***]**				
	Yes	Ref			
	No	3.74 (2.04,6.84)*	<0.001*	4.52 (1.21,16.6)*	0.02*
	**Adverse drug reaction**				
	No	Ref			
	Yes	1.79 (0.9,3.56)	0.98		
	**BMI[c]**				
	Per each unit decrease in BMI	1.15 (1.06,1.24)*	0.001*		
	**Diabetes**				
	No	Ref			
	Yes	2.33 (0.98,5.15)	0.053		
	**Smoking**				
	No	Ref			
	Yes	1.25 (0.7,2.23)	0.46		
	**Alcoholic**				
	No	Ref			
	Yes	1.23 (0.66,2.3)	0.48		
	**Hemoglobin[c,***]**				
	Per each unit decrease in hemoglobin	1.25 (1.09,1.43)*	0.001*		

	**S. albumin[c,***]**				
	Per each unit decrease in albumin	2.26 (1.34,3.80)*	0.002*	3.71 (1.22,11.3)*	0.02*
Johnson, 2022 (Karnataka) *Outcome: Death, treatment failure, loss to follow-up, and TB relapse as a composite outcome* [58]		Values below are odds ratios		Values below are adjusted odds ratios	
	**Sex**				
	Male	Ref		Ref	
	Female	0.93 (0.42,2.06)	0.86	0.97 (0.32,2.91)	0.95
	**Age (years)**				
	0-24	Ref		Ref	
	25-44	1.78 (0.49,6.43)	0.38	1.95 (0.39,9.82)	0.42
	45-64	1.57 (0.60,4.13)	0.36	1.60 (0.49,5.23)	0.44
	>65	2.23 (0.79,6.29)	0.13	2.64 (0.78,8.88)	0.12
	**Weight at initiation (kg)**				
	<50	Ref		Ref	
	>50	0.85 (0.42,1.72)	0.64	1.08 (0.46,2.52)	0.87
	**Occupation**				
	Business	Ref		Ref	
	Farmer	1.60 (0.28,9.13)	0.60	1.81 (0.23,13.91)	0.57
	Other	1.39 (0.27,7.26)	0.69	1.84 (0.25,13.80)	0.55
	Professional	2.14 (0.38,12.16)	0.39	3.34 (0.41,27.23)	0.26
	Students	4.36 (0.56,33.65)	0.16	3.41 (0.34,34.37)	0.30
	Worker	2.29 (0.19,27.99)	0.52	1.65 (0.10,28.75)	0.73
	**Form of TB**				
	PTB+	Ref		Ref	
	EPTB+	2.01 (0.43,9.33)	0.38	1.15 (0.18,7.35)	0.88
	Both	2.62 (0.40,17.31)	0.32	1.31 (0.12,14.40)	0.83
	**Type of TB case**				
	New	Ref		Ref	
	Retreatment case	18.69 (2.47,141.36)*	0.005*	13.13 (1.05,164.08)*	0.05*
	**HIV status**				
	Negative	Ref		Ref	
	Positive	3.80 (1.01,14.36)*	0.05	3.99 (0.83,19.16)	0.08
	**Year**				
	2015	Ref			
	2016	1.82 (0.39,8.55)	0.45	2.68 (0.44,16.46)	0.29
	2017	0.90 (0.39,2.09)	0.81	0.75 (0.27,2.08)	0.58
	2018	1.81 (0.68,4.80)	0.24	2.02 (0.67,6.14)	0.21
	**Drug resistance**				
	Non-MDR	Ref		Ref	
	MDR	0.37 (0.13,1.08)	0.07	0.34 (0.11,1.12)	0.08

	**Sputum smear**				
	Negative	Ref		Ref	
	Positive	0.50 (0.19,1.33)	0.16	0.48 (0.11,2.07)	0.33
	**Comorbidity**				
	Absent	Ref		Ref	
	Present	1.60 (0.47,5.39)	0.45	0.29 (0.03,3.25)	0.32
	**Type of comorbidity**				
	Anemia	Ref		Ref	
	Diabetes	0.69 (0.15,3.21)	0.63	0.26 (0.03,2.77)	0.27
	Drug-induced complications	0.95 (0.22,4.11)	0.94	0.26 (0.03,2.61)	0.25
	Hypothyroidism	0.99 (0.16,6.21)	0.99	0.36 (0.03,4.68)	0.43
	Kidney-related problems	0.25 (0.05,1.39)	0.11	0.03 (0.003,0.45)*	0.01*
	Weight loss	0.41 (0.11,1.49)	0.17	0.14 (0.02,1.21)	0.07
	Others	0.200 (0.03,1.41)	0.11	0.03 (0.001,0.42)*	0.01*
Kalagani, 2022 (Telangana) *Outcome: Death as a single outcome* [59]				Values below are adjusted odds ratios	
	**Age**				
	Per year increase in age			1.48 (0.36,6.13)	0.58
	**BMI**				
	Per unit increase in BMI			0.11 (0.05,0.28)*	<0.01*
	**Diabetic Status**				
	Non-diabetic			Ref	
	Diabetic			3.62 (0.90,14.50)	0.07
	**HIV Status**				
	Non-HIV			Ref	
	HIV			11.43 (2.65,49.24)*	0.01*
	**Tobacco Users**				
	Non-users			Ref	
	Users			21.38 (4.22,108.15)*	0.0002*
	**Alcohol Intake**				
	No			Ref	
	Yes			151.67 (7.30,3149.54)*	0.001*
	**Past History of TB**				
	No			Ref	
	Yes			0.094 (0.023,0.39)*	0.001
	**Cavitary lesions on Chest x-ray radiography**				
	No			Ref	
	Yes			17.71 (3.39,92.65)*	0.007*
Kalagani, 2022 (Telangana)					

Outcome: Loss to follow-up as a single outcome [59]					
	**Age**				
	Per year increase in age			4.52 (0.23,90.1)	0.32
	**BMI**				
	Per unit increase in BMI			0.53 (0.05,6.06)	0.61
	**Diabetic Status**				
	Non-diabetic			Ref	
	Diabetic			0.61 (0.03,12.41)	0.75
	**HIV Status**				
	Non-HIV			Ref	
	HIV			0.86 (0.04,17.74)	0.92
	**Tobacco Users**				
	Non-users			Ref	
	Users			15 (1.22,184.81)*	0.03*
	**Alcohol Intake**				
	No			Ref	
	Yes			27.5 (2.03,372.58)*	0.01*
	**Past History of TB**				
	No			Ref	
	Yes			1.62 (0.09,32.91)	0.75
	**Cavitary lesions on Chest x-ray radiography**				
	No			Ref	
	Yes			23.15 (1.14,467.95)*	0.04*
Kandi, 2021[a] (Telangana) *Outcome: Treatment failure, switch to XDR treatment, LTFU and death as a composite outcome* [60]		Values below are odds ratios			
	**Sex**				
	Female	Ref			
	Male	1.99 (1.31,3.02)*	0.001*		
	**Age (years)**				
	Younger than 50				
	Older than 50	1.93 (0.99,3.74)	0.053		
	**Treatment initiation**				
	Less than 1 month	Ref			
	More than 1 month	1.25 (0.67,2.32)	0.48		
	**Resistance**				
	Rifampin resistance	Ref			
	Rifampin and isoniazid resistance	0.98 (0.65,1.48)	0.98		
	**Weight**				

	Healthy weight, overweight, and obese	Ref			
	Underweight	2.00 (1.29,3.08)*	0.002*		
	**Diabetic status**				
	Non-diabetic	Ref			
	Diabetic	0.71 (0.33,1.53)	0.38		
	**Thyroid**				
	Euthyroid	Ref			
	Hypothyroid	0.80 (0.50,1.28)	0.34		
	**HIV reactivity**				
	Non-reactive	Ref			
	Reactive	1.34 (0.46,3.90)	0.59		
Keshari, 2023[a] (New Delhi) *Outcome: Death, treatment failure, modification of therapy, and loss to follow-up as a composite outcome* [61]		Values below are odds ratios			
	**Age (years)**				
	≤20	Ref			
	21-40	0.37 (0.09,1.45)	0.15		
	41-60	1.08 (0.27,4.25)	0.91		
	>60	1.80 (0.26,12.50)	0.55		
	**Sex**				
	Female	Ref			
	Male	1.85 (0.60,5.69)	0.28		
	**Symptoms**				
	Fever	Ref			
	Cough	0.85 (0.42,1.74)	0.67		
	Sputum	0.80 (0.39,1.64)	0.54		
	Hemoptysis	1.89 (0.66,5.38)	0.23		
	Chest pain	9.45 (1.87,47.85)*	0.007*		
	Loss of appetite	6.30 (1.17,34.03)*	0.03*		
	Loss of weight	2.10 (0.80,5.49)	0.13		
	**Smoking status**	1.05 (0.46,2.42)	0.91		
	Never and former smokers	Ref			
	Current smokers	19.95 (5.44,73.10)*	<0.0001*		
	**Alcohol Intake**				
	Never	Ref			
	Current or stopped one year ago	5.17 (1.87,14.29)*	0.002*		
	**Socio-Economic Class**				
	Upper and middle	Ref			
	Lower	18.12 (4.78,68.71)*	<0.0001*		

	**History of contact of TB case**				
	No	Ref			
	Yes	33.60 (9.15,123.32)*	<0.0001*		
	**Presumptive DR TB suspect criteria**				
	Other DR-TB suspect criteria	Ref			
	Any TB patient who is a household contact of MDR-TB case	46.95 (10.94,201.48)*	<0.0001*		
	**Past history of ATT**				
	Absent	Ref			
	Present	3.17 (0.84,11.92)	0.09		
	**Number of times ATT taken**				
	Once	Ref			
	More than once	0.90 (0.31,2.67)	0.85		
	**Full course treatment taken**				
	Yes	Ref			
	No	57.00 (11.52,281.92)*	<0.0001*		
	**Hemoglobin (g/dl)**				
	Normal (12-16)	Ref			
	Reduced (<12)	33.12 (4.20,261.46)*	0.0009*		
	**TLC ($10^3/mm^3$)**				
	Normal	Ref			
	Reduced	6.11 (1.06,35.12)*	0.04*		
	Increased	24.44 (5.74,104.02)*	<0.0001*		
	**Platelets ($10^3/mm^3$)**				
	Normal	Ref			
	Reduced	3.86 (1.21,12.28)*	0.02*		
	**LFT**				
	WNL	Ref			
	Deranged	14.75 (2.85,76.42)*	0.001*		
	**KFT**				
	WNL	Ref			
	Deranged	12.00 (1.27,113.74)*	0.03*		
	**Baseline sputum smear status**				
	<1+	Ref			
	>1+	12.76 (4.03,40.36)*	<0.0001*		
	**BMI (Kg/m^2)**				
	>17.5	Ref			
	<17.5	22.26 (6.58,75.30)*	<0.0001*		
	**Baseline chest X-ray findings**				

	Minimal	Ref			
	Moderately advanced	19.39 (1.07,351.00)*	0.04*		
	Far advanced	715.00 (27.58,18533.29)*	0.0001*		
	**Cavity at baseline chest X-ray**				
	Absent	Ref			
	Present	11.56 (3.83,34.89)*	<0.0001*		
	**Extent of disease at baseline chest X-ray**				
	Less extensive	Ref			
	More extensive	49.17 (9.60,251.86)*	<0.0001*		
	**Weight at 3 months follow-up**				
	Increased	Ref			
	Reduced or same	19.89 (2.35,168.40)*	0.006*		
	**Sputum smear status at 3 months follow-up**				
	Negative	Ref			
	Positive	29.00 (5.21,161.44)*	0.0001*		
	**CXR findings at 3 months follow-up**				
	Minimal	Ref			
	Moderately advanced and far advanced	174.00 (16.27,1860.68)*	<0.0001*		
	**Symptoms at 3 months follow-up**				
	Improved	Ref			
	Same or worsen	27.60 (3.23,236.15)*	0.003*		
	**Weight at 6 months follow-up**				
	Increased	Ref			
	Reduced or same	27.27 (2.98,249.95)*	0.003*		
	**Sputum smear status at 6 months follow-up**				
	Negative	Ref			
	Positive	270.60 (11.49,6373.34)*	0.0005*		
	**CXR findings at 6 months follow-up**				
	Minimal	Ref			
	Moderately advanced and far advanced	533.00 (19.64,14468.01)*	0.0002*		
	**Symptoms at 6 months follow-up**				
	Improved	Ref			
	Same or worsen	28.00 (4.28,183.09)*	0.0005*		
Kiran B., 2022[a] (New Delhi) *Outcome: Death, modification of therapy, and loss to follow-up as a composite outcome* [62]		Values below are odds ratios		Values below are adjusted odds ratios	
	**Age (years)**				

	<45	Ref			
	≥45	1.94 (0.54,6.91)	0.31		
	**Sex**				
	Female	Ref			
	Male	0.92 (0.32,2.67)	0.88		
	**Occupation**				
	Employed	Ref			
	Unemployed	0.80 (0.25,2.52)	0.38		
	**Social class**				
	Upper	Ref			
	Lower	1.25 (0.30,5.26)	0.76		
	**BMI (kg/m^2)**				
	Normal weight (≥18.5 kg/m^2)	Ref		Ref	
	Underweight (<18.5 kg/m^2)	7.33 (2.19,24.50)*	0.001*	0.42 (0.03,5.43)	0.51
	**History of smoking**				
	No	Ref			
	Yes	1.54 (0.45,5.25)	0.49		
	**History of ATT**				
	No	Ref			
	Yes	0.37 (0.12,1.13)	0.08		
	**Number of ATT courses in the past**				
	Single	Ref			
	Multiple times	16.29 (1.65,160.53)*	0.02*		
	**Past history of 2nd line ATT**				
	No	Ref			
	Yes	4.92 (0.19,130.37)	0.34		
	**TB contact history**				
	No	Ref			
	Yes	1.05 (0.33,3.37)	0.93		
	**Diabetes mellitus**				
	No	Ref			
	Yes	0.75 (0.15,3.72)	0.72		
	**Anaemia**				
	No	Ref		Ref	
	Yes	31.25 (5.96,163.92)*	<0.0001*	25.73 (1.18,751.73)*	0.05*
	**Total body protein**				
	<6 g/dl	Ref			
	>6 g/dl	0.52 (0.14,1.84)	0.31		
	**Chest X-ray laterality**				
	Unilateral	Ref		Ref	

	Bilateral	10.35 (2.78,38.49)*	0.0005*	1.36 (0.03,62.94)	0.87
	**Cavity**				
	No	Ref			
	Yes	1.70 (0.65,4.49)	0.28		
	**Extent if yes for cavity**				
	Single	Ref			
	Multiple	3.18 (0.53,19.05)	0.20		
	**CXR severity**				
	Minimal	Ref			
	Moderately advanced	3.00 (0.31,28.84)	0.34		
	Far advanced	25.50 (2.36,275.75)*	0.008*		
	**Far advanced vs. non far advanced CXR severity**				
	Non far advanced	Ref		Ref	
	Far advanced	10.20 (2.74,38.01)*	0.0005*	3.66 (0.07,197.87)	0.52
	**Sputum smear status**				
	Scanty positive	Ref			
	1+	0.23 (0.01,5.99)*	0.37		
	2+	0.33 (0.01,9.57)	0.52		
	3+	0.71 (0.02,22.34)	0.84		
	**Sputum conversion at end of 3rd month**				
	Negative	Ref			
	Positive	5.40 (0.52,56.60)	0.16		
	**Sputum conversion at end of 4th month**				
	Negative	Ref			
	Positive	27.00 (2.09,348.68)*	0.01*		
Kiran B., 2022 (New Delhi) *Outcome: Death as a single outcome* [62]		Values below are odds ratios		Values below are adjusted odds ratios	
	**BMI (kg/m^2)**				
	Normal weight (≥18.5 kg/m^2)	Ref		Ref	
	Underweight (<18.5 kg/m^2)	27.00 (1.45,501.51)*	0.03*	8.19 (0.63,107.23)	0.11
	**Anemia**				
	No	Ref		Ref	
	Yes	15.00 (0.81,277.29)	0.07	29.43 (1.25,864.86)*	0.05*
	**Chest X-ray laterality**				
	Unilateral			Ref	
	Bilateral			2.78 (0.13,60.99)	0.52
	**CXR severity**				
	Minimal	Ref			
	Moderately advanced	0.85 (0.03,23.06)	0.92		

	Far advanced	6.29 (0.31,127.07)	0.23		
	**Far advanced vs. non far advanced CXR severity**				
	Non far advanced	Ref		Ref	
	Far advanced	13.20 (1.46,119.52)*	0.02*	3.90 (0.13,119.83)	0.44
Kumar R., 2023[a] (Tamil Nadu)<br>*Outcome: Death and loss to follow-up as a composite outcome* [63]		Values below are odds ratios			
	**Age (years)**				
	31-50	Ref			
	Not 31-50	1.13 (0.42,3.01)	0.81		
	**Sex**				
	Female	Ref			
	Male	2.30 (0.81,6.50)	0.12		
	**Diabetic**				
	Non-diabetic	Ref			
	Diabetic	0.92 (0.35,2.41)	0.87		
	**Newly Diagnosed**				
	Newly diagnosed	Ref			
	Previously treated	1.98 (0.75,5.26)	0.17		
Kumari, 2022 (Andhra Pradesh)<br>*Outcome: Death, treatment failure, and loss to follow-up as a composite outcome* [64]					
	**Age (years)**				
	15-44	Ref			
	45-64	0.79 (0.41,1.51)	0.48		
	≥65	0.96 (0.37,2.46)	0.93		
	**Sex**				
	Female	Ref			
	Male	1.74 (0.84,3.61)	0.13		
	**HIV**				
	Non-reactive	Ref			
	HIV	2.66 (1.32,5.38)*	0.006*		
	**Diabetic**				
	No	Ref			
	Yes	0.62 (0.21,1.82)	0.38		
	**BMI**				
	≥18	Ref			
	<18	1.34 (0.28,6.48)	0.72		
	**First Line DST (Isoniazid KAT G)**				

	Susceptible	Ref			
	Resistance	3.42 (0.79,14.73)	0.10		
	Unknown	0.63 (0.03,11.83)	0.76		
	**First Line DST (Isoniazid Inh A)**				
	Susceptible	Ref			
	Resistance	2.23 (0.42,11.82)	0.34		
	Unknown	0.62 (0.03,11.60)	0.75		
	**Weight Change (Intensive phase)**				
	No change	Ref			
	Any loss	3.40 (0.05,219.14)	0.56		
	Any gain	0.75 (0.15,3.61)	0.72		
	**Weight Change (CP)**				
	No change	Ref			
	Any loss	1.00 (0.0039,255.6264)	1.00		
	Any gain	0.19 (0.00,9.65)	0.41		
	**Treatment Adherence (IP)**				
	<7 missed doses	Ref			
	>7 missed doses	6.00 (0.92,39.19)	0.06		
	**Treatment Adherence (CP)**				
	<7 missed doses	Ref			
	>7 missed doses	1.34 (0.40,4.50)	0.63		
Lohiya, 2020 (Delhi) *Outcome: Death, treatment failure, modification of therapy, treatment discontinuation for reasons other than adverse drug reactions, and loss to follow-up as a composite outcome* [65]		Values below are relative risk ratios		Values below are adjusted relative risk ratios	
	**Sex**				
	Female	Ref		Ref	
	Male	1.2 (0.8,1.8)	0.2	1.3 (0.9,1.9)	0.13
	**Age (years)**				
	Younger than 15	Ref		Ref	
	15 and older	1.6 (0.9,2.7)	0.08	1.6 (0.8,3.0)	0.14
	**TB site**				
	Lymph node	Ref			
	Other	1.2 (0.8,1.7)	0.4		
	**Basis of diagnosis**				
	CBNAAT	Ref			
	Other basis	1.0 (0.6,1.7)	0.9		
	**History of previous TB**				

	No	Ref		Ref	
	Yes	2.3 (1.0,5.7)*	0.04*	2.1 (1.1,4.8)*	0.03*
	**Adverse reaction**				
	Yes	Ref		Ref	
	No	1.3 (0.8,2.1)	0.2	1.4 (0.9,2.2)	0.15
	**Weight**				
	<30 kg	Ref		Ref	
	31-50 kg	2.0 (1.1,4.0)*	0.02*	1.8 (1.2,3.4)*	0.02*
	50 kg or more	1.8 (0.9,3.6)	0.09	1.6 (0.8,3.0)	0.1
	**HIV status**				
	Negative	Ref			
	Positive	2.0 (0.9,4.5)	0.3		
	**Diabetic status**				
	Non-diabetic	Ref		Ref	
	Diabetic	1.8 (0.9,3.8)	0.2	1.9 (0.9,3.4)	0.18
	**TB center (in Delhi)**				
	Drug-resistant TB Center 1	Ref		Ref	
	Drug-resistant TB Center 2 [receives many patients from outside of Delhi]	1.6 (1.0,2.5)	0.06	1.5 (1.0,2.5)*	0.05*
	Drug-resistant TB Center 3	1.05 (0.6,1.9)	0.8	1.0 (0.7,2.0)	0.8
Nair, 2016 (Tamil Nadu) *Outcome: Death, treatment failure, switched to extensively drug resistant TB treatment, interrupted treatment due to reasons other than adverse drug reaction, transfer out, and loss to follow-up as a composite outcome* [66]		Values below are relative risk ratios		Values below are adjusted relative risk ratios	
	**Sex**				
	Female	Ref		Ref	
	Male	1.4 (1.2,1.8)*	<0.001*	1.4 (1.2,1.6)*	0.001*
	**Age (years)**				
	15-44	Ref		Ref	
	>=45	1.1 (0.9,1.3)	0.13	1.1 (0.9,1.4)	0.08
	**Presumptive MDRTB criteria**				
	Failure	Ref			
	Retreatment	1.2 (0.9,1.4)	0.1		
	Smear-positive follow-up	0.9 (0.6,1.4)	0.67		
	Other	0.6 (0.3,1.4)	0.15		
	**Drug Resistance status**				
	Rifampicin+Isoniazid	Ref			
	Rifampicin only	1.0 (0.9,1.2)	0.63		

	**Type of diagnostic test**				
	Rapid	Ref			
	CDST	1.1 (0.9,1.3)	0.13		
	**Time to treatment**				
	<=14 days	Ref		Ref	
	15-30 days	1.1 (0.9,1.5)	0.28	1.1 (0.9,1.4)	0.36
	31+ days	1.3 (1.0,1.6)*	0.04*	1.3 (1.1,1.6)*	0.04*
	**HIV Status**				
	Non-reactive	Ref			
	Reactive	1.0 (0.7,1.5)	0.9		
Parmar, 2018 (7 States) *Outcome: Death treatment failure, and loss to follow-up as a composite outcome* [68]		Values below are odds ratios			
	**Sex**				
	Female	Ref			
	Male	1.38 (1.08,1.76)*	0.01*		
	**Age (years)**				
	<15	Ref			
	15-44	1.07 (0.27,4.33)	0.92		
	45-64	1.06 (0.26,4.36)	0.94		
	>64	2.58 (0.53,12.65)	0.24		
	**Initial registration type**				
	Relapse	Ref			
	Loss to follow-up	1.26 (0.85,1.86)	0.25		
	Treatment after failure	1.01 (0.77,1.33)	0.93		
	New contacts	1.44 (0.69,3.02)	0.34		
	Others	1.74 (0.85,3.57)	0.13		
	**Previous number of treatment episodes**[c]				
	Per each increase in number of previous treatment episodes	1.29 (1.09,1.53)*	<0.001*		
	**Retreatment regimen taken twice**				
	No	Ref			
	Yes	0.8 (0.56,1.15)	0.23		
	**Duration of previous episodes**[c]				
	Per each increase in month of duration of previous episode	0.98 (0.95,1.0)*	0.02*		
	**Treatment deal by drug susceptibility testing method**				
	Phenotypic	Ref			
	Genotypic	1.06 (0.1,11.05)	0.96		

	**Ethambutol resistance by Lowenstein-Jensen culture**				
	Resistance	Ref			
	Susceptible	0.65 (0.48,0.89)*	0.01*		
	**Streptomycin resistance by Lowenstein-Jensen culture**				
	Resistance	Ref			
	Susceptible	0.63 (0.39,1.03)	0.07		
	**First-line drug resistance**				
	Rifampin only	Ref			
	Isoniazid and rifampin only	1.67 (0.74,3.77)	0.22		
	Isoniazid and rifampin combination	0.73 (0.27,1.98)	0.53		
	Rifampin combination	2.72 (0.57,12.96)	0.21		
	**Treatment adherence (intensive phase)**				
	<7 missed doses	Ref			
	>=7 missed doses	2.76 (2.03,3.77)*	<0.001*		
	**Treatment adherence (continuation phase)**				
	<7 missed doses	Ref			
	>=7 missed doses	1.51 (1.15,1.98)*	<0.001*		
	**HIV status**				
	Negative	Ref			
	Positive	1.08 (0.5,2.34)	0.84		
	Unknown	0.65 (0.26,1.59)	0.35		
	**Diabetes**				
	No	Ref			
	Yes	0.96 (0.63,1.44)	0.83		
	**Body mass index**				
	>=18	Ref			
	<18	1.64 (1.28,2.11)*	<0.001*		
	**Cavitation**				
	No	Ref			
	Yes	1.1 (0.87,1.39)	0.44		
	**Weight change at 6 months**				
	None	Ref			
	Any loss	0.9 (0.59,1.37)	0.63		
	Any gain	1.05 (0.76,1.47)	0.75		
	**Weight change at 12 months**				
	None	Ref			
	Any loss	1.38 (0.81,2.33)	0.23		

	Any gain	0.92 (0.59,1.43)	0.7		
	**Source of most recent previous treatment**				
	Government	Ref			
	Private	0.89 (0.6,1.33)	0.57		
	Other	0.53 (0.25,1.14)	0.1		
Parmar, 2018 (7 States) *Outcome: Death as a single outcome* [68]				Values below are adjusted odds ratios	
	**Sex**				
	Female			Ref	
	Male			1.01 (0.75,1.36)	0.94
	**Age (years)**				
	<15			Ref	
	15-44			0.68 (0.16,2.98)	0.61
	45-64			0.84 (0.19,3.78)	0.82
	>64			2.29 (0.41,12.79)	0.35
	**Initial registration type**				
	Relapse			Ref	
	Loss to follow-up			1.33 (0.83,2.13)	0.24
	Treatment after failure			0.96 (0.67,1.36)	0.82
	New contacts			1.94 (0.82,4.56)	0.13
	Others			2.68 (1.2,5.99)*	0.02*
	**Previous number of treatment episodes[c]**				
	Per each increase in number of previous treatment episodes			1.18 (0.93,1.48)	0.17
	**Retreatment regimen taken twice**				
	No			Ref	
	Yes			1.04 (0.67,1.61)	0.88
	**Duration of previous episode[c]**				
	Per each increase in month of duration of previous episode			0.99 (0.97,1.02)	0.59
	**Cavitation**				
	No			Ref	
	Yes			1.41 (1.05,1.91)*	0.02*
	Unknown			1.53 (0.78,3.01)	0.22
	**Treatment delay by drug susceptibility testing method**				
	Phenotypic			Ref	
	Genotypic			1.41 (1.05,1.91)*	0.02*
	**Ethambutol resistance by Lowenstein-Jensen culture**				

	Resistance			Ref	
	Susceptible			1.53 (0.78,3.01)	0.22
	**Streptomycin resistance by Lowenstein-Jensen culture**				
	Resistance			Ref	
	Susceptible			0.45 (0.24,0.84)*	0.01*
	Unknown			0.6 (0.04,9.84)	0.72
	**First-line drug resistance**				
	Rifampin only			Ref	
	Isoniazid and rifampin only			1.48 (0.61,3.59)	0.39
	Isoniazid and rifampin combination			0.53 (0.17,1.71)	0.29
	Rifampin combination			3.62 (0.64,20.55)	0.15
	**Treatment adherence (intensive phase)**				
	<7 missed doses			Ref	
	>=7 missed doses			2.13 (1.46,3.12)*	<0.001*
	Unknown			0.08 (0.02,0.27)*	<0.001*
	**Treatment adherence (continuation phase)**				
	<7 missed doses			Ref	
	>=7 missed doses			0.98 (0.67,1.42)	0.91
	Unknown			22.06 (8.22,59.21)*	<0.001*
	**HIV Status**				
	Negative			Ref	
	Positive			0.76 (0.26,2.26)	0.63
	Unknown			0.73 (0.22,2.37)	0.6
	**Diabetes**				
	No			Ref	
	Yes			0.92 (0.53,1.61)	0.78
	**Body mass index**				
	>=18			Ref	
	<18			4.89 (3.4,7.06)*	<0.001*
	**Weight change at 6 months**				
	No change			Ref	
	Any loss			0.81 (0.49,1.35)	0.41
	Any gain			0.92 (0.62,1.37)	0.68
	**Weight change at 12 months**				
	No change			Ref	
	Any loss			1.34 (0.7,2.55)	0.38
	Any gain			0.52 (0.3,0.9)*	0.02*

	**Source of the most recent previous treatment**				
	Government			Ref	
	Private			0.86 (0.52,1.43)	0.55
	Other			0.52 (0.19,1.42)	0.2
Parmar, 2018 (7 States) *Outcome: Treatment failure as a single outcome* [68]				Values below are adjusted odds ratios	
	**Sex**				
	Female			Ref	
	Male			1.6 (1.1,2.33)*	0.02*
	**Age (years)**				
	<15			Ref	
	15-44			0.67 (0,infinity)	0.99
	45-64			0.55 (0,infinity)	0.99
	>64			0.12 (0,infinity)	0.99
	**Initial registration type**				
	Relapse			Ref	
	Loss to follow-up			1.19 (0.66,2.13)	0.57
	Treatment after failure			0.95 (0.61,1.47)	0.82
	New contacts			1.96 (0.67,5.72)	0.22
	Others			1.62 (0.53,4.94)	0.39
	**Previous number of treatment episodes**[c]				
	Per each increase in number of previous treatment episodes			1.04 (0.78,1.39)	0.78
	**Retreatment regimen taken twice**				
	No			Ref	
	Yes			1.12 (0.64,1.93)	0.7
	**Duration of previous episode**[c]				
	Per each increase in month of duration of previous episode			0.99 (0.96,1.03)	0.75
	**Cavitation**				
	No			Ref	
	Yes			0.89 (0.62,1.26)	0.5
	Unknown			0.27 (0.06,1.23)	0.09
	**Treatment delay by drug susceptibility testing method**				
	Phenotypic			Ref	
	Genotypic			0.55 (0.02,13.63)	0.72
	**Ethambutol resistance by Lowenstein-Jensen culture**				
	Resistance			Ref	

	Susceptible			0.6 (0.37,0.97)*	0.04*
	**Streptomycin resistance by Lowenstein-Jensen culture**				
	Resistance			Ref	
	Susceptible			0.63 (0.31,1.29)	0.21
	Unknown			4.3 (0.17,107.94)	0.38
	**First-line drug resistance**				
	Rifampin only			Ref	
	Isoniazid and rifampin only			1.57 (0.46,5.35)	0.48
	Isoniazid and rifampin combination			0.72 (0.16,3.28)	0.67
	Rifampin combination			3.46 (0,infinity)	0.98
	**Treatment adherence (intensive phase)**				
	<7 missed doses			Ref	
	>=7 missed doses			2.29 (1.46,3.59)*	<0.001*
	Unknown			0.14 (0.01,1.8)	0.13
	**Treatment adherence (continuation phase)**				
	<7 missed doses			Ref	
	>=7 missed doses			1.9 (1.29,2.79)*	<0.001*
	Unknown			1.89 (0.37,9.64)	0.44
	**HIV Status**				
	Negative			Ref	
	Positive			1.82 (0.7,4.71)	0.22
	Unknown			0.38 (0.04,3.31)	0.38
	**Diabetes**				
	No			Ref	
	Yes			1.31 (0.71,2.41)	0.39
	**Body mass index**				
	>=18			Ref	
	<18			1.82 (1.2,2.76)*	0.01*
	**Weight change at 6 months**				
	No change			Ref	
	Any loss			1.99 (1.04,3.79)*	0.04*
	Any gain			1.59 (0.92,2.74)	0.1
	**Weight change at 12 months**				
	No change			Ref	
	Any loss			1.12 (0.52,2.41)	0.77
	Any gain			1.08 (0.56,2.09)	0.81
	**Source of the most recent previous treatment**				

	Government			Ref	
	Private			1.64 (0.9,2.99)	0.1
	Other			0.18 (0.04,0.86)*	0.03*
Parmar, 2018 (7 States) *Outcome: Loss to follow-up as a single outcome* [68]				Values below are adjusted odds ratios	
	**Sex**				
	Female			Ref	
	Male			1.91 (1.38,2.66)*	<0.001*
	**Age (years)**				
	<15			Ref	
	15-44			2.3 (0.23,23.23)	0.48
	45-64			2.19 (0.21,22.46)	0.51
	>64			6.38 (0.53,76.79)	0.14
	**Initial registration type**				
	Relapse			Ref	
	Loss to follow-up			1.32 (0.81,2.14)	0.27
	Treatment after failure			0.72 (0.5,1.05)	0.09
	New contacts			1.2 (0.49,2.95)	0.69
	Others			0.84 (0.31,2.3)	0.74
	**Previous number of treatment episodes**[c]				
	Per each increase in number of previous treatment episodes			0.96 (0.75,1.22)	0.72
	**Retreatment regimen taken twice**				
	No			Ref	
	Yes			1.02 (0.63,1.64)	0.94
	**Duration of previous episode**[c]				
	Per each increase in month of duration of previous episode			1 (0.97,1.03)	0.89
	**Cavitation**				
	No			Ref	
	Yes			0.98 (0.72,1.34)	0.91
	Unknown			1.72 (0.88,3.36)	0.11
	**Treatment delay by drug susceptibility testing method**				
	Phenotypic			Ref	
	Genotypic			1.04 (0.07,15.31)	0.98
	**Ethambutol resistance by Lowenstein-Jensen culture**				
	Resistance			Ref	
	Susceptible			0.61 (0.41,0.91)*	0.02*

	**Streptomycin resistance by Lowenstein-Jensen culture**				
	Resistance			Ref	
	Susceptible			0.6 (0.32,1.15)	0.12
	Unknown			2.41 (0.16,35.11)	0.52
	**First-line drug resistance**				
	Rifampin only			Ref	
	Isoniazid and rifampin only			2.19 (0.86,5.57)	0.1
	Isoniazid and rifampin combination			0.72 (0.22,2.41)	0.6
	Rifampin combination			4.88 (0.82,29.03)	0.08
	**Treatment adherence (intensive phase)**				
	<7 missed doses			Ref	
	>=7 missed doses			4.36 (2.97,6.39)*	<0.001*
	Unknown			0.05 (0.01,0.16)*	<0.01*
	**Treatment adherence (continuation phase)**				
	<7 missed doses			Ref	
	>=7 missed doses			1.85 (1.27,2.70)*	<0.001*
	Unknown			36.69 (13.7,98.4)*	<0.001*
	**MDR-TB patients with baseline second line drug susceptibility testing**				
	Ofloxacin susceptible			Ref	
	Ofloxacin resistant			3.19 (1.40,7.28)*	0.006*
	**HIV Status**				
	Negative			Ref	
	Positive			0.55 (0.17,1.77)	0.32
	Unknown			1.24 (0.42,3.69)	0.7
	**Diabetes**				
	No			Ref	
	Yes			0.99 (0.56,1.73)	0.97
	**Body mass index**				
	>=18			Ref	
	<18			1.6 (1.12,2.29)*	0.01*
	**Weight change at 6 months**				
	No change			Ref	
	Any loss			0.63 (0.36,1.11)	0.11
	Any gain			0.95 (0.62,1.47)	0.83
	**Weight change at 12 months**				
	No change			Ref	
	Any loss			2.27 (0.97,5.34)	0.06

	Any gain			1.7 (0.81,3.57)	0.16
	**Source of the most recent previous treatment**				
	Government			Ref	
	Private			1.07 (0.62,1.83)	0.81
	Other			0.54 (0.19,1.56)	0.26
Rupani, 2020 (Gujarat) *Outcome: Treatment discontinuation/non-adherence/interruption as a single outcome* [70]				Values below are adjusted odds ratios	
	**Age (years)[c]**				
	Per each increase in year			1.02 (0.95,1.09)	0.65
	**Marital status**				
	Unmarried			1.48 (0.28,7.7)	0.64
	Married			Ref	
	**Years of schooling[c]**				
	Per each increase in year of schooling			1.09 (0.87,1.35)	0.45
	**Per Capita Income[c]**				
	Per each increase in capita income (unit of change undefined)			1.0 (0.99,1.0)	0.64
	**Duration of Treatment[c]**				
	Per each increase in treatment duration (unit of change undefined)			1.06 (0.97,1.14)	0.17
	**Intensive phase of MDR-TB**				
	Intensive phase			2.31 (0.49,10.8)	0.29
	Continuous phase			Ref	
	**Adverse drug reaction**				
	Present			1.44 (0.27,7.6)	0.67
	Absent			Ref	
Saha, 2017[a] (Maharashtra) *Outcome: Death, treatment failure, and loss to follow-up as a composite outcome* [71]		Values below are odds ratios			
	**Age (years)**				
	<45	Ref		Ref	
	>=45	6.67 (1.55,28.62)*	0.01*	15.3 (1.69,138.99)*	0.02*
	**Sex**				
	Female	Ref		Ref	
	Male	2.86 (0.85,9.63)	0.1	2.09 (0.42,10.36)	0.37
	**Past history of TB**				
	Absent	Ref			
	Present	1.43 (0.44,4.67)	0.75		

	**Site of disease**				
	Extrapulmonary	Ref			
	Pulmonary	2.63 (0.28,24.55)	0.67		
	**Comorbidities**				
	Without comorbidities	Ref			
	With comorbidities	2.86 (0.70,11.65)	0.21		
	**Resistance to >=5 drugs**				
	No	Ref		Ref	
	Yes	9.51 (2.50,38.18)*	0.001*	12.43 (2.04,75.88)*	0.01*
Sharma, 2020 (Delhi) *Outcome: Death, treatment failure, switched to extensively drug-resistant TB therapy, treatment stopped due to adverse drug reaction, and loss to follow-up as a composite outcome* [118]				Values below are adjusted odds ratios	
	**Age (years)**				
	Younger than 18			Ref	
	18-34			1.19 (0.86,1.65)*	<0.001*
	35 and older			2.1 (1.47,3)*	<0.001*
	**Sex**				
	Female			Ref	
	Male			1.4 (1.12,1.75)*	0.002*
	**Pretreatment body mass index**				
	Normal/overweight (body mass index >=18.5)			Ref	
	Undernourished (body mass index <18.5)			1.88 (1.48,2.38)*	<0.001*
Shringarpure, 2015 (Gujarat) *Outcome: Loss to follow-up as a single outcome* [72]		Values below are hazard ratios		Values below are adjusted hazard ratios	
	**Age (years)**				
	<35	Ref		Ref	
	>35	0.99 (0.91,1.08)		0.98 (0.78,1.23)	
	**Sex**				
	Female	Ref		Ref	
	Male	1.14 (0.86,1.50)		1.08 (0.49,1.67)	
	**Rural residence**				
	No	Ref		Ref	
	Yes	0.95 (0.83,1.08)		1.02 (0.76,1.38)	
	**Living below poverty line**				
	No	Ref		Ref	
	Yes	0.97 (0.74,1.27)		0.85 (0.49,1.47)	

	**Previous TB treatment**				
	No	Ref		Ref	
	Yes	0.99 (0.991,0.997)*		0.92 (0.83,1.02)	
	**Chest X-ray bilateral involvement**				
	No	Ref		Ref	
	Yes	1.03 (0.95,1.11)		0.88 (0.57,1.36)	
	**Chest X-Ray Cavitation**				
	No	Ref		Ref	
	Yes	0.99 (0.92,1.08)		1.08 (0.72,1.64)	
	**Culture Conversion Time >4 months**				
	No	Ref		Ref	
	Yes	1.10 (1.06,1.15)*		1.34 (1.21,1.49)*	
	**No adverse events in IP**				
	No	Ref		Ref	
	Yes	0.75 (0.55,1.00)		1.13 (0.61,2.09)	
	**No adverse events in CP**				
	No	Ref		Ref	
	Yes	0.99 (0.65,1.49)		2.63 (0.35,19.95)	
	**Weight >45 kg**				
	No	Ref		Ref	
	Yes	0.89 (0.77,1.04)		1.19 (0.87,1.61)	
	**Ambulatory initiation of treatment**				
	No	Ref		Ref	
	Yes	1.68 (0.62,4.55)		2.63 (1.01,6.86)*	
	**Different DOT provider in IP and CP**				
	No	Ref		Ref	
	Yes	1.15 (1.11,1.19)*		1.27 (1.18,1.38)*	
Velayutham, 2022 (Over 18 states)<br>*Outcome: Death, treatment failure, modification of therapy, and loss to follow-up as a composite outcome*		Values below are odds ratios		Values below are adjusted odds ratios	
	**Age (years)**				
	≤40	Ref		Ref	
	>40	1.14 (1.03,1.25)*	0.01*	1.08 (0.94,1.23)	0.27
	**Sex**				
	Female	Ref		Ref	
	Male	1.61 (1.43,1.82)*	<0.001	1.59 (1.34,1.88)*	<0.001*
	**Body weight (kg)**				
	≥70	Ref			
	46-70	0.9 (0.4,1.8)	0.84		

	30-45	1.19 (0.6,2.35)	0.60		
	16-29	1.5 (0.68,3.4)	0.31		
	**HIV status**				
	Non-reactive	Ref		Ref	
	Reactive	1.53 (1.17,2.0)*	0.002*	1.57 (1.12,2.2)*	0.008*
	**Diabetes**				
	Non-diabetic	Ref			
	Diabetic	1.06 (0.9,1.24)	0.43		
	**Current tobacco use**				
	No	Ref			
	Yes	1.45 (1.25,1.67)*	<0.001*		
	**Alcohol intake**				
	No	Ref			
	Yes	1.6 (1.3,1.89)	<0.001*		
	**Resistance to FQ**				
	No	Ref			
	Yes	3.19 (2.49,4.10)*	<0.001*		
	**INH resistance on FL-LPA**				
	High-level *kat*G resistance	Ref			
	Low-level *inh*A resistance	1.14 (1.01,1.27)*	0.02*		
	**INH resistance on FL-LPA**				
	Low-level *inh*A resistance			Ref	
	High-level *kat*G resistance			1.23 (1.07,1.42)*	0.005*
	**Social habit**				
	No tobacco or alcohol use			Ref	
	Only alcohol use			1.43 (1.08,1.9)*	0.01*
	Only tobacco use			1.19 (0.94,1.51)	0.15
	Both alcohol and tobacco use			1.42 (1.16,1.74)*	0.001*
Studies of people with drug-susceptible TB which include people with new and previously treated TB					
Babiarz, 2014 (Bihar) *Population: New and previously treated TB patients (sputum smear positive pulmonary, sputum smear negative pulmonary, and extrapulmonary) as a combined population* *Outcome: Loss to follow-up as a single outcome (i.e., treatment discontinuation <25 weeks after initiation)* [3]		Values below are odds ratios		Values below are adjusted odds ratios	
	**Sex**				

	Female	Ref		Ref	
	Male	1.29 (0.85,1.95)		1.32 (0.82,2.14)	
	**Age[c]**				
	Per each year increase in age	0.96 (0.91,1.00)		0.96 (0.91,1.01)	
	**Religion**				
	Non-Hindu	Ref		Ref	
	Hindu	0.83 (0.51,1.35)		0.81 (0.47,1.40)	
	**Caste/tribe**				
	Other	Ref		Ref	
	Scheduled caste/tribe/OBC	0.87 (0.53,1.44)		0.84 (0.47,1.50)	
	**Number of children in household[c]**				
	Per each increase in number of children	0.95 (0.82,1.09)		1.02 (0.86,1.21)	
	**Education**				
	Yes	Ref		Ref	
	No	1.01 (0.96,1.06)		0.99 (0.93,1.06)	
	**Poor**				
	No	Ref		Ref	
	Yes	1.09 (0.68,1.74)		0.97 (0.56,1.66)	
	**Middle income**				
	No	Ref		Ref	
	Yes	0.70 (0.36,1.36)		0.86 (0.48,1.54)	
	**Household size[c]**				
	Per each person increase in household	0.97 (0.88,1.06)		0.96 (0.86,1.07)	
	**Prior TB treatment episode**				
	No	Ref		Ref	
	Yes	6.15 (2.60,14.53)*		4.77 (1.98,11.53)*	
	**Prior TB and completed prior treatment**				
	No	Ref		Ref	
	Yes	0.23 (0.09,0.60)*		0.22 (0.88,0.59)	
	**Total weeks from symptom onset to treatment initiation[c]**				
	Per each week increase in symptoms	1.00 (0.95,1.05)		1.02 (0.97,1.07)	
	**Number of symptoms at treatment initiation**				
	>=5	Ref		Ref	
	2 or fewer	1.18 (0.75,1.85)		1.62 (0.96,2.73)	
	3 to 4	1.38 (0.74,2.56)		1.54 (0.83,2.86)	
	**Spent money on travel costs to reach care**				
	No	Ref		Ref	

	Yes	2.70 (1.07,6.82)*		2.55 (1.03,6.33)*	
	**Number of providers visited[d]**				
	Per each increase in number of providers visited	4.68 (2.64,8.27)*		3.67 (1.94,6.95)*	
	**Spending on treatment or medication fees**				
	No	Ref		Ref	
	Yes	6.83 (2.18,21.41)*		4.6 (1.38,15.40)*	
Bagchi, 2010[a] (Maharashtra) *Population: New and previously treated sputum smear positive pulmonary TB patients, excluding new patients in the first two months of therapy* *Outcome: Medication non-adherence as a single outcome (i.e., at least one week's worth of missed TB medication doses in any treatment month)* [4]		Values below are odds ratios		Values below are adjusted odds ratios	
	**Sex**				
	Female	Ref			
	Male	1.9 (1.0,3.6)*	Not reported		
	**Household members**				
	0-3	Ref			
	>3	0.7 (0.3,1.3)	Not reported		
	**Any history of smoking**				
	No	Ref		Ref	
	Yes	2.4 (1.2,5.1)	Not reported	1.9 (0.8,4.5)	Not reported
	**Any history of tobacco-chewing**				
	No	Ref			
	Yes	1.3 (0.7,2.3)	Not reported		
	**Any history of alcohol use**				
	No	Ref		Ref	
	Yes	4.8 (2.2,10.3)*	Not reported	3.6 (1.5,8.3)*	Not reported
	**Hid disease from family members**				
	No	Ref		Ref	
	Yes	0.5 (0.2,1.7)	Not reported	1.9 (0.8,4.2)	Not reported
	**Treatment duration perceived as being too long**				
	No	Ref			

	Yes	1.7 (1.0,2.9)*	Not reported		
	**Treatment discontinued once symptoms resolved**				
	No	Ref			
	Yes	1.5 (0.8,3.1)	Not reported		
	**Knows about problems with stopping treatment early**				
	Yes	Ref			
	No	1.5 (0.9,2.6)	Not reported		
	**Feels confident about completing treatment**				
	No	1		Ref	
	Yes	1.8 (0.5,7.0)	Not reported	2.5 (0.5,11)	
	**Travel mode to health center**				
	Walking	Ref			
	Other	0.6 (0.2,1.2)	Not reported		
	**Travel to health center is a problem**				
	No	Ref			
	Yes	1.8 (0.8,3.9)	Not reported		
	**Has concerns about transportation to the health center**				
	No	Ref			
	Somewhat or very concerned	1.1 (0.5,2.4)	Not reported		
	**Concerned about distance to the health center**				
	No	Ref			
	Somewhat or very concerned	1.3 (0.6,2.4)	Not reported		
	**Concerned about the time to reach the health center**				
	No	Ref			
	Somewhat or very concerned	1.3 (0.7,2.6)	Not reported		
	**Doctor communicated problems related to stopping medication early**				
	Yes	Ref			
	No	1.3 (0.8,2.2)	Not reported		
	**Where patient gets most TB information**				
	DOTS center	Ref			

	Other sources	1.2 (0.6,2.2)	Not reported		
	**Missed treatment due to lack of drug availability**				
	No	Ref		Ref	
	Yes	5.6 (1.8,15)	Not reported	5.1 (1.6,16)*	Not reported
Balasubramanian, 2004 (Tamil Nadu)<br>*Population: New and previously treated TB patients (smear positive pulmonary, smear negative pulmonary, and extrapulmonary) as a combined population*<br>*Outcome: Loss to follow-up as a single outcome* [5]		Values below are odds ratios		Values below are adjusted odds ratios	
	**Sex**				
	Female	Not reported		Ref	
	Male	Not reported		2.1 (1.1,3.9)*	0.02*
	**Employment**				
	Unemployed	Not reported		Ref	
	Employed	Not reported		1.7 (1.1,2.4)*	<0.01*
	**History of TB**				
	No previous history of treatment	Not reported		Ref	
	Previous history of treatment	Not reported		3.9 (2.6,5.6)*	<0.001*
	**Alcohol**				
	No alcohol use	Not reported		Ref	
	Alcoholism	Not reported		2.2 (1.5,3.3)*	<0.001*
Banerjee, 2020 (West Bengal)<br>*Population: New and previously treated TB patients (pulmonary and extrapulmonary) as a combined population*<br>*Outcome: Death, treatment failure, and loss to follow-up as a composite outcome*<br>[75]		Values below are odds ratios		Values below are adjusted odds ratios	
	**Sex**				
	Female	Ref		Ref	
	Male	2.6 (1.1,6.2)*		3.07 (1.11,8.52)*	
	**Type of family**				
	Nuclear	Ref		Ref	
	Joint	2.8 (1.2,6.7)*		4.71 (1.66,13.39)*	
	**Educational status**				
	Literate	Ref		Ref	
	Illiterate	2.78 (1.14,6.78)*		1.19 (0.36,3.9)	

	**Type of disease**				
	Extrapulmonary	Ref		Ref	
	Pulmonary	3.9 (1.4,11.0)*		1.9 (0.52,6.9)	
	**Category of disease**				
	New	Ref		Ref	
	Previously treated	3.6 (1.5,8.5)*		3.39 (1.23,9.34)*	
	**Smoking tobacco use**				
	Never smoker	Ref		Ref	
	Ever smoker	2.5 (1.2,5.8)*		1.5 (0.3,6.9)	
	**Perceived discrimination from family, neighborhood residents, or workplace colleagues**				
	No	Ref		Ref	
	Yes	2.26 (1.09,5.65)*		2.61 (1.04,7.84)*	
Bhagyalaxmi, 2010[a] (Gujarat) *Population: New and previously treated TB patients (pulmonary and extrapulmonary) as a combined population* *Outcome: Death, treatment failure, and loss to follow-up as a composite outcome* [76]		Values below are odds ratios			
	**Type of DOTS supporter**				
	TB health visitor	Ref			
	Non-TB health visitors (e.g., Anganwadi workers, community volunteers, private practitioners)	0.46 (0.22,0.98)*	0.045*		
Bhargava, 2013 (Chhattisgarh) *Population: New and previously treated TB patients (sputum smear positive pulmonary, sputum smear negative pulmonary, and extrapulmonary) as a combined population* *Outcome: Death as a single outcome* [77]		Values below are odds ratios		Values below are adjusted odds ratios	
	**Age**				
	Per 10 years increase in age	1.48 (1.10,1.97)*		1.34 (1.10,1.79)*	
	**Sex**				
	Female	Ref		Ref	
	Male	1.90 (1.03,3.52)*		1.59 (0.67,3.75)	
	**Pre-treatment weight**				
	Per 5 kg increase in weight	0.82 (0.70,1.00)		0.59 (0.47,0.77)*	

	**Height at diagnosis**				
	Per cm increase in height	1.03 (0.99,1.06)		1.06 (1.00,1.12)	
	**Body mass index**				
	Per unit increase in BMI	0.80 (0.71,0.91)*		0.78 (0.68,0.90)*	
	**Sputum status**				
	Smear negative	Ref		Ref	
	Smear positive	0.83 (0.48,1.42)		1.00 (0.55,1.85)	
	**Sputum grade**				
	0 (smear negative)	Ref		1 (reference)	
	1+	0.72 (0.33,1.50)		0.85 (0.37,1.94)	
	2+	0.52 (0.22,1.24)		0.67 (0.26,1.68)	
	3+	1.16 (0.62,2.18)		1.56 (0.78,3.10)	
	**HIV status**				
	HIV negative	Ref		Ref	
	HIV positive	16.60 (6.45,42.7)*		19.78 (6.83,57.31)*	
	**Treatment category**				
	New case	Ref		Ref	
	Previously treated	1.49 (0.80,2.80)		1.24 (0.62,2.48)	
	**Location of residence**				
	Group 1 (Village health program and outreach clinics)	Ref			
	Group 2 (Within 20 km radius of hospital)	1.61 (0.67,3.83)			
	Group 3 (Beyond 20 km radius of hospital)	0.91 (0.46,1.83)			
	**Family history of TB**				
	No history of TB	Ref			
	History of TB	1.14 (0.63,2.09)			
Brahmapurkar[a], 2017 (Chhattisgarh) *Population: New and previously treated TB patients (sputum smear positive pulmonary, sputum smear negative pulmonary, and extrapulmonary) as a combined population Outcome: Death, treatment failure, loss to follow-up, and transferred as a composite outcome* [78]		Values below are odds ratios			
	**Age (years)**				
	<40	Ref			
	>40	2.4 (1.49,3.86)*	0.0003*		
	**Sex**				
	Female	Ref			
	Male	1.53 (0.91,2.58)	0.11		

	**Category**				
	New	Ref			
	Previously treated	2.54 (1.52,4.23)*	0.0003*		
	**TB case**				
	New smear positive	Ref			
	New smear negative	0.72 (0.40,1.29)	0.27		
	Previously treated	2.11 (1.16,3.83)*	0.01*		
Cox, 2021 (2 Indian states) *Population: Men with drug-susceptible or presumed drug-susceptible pulmonary TB* *Outcome: Treatment failure as a single outcome* [79]		Values below are odds ratios		Values below are adjusted odds ratios	
	**Ever had a drink of alcohol**				
	No	Ref		Ref	
	Yes	2.06 (1.30,3.28)*	0.002*	1.60 (0.95,2.68)	0.08
	**Unhealthy alcohol use (AUDIT-C >= 4)**				
	No	Ref		Ref	
	Yes	1.92 (1.24,2.96)*	0.003*	1.36 (0.83,2.22)	0.25
	**AUDIT-C continuous[c]**				
	No	Ref		Ref	
	Yes	1.09 (1.03,1.16)*	0.002*	1.04 (0.98,1.11)	0.18
	**Underweight (BMI<18.5) and severe alcohol use (AUDIT-C > 4)**				
	Not underweight and not severe alcohol use	Not reported		Ref	
	Not underweight but with severe alcohol use	Not reported		0.62 (0.26,1.50)	0.29
	Underweight but not severe alcohol use	Not reported		0.77 (0.39,1.50)	0.44
	Underweight and severe alcohol use	Not reported		1.68 (0.92,3.07)	0.09
	**Severely underweight (BMI<16.5) and severe alcohol use (AUDIT-C > 4)**				
	Not severely underweight and not severe alcohol use	Not reported		Ref	
	Not severely underweight but with severe alcohol use	Not reported		0.99 (0.54,1.83)	0.98
	Severely Underweight but not severe alcohol use	Not reported		0.67 (0.32,1.41)	0.29
	Severely Underweight and severe alcohol use	Not reported		1.76 (0.96,3.25)	0.07
Dandona, 2004 (4 Indian States) *Population: New and previously treated pulmonary TB patients as a*				Values below are adjusted odds ratios	

combined population Outcome: Loss to follow-up as a single outcome [80]					
	**Age (years)**				
	16-30			Ref	
	31-50			1.58 (0.92,1.46)	
	>50			1.1 (0.85,1.43)	
	**Sex**				
	Female			Ref	
	Male			2.15 (1.76,2.62)*	
	**Marital status**				
	Never married			Ref	
	Ever married			1.69 (1.21,2.37)*	
	**Literacy**				
	Literate			Ref	
	Illiterate			1.17 (0.97,1.42)	
	**Monthly family income (Indian rupees)**				
	>5000			Ref	
	3001-5000			1.24 (0.75,2.06)	
	<=3000			1.3 (0.86,1.96)	
	**Satisfaction with behavior of DOTS provider**				
	Satisfied			Ref	
	Neither satisfied nor dissatisfied			3.51 (2.21,5.57)*	
	Dissatisfied			8.68 (4.41,17.09)*	
	Refused to answer			9.03 (3.33,24.52)*	
	**Distance to DOTS provider from home**				
	<=10 km			Ref	
	>10 km			1.15 (0.79,1.67)	
	**Type of DOTS provider**				
	Health facility staff			Ref	
	Anganwadi worker			0.69 (0.56,0.86)*	
	Community volunteer			0.83 (0.58,1.2)	
	Family member			0.63 (0.28,1.39)	
	Medicine with self			0.26 (0.15,0.47)*	
	Refused to answer			0.17 (0.05,0.59)*	
	**Patient informed that TB is curable**				
	Yes			Ref	
	No			1.75 (1.11,2.75)*	
	Doesn't remember			2.29 (1.2,4.26)*	
	**Patient informed of treatment duration**				

	Yes			Ref	
	No			3.11 (2.08,4.66)*	
	Doesn't remember			2.59 (1.66,4.02)*	
Das, 2014[a] (Nagaland)<br>*Population: New and previously treated TB patients (smear positive pulmonary, smear negative pulmonary, and extrapulmonary) as a combined population*<br>*Outcome: Death, treatment failure, and loss to follow-up as a composite outcome* [81]		Values below are odds ratios		Values below are adjusted odds ratios	
	**Age (years)[c]**				
	Per each year increase in age	Not reported	Not reported	1.03 (1.01,1.05)*	
	**Sex**				
	Female	Ref		Ref	
	Male	2.08 (1.13,3.81)	0.02*	1.5 (0.78,2.86)	
	**Residence**				
	Rural	Ref			
	Semi-urban	1.24 (0.68,2.26)	0.48		
	**TB site**				
	Pulmonary	Ref			
	Extrapulmonary	1.85 (0.82,4.16)	0.14		
	**TB treatment regimen**				
	Category I	Ref		Ref	
	Category II	2.37 (1.28,4.38)*	0.006*	1.81 (0.95,3.47)	
Dey, 2021 (West Bengal)<br>*Population: New and previously treated TB patients as a combined population*<br>*Outcome: Loss to follow-up as a single outcome* [82]		Values below are odds ratios			
	**Age (years)**				
	0-14	Ref			
	15-29	5.33 (1.31,21.71)*	0.02*		
	30-44	5.66 (1.39,23.10)*	0.02*		
	45-59	5.31 (1.30,21.69)	0.02*		
	60 and above	3.83 (0.92,15.91)	0.06		
	**Sex**				
	Female	Ref			
	Male	1.25 (0.99,1.58)	0.06		
	Transgender	7.34 (0.30,181.33)	0.22		
	**Education**				
	Graduate and Above	Ref			

	Illiterate	1.55 (0.90,2.68)	0.11		
	Primary School	1.56 (0.93,2.62)	0.09		
	High School	1.06 (0.61,1.87)	0.83		
	**Occupation**				
	Earning Family Members	Ref			
	Dependent Family Members	0.75 (0.61,0.93)*	0.01*		
	Student	0.49 (0.31,0.77)*	0.002*		
	**Migratory Family**				
	No	Ref			
	Yes	1.12 (0.67,1.88)	0.66		
	**Tobacco Use**				
	No	Ref			
	Yes	2.08 (1.67,2.58)*	<0.0001*		
	**Alcohol Use**				
	No	Ref			
	Yes	2.26 (1.73,2.94)*	<0.0001*		
	**Type of Diet**				
	Non-Veg	Ref			
	Veg	0.67 (0.27,1.65)	0.38		
	**Type of TB**				
	DS-TB	Ref			
	DR-TB	2.70 (1.92,3.80)*	<0.0001*		
	**Phase of Treatment**				
	IP	Ref			
	CP	2.43 (1.97,3.00)*	<0.0001*		
	**History of TB**				
	No	Ref			
	Yes	1.51 (1.18,1.95)*	0.001*		
	**Diabetes status**				
	No	Ref			
	Yes	1.07 (0.81,1.42)	0.63		
	Unknown	0.75 (0.53,1.07)	0.11		
	**HIV Status**				
	Negative	Ref			
	Positive	1.13 (0.35,3.63)	0.84		
	Unknown	0.94 (0.70,1.27)	0.70		
	**Current Symptoms**				
	No Symptoms	Ref			
	Have some symptoms	1.66 (1.32,2.10)*	<0.0001*		
	**Ambulatory**				
	Yes	Ref			
	No	1.44 (1.01,2.06)*	0.04*		

Gopi, 2007 (Tamil Nadu)<br>*Population: New and previously treated pulmonary TB patients (sputum smear positive and sputum smear negative) during the intensive phase of therapy as a combined population*<br>*Outcome: Treatment non-adherence as a single outcome* [83]		Values below are odds ratios		Values below are adjusted odds ratios	
	**Sex**				
	Female	Ref			
	Male	1.13 (0.9,1.43)	Not reported		
	**Age (years)**				
	<=45	Ref			
	>45	1.19 (0.96,1.47)	Not reported		
	**Education**				
	Literate	Ref		Ref	
	Illiterate	1.47 (1.19,1.83)*	<0.01*	1.33 (1.07,1.66)*	<0.05*
	**Occupation**				
	Employed	Ref			
	Unemployed	1.02 (0.82,1.27)	Not reported		
	**Case type**				
	Previously treated pulmonary TB patients	Ref		2.29 (1.51,3.47)*	<0.001*
	New sputum smear positive pulmonary TB patients	1.86 (1.27,2.75)*	<0.01*	1.31 (1.05,1.64)*	<0.05*
	New sputum smear-negative pulmonary TB patients	2.43 (1.66,3.56)*	<0.01*	Ref	
	**Loss of wages due to directly observed therapy**				
	Yes	Ref			
	No	1.28 (0.78,2.12)	Not reported		
	**Problem in taking drugs**				
	No	Ref			
	Yes	1.07 (0.87,1.32)			
	**Directly observed therapy interferes with daily activities**				
	No	Ref			
	Yes	1.49 (1.03,2.16)*	<0.05*		
	**Smoking**				
	No	Ref			

	Yes	1.16 (0.94,1.43)	Not reported		
	**Alcoholism**				
	Yes	Ref			
	No	1.06 (0.84,1.33)	Not reported		
	**Difficulty accessing the health facility**				
	No	Ref		Ref	
	Yes	2.96 (2.06,4.24)*	<0.01*	3.02 (2.10,4.34)*	<0.001*
	**Need escort to get to the directly observed therapy center**				
	Yes	Ref			
	No	1.09 (0.69,1.73)	Not reported		
	**Directly observed therapy center type**				
	Government	Ref		Ref	
	Non-government	2.14 (1.73,2.65)*	<0.01*	2.11 (1.70,2.61)*	<0.001*
Huddart, 2021 (Bihar) *Population: New and previously treated TB patients as a combined population* *Outcome: Death as a single outcome* [85]		Values below are unweighted model adjusted hazard ratios[d]		Values below are weighted model adjusted hazard ratios[e]	
	**Sex**				
	Male	Ref		Ref	
	Female	0.75 (0.54,1.02)		0.71 (0.47,1.05)	
	**Age**				
	Per each year increase in age	1.03 (1.02,1.03)*		1.03 (1.02,1.04)*	
	**Residence**				
	Out of Patna	Ref		Ref	
	Rural Patna	0.90 (0.57,1.33)		0.99 (0.58,1.56)	
	Urban Patna	0.72 (0.51,1.08)		0.79 (0.53,1.15)	
	**Slum residence**				
	Non-slum	Ref		Ref	
	Slum	0.81 (0.60,1.07)		0.72 (0.51,1.00)	
	**Treatment category**				
	New	Ref		Ref	
	Retreatment	1.37 (0.86,2.14)		1.34 (0.74,2.26)	
	**Type of TB**				
	Pulmonary	Ref		Ref	
	Extrapulmonary	0.95 (0.68,1.31)		0.84 (0.57,1.19)	
Islam, 2023[b] (West Bengal) *Population: New and previously treated TB patients as a combined population*		Values below are odds ratios		Values below are adjusted odds ratios	

Outcome: Medication non-adherence as a single outcome [86]					
	**Age (years)**				
	≤45	Ref			
	>45	0.43 (0.17,1.08)			
	**Sex**				
	Male	Ref		Ref	
	Female	0.87 (0.29,2.66)		0.93 (0.22,3.96)	
	**Literacy**				
	Illiterate	Ref		Ref	
	Literate	1.75 (0.7,4.38)		1.85 (0.61,5.56)	
	**Occupation**				
	Employed	Ref		Ref	
	Unemployed	0.72 (0.28,1.84)		0.95 (0.31,2.88)	
	**SES**				
	Upper, upper middle, or middle	Ref		Ref	
	Lower middle or lower	1.02 (0.38,2.72)		0.84 (0.28,2.57)	
	**Past h/o alcoholism**				
	No	Ref		Ref	
	Yes	1.32 (0.53,3.31)		1.16 (0.37,3.67)	
	**Clinical type of TB**				
	Pulmonary	Ref		Ref	
	Extra-pulmonary	0.32 (0.06,1.6)		0.12 (0.02,0.77)*	
Jaggarajamma, 2007[a] (Tamil Nadu) *Population: New and previously treated TB patients (sputum smear positive pulmonary, sputum smear negative pulmonary, and extrapulmonary) as a combined population* *Outcome: Loss to follow-up as a single outcome* [87]		Values below are odds ratios			
	**Sex**				
	Female	Ref			
	Male	3.5 (2.12,5.77)*	<0.0001*		
	**Age (years)**				
	<45	Ref			
	>=45	1.31 (0.95,1.81)	0.1		
	**Education**				
	Literate	Ref			
	Illiterate	1.19 (0.83,1.72)	0.34		
	**Occupation**				
	Employed	Ref			

	Unemployed	0.73 (0.49,1.1)	0.13		
	**Category of treatment**				
	Category I (new sputum smear-positive pulmonary TB patients)	Ref			
	Category II (previously treated sputum smear-positive pulmonary TB patients)	2.68 (1.81,3.95)*	<0.0001*		
	Category III (new extrapulmonary and sputum smear-negative pulmonary TB patients)	0.54 (0.36,0.83)*	0.004*		
	**Sputum smear status**				
	Sputum smear-negative pulmonary TB patients	Ref			
	Sputum smear-positive pulmonary TB patients	2.06 (1.46,2.92)*	<0.0001*		
	**Type of disease**				
	Extrapulmonary	Ref			
	Pulmonary	2.46 (1.11,5.46)*	0.03*		
	**Alcoholism**				
	No	Ref			
	Yes	2.63 (1.83,3.77)*	<0.0001*		
	**DOT reported as being convenient by the patient**				
	Yes	Ref			
	No	2.16 (1.28,3.62)*	0.004*		
Jan Swasthya Sahyog, 2018[a] (Chhattisgarh) *Population: New and previously treated TB patients (sputum smear positive pulmonary, sputum smear negative pulmonary, and extrapulmonary) as a combined population* *Outcome: Death, treatment failure, loss to follow-up, and transferred out as a composite outcome* [89]		Values below are odds ratios			
	**Pretreatment sputum status (among patients with sputum samples)**				
	Positive	Ref			
	Negative	1.04 (0.87,1.23)	0.69		
	**AFB Grade (among sputum positive patients)**				
	3+	Ref			
	1+	0.89 (0.70,1.13)	0.35		
	2+	0.80 (0.63,1.01)	0.06		

Jan Swasthya Sahyog, 2018 (Chhattisgarh)<br>*Outcome: Death, treatment failure, loss to follow-up, and transferred out as a composite outcome* [89]		Values below are relative risk ratios			
	**Age (years)**				
	<50	Ref			
	>=50	1.71 (1.45,2.02)*	<0.01*		
	**Sex (by treatment site)**				
	Male, secondary care hospital	Ref			
	Male, primary care clinic	0.56 (0.41,0.74)*	<0.01*		
	Female, secondary care hospital	0.77 (0.66,0.90)*	<0.01*		
	Female, primary care clinic	0.59 (0.42,0.84)*	<0.01*		
	**Treatment history**				
	No prior TB treatment	Ref			
	Any prior TB treatment	1.28 (1.00,1.63)*	0.05*		
	**Season and distance from treatment**				
	Winter and <45 minutes	Ref			
	Winter and 45 minutes-1.5 hours	1.01 (0.75,1.37)	0.93		
	Winter and 1.5-4 hours	1.31 (0.96,1.78)	0.08		
	Winter and >4 hours	1.13 (0.83,1.55)	0.43		
	Summer and <45 minutes	1.33 (1.00,1.77)*	0.05*		
	Summer and 45 minutes-1.5 hours	1.48 (1.08,2.05)*	0.02*		
	Summer and 1.5-4 hours	1.77 (1.24,2.52)*	<0.01*		
	Summer and >4 hours	2.49 (1.79,3.47)*	<0.01*		
	Monsoon and <45 minutes	1.21 (0.91,1.61)	0.19		
	Monsoon and 45 minutes-1.5 hours	1.02 (0.75,1.40)	0.86		
	Monsoon and 1.5-4 hours	1.30 (0.93,1.81)	0.13		
	Monsoon and >4 hours	1.35 (0.99,1.85)	0.06		
Jonnalagada, 2011 (Andhra Pradesh)<br>*Population: New and previously treated TB patients (sputum smear positive pulmonary, sputum smear negative pulmonary, and extrapulmonary) as a combined population*<br>*Outcome: Death, treatment failure, loss to follow-up, and transferred out as a composite outcome* [90]		Values below are odds ratios			
	**Type and category of TB**				
	Extrapulmonary TB patients	Ref			

	New sputum smear negative pulmonary TB patients	1.34 (1.06,1.68)*	0.01*		
	New sputum smear positive pulmonary TB patients	1.64 (1.35,2.00)*	<0.0001*		
	Previously treated TB patients	3.14 (2.68,3.69)*	<0.0001*		
Joseph, 2011[a] (Karnataka) *Population: New and previously treated sputum smear positive pulmonary TB patients as a combined population* *Outcome: Treatment failure and loss to follow-up as a composite outcome* [13]		Values below are odds ratios			
	**Sex**				
	Female	Ref			
	Male	1.33 (0.71,2.52)	0.37		
	**Location**				
	Rural	Ref			
	Urban	2.04 (1.25,3.31)*	0.004*		
	**Treatment category**				
	Category I (new sputum smear positive pulmonary TB patients)	Ref			
	Category II (previously treated sputum smear positive pulmonary TB patients)	3.81 (2.18,6.65)*	<0.0001*		
Kamble, 2022[b] (Haryana) *Population: New and previously treated TB patients (sputum smear positive pulmonary, sputum smear negative pulmonary, and extrapulmonary) as a combined population* *Outcome: Death, treatment failure, loss to follow-up, shift to category IV, and transferred out as a composite outcome* [91]		Values below are odds ratios		Values below are adjusted odds ratios	
	**Sex**				
	Male	Ref		Ref	
	Female	0.77 (0.62,0.96)*	0.02*	0.98 (0.77,1.24)	0.86
	**Age (years)**				
	15-19	Ref			
	20-24	1.01 (0.81,1.26)	0.95		
	**Category of treatment**				
	I	Ref		Ref	
	II	5.00 (3.92,6.37)*	<0.001*	1.25 (0.53,2.92)	0.61
	**Sputum result**				

	Negative	Ref		Ref	
	Scanty	1.92 (1.37,2.70)*	<0.001*	1.82 (0.87,3.79)	0.11
	1+	2.78 (2.00,3.85)*	<0.001*	1.49 (1.05,2.12)*	0.03*
	2+	3.23 (2.12,4.91)*	<0.001*	2.17 (1.55,3.05)*	<0.001*
	3+	2.44 (1.20,4.97)*	0.01*	2.38 (1.66,3.42)*	<0.001*
	**History of TB treatment**				
	Yes	Ref		Ref	
	No	0.22 (0.18,0.28)*	<0.001*	0.35 (0.15,0.80)*	0.01*
	**Type of TB**				
	Pulmonary	Ref		Ref	
	Extrapulmonary	0.13 (0.09,0.19)*	<0.001*	0.31 (0.04,2.36)	0.25
Karanjekar, 2014[a] (Maharashtra) *Population: New and previously treated TB patients (sputum smear positive pulmonary, sputum smear negative pulmonary, and extrapulmonary) as a combined population Outcome: Death, loss to follow-up, and transferred out as a composite outcome* [92]		Values below are odds ratios			
	**Age (years)**				
	15-24	Ref			
	25-34	0.92 (0.25,3.36)	0.89		
	35-44	1.01 (0.30,3.90)	0.9		
	45-54	1.46 (0.37,5.80)	0.59		
	54+	1.42 (0.33,6.00)	0.64		
	**Sex**				
	Female	Ref			
	Male	2.54 (0.99,6.48)	0.05		
	**Education**				
	More than high school	Ref			
	Up to high school	0.91 (0.26,3.26)	0.89		
	Illiterate	0.63 (0.16,2.52)	0.51		
	**Treatment category**				
	Category I (new sputum smear-positive pulmonary TB patients)	Ref			
	Category II (previously treated sputum smear-positive pulmonary TB patients)	1.95 (0.64,5.88)	0.24		
	Category III (new extrapulmonary and sputum smear-negative pulmonary TB patients)	1.17 (0.47,2.92)	0.74		
	**Type of TB**				

	Pulmonary	Ref			
	Extrapulmonary	3.86 (1.37,10.89)*	0.01*		
Kumar, 2018 (Madhya Pradesh)<br>*Population: New and previously treated TB patients (sputum smear positive pulmonary, sputum smear negative pulmonary, and extrapulmonary) as a combined population*<br>*Outcome: Treatment failure, LTFU, transferred out and death as a composite outcome* [93]		Values below are odds ratios			
	**Sex**				
	Female	Ref			
	Male	3.86 (2.09,7.12)*	<0.0001*		
	**Age (years)**				
	15 to 25	Ref			
	0 to 14	0.11 (0.01,0.86)	0.04		
	26 to 35	1.12 (0.55,2.29)	0.75		
	36 to 45	1.04 (0.44,2.42)	0.94		
	>45	3.05 (1.61,5.78)*	0.0006*		
	**Category of TB**				
	Category I (new TB patients)	Ref			
	Category II (previously treated TB patients)	2.35 (1.36,4.07)*	0.002*		
	**Site involvement**				
	Extrapulmonary TB patients	Ref			
	Pulmonary TB patients	3.66 (1.77,7.58)*	0.0005*		
Kuruva, 2020 (Telangana)<br>*Population: New and previously treated TB patients (sputum smear positive pulmonary and sputum smear negative pulmonary) as a combined population*<br>*Outcome: Death, treatment failure, and loss to follow-up as a composite outcome*<br>[94]		Values below are odds ratios			
	**Diabetes**				
	No	Ref			
	Yes	2.84 (1.21,6.66)*	0.02*		
Lata, 2021[a] (Jammu and Kashmir)<br>*Population: New and previously treated TB patients who completed at least 2 months of TB*		Values below are odds ratios			

therapy as a combined population<br>*Outcome: Medication non-adherence as a single outcome (i.e., self-reported non-ingestion of at least one medication dose as measured by the Morisky adherence scale)* [95]					
	**Sex**				
	Female	Ref			
	Male	1.25 (0.24,6.47)	0.79		
Maroof, 2022 (Uttarakhand)<br>*Population: New and previously treated TB patients as a combined population who could be interviewed*<br>*Outcome: Loss to follow-up as a single outcome* [96]		Values below are odds ratios		Values below are adjusted odds ratios	
	**Age (years)**				
	60 and above	Ref		Ref	
	40-59	2.1 (0.2, 19.4)		1.5 (0.1, 19.6)	
	18-39	2.1 (0.3, 16.9)		2.0 (0.1, 27.5)	
	**Sex**				
	Male	Ref		Ref	
	Female	1.2 (0.4, 3.4)		0.5 (0.1, 5.1)	
	**Area of residence**				
	Urban	Ref		Ref	
	Rural	1.6 (0.5, 4.5)		5.4 (1.0, 28.6)	
	**Occupation**				
	Not employed	Ref		Ref	
	Job (government or private)	1.3 (0.1, 12.7)		6.3 (0.1, 375.6)	
	Home maker	2.3 (0.3, 20.0)		12.2 (0.1, 1230.2)	
	Student	0.8 (0.1, 13.2)		1.5 (0.0, 120.5)	
	Self employed	1.4 (0.1, 13.8)		4.2 (0.1, 219.2)	
	Labourers	1.1 (0.1, 19.1)		4.9 (0.1, 408.7)	
	**Socio-economic class**				
	Upper class	Ref		Ref	
	Upper middle class	1.1 (0.1, 12.9)		1.0 (0.1, 16.5)	
	Middle class	1.4 (0.1, 14.1)		1.0 (0.1, 14.0)	
	Lower middle class	2.2 (0.3, 18.7)		1.1 (0.1, 15.4)	
	Lower class	3.3 (0.3, 38.5)		1.5 (0.1, 33.1)	
	**Family Support**				
	Yes	Ref		Ref	
	No	6.3 (0.6, 64.8)		29.2 (0.3, 2907.8)	
	**Social support**				

	Yes	Ref		Ref	
	No	5.8 (1.1, 30.5)*		4.2 (0.4, 49.0)	
	**Distance of DOTS centre is far away**				
	No	Ref		Ref	
	Yes	1.2 (0.4, 3.4)		1.5 (0.2, 13.7)	
	**Travel or transport difficult**				
	No	Ref		Ref	
	Yes	1.2 (0.4, 3.3)		1.0 (0.1, 8.9)	
	**Did you feel treatment is costly**				
	No	Ref		Ref	
	Yes	1.5 (0.4, 5.5)		1.7 (0.4, 7.5)	
	**Perception that too much time consumed for taking medicine**				
	No	Ref		Ref	
	Yes	1.2 (0.4, 3.5)		1.3 (0.4, 3.9)	
	**Did doctors mention the effects of stopping DOTS midway**				
	Yes	Ref		Ref	
	No	1.4 (0.5, 4.1)		1.3 (0.4, 3.8)	
	**Satisfied with the health service**				
	Satisfied	Ref		Ref	
	Dissatisfied	4.7 (0.5, 45.2)		5.0 (0.5, 52.5)	
	**Diabetes**				
	No	Ref		Ref	
	Yes	1.2 (0.2, 10.2)		1.3 (0.2, 12.1)	
	Don't know	0		0	
	**HIV**				
	No	Ref		Ref	
	Yes	0		0	
	Don't know	0		1.9 (0, 0)	
	**Depression**				
	No	Ref		Ref	
	Yes	7.0 (1.7, 29.3)*		7.5 (1.8, 32.0)*	
	Don't know	0		0	
	**DOTS category**				
	New	Ref		Ref	
	Previously treated	1.5 (0.5, 4.2)		2.5 (0.7, 9.5)	
	MDR TB	0		0	
	**Perception that treatment is too long**				
	No	Ref		Ref	

	Yes	1.4 (0.5, 3.9)		1.6 (0.4, 6.4)	
	**Perception that treatment should be discontinued once symptoms resolve**				
	No	Ref		Ref	
	Yes	9.3 (3.0, 29.0)*		12.6 (3.7, 42.8)*	
	**Experienced drug ingestion problems or side effects**				
	No	Ref		Ref	
	Yes	1.8 (0.6, 5.5)		2.1 (0.5, 8.8)	
	**Perception that drug frequency is high**				
	No	Ref		Ref	
	Yes	1.1 (0.4, 3.1)		0.8 (0.2, 3.8)	
	**Knowledge of how tuberculosis is transmitted**				
	Yes	Ref		Ref	
	No	3.9 (1.3, 11.7)*		4.6 (1.3, 16.5)*	
	**Smoking status**				
	Never	Ref		Ref	
	Past	1.5 (0.4, 5.8)		0.4 (0.1, 3.1)	
	Present	2.5 (0.5, 12.2)		0.4 (0.0, 10.2)	
	**Tobacco chewing**				
	Never	Ref		Ref	
	Past	1.8 (0.4, 8.4)		1.0 (0.1, 7.5)	
	Present	4.6 (1.1, 18.3)*		7.0 (0.5, 104.5)	
	**Alcohol use**				
	Never	Ref		Ref	
	Past	3.3 (1.0, 10.8)*		4.5 (0.9, 22.5)	
	Present	5.2 (1.2, 22.0)*		3.6 (0.4, 38.1)	
	**Substance abuse**				
	Absent	Ref		Ref	
	Present	1.3 (0.3, 6.2)		1.7 (0.3, 9.9)	
	**Know problems of stopping treatment (DOTS therapy)**				
	Yes	Ref		Ref	
	No	1.2 (0.4, 3.8)		1.4 (0.4, 5.2)	
	**Felt stigmatized**				
	No	Ref		Ref	
	Yes	1.6 (0.6, 4.6)		1.6 (0.4, 6.0)	
	**Perceived change in health status**				
	Improved	Ref		Ref	

	Not improved	2.1 (0.6, 7.8)		1.7 (0.4, 7.8)	
	**Missed treatment due to problem in accessing medicines during lockdown**				
	No	Ref		Ref	
	Yes	21.8 (6.2, 75.9)*		26.2 (6.4, 107.8)*	
	**Fear of contracting COVID-19 In institutional settings**				
	No	Ref		Ref	
	Yes	1.8 (0.6, 5.5)		1.9 (0.5, 7.7)	
	**Patients stranded in different geographic location due to lockdown**				
	No	Ref		Ref	
	Yes	1.3 (0.4, 3.8)		0.4 (0.1, 1.9)	
Mittal, 2011[a] (Uttar Pradesh) *Population: New and previously treated TB patients (sputum smear positive pulmonary, sputum smear negative pulmonary, and extrapulmonary) as a combined population* *Outcome: Death,treatment failure, loss to follow-up, and transferred out as a composite outcome* [98]		Values below are odds ratios			
	**Treatment category**				
	Category III (new extrapulmonary and sputum smear-negative pulmonary TB patients)	Ref			
	Category I (new sputum smear-positive pulmonary TB patients)	2.23 (1.49,3.34)*	0.0001*		
	Category II (previously treated sputum smear-positive pulmonary TB patients)	5.26 (3.45,8.02)*	<0.0001*		
	**Type of disease**				
	Extrapulmonary	Ref			
	Pulmonary	2.44 (1.67,3.56)*	<0.0001*		
	**Type of patient based on outcome of previous treatment**				
	New/transfer-in (i.e., no prior TB history)	Ref			
	Treatment after loss to follow-up	3.48 (2.28,5.33)*	<0.0001*		
	Treatment failure during prior treatment	2.94 (1.60,5.39)*	0.0005*		
	Relapse (i.e., previous treatment was completed)	24.67 (2.94,206.79)*	0.003*		

	Others	5.16 (3.31,8.06)*	<0.0001*		
	**Pre-treatment sputum smear status**				
	Not done	Ref			
	Positive smear by sputum microscopy	2.57 (1.75,3.79)*	<0.0001*		
	Negative smear by sputum microscopy	2.07 (1.36,3.15)*	0.0007*		
Mittal, 2011[a] (Uttar Pradesh) *Population: New and previously treated TB patients (sputum smear positive pulmonary, sputum smear negative pulmonary, and extrapulmonary) as a combined population Outcome: Treatment LTFU as a single outcome* [98]		Values below are odds ratios			
	**Age (years)**				
	<15	Ref			
	16-30	5.81 (2.46,13.70)*	0.0001*		
	31-45	5.20 (2.15,12.60)*	0.0003*		
	>45	8.11 (3.24,20.28)*	<0.0001*		
	**Sex**				
	Female	Ref			
	Male	2.19 (1.44,3.33)*	0.0002*		
	**Religion**				
	Hindu	Ref			
	Muslim	0.69 (0.41,1.15)	0.16		
	**Occupation**				
	Laborer	Ref			
	Service	0.90 (0.33,2.41)	0.83		
	Business	1.97 (0.93,4.17)	1.78		
	Housewife	0.51 (0.30,0.86)	2.51		
	Unemployed/retired	1.49 (0.65,3.45)	0.35		
	Student	0.37 (0.19,0.74)*	0.005*		
	Not defined (child under age of 5)	0.14 (0.02,1.04)	0.05		
	**Type of disease**				
	Extrapulmonary	Ref			
	Pulmonary	3.28 (1.88,5.74)*	<0.0001*		
	**Type of patients**				
	New/transfer-in	Ref			
	Treatment after default	4.14 (2.51,6.81)*	<0.0001*		
	Failure	2.40 (1.11,5.21)*	0.03*		
	Relapse	7.81 (1.70,35.77)*	0.008*		
	Others	6.42 (3.92,10.51)*	<0.0001*		
	**Treatment category**				

	Category I (new sputum smear-positive pulmonary TB patients)	Ref			
	Category II (previously treated sputum smear-positive pulmonary TB patients)	2.79 (1.82,4.27)*	<0.0001*		
	Category III (new extrapulmonary and sputum smear-negative pulmonary TB patients)	0.94 (0.57,1.53)	0.79		
	**Pre-treatment sputum status**				
	Positive	Ref			
	Negative	0.93 (0.62,1.39)	0.72		
Motappa, 2022[a] (Karnataka) *Population: New and previously treated TB patients (sputum smear positive pulmonary, sputum smear negative pulmonary, and extrapulmonary) as a combined population Outcome: Medication non-adherence as a single outcome (i.e., self-reported non-ingestion of at least one medication dose)* [99]		Values below are odds ratios		Values below are adjusted odds ratios	
	**Knowledge regarding causes of TB**				
	Correct			Ref	
	Incorrect			4.19 (4.19,8.54)*	
	**Knowledge regarding prevention of TB**				
	Correct			Ref	
	Incorrect			3.38 (1.81,6.30)*	
	**Knowledge regarding TB being curable**				
	Correct			Ref	
	Incorrect			11.57 (5.55,24.12)*	
	**Awareness of daily regimen**				
	Yes			Ref	
	No			7.99 (3.80,16.77)*	
	**Patient provider relationship**				
	Good			Ref	
	Bad			27.00 (6.03,120.92)*	
	**Side effects of TB medication**				
	No			Ref	
	Yes			5.24 (2.74,10.03)*	

	**Support of family during treatment duration**				
	Yes			Ref	
	No			9.22 (1.01,84.26)*	
	**Satisfied with treatment at the health center**				
	Yes			Ref	
	No			6.14 (2.59,14.57)*	
	**HIV status**				
	Seronegative			Ref	
	Seropositive			9.82 (2.02,47.71)*	
	**Feeling of depression and anxiety ever since disease was confirmed**				
	No			Ref	
	Yes			3.82 (2.04,7.15)*	
	**Being busy with other work**				
	No			Ref	
	Yes			11.72 (1.34,102.57)*	
	**Perception that treatment course is long**				
	No			Ref	
	Yes			4.87 (2.55,9.30)*	
	**Fearing of pill burden**				
	No			Ref	
	Yes			6.56 (3.40,12.65)*	
Mukhopadhyay, 2011[a] (West Bengal) *Population: New and previously treated TB patients (sputum smear positive pulmonary, sputum smear negative pulmonary, and extrapulmonary) as a combined population Outcome: Death, treatment failure, loss to follow-up, and transferred out as a composite outcome* [100]		Values below are odds ratios			
	**Age (years)**				
	<=19	Ref			
	20-60	4.15 (1.49,11.55)*	0.007*		
	>60	7.38 (2.25,24.15)*	0.001*		
	**Sex**				
	Female	Ref			
	Male	2.48 (1.47,4.20)*	0.0007*		
	**Residence**				
	Rural	Ref			

	Urban	1.17 (0.78,1.75)	0.45		
	**TB classification**				
	Extrapulmonary	Ref			
	New sputum negative	1.35 (0.49,3.69)	0.56		
	**Previous treatment history**				
	Other (i.e., smear negative or extrapulmonary)	Ref			
	Relapse (prior treatment completion)	2.16 (0.77,6.08)	0.14		
	Prior treatment failure	5.17 (1.24,21.59)*	0.02*		
	Treatment after loss to follow-up	3.95 (1.27,12.28)*	0.02*		
Mundra, 2017 (Maharashtra) *Population: New and previously treated TB patients (sputum smear positive pulmonary, sputum smear negative pulmonary, and extrapulmonary) as a combined population Outcome: Death, treatment failure, treatment modification, and loss to follow-up as a composite outcome* [101]				Values below are adjusted hazard ratios	
	**Age (years)**				
	0-29	Not reported		Ref	
	30-44	Not reported		1.55 (0.82,2.90)	
	45-59	Not reported		2.99 (1.58,5.68)*	
	60 and older	Not reported		2.43 (1.28,4.61)*	
	**Sex**				
	Female	Not reported		Ref	
	Male	Not reported		1.35 (0.82,2.24)	
	**Residence**				
	Rural	Not reported		Ref	
	Urban	Not reported		0.99 (0.64,1.54)	
	**Pulmonary sputum**				
	Positive	Not reported		Ref	
	Negative	Not reported		0.91 (0.56,1.47)	
	Extrapulmonary	Not reported		0.33 (0.15,0.75)*	
	**Category of TB**				
	Category I	Not reported		Ref	
	Category II	Not reported		1.46 (0.91,2.33)	
	**HIV**				
	Negative	Not reported		Ref	
	Positive	Not reported		0.49 (0.15,1.58)	
	Unknown	Not reported		0.97 (0.39,2.43)	

	**Diabetic status**				
	Non-diabetic	Not reported		Ref	
	Diabetic	Not reported		0.35 (0.05,2.56)	
	Unknown	Not reported		1.06 (0.54,1.89)	
Mundra, 2017 (Maharashtra) *Population: New and previously treated TB patients (sputum smear positive pulmonary, sputum smear negative pulmonary, and extrapulmonary) as a combined population Outcome: Treatment failure as a single outcome* [101]				Values below are adjusted hazard ratios	
	**Age (years)**				
	0-29	Not reported		Ref	
	30-44	Not reported		1.59 (0.25,10.05)	
	45-59	Not reported		3.18 (0.35,29.12)	
	60 and older	Not reported		2.98 (0.41,21.82)	
	**Sex**				
	Female	Not reported		Ref	
	Male	Not reported		1.03 (0.17,6.34)	
	**Residence**				
	Rural	Not reported		Ref	
	Urban	Not reported		1.26 (0.31,5.08)	
	**Pulmonary sputum**				
	Positive	Not reported		Ref	
	Negative	Not reported		0	
	Extrapulmonary	Not reported		0	
	**Category of TB**				
	Category I	Not reported		Ref	
	Category II	Not reported		0.40 (0.07,2.24)	
	**HIV**				
	Negative	Not reported		Ref	
	Positive	Not reported		0	
	Unknown	Not reported		0	
	**Diabetic status**				
	Non-diabetic	Not reported		Ref	
	Diabetic	Not reported		0	
	Unknown	Not reported		4.01 (0.67,23.93)	
Mundra, 2017 (Maharashtra) *Population: New and previously treated TB patients (sputum smear positive pulmonary, sputum smear negative pulmonary, and extrapulmonary) as a combined population*				Values below are adjusted hazard ratios	

Outcome: Loss to follow-up as a single outcome [101]					
	**Age (years)**				
	0-29	Not reported		Ref	
	30-44	Not reported		2.21 (0.81,6.05)	
	45-59	Not reported		5.18 (1.95,13.79)*	
	60 and older	Not reported		3.97 (1.47,10.70)*	
	**Sex**				
	Female	Not reported		Ref	
	Male	Not reported		3.03 (1.26,7.31)*	
	**Residence**				
	Rural	Not reported		Ref	
	Urban	Not reported		1.08 (0.58,2.03)	
	**Pulmonary sputum**				
	Positive	Not reported		Ref	
	Negative	Not reported		1.10 (0.58,2.12)	
	Extrapulmonary			0.44 (0.15,1.33)	
	**Category of TB**				
	Category I	Not reported		Ref	
	Category II	Not reported		1.36 (0.70,2.63)	
	**HIV**				
	Negative	Not reported		Ref	
	Positive	Not reported		0.60 (0.14,2.55)	
	Unknown	Not reported		1.78 (0.62,5.10)	
	**Diabetic status**				
	Non-diabetic	Not reported		Ref	
	Diabetic	Not reported		0	
	Unknown	Not reported		1.01 (0.54,1.89)	
Mundra, 2017 (Maharashtra) *Population: New and previously treated TB patients (sputum smear positive pulmonary, sputum smear negative pulmonary, and extrapulmonary) as a combined population* *Outcome: Treatment modification as a single outcome* [101]				Values below are adjusted hazard ratios	
	**Age (years)**				
	0-29	Not reported		Ref	
	30-44	Not reported		0.11 (0.004,3.34)	
	45-59	Not reported		0	
	60 and older	Not reported		0	
	**Sex**				
	Female	Not reported		Ref	

	Male	Not reported		0.06 (0.002,1.92)	
	**Residence**				
	Rural	Not reported		Ref	
	Urban	Not reported		0.62 (0.04,9.54)	
	**Pulmonary sputum**				
	Positive	Not reported		Ref	
	Negative	Not reported		0.81 (0.04,15.54)	
	Extrapulmonary	Not reported		0	
	**Category of TB**				
	Category I	Not reported		Ref	
	Category II	Not reported		23.01 (0.87,609.68)	
	**HIV**				
	Negative	Not reported		Ref	
	Positive	Not reported		0	
	Unknown	Not reported		0	
	**Diabetic status**				
	Non-diabetic	Not reported		Ref	
	Diabetic	Not reported		0	
	Unknown	Not reported		0	
Mundra, 2017 (Maharashtra) *Population: New and previously treated TB patients (sputum smear positive pulmonary, sputum smear negative pulmonary, and extrapulmonary) as a combined population Outcome: Death as a single outcome* [101]				Values below are adjusted hazard ratios	
	**Age (years)**				
	0-29	Not reported		Ref	
	30-44	Not reported		1.21 (0.44,3.30)	
	45-59	Not reported		1.76 (0.59,5.25)	
	60 and older	Not reported		1.74 (0.60,4.99)	
	**Sex**				
	Female	Not reported		Ref	
	Male	Not reported		0.74 (0.34,1.60)	
	**Residence**				
	Rural	Not reported		Ref	
	Urban	Not reported		0.97 (0.46,2.05)	
	**Pulmonary sputum**				
	Positive	Not reported		Ref	
	Negative	Not reported		0.97 (0.43,2.22)	
	Extrapulmonary	Not reported		0.37 (0.10,1.31)	
	**Category of TB**				

	Category I	Not reported		Ref	
	Category II	Not reported		1.63 (0.73,3.66)	
	**HIV**				
	Negative	Not reported		Ref	
	Positive	Not reported		0.51 (0.07,3.95)	
	Unknown	Not reported		0.53 (0.07,3.97)	
	**Diabetic status**				
	Non-diabetic	Not reported		Ref	
	Diabetic	Not reported		1.30 (0.16,10.49)	
	Unknown	Not reported		1.18 (0.53,2.59)	
Mundra, 2018 (Maharashtra) *Population: New and previously treated TB patients (sputum smear positive pulmonary, sputum smear negative pulmonary, and extrapulmonary) as a combined population Outcome: Death, treatment failure, treatment modification, and loss to follow-up as a composite outcome* [102]		Values below are odds ratios		Values below are adjusted odds ratios	
	**Age (years)**				
	0-29	Ref	0.01*		
	30-44	1.63 (0.79,3.37)			
	45-59	3.32 (1.57,7.04)*			
	>=60	2.43 (1.12,5.27)*			
	**Sex**				
	Female	Ref			
	Male	2.16 (1.24,3.77)*	0.007*		
	**Caste**				
	General	Ref	0.91		
	OBC	1.17 (0.58,2.35)			
	SC/ST/NT	1.10 (0.56,2.14)			
	**Residence**				
	Urban	Ref			
	Rural	1.24 (0.75,2.07)	0.4		
	**Education**				
	Graduate or above	Ref	0.01*		
	Less than primary	4.64 (1.70,12.63)*			
	Primary	4.33 (1.57,11.98)*			
	Secondary	3.58 (1.37,9.36)*			
	High School	2.14 (0.84,5.44)			
	**Occupation**				
	Clerical or professional	Ref	0.001*		

	Sem-skilled or skilled labor	3.33 (1.32,8.42)*			
	Unskilled labor	5.00 (1.87,13.36)*			
	Unemployed or students	1.52 (0.60,3.86)			
	**Socioeconomic status**				
	Above the poverty line	Ref			
	Below the poverty line	1.81 (1.07,3.04)*	0.03*		
	**Type of illness**				
	New	Ref			
	Retreatment	2.24 (1.29,3.89)*	0.004*		
	**Site of disease and sputum status**				
	Extrapulmonary TB patients	Ref	0.001*	Ref	
	Pulmonary sputum smear negative TB patients	6.55 (2.45,17.49)*		3.76 (1.18,11.96)*	0.03*
	Pulmonary sputum smear positive TB patients	5.86 (2.11,16.23)*		4.53 (1.36,15.10)*	0.01*
	**Smear conversion at end of the intensive phase**				
	Smear converted	Ref			
	Smear not converted	13.33 (5.16,34.42)*	<0.001*		
	**Experienced side effect of medicines**				
	No	Ref			
	Yes	1.28 (0.64,2.57)	0.48		
	**Comorbidities**				
	Absent	Ref			
	Present	2.90 (1.70,4.93)*	<0.001*		
	**HIV status**				
	Negative	Ref			
	Positive	0.52 (0.14,1.89)			
	Unknown	1.15 (0.37,3.55)			
	**Diabetic status**				
	Non-diabetic	Ref	0.42		
	Diabetic	1.47 (0.37,5.82)			
	Unknown	0.76 (0.45,1.28)			
	**Median delay in days (IQR)[c]**				
	Per each day increase in the period of delay				
	In visiting health facility after developing symptoms	1.00 (0.99,1.01)	0.71		
	In diagnosis from initial health facility visit	0.99 (0.97,1.01)	0.18		
	In treatment initiation after diagnosis	1.02 (0.98,1.07)	0.25		
	**Perception of early or late care-seeking**				

	Early	Ref			
	Late	1.70 (1.01,2.85)*	0.05*		
	**Ever felt discriminated against due to having TB**				
	No	Not reported		Ref	
	Yes	Not reported		2.20 (1.08,4.51)*	0.03*
	**Satisfaction with services at diagnostic facility/DOTS center**				
	Totally satisfied	Not reported		Ref	
	Good	Not reported		4.08 (1.39,11.97)*	0.01*
	Average or less	Not reported		3.18 (1.06,9.51)*	0.04*
	**Addiction**				
	None	Ref			
	Any addiction	3.00 (1.73,5.20)*	<0.001*		
	Smokeless tobacco	2.14 (1.27,3.60)*	0.004*		
	Smoking	2.27 (1.13,4.59)*	0.02*		
	Ever smoked	3.79 (2.17,6.61)*	<0.001*		
	Alcohol	3.71 (2.11,6.53)*	<0.001*		
	**Missing any dose during treatment**				
	No	Ref			
	Yes	1.16 (0.69,1.95)			
	**Felt cured and the need to stop medicines during treatment**				
	No	Ref			
	Yes	2.75 (1.35,5.61)*	0.005*		
	**Family type**				
	Joint	Ref			
	Nuclear	1.06 (0.60,1.88)	0.84		
	**Family problem**				
	No	Ref			
	Yes	3.04 (1.66,5.59)*	<0.001*		
	**Family support**				
	Yes	Ref			
	No	7.22 (2.74,19.06)*	<0.001*		
	**Missing work or education during treatment**				
	No	Ref			
	Yes	1.17 (0.70,1.94)			
	**STS ever visited patient**				
	No	Ref			
	Yes	0.53 (0.32,0.89)*	0.02*		

	**Indoor air pollution**				
	Absent	Ref		Ref	
	Present	5.37 (2.80,10.30)*	<0.001*	4.06 (1.67,9.89)*	0.002*
	**Median travel cost in INR (IQR)[c]**				
	Per each INR increase in cost	Ref			
	Cost of travelling to diagnostic center	1.00 (0.99,1.01)	0.37		
	Cost of travelling to DOTS center	1.00 (0.99,1.02)	0.87		
	**Median distance of health facilities from resident in km (IQR)[c]**				
	Per each km increase in distance				
	Nearest government health facility	0.97 (0.90,1.05)	0.48		
	Distance of diagnostic facility	0.99 (0.97,1.02)	0.58		
	Distance of DOTS center	1.00 (0.97,1.03)	0.8		
	**Type of DOTS provider**				
	Public health facility based	Ref	0.35		
	ASHA/community center	1.03 (0.61,1.75)			
	Others	2.26 (0.74,6.92)			
	**Behavior of DOTS provider**				
	Very good	Ref	0.03		
	Good	3.39 (0.97,11.87)			
	Average	4.12 (1.14,14.83)*			
	Bad	14.67 (1.83,117.68)*			
	**Residence of service provider at DMC**				
	Same village/ward	Ref			
	Different village/ward	1.19 (0.45,3.20)	0.73		
	**Counselling before treatment initiation**				
	Yes	Ref			
	No	1.15 (0.66,1.99)	0.62		
	**Residence of regular DOTS provider**				
	Same village/ward	Ref			
	Different village/ward	0.87 (0.51,1.47)	0.59		
	**DOTS provider visited home for giving medicines**				
	Yes	Ref			
	No	1.25 (0.75,2.08)	0.4		
Nahar, 2014 (Madhya Pradesh) *Population: TB patients being treated in the*		Values below are odds ratios			

government TB program (no further description, but presumed to comprise new and previously treated patients) *Outcome: Loss to follow-up as a single outcome* [103]					
	**Living situation**				
	Stable living situation	Ref			
	Frequent change of residence or homelessness	2.30 (2.10,2.50)*	0.03*		
	**Alcoholism**				
	No	Ref			
	Yes	1.76 (1.40,2.12)*	0.04*		
	**Awareness of exact duration of treatment**				
	Aware	Ref			
	Not aware	1.72 (1.40,2.04)*	0.04*		
	**Awareness of consequences of cessation of treatment**				
	Aware	Ref			
	Not aware	1.77 (1.53,2.04)*	0.04*		
	**Uncertainty about treatment success**				
	Certain	Ref			
	Uncertain	1.50 (1.24,1.76)*	0.05*		
Nandakumar, 2013 (Kerala) *Population: New and previously treated TB patients (sputum smear positive pulmonary, sputum smear negative pulmonary, and extrapulmonary) as a combined population* *Outcome: Death, treatment failure, loss to follow-up, and transferred out as a composite outcome* [104]		Values below are relative risk ratios		Values below are adjusted relative risk ratios	
	**Sex**				
	Female	Ref		Ref	
	Male	1.94 (1.57,2.40)*		1.60 (1.28,1.99)*	
	**Age (years)**				
	15-44	Ref		Ref	
	>45	2.06 (1.71,2.50)*		1.72 (1.40,2.10)*	
	**Site**				
	Extrapulmonary	Ref		Ref	
	Pulmonary	1.72 (1.38,2.14)*		1.3 (0.99,1.72)	
	**Type of case**				
	New	Ref		Ref	

	Previously treated	1.64 (1.34,2.01)*		1.43 (1.18,1.75)*	
	**Sputum smear status**				
	Negative/unknown smear	Ref		Ref	
	Positive smear	1.41 (1.19,1.68)*		1.02 (0.86,1.28)	
	**HIV status**				
	Negative	Ref		Ref	
	Positive	1.64 (0.88,3.04)		1.93 (1.06,3.5)*	
	Unknown	1.65 (1.37,1.99)*		1.51 (1.23,1.84)*	
	**Diabetes**				
	No	Ref		Ref	
	Yes	1.25 (1.02,1.53)*		0.99 (0.81,1.21)	
	Unknown	1.67 (1.33,2.10)*		1.34 (1.05,1.70)*	
	**Diabetic control**				
	Yes	Ref			
	No	2 (0.97,4.13)			
	Unknown	2.14 (1.11,4.13)*			
	**DOT in IP**				
	Regular	Ref			
	Missed doses	2.85 (2.32,3.49)*			
Nandi, 2022 (West Bengal) *Population: New and previously treated sputum smear positive pulmonary TB patients as a combined population* *Outcome: Death, treatment failure, and loss to follow-up as a composite outcome* [105]		Values below are odds ratios			
	**Age (years)**				
	10-19	Ref			
	20-39	2.53 (0.14,45.61)	0.53		
	40-59	1.34 (0.07,26.28)	0.84		
	≥60	1.67 (0.08,36.64)	0.75		
	**Sex**				
	Female	Ref			
	Male	0.62 (0.20,1.87)	0.39		
	**Religion**				
	Hindu	Ref			
	Muslim	0.27 (0.04,2.12)	0.21		
	**Category of treatment**				
	Cat I	Ref			
	Cat II	1.72 (0.37,8.09)	0.49		
	**Treatment interruption**				
	Uninterrupted	Ref			

	Interrupted	28.05 (8.32,94.62)*	<0.0001*		
	**Duration of interruption**				
	<1 week	Ref			
	1-2 weeks	0.85 (0.10,7.04)	0.87		
	≥2 weeks	38.50 (2.92,508.49)*	0.006*		
Panati, 2023 (Andhra Pradhesh) *Population: New and previously treated TB patients as a combined population* *Outcome: Low to medium adherence as a single outcome* [106]		Values below are odds ratios		Values below are adjusted odds ratios	
	**Age (years)**				
	>70	Ref		Ref	
	≤70	0.94 (0.4,2.1)		0.85 (0.2,3.0)	
	**Sex**				
	Female	Ref		Ref	
	Male	0.66 (0.3,1.5)		0.63 (0.2,2.2)	
	**Marital status**				
	Not with partner	Ref		Ref	
	With partner	1.23 (0.5,2.8)		1.26 (0.4,4.4)	
	**Educational status**				
	Illiterate	Ref		Ref	
	Primary school	0.39 (0.1,1.7)		0.41 (0.0,25.0)	
	Secondary	1.22 (0.2,6.2)		0.32 (0.0,56.7)	
	High School	0.33 (0.1,1.4)		0.35 (0.0,102)	
	College	0.39 (0.1,1.3)		1.62 (0.0,501)	
	**Occupation**				
	Unemployed	Ref		Ref	
	Unskilled	0.55 (0.1,3.0)		1.48 (0.1,38.7)	
	Semi-skilled	1.02 (0.2,4.7)		3.05 (0.0,542)	
	Skilled	0.60 (0.2,2.3)		1.99 (0.0,649)	
	Semi-professional	0.26 (0.1,1.1)		0.65 (0.0,298)	
	Professional	0.36 (0.1,2.0)		0.64 (0.0,325)	
	**Locality**				
	Rural	Ref		Ref	
	Urban	1.33 (0.6,3.0)		2.29 (0.4,12.5)	
	**Previous history of TB infection**				
	No	Ref		Ref	
	Yes	0.66 (0.2,1.8)		0.60 (0.1,2.9)	
	**Disease status at the end of Intensive phase**				
	Smear negative	Ref		Ref	

	Smear positive	1.16 (0.5,2.7)		1.98 (0.1,9.8)	
	**Presence of depression**				
	No	Ref		Ref	
	Yes	30.9 (9.9,97)*		43.6 (9.5,199)*	
Pardeshi, 2007[a] (Maharashtra) *Population: New and previously treated sputum smear positive pulmonary TB patients as a combined population* *Outcome: Death,treatment failure, and loss to follow-up as a composite outcome* [107]		Values below are odds ratios			
	**TB classification**				
	New smear positive	Ref			
	Retreatment	2.71 (2.12,3.45)*	<0.0001*		
Pardeshi, 2010[a] (Maharashtra) *Population: New and previously treated sputum smear positive pulmonary TB patients as a combined population* *Outcome: Death, treatment failure, loss to follow-up, and transferred out as a composite outcome* [44]		Values below are odds ratios			
	**Follow-up sputum status at the end of intensive phase**				
	Negative sputum smear (i.e., sputum conversion)	Ref			
	Positive sputum smear (i.e., nonconversion of sputum)	70.29 (26.48,186.62)*	<0.0001*		
Patra, 2013 (Delhi) *Population: New and previously treated TB patients (sputum smear positive pulmonary, sputum smear negative pulmonary, and extrapulmonary) as a combined population among individuals >=60 years old* *Outcome: Death, treatment failure, loss to follow-up, and transferred out as a composite outcome* [108]		Values below are odds ratios		Values below are adjusted odds ratios	
	**Age (years)**				
	60-64	Ref			
	65-74	1.2 (0.8,1.7)	Not reported		
	75 and older	1.4 (0.8,2.7)	Not reported		
	**Sex**				

	Female	Ref		Ref	
	Male	1.8 (1.2,2.5)*	Not reported	1.6 (1.1,2.2)*	0.02*
	**TB classification**				
	Extrapulmonary TB patients	Ref		Ref	
	Smear-positive pulmonary TB patients	2.6 (1.6,4.3)*	Not reported	2.2 (1.3,3.8)*	0.002*
	Smear-negative pulmonary TB patients	1.7 (0.9,3.0)	Not reported	1.5 (0.8,2.6)	
	Unknown TB classification	1.8 (0.5,7.2)	Not reported	1.8 (0.4,7.0)	
	**TB patient type**				
	New	Ref			
	Retreatment	1.5 (1.0,2.1)	Not reported		
Paunikar, 2019 (Maharashtra) *Population: New and previously treated TB patients (sputum smear positive pulmonary, sputum smear negative pulmonary, and extrapulmonary) as a combined population Outcome: Loss to follow-up as a single outcome* [109]		Values below are hazard ratios			
	**Sex**				
	Female	Ref			
	Male	9.09 (1.27,60.30)*	0.04*		
	**HIV status**				
	Negative	Ref			
	Positive	4.00 (1.33,12.00)	0.19		
	**Smoking**				
	Non-smoker	Ref			
	Person who smokes	3.70 (1.23,11.11)*	0.02*		
	**Alcohol**				
	Non-drinker	Ref			
	Person who drinks alcohol	1.67 (0.56,4.99)	0.36		
Pore, 2020[a] (Maharashtra) *Population: Presumed drug-susceptible new and previously treated TB patients and multidrug-resistant TB patients (sputum smear positive pulmonary, sputum smear negative pulmonary, and extrapulmonary) as a combined population Outcome: Treatment non-adherence as a single outcome* [110]		Values below are odds ratios			
	**Age group (years)**				

	21-30	Ref			
	18-20	3.41 (0.50,23.36)	0.21		
	31-40	1.82 (0.56,5.85)	0.32		
	41-50	1.95 (0.53,7.15)	0.32		
	51-60	0.85 (0.19,3.84)	0.84		
	61-70	3.41 (0.50,23.36)	0.21		
	**Sex**				
	Female	Ref			
	Male	1.63 (0.56,4.77)	0.37		
	**Education**				
	Graduate and above	Ref			
	Illiterate	1.75 (0.47,6.45)	0.4		
	School not completed	0.87 (0.26,2.97)	0.83		
	High school completed	0.42 (0.10,1.68)	0.22		
	**Occupation**				
	Private job	Ref			
	Farmer	0.96 (0.21,4.34)	0.96		
	Housewife	0.89 (0.19,4.24)	0.88		
	Laborer	1.60 (0.38,6.82)	0.53		
	Self-employed	0.44 (0.08,2.38)	0.34		
	Service	0.80 (0.13,4.75)	0.81		
	Others	4.80 (0.38,59.90)	0.22		
	**Treatment category**				
	Category I (new TB patients)	Ref			
	Category II (previously treated TB patients)	52.00 (10.64,254.23)*	<0.0001*		
	Multidrug-resistant TB patients	13.70 (0.52,358.07)	0.12		
	**Disclosure to family about disease**				
	Yes	Ref			
	No	0.61 (0.11,3.35)	0.57		
Potty, 2021[b] (Karnataka and Telangana) *Population: New and previously treated TB patients (sputum smear positive pulmonary, sputum smear negative pulmonary, and extrapulmonary) as a combined population* *Outcome: Death, treatment failure, loss to follow-up, and transferred out as a composite outcome* [111]		Values below are odds ratios (Confidence Intervals not provided)		Values below are adjusted odds ratios	
	**Name of District**				
	Bengaluru	Ref		Ref	
	Hyderabad	0.21	<0.001*	0.16 (0.10,0.24)*	<0.001*

	**Age (years)**				
	<15	Ref			
	15-49	2.94	0.04*		
	50+	5.00	0.002*		
	**Sex**				
	Female	Ref		Ref	
	Male	2.86	<0.001*	2.33 (1.64,3.29)*	<0.001*
	**Marital status**				
	Currently married	Ref		Ref	
	Marriage dissolved	1.11	0.67	1.12 (0.62,2.04)	0.69
	Single (Never married)	0.48	<0.001*	0.63 (0.42,0.95)*	0.03*
	**HIV status**				
	Negative	Ref		Ref	
	Positive	3.03	0.01*	3.33 (1.12,9.89)*	0.03*
	Unknown	0.47	<0.001*	0.83 (0.52,1.30)	0.42
	**Type of TB**				
	Extra Pulmonary TB	Ref		Ref	
	Pulmonary TB	2.33	<0.001*	1.72 (1.04,2.86)*	0.03*
	**History of previous TB treatment**				
	No	Ref		Ref	
	Yes	2.04	<0.001*	1.72 (1.08,2.74)*	0.02*
	**Initial weight**				
	Below median value	Ref			
	Median value or above	0.63	0.03*		
	Unknown	0.75	0.12		
	**Patient's relationship with care supporter**				
	No care supporter	Ref		Ref	
	Parent	1.30	0.21	0.68 (0.40,1.14)	0.14
	Siblings/Son/Daughter	2.22	<0.001*	1.06 (0.62,1.82)	0.83
	Spouse	1.56	0.03*	0.67 (0.40,1.12)	0.13
	Others	0.97	0.928	0.57 (0.31,1.08)	0.08
	**Number of visits during CP**				
	<2	Ref			
	2-3	0.13	<0.001*		
	4+	0.06	<0.001*		
	**Number of visits during IP**				
	<2	Ref			
	2-3	0.39	<0.001*		
	4+	0.50	<0.001*		

	**Number of follow-up visits by community healthcare workers**				
	<4	Ref		Ref	
	4-7	0.16	<0.001*	0.15 (0.11,0.23)*	<0.001*
	8+	0.09	<0.001*	0.04 (0.03,0.07)*	<0.001*
	**Missed any doses**				
	No	Ref		Ref	
	Yes	2.44	<0.001*	4.00 (2.63,6.08)*	<0.001*
	**Provided TB awareness counseling**				
	No	Ref			
	Yes	0.36	<0.001*		
	**Provided adherence counseling**				
	No	Ref		Ref	
	Yes	0.30	<0.001*	0.62 (0.38,1.01)	0.06
	**Provided nutritional counseling/support**				
	No	Ref			
	Yes	0.41	<0.001*		
	**Provided family level counseling**				
	No	Ref			
	Yes	0.53	0.03*		
Potty, 2023 (Karnataka and Telangana) *Population: New and previously treated TB patients (sputum smear positive pulmonary, sputum smear negative pulmonary, and extrapulmonary) as a combined population* *Outcome: Death, treatment failure, modification of therapy, and loss to follow-up, as a composite outcome* [112]				Values below are adjusted odds ratios	
	**Participation in support group meetings**				
	Participation			Ref	
	Non-participation			2.44 (2.10,2.82)*	
Prudhivi, 2019[b] (Andhra Pradesh) *Population: New and previously treated pulmonary TB patients (sputum smear positive and sputum smear negative) as a combined population* *Outcome: Death, treatment failure, loss to follow-up, and*		Values below are odds ratios		Values below are adjusted odds ratios	

transferred out as a composite outcome [113]					
	**Age (years)**				
	<=50	Ref		Ref	
	>50	1.45 (1.06,1.98)*	<0.001*	2.13 (0.50,8.98)	
	**Sex**[f]				
	Female	Ref		Ref	
	Male	1.84 (1.28,2.64)*	<0.001*	1.96 (1.4,2.52)*	<0.001*
	**Residence**				
	Urban	Ref		Ref	
	Rural	1.41 (0.93,2.14)		1.52 (0.96,2.38)	
	**Type of pulmonary TB**[f]				
	Sputum smear negative	Ref		Ref	
	Sputum smear positive	0.28 (0.21,0.39)*	<0.001*	0.35 (0.28,0.42)*	<0.001*
	**TB category**				
	New TB patients	Ref		Ref	
	Previously treated TB patients	3.57 (2.56,4.99)*	<0.001*	2.94 (2.31,3.74)*	<0.001*
	**HIV status**[f]				
	Negative	Ref		Ref	
	Positive	2.87 (1.97,4.17)*	<0.001*	3.01 (2.11,3.91)*	<0.001*
	**Smoking**				
	No	Ref		Ref	
	Yes	1.5 (1.12,2.12)*	<0.001*	1.7 (1.32,2.08)*	<0.001*
	**Alcohol**				
	No	Ref		Ref	
	Yes	1.47 (1.07,2.03)*	<0.001*	1.39 (0.99,1.79)*	<0.001*
Ratnesh, 2020 (Uttar Pradesh) *Population: New and previously treated TB patients as a combined population, without further description of the population Outcome: Loss to follow-up as a single outcome* [114]		Values below are odds ratios			
	**History of treatment interruption**				
	No	Ref			
	Yes	7.42 (5.34,10.31)*	0.001*		
Saini, 2016[a] (Chandigarh) *Population: New and previously treated TB patients (sputum smear positive pulmonary, sputum smear negative pulmonary, and extrapulmonary) as a combined population Outcome: Death, treatment failure, loss to follow-up, and*		Values below are odds ratios			

treatment regimen changed to Cat IV as a composite outcome [115]					
	**HIV status**				
	Negative	Ref			
	Positive	0.81 (0.35,1.87)	0.62		
	Unknown	2.67 (1.16,6.15)*	0.02*		
Shabil, 2019[a] (Karnataka) *Population: New and previously treated TB patients as a combined population, without further description of the population Outcome: Loss to follow-up as a single outcome* [116]		Values below are odds ratios			
	**Age (years)**				
	18-29	Ref			
	30-49	1.74 (0.17,18.02)	0.64		
	50-59	2.77 (0.23,33.88)	0.43		
	60-69	9.00 (0.92,88.17)	0.06		
	>=70	3.00 (0.16,55.72)	0.46		
	**Sex**				
	Female	Ref			
	Male	1.80 (0.50,6.49)	0.37		
	**Residence**				
	Rural	Ref			
	Urban	0.80 (0.22,2.89)	0.73		
	**Education**				
	Primary Education	Ref			
	Illiterate	0.44 (0.08,2.38)	0.34		
	Secondary Education	0.46 (0.11,1.98)	0.3		
	Graduate	0.29 (0.01,5.79)	0.42		
	**Association of interruptions with treatment phase**				
	Late continuation phase	Ref			
	Intensive phase	7.86 (0.28,217.12)	0.22		
	Early continuation phase	8.33 (0.32,215.69)	0.2		
	**Smoker**				
	No	Ref			
	Yes	0.26 (0.01,4.74)	0.36		
	**Alcohol use**				
	No	Ref			
	Yes	8.33 (1.46,47.64)*	0.02*		
	**Both smoking and alcohol**				
	No	Ref			

	Yes	1.09 (0.12,9.95)	0.94		
	**Family support**				
	Yes	Ref			
	No	0.50 (1.15,1.70)	0.27		
	**Distance**				
	0-2 km	Ref			
	2-5 km	5.33 (0.56,50.82)	0.15		
	5-10 km	9.41 (1.02,87.20)*	0.05*		
	>10 km	12.80 (0.97,168.74)	0.05		
	**Problems faced in getting medicine**				
	None	Ref			
	Transport	2.25 (0.45,11.31)	0.32		
	Staying alone	2.63 (0.41,16.83)	0.31		
	Time	0.56 (0.03,11.31)	0.7		
	Workload	5.25 (0.94,29.44)	0.06		
	**Availability of medicines**				
	Always	Ref			
	Sometimes	4.00 (0.85,18.84)	0.08		
	**Satisfaction with DOTS providers attitudes**				
	Satisfied	Ref			
	Dissatisfied	2.56 (0.72,9.10)	0.15		
Sharma, 2003[a] (Delhi) *Population: New and previously treated TB patients as a combined population, without further description of the population Outcome: Treatment failure, LTFU, transferred out and death as a composite outcome* [117]		Values below are odds ratios			
	**Classification**				
	Extrapulmonary	Ref			
	New sputum smear positive pulmonary TB patients	8.32 (0.43,162.0)	0.16		
	New sputum smear negative sputum pulmonary TB patients	2.16 (0.08,56.71)	0.64		
	Previously treated TB patients	22.14 (0.86,571.32)	0.06		
Sharma, 2021[a] (West Bengal) *Population: New and previously treated TB patients (sputum smear positive pulmonary, sputum smear negative pulmonary, and extrapulmonary) as a combined population*		Values below are relative risk ratios		Values below are adjusted relative risk ratios	

Outcome: Death, treatment failure, treatment modified to drug-resistant TB therapy, loss to follow-up, and not evaluated as a composite outcome [118]					
	**Age (years)**				
	15-24	Ref		Ref	
	0-14	1.5 (0.9,2.7)	Not reported	1.7 (1.0,2.7)*	0.04*
	25-34	1.2 (0.9,1.8)	Not reported	1.6 (1.1,2.1)*	0.008*
	35-44	1.4 (1.0,2.0)*	Not reported	2.2 (1.5,3.2)*	<0.001*
	45-54	1.4 (1.0,2.0)*	Not reported	1.7 (1.2,2.5)*	0.009*
	55-64	1.1 (0.7,1.7)	Not reported	1.4 (0.9,2.2)	0.13
	65 and older	1.5 (1.0,2.3)*	Not reported	2.1 (1.3,3.2)*	0.002*
	**Sex**				
	Female	Ref			
	Male	1.0 (0.8,1.3)	Not reported		
	**Type of TB**				
	Clinically-diagnosed	Ref		Ref	
	Microbiologically confirmed	1.3 (1.0,1.6)*	Not reported	1.2 (0.9,8.5)	0.16
	**Site of TB**				
	Pulmonary	Ref			
	Extrapulmonary	1.1 (0.8,1.4)	Not reported		
	**Category of TB**				
	New	Ref		Ref	
	Previously treated	1.4 (1.0,2.1)*	Not reported	1.1 (0.8,1.6)	0.61
	**Source of TB medications**				
	Private pharmacy	Ref		Ref	
	National TB Program (free)	3.6 (3.0,4.3)*	Not reported	4.0 (3.1,5.0)*	<0.001*
	**HIV status**				
	Negative/Unknown	Ref			
	Positive	1.8 (0.3,9.8)	Not reported		
	**Diabetic status**				
	No/Unknown	Ref			
	Diabetic	1.1 (0.8,1.5)	Not reported		
	**District**				
	East Medinipur	Ref		Ref	
	Kolkota	4.8 (1.6,14.6)*	Not reported	2.8 (0.9,8.5)	0.07

	Howrah	3.7 (1.2,11.5)*	Not reported	2.5 (0.8,8.3)	0.13
	Hooghly	3.0 (0.9,9.8)	Not reported	2.0 (0.6,6.6)	0.25
	North 24 Parganas	3.4 (1.1,10.8)*	Not reported	2.3 (0.7,7.5)	0.16
	South 24 Parganas	4.6 (1.8,17.6)*	Not reported	3.3 (1.1,10.3)*	0.04*
	**TB care in the district of residence**				
	Same district	Ref			
	Different district	1.1 (0.8,1.4)	Not reported		
	**Type of health facility**				
	Clinic	Ref			
	Hospital/Nursing home	1.5 (0.8,3.0)	Not reported		
	**Education of provider**				
	MBBS	1.0 (0.8,1.3)	Not reported		
Shivam, 2014[a] (West Bengal) *Population: New and previously treated TB patients (sputum smear positive pulmonary, sputum smear negative pulmonary, and extrapulmonary) as a combined population* *Outcome: Death, treatment failure, and loss to follow-up as a composite outcome* [23]		Values below are odds ratios			
	**Treatment category**				
	Category I (new)	Ref			
	Category II (previously treated)	2.10 (1.40,3.16)*	0.0004*		
Siddiqui, 2016 (Delhi) *Population: New and previously treated TB patients (sputum smear positive pulmonary, sputum smear negative pulmonary, and extrapulmonary) as a combined population* *Outcome: Death, treatment failure, treatment modified to drug-resistant TB therapy, and loss to follow-up as a composite outcome* [119]				Values below are adjusted odds ratios	
	**Sex**				
	Female			Ref	
	Male			0.31 (0.06,1.76)	
	**Age (years)[c]**				
	Per each year increase in age			0.96 (0.90,1.02)	
	**Category of TB**				

	Cat I			0.84 (0.24,2.89)	
	Cat II			Ref	
	**TB history**				
	No			Ref	
	Yes			2.59 (0.26,25.82)	
	**ADR incidence**				
	No			Ref	
	Yes			0.64 (0.19,2.21)	
	**Fever**				
	No			Ref	
	Yes			0.81 (0.09,2.16)	
	**Dyspnea**				
	No			Ref	
	Yes			1.97 (0.34,5.87)	
	**Chest pain**				
	No			Ref	
	Yes			1.37 (0.49,6.14)	
	**Hemoptysis**				
	No			Ref	
	Yes			0.81 (0.29,2.28)	
	**Diabetes**				
	No			Ref	
	Yes			0.71 (0.16,3.28)	
	**BMI**[c]				
	Per each kg/m^2 increase in age			1.19 (0.97,1.45)	
	**Weight gain**				
	No			Ref	
	Yes			0.71 (0.06,8.52)	
	**Anorexia**				
	No			Ref	
	Yes			0.56 (0.05,3.12)	
	**Alcohol intake**				
	No			Ref	
	Yes			0.67 (0.17,2.56)	
	**Smoking**				
	No			Ref	
	Yes			0.75 (0.24,2.39)	
	**Chewing tobacco**				
	No			Ref	
	Yes			0.78 (0.24,2.54)	
Singh, 2020 (Uttarakhand) *Population: New and previously treated TB*		Values below are relative risk ratios		Values below are adjusted relative risk ratios	

patients (sputum smear positive pulmonary, sputum smear negative pulmonary, and extrapulmonary) as a combined population Outcome: Death, treatment failure, treatment regimen modified, loss to follow-up, transferred out and not evaluated as a composite outcome [120]					
	**Sex**				
	Female	Ref			
	Male	1.5 (0.8,2.7)	0.19	1.3 (0.7,2.2)	0.4
	**Age (years)**				
	0-14	Ref			
	15-44	0.9 (0.2,5.5)	0.9		
	45 and older	1.5 (0.3,9.0)	0.8		
	**Category of patient**				
	Category I (new)	Ref		Ref	
	Category II (previously treated)	2.0 (1.2,3.6)*	0.02*	3.2 (2.1,4.9)*	0.0001*
	**Site of TB**				
	Extrapulmonary	Ref		Ref	
	Pulmonary	9.3 (1.3,35.5)*	0.003*	5.6 (0.8,39.8)	0.08
	**Bacteriological status**				
	Clinically confirmed	Ref			
	Bacteriologically confirmed	2.3 (1.2,4.5)*	0.008*		
	**Type of regimen**				
	Daily	Ref			
	Intermittent	1.2 (0.7,2.2)	0.4		
	**Treatment delays (days)**				
	7 or fewer	Ref		Ref	
	More than 7	1.7 (0.8,3.9)	0.2	1.5 (0.8,2.0)	0.2
	**Type of case finding**				
	Passive case finding	Ref		Ref	
	Active case finding	2.5 (1.5,4.2)*	<0.001*	2.6 (1.7,4.0)*	<0.001*
Sodhi, 2023 (Gujarat, Madhya Pradesh, Bihar, New Delhi, and Haryana) *Population: New and previously treated TB patients (sputum smear positive pulmonary, sputum smear negative pulmonary, and extrapulmonary) as a combined population Outcome: Death, treatment failure, and loss to follow-up*				Values below are adjusted odds ratios	

as a composite outcome [121]					
	**Received free drugs (Model E)**				
	Yes			Ref	
	No			1.45 (1.29,1.64)*[g]	<0.01
Subbaraman, 2021 (Tamil Nadu and Maharashtra) *Population: New and previously treated TB patients (sputum smear positive pulmonary, sputum smear negative pulmonary, and extrapulmonary) as a combined population* *Outcome: Medication non-adherence as a single outcome* [122]		Values below are odds ratios		Values below are adjusted odds ratios	
	**Sex**				
	Female	Ref		Ref	
	Male	1.3 (0.8,2.1)	0.31	1.0 (0.5,1.8)	0.92
	**Age**				
	18-29	Ref		Ref	
	30-44	2.1 (1.2,3.8)*	0.01*	1.1 (0.6,2.2)	0.71
	≥45	1.2 (0.6,2.4)	0.62	0.6 (0.3,1.2)	0.15
	**Monthly Income**				
	INR <7500	Ref		Ref	
	INR 7500-14,999	0.9 (0.5,1.5)	0.74	1.4 (0.8,2.7)	0.24
	INR ≥15,000	0.5 (0.3,1.1)	0.08	0.9 (0.4,2.0)	0.74
	**Occupation**				
	Self-employed	Ref		Ref	
	Employed in government or private sector	1.5 (0.7,3.2)	0.35	1.7 (0.7,3.9)	0.23
	Laborer on daily wages	2.5 (1.1,5.5)*	0.03*	2.7 (1.1,6.5)*	0.03*
	Housewife, student, or unemployed	1.3 (0.6,2.6)	0.47	1.6 (0.7,3.6)	0.26
	**Phase of therapy**				
	Intensive phase	Ref		Ref	
	Early continuation phase	0.7 (0.4,1.4)	0.33	1.1 (0.5,2.1)	0.87
	Late continuation phase	1.5 (0.8,2.7)	0.16	2.0 (1.1,3.9)*	0.03*
	**Category of TB**				
	New	Ref		Ref	
	Previously treated	1.4 (0.9,2.5)	0.17	1.4 (0.8,2.5)	0.24
	**Type of TB**				
	Extrapulmonary	Ref		Ref	
	Smear-negative pulmonary	2.1 (0.9,4.8)	0.08	1.9 (0.8,4.7)	0.15
	Smear-positive pulmonary	2.0 (1.1,3.6)*	0.03*	2.1 (1.1,3.9)*	0.03*
	**People with HIV**				

	No	Ref		Ref	
	Yes	2.5 (1.5,4.1)	.0003*	1.5 (0.6,3.6)	0.43
	**Transport Mode to Clinic**				
	Walking or bicycle	Ref			
	Motorcycle or car	2.2 (0.7,6.5)	0.16		
	Autorickshaw or taxi	2.0 (0.9,4.5)	0.08		
	Public transportation	3.9 (2.0,7.4)*	<0.0001*		
	**Money Spent to Collect Medication Refills**				
	INR 0-24	Ref			
	INR 25-49	1.3 (0.6,2.8)	0.58		
	INR 50-75	2.3 (1.1,4.7)*	0.02*		
	INR >75	2.6 (1.4,4.8)*	0.003*		
	**Time Spent to Collect Medication Refills**				
	<30 minutes	Ref		Ref	
	30-59 minutes	6.0 (1.4,26.1)*	0.02*	6.6 (1.5,29.5)*	0.01*
	≥60 minutes	10.3 (2.5,43.0)*	0.001*	9.0 (1.8,44.2)*	0.007*
	**Current Tobacco Use**				
	No	Ref			
	Smokeless tobacco only	0.7 (0.2,2.0)	0.5		
	Cigarette or beedi use	2.5 (1.3,4.9)*	0.006*		
	**Probable Alcohol Use**				
	No alcohol use	Ref		Ref	
	Any alcohol use	3.2 (1.7,6.1)*	0.0003*	2.5 (1.2,5.2)*	0.01*
Vasantha, 2008 (Tamil Nadu) *Population: New and previously treated TB patients (sputum smear positive pulmonary, sputum smear negative pulmonary, and extrapulmonary) as a combined population* *Outcome: Death as a single outcome* [123]				Values below are adjusted hazard ratios	
	**Sex**				
	Female			Ref	
	Male			1.32 (0.73,2.39)	0.36
	**Age (years)**				
	<45			Ref	
	>=45			2.35 (1.56,3.55)*	<0.001*
	**Occupation**				
	Employed			Ref	
	Unemployed			1.38 (0.92,2.06)	0.12
	**Education**				
	Literate			Ref	

	Illiterate			1.28 (0.87,1.88)	0.22
	**Treatment category**				
	Category III			Ref	
	Category I			1.19 (0.76,1.86)	Not reported
	Category II			0.77 (0.39,1.51)	Not reported
	**Previous treatment**				
	New patient			Ref	
	Retreatment (previously treated)			1.62 (1.10,2.37)*	<0.05*
	**Bodyweight**				
	>=35 kg			Ref	
	<35 kg			3.71 (2.43,5.65)*	<0.001*
	**Alcoholism**				
	No alcoholism			Ref	
	Alcoholism			2.02 (1.36,2.99)*	<0.005*
	**Smoking**				
	No smoking			Ref	
	Smoking			0.73 (0.44,1.23)	0.24
	**Type of DOT provider**				
	Friends, relatives, self, and others			Ref	
	Government DOT			1.31 (0.72,2.38)	0.38
	Community DOT			0.99 (0.54,1.81)	0.97
	**Supervision under IP**				
	Always			Ref	
	Never			1.17 (0.77,1.78)	0.47
Vashishtha, 2013[a] (Delhi) *Population: New and previously treated TB patients (sputum smear positive pulmonary, sputum smear negative pulmonary, and extrapulmonary) as a combined population among people with and without HIV Outcome: Death, treatment failure, treatment regimen modified, and loss to follow-up as a composite outcome* [30]		Values below are odds ratios			
	**HIV status**				
	HIV-negative	Ref			
	HIV-positive	4.25 (2.02,8.96)*	0.0001*		
Vasudevan, 2014[a] (Puducherry) *Population: New and previously treated TB patients (sputum smear positive pulmonary, sputum*		Values below are odds ratios			

smear negative pulmonary, and extrapulmonary) as a combined population *Outcome: Loss to follow-up as a single outcome* [124]					
	**Treatment category**				
	Category I (new)	Ref			
	Category II (previously treated)	2.03 (1.13,3.65)*	0.02*		
Viswanathan, 2014[a] (Tamil Nadu) *Population: New and previously treated TB patients (sputum smear positive pulmonary, sputum smear negative pulmonary, and extrapulmonary) as a combined population* *Outcome: Death, treatment failure, and loss to follow-up as a composite outcome* [34]		Values below are odds ratios			
	**Diabetic status**				
	Non-diabetic	Ref			
	Diabetic	2.59 (0.84,8.01)	0.1		
Washington, 2020 (Karnataka and Telangana) *Population: Presumed drug-susceptible new and previously treated TB patients and multidrug-resistant TB patients (sputum smear positive pulmonary, sputum smear negative pulmonary, and extrapulmonary) as a combined population* *Outcome: Death, treatment failure, and loss to follow-up as a composite outcome* [125]				Values below are adjusted odds ratios	
	**Sex**				
	Female			Ref	
	Male			1.69 (1.24,2.30)*	<0.001*
	**Age (years)**				
	Below 60			Ref	
	60 and above			1.12 (0.79,1.60)	0.53
	**State**				
	Telangana			Ref	
	Karnataka			2.46 (1.79,3.39)*	<0.001*
	**Religion**				
	Muslim			Ref	
	Hindu			1.10 (0.82,1.47)	0.54

	Other religion			1.40 (0.74,2.6)	0.3
	**Marital status**				
	Single			Ref	
	Married			0.95 (0.68,1.34)	0.78
	Marriage dissolved			1.22 (0.66,2.24)	0.52
	Marriage not known			3.23 (0.70,14.96)	0.13
	**Living alone**				
	No			Ref	
	Yes			0.40 (0.38,1.42)	0.47
	**Education**				
	10th standard or more			Ref	
	Less than 5th standard			2.74 (1.71,4.41)*	<0.001*
	5th to 10th standard			2.41 (1.51,3.87)*	<0.001*
	**TB site**				
	Extrapulmonary			Ref	
	Pulmonary			1.37 (0.96,1.95)	0.08
	**Previously treated for TB**				
	No			Ref	
	Yes			1.58 (1.18,2.11)*	0.003*
	**Drug resistant-TB**				
	No			Ref	
	Yes			2.33 (1.41,3.87)*	0.001*
	**Drink alcohol**				
	No			Ref	
	Yes			1.38 (1.01,1.88)*	0.04*
	**HIV**				
	No			Ref	
	Yes			2.61 (1.41,4.82)*	0.002*
	**Diabetes**				
	No			Ref	
	Yes			0.70 (0.37,1.32)	0.27
	**Initial weight**				
	Greater than median			Ref	
	Less than median			1.89 (1.43,2.50)*	<0.001*
	Unknown			1.70 (1.22,2.37)*	0.002*
Washington, 2020 (Karnataka and Telangana) *Population: Presumed drug-susceptible new and previously treated TB patients and multidrug-resistant TB patients (sputum smear positive pulmonary, sputum smear negative pulmonary, and*				Values below are adjusted odds ratios	

extrapulmonary) as a combined population Outcome: Death as a single outcome [125]					
	**Sex**				
	Female			Ref	
	Male			0.95 (0.61,1.48)	0.83
	**Age (years)**				
	Below 60			Ref	
	60 and above			2.15 (1.37,3.37)*	0.001*
	**State**				
	Telangana			Ref	
	Karnataka			1.54 (1.01,2.35)*	0.05*
	**Religion**				
	Muslim			Ref	
	Hindu			0.94 (0.62,1.45)	0.79
	Other religion			1.71 (0.75,3.91)	0.21
	**Marital status**				
	Single			Ref	
	Married			1.09 (0.62,1.93)	0.76
	Marriage dissolved			0.88 (0.35,2.23)	0.8
	**Living alone**				
	No			Ref	
	Yes			0.68 (0.24,1.89)	0.46
	**Education**				
	10th standard or more			Ref	
	Less than 5th standard			5.38 (2.10,13.83)*	<0.001*
	5th to 10th standard			3.99 (1.55,10.31)*	<0.004*
	**TB site**				
	Extrapulmonary			Ref	
	Pulmonary			1.09 (0.66,1.80)	0.74
	**Previously treated for TB**				
	No			Ref	
	Yes			1.65 (1.08,2.51)*	0.02*
	**Drug-resistant TB**				
	No			Ref	
	Yes			1.83 (0.86,3.89)	0.12
	**Drink alcohol**				
	No			Ref	
	Yes			2.09 (1.35,3.25)*	0.001*
	**HIV**				
	No			Ref	
	Yes			4.75 (2.29,9.86)*	<0.001*

	**Diabetes**				
	No			Ref	
	Yes			0.74 (0.29,1.86)	0.52
	**Initial weight**				
	Greater than median			Ref	
	Less than median			1.98 (1.30,3.00)*	0.001*
	Unknown			1.96 (1.22,3.15)*	0.005*
Studies in children with TB					
Dhakulkar, 2021 (Maharashtra) *Population: Children ages 0-19 with drug-resistant TB Outcome: Death, treatment failure, and loss to follow-up as a composite outcome* [133]		Values below are odds ratios		Values below are adjusted odds ratios	
	**Age (years)**				
	0-9	Ref		Ref	
	10—19	10.0 (1.3,77.1)*		4.4 (0.4,42.9)	
	**Sex**				
	Female	Ref		Ref	
	Male	1.4 (0.8,2.3)		1.2 (0.6,2.4)	
	**TB site**				
	Extrapulmonary	Ref		Ref	
	Pulmonary	3.5 (1.8,6.8)*		1.9 (0.8,4.4)	
	**TB resistant patterns**				
	Multidrug-resistant TB	Ref		Ref	
	Pre-extensively drug-resistant TB	1.9 (1.1,3.4)*		1.7 (0.8,3.4)	
	Extensively drug-resistant TB	7.5 (3.2,17.3)*		4.3 (1.3,13.8)*	
	**Previous TB episodes**				
	Absent	Ref		Ref	
	Present	2.0 (1.2,3.4)*		1.4 (0.7,2.9)	
	**Nutritional status**				
	Normal	Ref		Ref	
	Undernourished	3.8 (2.1,6.9)*		2.5 (1.3,4.8)*	
Raizada, 2018[a] (4 Indian States) *Population: Children ages 0-14 with drug-susceptible TB Outcome: Death as a single outcome versus treatment completion (with exclusion of patients still on treatment, with treatment failure, or with loss to follow-up from the analysis)* [134]		Values below are odds ratios			
	**Age (years)**				

	10 to 14	Ref			
	5 to 9	1.59 (0.86,2.94)	0.14		
	0 to 4	3.31 (1.92,5.72)*	<0.0001*		
	**Sex**				
	Female	Ref			
	Male	1.36 (0.84,2.19)	0.21		
Raizada, 2018[a] (4 Indian States)<br>*Population: Children ages 0-14 with drug-susceptible TB*<br>*Outcome: Death, treatment failure, and loss to follow-up as a composite outcome versus treatment completion (with exclusion of patients still on treatment from the analysis)* [134]		Values below are odds ratios			
	**Age (years)**				
	10 to 14	Ref			
	5 to 9	1.56 (0.88,2.75)	0.12		
	0 to 4	2.76 (1.63,4.67)*	0.0002*		
	**Sex**				
	Female	Ref			
	Male	1.17 (0.74,1.86)	0.49		
Sadana, 2020[a] (Punjab)<br>*Population: Children 0-14 years with new or previously treated TB*<br>*Outcome: Death, treatment modified, and loss to follow-up as a composite outcome* [135]		Values below are odds ratios			
	**Age (years)**				
	0 to 5	Ref			
	6 to 10	7.0 (0.32,152.96)	0.22		
	11 to 14	0.82 (0.03,21.58)	0.9		
	**Sex**				
	Male	Ref			
	Female	0.45 (0.06,3.45)	0.44		
	**Area of residence**				
	Urban	Ref			
	Rural	1.14 (0.11,11.81)	0.91		
	**Diagnosis**				
	Ziehl-Neelson sputum microscopy	Ref			
	Chest X-Ray	2.30 (0.13,40.55)	0.57		
	Others	2.19 (0.18,25.96)	0.53		
	Cartridge-based nucleic acid amplification testing	1.74 (0.06,49.90)	0.75		

	**Site**				
	Pulmonary	Ref			
	Extrapulmonary	3.21 (0.32,32.74)	0.32		
	**Type of case**				
	Retreatment (previously treated)	Ref			
	New	0.93 (0.04,19.55)	0.96		
	**Contact history**				
	Absent	Ref			
	Present	33.5 (1.69,665.30)*	0.02*		
Satyanarayana, 2010[a] (Delhi) *Population: Children 0-14 years with new or previously treated TB* *Outcome: Death, treatment failure, lost to follow-up, and transferred out as a composite outcome* [136]		Values below are odds ratios			
	**Sex**				
	Female	Ref			
	Male	0.70 (0.39,1.26)	0.23		
	**Age (years)**				
	<5	Ref			
	5 to 10	1.19 (0.46,3.07)	0.72		
	11 to 15	1.59 (0.65,3.89)	0.31		
	**TB classification**				
	Extrapulmonary	Ref			
	Pulmonary	2.08 (1.20,3.61)*	0.009*		
	**Type of TB**				
	New extrapulmonary	Ref			
	New smear negative	1.85 (0.89,3.86)	0.1		
	New smear positive	2.78 (1.33,5.83)*	0.007*		
	Previously treated "other" (i.e., smear negative or extrapulmonary)	3.23(1.16,9.00)*	0.02*		
	Previously treated smear positive	5.82 (1.57,21.60)*	0.008*		
	**Extrapulmonary TB site**				
	Peripheral lymph node	Ref			
	Other sites	1.27 (0.51,3.21)	0.61		
	**Treatment category**				
	Category I (mostly new smear positive pulmonary TB patients)	Ref			
	Category II (previously treated TB patients)	2.40 (1.07,5.37)*	0.03*		

	Category III (mostly new smear negative and extrapulmonary TB patients who were not seriously ill)	0.69 (0.31,1.50)	0.35		
	**RNTCP pre-treatment weight bands**				
	>17-25 kg	Ref			
	<=10 kg	1.17 (0.42,3.27)	0.76		
	>10-17 kg	0.95 (0.42,2.17)	0.9		
	>25-30 kg	1.38 (0.60,3.18)	0.45		
	>30 kg	0.74 (0.33,1.64)	0.46		
	**DOT center type**				
	Government health facility	Ref			
	Other DOT provider	0.87 (0.45,1.65)	0.67		
Studies in people with HIV being treated for TB					
Ambadekar, 2015[a,b,***] (Maharashtra) *Outcome: Death, treatment failure, loss to follow-up, and transfer out as a composite outcome* [127]		Values below are odds ratios		Values below are adjusted odds ratios	
	**Age (years)**				
	<14	Ref		Ref	
	>14	6.09 (1.46,25.34)*	0.01	5.18 (1.24,21.7)*	
	**Co-trimoxazole Prophylaxis Therapy (CPT) status**				
	CPT +	Ref		Ref	
	CPT -	1.61 (1.1,2.36)*	0.01	1.44 (0.97,2.12)	
	**Type**				
	Pulmonary	1.85 (1.3,2.64)*	0.001	1.72 (1.2,2.48)*	
	Extrapulmonary	Ref		Ref	
	**Patient Status**				
	New	Ref		Ref	
	Retreatment	1.80 (1.17,2.78)*	0.008	1.60 (1.03,2.5)*	
Ambadekar, 2015[a] (Maharashtra) *Outcome: Death as a single outcome* [127]		Values below are odds ratios		Values below are adjusted odds ratios	
	**Age (years)**				
	<14	0.14 (0.02,1.01)	0.05	0.17 (0.02,1.23)	
	>14	Ref		Ref	
	**Co-trimoxazole Prophylaxis Therapy (CPT) status**				
	CPT +	0.63 (0.04,0.99)*	0.05	0.71 (0.45,1.13)	
	CPT -	Ref		Ref	

	**Type**				
	Pulmonary	2.21 (1.42,3.44)*	0.0005	2.08 (1.33,3.25)*	
	Extrapulmonary	Ref		Ref	
	**Patient Status**				
	New	0.60 (0.36,1.00)	0.05	0.68 (0.40,1.15)	
	Retreatment	Ref		Ref	
Madan, 2018 (New Delhi) *Outcome: Death, treatment failure, loss to follow-up, transfer out, and switched to a MDR-TB treatment regimen as a composite outcome* [128]		Values below are relative risks			
	**Age (years)**				
	>=55	Ref			
	45-54	0.88 (0.4,1.8)	0.73		
	35-44	0.72 (0.4,1.4)	0.34		
	25-34	0.87 (0.4,1.6)	0.66		
	15-24	1.5 (0.8,3.0)	0.18		
	**Sex**				
	Female	Ref			
	Male	1.1 (0.8,1.6)	0.57		
	Transgender	1.4 (0.3,8.0)	0.7		
	**Type of patient**				
	New	Ref			
	Previously treated	1.6 (1.2,2.1)*	0.002*		
	**Disease type**				
	Extrapulmonary	Ref			
	Pulmonary	2.4 (1.8,3.4)*	<0.001*		
	Both	2.8 (1.5,5.3)*	0.002*		
	**Sputum smear grade**				
	Negative	Ref			
	1+	1.9 (1.2,2.9) *	0.006*		
	2+	1.7(0.95,3.0)	0.07		
	3+	2.3 (1.6,2.5)*	<0.001*		
	Scanty	0.99 (0.3,2.8)	0.99		
	Positive, unknown	1.6 (0.5,1.1)	0.28		
	**CD4 cell count at ATT initiation (cells/mm^3)**				
	>500	Ref			
	350-499	2.7(0.6,12)	0.23		
	200-349	4.2 (1.0,17)*	0.04*		
	51-199	4.8 (1.2,19)*	0.02*		
	>50	7.0 (1.7,29)*	0.007*		

	**HIV status at TB diagnosis**				
	Known HIV, on ART	Ref			
	Known HIV, not on ART	2.07 (1.3,3.0)*	0.003*		
	HIV diagnosis after TB diagnosis	0.78 (0.5,1.1)	0.20		
	Known HIV, ART status not recorded at diagnosis	1.48 (1.03,2.1)*	0.03*		
		Values below are odds ratios			
	**TB treatment category and smear grade**				
	New smear Negative	Ref			
	Smear positive (grade 1+)	5.78 (1.84,18.16)*	0.003*		
	New smear Positive (grade 2+)	2.69 (0.32,22.71)	0.36		
	New smear Positive (grade 3+)	1.69 (0.41,6.93)	0.47		
	New TB (no sputum smear)	1.67 (0.43,6.56)	0.46		
	New smear positive (grade unknown)	1.00 (0.38,2.61)	0.10		
	Previously treated smear Negative	1.35 (0.57,3.18)	0.49		
	Previously treated smear Positive (grade Scanty)	4.74 (0.20,108.42)	0.33		
	Previously treated smear positive (grade 1+)	1.61 (0.24,10.76)	0.62		
	Previously treated smear Positive (grade 2+)	1.05 (0.04,29.31)	0.98		
	Previously treated smear Positive (grade 3+)	7.54 (1.30,43.73)*	0.02*		
	Retreatment TB (no sputum smear)	2.46 (0.84,7.17)	0.098		
	Previously treated smear positive (grade unknown)	30.37 (2.47,373.47)*	0.008*		
	**Disease classification**				
	Extra-pulmonary	Ref			
	Pulmonary	1.83 (1.19,2.81)*	0.005*		
	Both	5.86 (2.12,16.19)*	0.001*		
	**HIV diagnosis status at TB diagnosis and CD4 Cell count (cells/mm3)**				
	> = 500	Ref			
	> = 350-<500	8.09 (0.48,135.90)	0.15		
	> = 200-<350	6.14 (0.68,54.93)	0.10		
	>50-<200	16.35 (1.14,234.17)*	0.04*		
	<50	38.76 (1.16,1289.61)*	0.04*		
	**Known HIV seropositive not on ART with CD4 cell count**				
	> = 500	Ref			
	> = 350-<500	53.44 (1.07,2656.37)	0.05*		

	> = 200-<350	65.98 (828.00,525.49)	0.00*		
Maji, 2022 (New Delhi)<br>*Outcome: Death and loss to follow-up as a composite outcome* [129]		Values below are odds ratios		Variables below are adjusted odds ratios	
	**Age (years)**				
	<35	Ref			
	≥35	1.45 (0.29,7.23)	0.46		
	**Sex**				
	Female	Ref			
	Male	1.08 (0.11,10.37)	0.95		
	**Marital status**				
	Married	Ref			
	Unmarried	0.17 (0.009,3.10)	0.23		
	**Socio-economic status**				
	Upper middle	Ref			
	Lower middle	0.31 (0.005,18.00)	0.57		
	Upper lower	0.96 (0.04,23.48)	0.98		
	Lower	9.00 (0.39,210.40)	0.17		
	**Socio-economic status**				
	Reference group is not clearly denoted to understand findings			20.50 (3.11,134.95)*	0.772
	**Functional status**				
	Working	Ref			
	Ambulatory	14.33 (0.71,290.45)	0.08		
	Bedridden	21.5 (2.54,182.38)*	0.005*		
	**Functional status**				
	Reference group is not clearly denoted to understand findings			16.50 (2.10,129.63)*	0.002*
	**BMI (kg/m^2)**				
	>18.5	Ref		Ref	
	≤18.5	86.00 (7.65,966.21)*	0.0003*	86.00 (7.65,966.21)*[h]	<0.001*
	**Type of tuberculosis**				
	PTB	Ref			
	EPTB	1.15 (0.19,7.07)	0.88		
	PTB with EPTB	1.50 (0.11,20.30)	0.76		
	**Previously treated with ATT**				
	No	Ref			
	Yes	0.69 (0.07,6.43)	0.74		
	**Chest X-ray laterality**				
	Normal	Ref			
	Unilateral	1.30 (0.12,14.21)	0.83		

	Bilateral	0.87 (0.14,5.31)	0.88		
	**Cavity in chest X-ray**				
	No	Ref			
	Yes	0.91 (0.16,5.29)	0.92		
	**Anaemia (Hb <12 g/dl)**				
	No	Ref		Ref	
	Yes	10.24 (1.13,92.38)*	0.04*	10.23 (1.13,92.38)*h	0.04*
	**Hypoalbuminemia (<3.5 g/dl)**				
	No	Ref		Ref	
	Yes	17.79 (0.96,329.77)	0.053	17.79 (0.96,329.77)*h	0.588
	**CD4 cell count at TB diagnosis (<100 cells/mm^3)**				
	No	Ref		Ref	
	Yes	9.00 (1.51,53.54)*	0.02*	9.00 (1.51,53.54)*h	0.898
Ranganath, 2021 (Karnataka) *Outcome: Non-adherence as a single outcome* [130]		Values below are odds ratios		Values below are adjusted odds ratios	
	**Age (years)**				
	38-47	Ref			
	18-27	0.71 (0.31,1.60)	0.265		
	28-37	0.74 (0.46,1.18)			
	48-57	1.46 (0.72,2.96)			
	>58	0.59 (0.22,1.56)			
	**Sex**				
	Male	Ref			
	Female	1.14 (0.74,1.75)	0.58		
	**Religion**				
	Hindu	Ref			
	Muslim	1.08 (0.34,3.40)	0.97		
	Others	1.23 (0.25,6.04)			
	**SES**				
	Upper middle	Ref			
	Lower class	1.73 (0.88,3.41)	0.27		
	Lower middle	1.66 (0.94,2.93)			
	Middle class	1.10 (0.61,1.99)			
	Upper class	1.11 (0.39,3.16)			
	**Family type**				
	Nuclear family	Ref			
	Joint family	2.56 (1.03,6.34)*	0.03*		
	**Wage loss**				
	<250	Ref			

	250-499	0.49 (0.28,0.85)*	0.04*		
	500-749	0.66 (0.37,1.18)*			
	750-99	0.65 (0.34,1.26)			
	≥1000	0.22 (0.06,0.82)*			
	**Type of TB**				
	Extra-pulmonary	Ref			
	Pulmonary	0.78 (0.51,1.18)	0.14		
	**Type of diagnosis**				
	BC	Ref			
	CC	0.94 (0.63,1.42)	0.44		
	**Past history**				
	New case	Ref			
	Previously diagnosed	1.65 (1.01,2.71)*	0.03*		
	**Family history of TB**				
	No	Ref			
	Yes	0.08 (0.03,0.21)*	<0.001*		
	**Co-morbidities**				
	No	Ref			
	Yes	0.18 (0.08,0.39)*	<0.001*		
	**Smoking**				
	No	Ref			
	Yes	1.20 (0.64,2.28)	0.33		
	**Alcohol Use**				
	No	Ref			
	Yes	0.65 (0.37,1.13)	0.06		
	**BMI (Kg/m^2)**				
	Underweight	Ref			
	Normal	0.27 (0.18,0.40)	0.15		
	Overweight	0.89 (0.34,2.35)			
	**Duration of HIV (years)**				
	>3	Ref			
	<3	1.52 (1.01,2.31)*	0.04*		
	**Place of diagnosis**				
	IRL	Ref			
	DMC	1.35 (0.81,2.25)	0.37		
	Private labs	1.35 (0.77,2.36)			
	**Type of treatment supporter**				
	Health care worker	Ref			
	Family/friend	0.38 (0.24,0.61)*	<0.001*		
	**Counseling before initiation of TB treatment**				
	Yes	Ref		Ref	

	No	5.52 (3.57,8.55)*	<0.001*	5.25 (3.01,9.17)*	<0.001*
	**HIV regimen**				
	Tenofovir based	Ref		Ref	
	Zidovudine based	5.01 (3.13,7.98)*	<0.001*	4.87 (2.69,8.83)*	0.013*
	Others	1.98 (0.50,7.85)		3.71 (0.60,22.7)	
	**Side effects of treatment**				
	Nil	Ref		Ref	
	<3	4.89 (2.71,8.82)*	<0.001*	2.26 (1.25,4.08)*	0.006*
	>3	2.08 (1.16,3.73)*		6.45 (3.03,13.7)*	0.001*
	**Status disclosure**				
	Yes	Ref		Ref	
	No	2.25 (1.48,3.41)*	<0.001*	2.06 (1.19,3.58)*	0.012*
	**Co-trimoxazole prophylaxis**				
	Yes	Ref		Ref	
	No	13.24 (5.85,29.9)*	<0.001*	9.90 (2.92,33.5)*	<0.001*
Sharma, 2014 (Delhi) *Outcome: Death, treatment failure, and loss to follow-up as a composite outcome* [131]		Values below are odds ratios		Values below are adjusted odds ratios	
	**Age (years)**				
	>40	Ref			
	<=40	1.17 (0.67,2.03)	0.57		
	**Sex**				
	Female	Ref			
	Male	1.97 (1.02,3.8)*	0.04*	1.87 (0.96,3.67)	0.06
	**Classification**				
	Extrapulmonary	Ref			
	Pulmonary	1.16 (0.73,1.84)	0.51		
	**Sputum smear**				
	Smear negative	Ref			
	Smear positive	1.41 (0.67,2.96)	0.35		
	**CD4 count/mm^3**				
	>200	Ref			
	<=200	2.61 (1.20,5.66)*	0.01*	2.32 (1.06,5.09)*	0.03*
	**Patient type**				
	New	Ref			
	Previously treated	3.33 (1.42,7.81)*	0.004*	2.91 (1.22,6.89)*	0.02*
	**ATT type**				
	Daily therapy	Ref			
	DOTS	0.99 (0.53,1.85)	0.98		
	**ATT side effects**				
	No	Ref			

	Yes	1.52 (0.90,2.57)	0.11		
	**ART at diagnosis of TB**				
	On ART	No			
	ART naïve	2.62 (0.90,7.58)	0.06	2.42 (0.82,7.07)	0.1
Shastri, 2013[a] (Karnataka) *Outcome: Death, treatment failure, and loss to follow-up as a composite outcome* [132]		Values below are odds ratios			
	**ART**				
	On ART	Ref			
	Not on ART	2.83 (2.43,3.29)*	<0.0001*		
	**HIV co-infection**				
	TB only	Ref			
	HIV/TB co-infection	1.16 (1.08,1.25)*	<0.0001*		
Vijay, 2011 (Karnataka) *Outcome: Death, treatment failure, and loss to follow-up as a composite outcome* [137]		Values below are odds ratios		Values below are adjusted odds ratios	
	**Disease Classification**				
	Pulmonary	2.46 (1.35,4.53)	0.002*	1.96 (1.02,3.77)*	0.04*
	Extra-pulmonary	Ref		Ref	
	**Sputum smear**				
	Smear positive	1.77 (0.80,3.94)	0.12		
	Smear negative	Ref			
	**Type of patient**				
	Previously treated patient	4.04 (1.96,8.35)	<0.001*	4.78 (2.12,10.76)*	<0.001*
	New patient	Ref		Ref	
	**Regularity of treatment**				
	Irregular	1.63 (0.86,3.09)	0.107		
	Regular	Ref			
	**Baseline CD4 count/mm^3**				
	<=350	0.80 (0.26,3.29)	0.83		
	>350	Ref			
	**ART initiation**				
	Not on ART	9.12 (4.48,18.88)*	<0.001*	4.9 (1.85,12.96)*	0.001*
	On ART	Ref		Ref	
	**CPT provision**				
	Not initiated	6.65 (3.53,12.61)*	<0.001*	2.19 (0.90,5.32)	0.08
	Initiated	Ref		Ref	
Vijay, 2011 (Karnataka) *Outcome: Death as a single outcome* [137]		Values below are odds ratios		Values below are adjusted odds ratios	
	**Disease Classification**				
	Pulmonary	2.21 (1.26,3.88)*	0.002*	1.82 (1.00,1.33)*	0.050*

	Extra-pulmonary	Ref		Ref	
	**Sputum smear**				
	Smear positive	1.53 (0.72,3.26)	0.23		
	Smear negative	Ref			
	**Type of patient**				
	Previously treated patient	2.05 (1.01,4.18)*	0.03*	1.94 (0.89,4.21)	0.09
	New patient	Ref		Ref	
	**Regularity of treatment**				
	Irregular	2.07 (1.14,3.78)*	0.01*		
	Regular	Ref			
	**Baseline CD4 count/mm^3**				
	<=200	0.59 (0.26,1.35)	0.17		
	>200	Ref			
	**ART initiation**				
	Not on ART	7.75 (4.12,14.71)*	<0.001*	2.80 (1.15,6.81)*	0.023*
	On ART	Ref		Ref	
	**Cotrimoxazole prophylactic therapy provision**				
	Not initiated	7.76 (4.22,14.35)*	<0.001*	3.46 (1.47,8.14)*	0.004*
	Initiated	Ref		Ref	

SAT, Self-administered Treatment; DOTS, Directly Observed Treatment Shortcourse; TB, tuberculosis; km, kilometer; OBC, Other Backward Class; kg, kilogram; SES, socioeconomic status; PHI, peripheral health institution; SAMVAD, Sensitization and Advocacy in Marginalized and Vulnerable Areas of the District; BMI, body mass index; IP, intensive phase; LTFU, loss to follow-up; HIV, human immunodeficiency virus; ATT, anti-tuberculosis therapy; ART, anti-retroviral therapy; INH, isoniazid; TAD, treatment after default; RNTCP, Revised National TB Control Programme; NGO, non-governmental organization; S, Streptomycin; H, Isoniazid; E, Ethambutol; MDR, multidrug-resistant; XDR, extensively drug-resistant; CBNAAT, cartridge-based nucleic acid amplification testing; CDST, Culture and Drug Susceptibility Testing; CP, continuous phase, AUDIT-C, Alcohol Use Disorders Identification Test-Consumption; AFB; acid-fast bacillus; SC/ST/NT, Scheduled Caste/Scheduled Tribe/Nomadic Tribe; IQR, interquartile range; STS, Senior Treatment Supervisor; INR, Indian Rupee; DMC, designated microscopy center; CD4, clusters of differentiation 4; CPT, cotrimoxazole preventive therapy.

*Indicates statistical significance

**The treatment category of retreatment "other" was used by the TB program to refer to previously treated patients with smear-negative or extrapulmonary TB.

***The reference group, and therefore the effect estimate and confidence interval, was "flipped" to facilitate comparability of the reference group with other studies

[a]Unadjusted odds ratios and/or p-values were estimated by the systematic review team from the raw data, as these were not provided in the original study.

[b]Study reported successful treatment as the outcome, so effect estimates (odds ratio or relative risk and 95% confidence interval) were flipped to show effect estimates for the outcome of not achieving treatment success.

[c]Variable was included in the analysis as a continuous variable, we have specified the unit of change in the variable associated with the effect estimate.

[d]This is also an adjusted model but represents analysis on an unweighted cohort (i.e., only individuals who responded to a survey)

[e]This is an adjusted model that represents analysis on a weighted cohort (i.e., includes individual who responded to a survey

[f]Reference group was switched for comparability across studies, resulting in flipping of the effect estimate

[g]This is an adjusted model that came out of propensity score matching

[h]We are not including multivariate analyses in Forest Plots as the multivariate findings appear the exact same as univariate analyses we calculated, suggesting these adjusted multivariate values may not be accurate

References

1. Subbaraman R, Nathavitharana RR, Satyanarayana S, Pai M, Thomas BE, Chadha VK, et al. The Tuberculosis Cascade of Care in India's Public Sector: A Systematic Review and Meta-analysis. PLoS Med. 2016;13: e1002149. doi:10.1371/journal.pmed.1002149

2. Ahmed J, Chadha VK, Singh S, Venkatachalappa B, Kumar P. Utilization of RNTCP services in rural areas of Bellary District, Karnataka, by gender, age and distance from health centre. Indian J Tuberc. 2009;56: 62–68.

3. Babiarz KS, Suen S, Goldhaber-Fiebert JD. Tuberculosis treatment discontinuation and symptom persistence: an observational study of Bihar, India's public care system covering >100,000,000 inhabitants. BMC Public Health. 2014;14: 418. doi:10.1186/1471-2458-14-418

4. Bagchi S, Ambe G, Sathiakumar N. Determinants of Poor Adherence to Anti-Tuberculosis Treatment in Mumbai, India. Int J Prev Med. 2010;1: 223–32.

5. Balasubramanian R, Garg R, Santha T, Gopi PG, Subramani R, Chandrasekaran V, et al. Gender disparities in tuberculosis: report from a rural DOTS programme in south India. Int J Tuberc Lung Dis. 2004;8: 323–332.

6. Barathi A, Krishnamoorthy Y, Sinha P, Horsburgh C, Hochberg N, Johnson E, et al. Effect of treatment adherence on the association between sex and unfavourable treatment outcomes among tuberculosis patients in Puducherry, India: a mediation analysis. J Public Health (Oxf). 2023;45: 304–311. doi:10.1093/pubmed/fdac062

7. Bhatt AN, Tharyan P, Michael JS, Christopher DJ, Varghese GM, Sathyendra S, et al. Treatment outcomes with daily self-administered treatment and thrice-weekly directly-observed treatment in two cohorts of newly-diagnosed, sputum-positive adults with pulmonary tuberculosis. Indian Journal of Tuberculosis. 2020;67: 105–111. doi:10.1016/j.ijtb.2017.05.012

8. Chakrabarti S, Saha I, Das DK, Prasad Sarkar A, Roy R, Hossain A. Comparative study of the profiles of tribal and non-tribal tuberculosis patients in a tuberculosis unit of West Bengal, India. int j tuberc lung dis. 2012;16: 1205–1209. doi:10.5588/ijtld.11.0501

9. Chen AZ, Kumar R, Baria RK, Shridhar PK, Subbaraman R, Thies W. Impact of the 99DOTS digital adherence technology on tuberculosis treatment outcomes in North India: a pre-post study. BMC Infect Dis. 2023;23: 504. doi:10.1186/s12879-023-08418-2

10. Gopi PG, Chandrasekaran V, Subramani R, Santha T, Thomas A, Selvakumar N, et al. Association of conversion & cure with initial smear grading among new smear positive pulmonary tuberculosis patients treated with Category I regimen. Indian J Med Res. 2006;123: 807–814.

11. Gopalan N, Srinivasalu VA, Chinnayan P, Velayutham B, Bhaskar A, Santhanakrishnan R, et al. Predictors of unfavorable responses to therapy in rifampicin-sensitive pulmonary tuberculosis using an integrated approach of radiological presentation and sputum mycobacterial burden. PLoS One. 2021;16: e0257647. doi:10.1371/journal.pone.0257647

12. Gupta H, Mahajan S, Lal M, Toor AK, Deepti SS, Chawla N. Prevalence of tobacco consumption and smoking and its effect on outcome among microbiologically confirmed new pulmonary tuberculosis patients on daily regimen of DOTS in Amritsar city. J Family Med Prim Care. 2022;11: 2150–2154. doi:10.4103/jfmpc.jfmpc_1170_21

13. Joseph N. Treatment outcomes among new smear positive and retreatment cases of tuberculosis in Mangalore, South India – a descriptive study. AMJ. 2011;4: 162–167. doi:10.4066/AMJ.2011.585

14. Kulkarni P, Akarte S, Mankeshwar R, Bhawalkar J, Banerjee A, Kulkarni A. Non-adherence of new pulmonary tuberculosis patients to anti-tuberculosis treatment. Ann Med Health Sci Res. 2013;3: 67–74. doi:10.4103/2141-9248.109507

15. Mave V, Gaikwad S, Barthwal M, Chandanwale A, Lokhande R, Kadam D, et al. Diabetes Mellitus and Tuberculosis Treatment Outcomes in Pune, India. Open Forum Infectious Diseases. 2021;8: ofab097. doi:10.1093/ofid/ofab097

16. Mukherjee A, Sarkar A, Saha I, Biswas B, Bhattacharyya P. Outcomes of different subgroups of smear-positive retreatment patients under RNTCP in rural West Bengal, India. Rural and Remote Health. 2009;9: 926. doi:10.22605/RRH926

17. Mukherjee A, Saha I, Sarkar A, Chowdhury R. Gender differences in notification rates, clinical forms and treatment outcome of tuberculosis patients under the RNTCP. Lung India. 2012;29: 120-122. doi:10.4103/0970-2113.95302

18. Prajapati AC, Shah T, Panchal S, Joshi B, Shringarpure K, Jakasania A, et al. Treatment outcomes and associated factors among patients with drug-sensitive tuberculosis on daily fixed-dose combination drugs: A cohort study from Ahmedabad, India. J Family Med Prim Care. 2023;12: 452–459. doi:10.4103/jfmpc.jfmpc_1331_22

19. Ramachandran G, Chandrasekaran P, Gaikwad S, Agibothu Kupparam HK, Thiruvengadam K, Gupte N, et al. Subtherapeutic Rifampicin Concentration Is Associated With Unfavorable Tuberculosis Treatment Outcomes. Clin Infect Dis. 2020;70: 1463–1470. doi:10.1093/cid/ciz380

20. Rouf A, Masoodi MA, Dar MM, Khan SMS, Bilquise R. Depression among Tuberculosis patients and its association with treatment outcomes in district Srinagar. J Clin Tuberc Other Mycobact Dis. 2021;25: 100281. doi:10.1016/j.jctube.2021.100281

21. M. S, K. M, Marconi S, V. K, S. R, Prasad J. A community based case control study on risk factors for treatment interruptions in people with tuberculosis in Kollam district, Kerala, southern India. Int J Community Med Public Health. 2016;3: 962–967. doi:10.18203/2394-6040.ijcmph20160937

22. Shewade HD, Gupta V, Satyanarayana S, Pandey P, Bajpai UN, Tripathy JP, et al. Patient characteristics, health seeking and delays among new sputum smear positive TB patients identified through active case finding when compared to passive case finding in India. PLoS One. 2019;14: e0213345. doi:10.1371/journal.pone.0213345

23. Shivam S, Saha I, Mondal T, Misra R, Dasgupta S, Roy R, et al. Gender Differentials in Tuberculosis: An Experience from a Rural Tuberculosis Unit of Burdwan District, West Bengal, India. TAF Prev Med Bull. 2014;13: 109. doi:10.5455/pmb.1-1371096967

24. Singla R, Sarin R, Khalid UK, Mathuria K, Singla N, Jaiswal A, et al. Seven-year DOTS-Plus pilot experience in India: results, constraints and issues. Int J Tuberc Lung Dis. 2009;13: 976–981.

25. Singla R, Bharty SK, Gupta UA, Khayyam KU, Vohra V, Singla N, et al. Sputum smear positivity at two months in previously untreated pulmonary tuberculosis patients. Int J Mycobacteriol. 2013;2: 199–205. doi:10.1016/j.ijmyco.2013.08.002

26. Sinha P, Ponnuraja C, Gupte N, Prakash Babu S, Cox SR, Sarkar S, et al. Impact of Undernutrition on Tuberculosis Treatment Outcomes in India: A Multicenter, Prospective, Cohort Analysis. Clin Infect Dis. 2023;76: 1483–1491. doi:10.1093/cid/ciac915

27. Tiwari S, Kumar A, Kapoor SK. Relationship between sputum smear grading and smear conversion rate and treatment outcome in the patients of pulmonary tuberculosis underoing DOTS--A Prospective cohort study. Indian Journal of Tuberculosis. 2012; 59(3):135-140.

28. Trivedi PR, Khakhkhar TM. Treatment outcome of tuberculosis patients under directly observed treatment short-course and factors affecting the outcome in tertiary care hospital. Int J Basic Clin Pharmacol. 2019;8: 981. doi:10.18203/2319-2003.ijbcp20191588

29. Umayorubhagom A, Baliga SS. Factors affecting tuberculosis treatment outcome among newly diagnosed tuberculosis patients – A longitudinal study. Indian J Tuberc. 2023. Epub 2023 Jun 7. doi:10.1016/j.ijtb.2023.06.007

30. Vashishtha R, Mohan K, Singh B, Devarapu SK, Sreenivas V, Ranjan S, et al. Efficacy and safety of thrice weekly DOTS in tuberculosis patients with and without HIV co-infection: an observational study. BMC Infect Dis. 2013;13: 468. doi:10.1186/1471-2334-13-468

31. Velayutham B, Nair D, Chandrasekaran V, Raman B, Sekar G, Watson B, et al. Profile and Response to Anti-Tuberculosis Treatment among Elderly Tuberculosis Patients Treated under the TB Control Programme in South India. PLoS ONE. 2014;9: e88045. doi:10.1371/journal.pone.0088045

32. Velayutham B, Chadha VK, Singla N, Narang P, Gangadhar Rao V, Nair S, et al. Recurrence of tuberculosis among newly diagnosed sputum positive pulmonary tuberculosis patients treated under the Revised National Tuberculosis Control Programme, India: A multi-centric prospective study. PLoS One. 2018;13: e0200150. doi:10.1371/journal.pone.0200150

33. Vijay S, Kumar P, Chauhan LS, Vollepore BH, Kizhakkethil UP, Rao SG. Risk Factors Associated with Default among New Smear Positive TB Patients Treated Under DOTS in India. PLoS ONE. 2010;5: e10043. doi:10.1371/journal.pone.0010043

34. Viswanathan V, Vigneswari A, Selvan K, Satyavani K, Rajeswari R, Kapur A. Effect of diabetes on treatment outcome of smear-positive pulmonary tuberculosis—A report from South India. Journal of Diabetes and its Complications. 2014;28: 162–165. doi:10.1016/j.jdiacomp.2013.12.003

35. Viswanathan V, Devarajan A, Kumpatla S, Dhanasekaran M, Babu S, Kornfeld H. Effect of prediabetes on tuberculosis treatment outcomes: A study from South India. Diabetes Metab Syndr. 2023;17: 102801. doi:10.1016/j.dsx.2023.102801

36. Zaman F, Sheikh S, Das K, Zaman G, Pal R. An epidemiological study of newly diagnosed sputum positive tuberculosis patients in Dhubri district, Assam, India and the factors influencing their compliance to treatment. J Nat Sc Biol Med. 2014;5: 415–420. doi:10.4103/0976-9668.136213

37. Zhou TJ, Lakshminarayanan S, Sarkar S, Knudsen S, Horsburgh CR, Muthaiah M, et al. Predictors of Loss to Follow-Up among Men with Tuberculosis in Puducherry and Tamil Nadu, India. The American Journal of Tropical Medicine and Hygiene. 2020;103: 1050–1056. doi:10.4269/ajtmh.19-0415

38. Ahmed MV, Nirgude AS, Naik PR, Mandolikar RY. Assessment of patient related risk factors pertaining to default and non-default among study population. Journal of Pharmaceutical Negative Results. 2022; 2410–2415. doi:10.47750/pnr.2022.13

39. Bhagat VM, Gattani PL. Factors affecting tuberculosis retreatment defaults in Nanded, India. Southeast Asian J Trop Med Public Health. 2010;41: 1153–1157.

40. Chandrasekaran V, Gopi PG, Santha T, Subramani R, Narayanan PR. Status of re-registered patients for tuberculosis treatment under DOTS programme. Indian Journal of Tuberculosis. 54: 12–18.

41. Deepa D, Achanta S, Jaju J, Rao K, Samyukta R, Claassens M, et al. The Impact of Isoniazid Resistance on the Treatment Outcomes of Smear Positive Re-Treatment Tuberculosis Patients in the State of Andhra Pradesh, India. PLoS ONE. 2013;8: e76189. doi:10.1371/journal.pone.0076189

42. Jha UM, Satyanarayana S, Dewan PK, Chadha S, Wares F, Sahu S, et al. Risk Factors for Treatment Default among Re-Treatment Tuberculosis Patients in India, 2006. PLoS ONE. 2010;5: e8873. doi:10.1371/journal.pone.0008873

43. Burugina Nagaraja S, Satyanarayana S, Chadha SS, Kalemane S, Jaju J, Achanta S, et al. How do patients who fail first-line TB treatment but who are not placed on an MDR-TB regimen fare in South India? PLoS One. 2011;6: e25698. doi:10.1371/journal.pone.0025698

44. Pardeshi G. Time of default in tuberculosis patients on directly observed treatment. J Global Infect Dis. 2010;2: 226-230. doi:10.4103/0974-777X.68533

45. Sarpal SS, Goel NK, Kumar D, Janmeja AK. Treatment Outcome Among the Retreatment Tuberculosis (TB) Patients under RNTCP in Chandigarh, India. J Clin Diagn Res. 2014;8: 53–56. doi:10.7860/JCDR/2014/6510.4006

46. Sisodia RS, Wares DF, Sahu S, Chauhan LS, Zignol M. Source of retreatment cases under the Revised National TB Control Programme in Rajasthan, India, 2003. Int J Tuberc Lung Dis. 2006;10: 1373-1379.

47. Srinath S, Sharath B, Santosha K, Chadha SS, Roopa S, Chander K, et al. Tuberculosis “retreatment others”: profile and treatment outcomes in the state of Andhra Pradesh, India. Int J Tuberc Lung Dis. 2011;15: 105–109.

48. Velavan A, Purty AJ, Shringarpure K, Sagili KD, Mishra AK, Selvaraj KS, et al. Tuberculosis retreatment outcomes and associated factors: a mixed-methods study from Puducherry, India. public health action. 2018;8: 187–193. doi:10.5588/pha.18.0038

49. Bhatt R, Chopra K, Vashisht R. Impact of integrated psycho-socio-economic support on treatment outcome in drug resistant tuberculosis – A retrospective cohort study. Indian Journal of Tuberculosis. 2019;66: 105–110. doi:10.1016/j.ijtb.2018.05.020

50. Dela A, Tank NK, Singh A, Piparva K. Adverse drug reactions and treatment outcome analysis of DOTS-plus therapy of MDR-TB patients at district tuberculosis centre: A four year retrospective study. Lung India. 2017;34: 522-526. doi:10.4103/0970-2113.217569

51. Dash M, Behera BP. Socioepidemiological status and clinical outcome of MDR TB patients in a tertiary medical college in Southern Odisha. J Family Med Prim Care. 2022;11: 1275–1281. doi:10.4103/jfmpc.jfmpc_1015_21

52. Dole SS, Waghmare VN, Shaikh AM. Clinical Profile and Treatment Outcome of Drug Resistant Tuberculosis Patients of Western Maharashtra, India. J Assoc Physicians India. 2017;65: 18–21.

53. Duraisamy K, Mrithyunjayan S, Ghosh S, Nair SA, Balakrishnan S, Subramoniapillai J, et al. Does Alcohol Consumption during Multidrug-resistant Tuberculosis Treatment Affect Outcome?. A Population-based Study in Kerala, India. Ann Am Thorac Soc. 2014;11: 712–718. doi:10.1513/AnnalsATS.201312-447OC

54. Giri VP, Giri OP, Pandey PT, Mishra KN, Prasad RS, Lal PK, et al. The Characteristics and Patterns of Drug-Resistant Pulmonary Tuberculosis in Eastern India. Trop Med Infect Dis. 2022;7: 244. doi:10.3390/tropicalmed7090244

55. Isaakidis P, Varghese B, Mansoor H, Cox HS, Ladomirska J, Saranchuk P, et al. Adverse Events among HIV/MDR-TB Co-Infected Patients Receiving Antiretroviral and Second Line Anti-TB Treatment in Mumbai, India. Wilkinson RJ, editor. PLoS ONE. 2012;7: e40781. doi:10.1371/journal.pone.0040781

56. Jain K, Desai M, Solanki R, Dikshit RK. Treatment outcome of andardized regimen in patients with multidrug resistant tuberculosis. Journal of Pharmacology and Pharmacotherapeutics. 2014;5: 145–149. doi:10.4103/0976-500X.130062

57. Janmeja AK, Aggarwal D, Dhillon R. Factors predicting treatment success in multi-drug resistant tuberculosis patients treated under programmatic conditions. Indian J Tuberc. 2018;65: 135–139. doi:10.1016/j.ijtb.2017.12.015

58. Johnson JM, Mohapatra AK, Velladath SU, Shettigar KS. Predictors of Treatment Outcomes in Drug Resistant Tuberculosis-Observational Retrospective Study. The International Journal of Mycobacteriology. 2022;11: 38-46. doi:10.4103/ijmy.ijmy_244_21

59. Kalagani Y, Chary VG. Predictors of Unfavorable Treatment Outcome in Patients with Multidrug-Resistant Tuberculosis: A Prospective Study. European Journal of Molecular and Clinical Medicine. 2022;9: 4662–4668.

60. Kandi S, K TK, Kandi SR, Mathur N, D CD, Adepu R. Study of treatment outcomes of multidrug-resistant tuberculosis under programmatic conditions and factors influencing the outcomes in Hyderabad District. Indian Journal of Tuberculosis. 2021;68: 379–383. doi:10.1016/j.ijtb.2020.12.008

61. Keshari A, Gupta A, Bhatnagar A. Treatment Outcomes of Isoniazid Monoresistant Pulmonary Tuberculosis Patients Under RNTCP. J Ind Acad Clin Med. 2023;24: 34–39.

62. B K, Singla R, Singla N, V V, Singh K, Choudhury MP, et al. Factors affecting the treatment outcome of injection based shorter MDR-TB regimen at a referral centre in India. Monaldi Arch Chest Dis. 2022;93. doi:10.4081/monaldi.2022.2396

63. Rajesh Kumar B, Senthilkumar A, Murugan JA, Ramasamy H, Rathinam P. Clinico-Epidemiological Profile and Treatment Outcome of Multi- Drug Resistant Tuberculosis Patients of a South Indian District: An Observational Study. International Journal of Pharmaceutical and Clinical Research. 15: 438–443.

64. Kumari SL, Kongara S, Bhaskar K, Srikanti R, Bhushana Rao CRN, Sanjana PH. Outcomes and adherence of shorter MDR TB regimen in patients with multidrug resistant tuberculosis. Indian J Tuberc. 2023;70:103-106. doi: 10.1016/j.ijtb.2022.03.021.

65. Lohiya S, Tripathy JP, Sagili K, Khanna V, Kumar R, Ojha A, et al. Does Drug-Resistant Extrapulmonary Tuberculosis Hinder TB Elimination Plans? A Case from Delhi, India. Trop Med Infect Dis. 2020;5: 109. doi:10.3390/tropicalmed5030109

66. Nair D, Navneethapandian PD, Tripathy JP, Harries AD, Klinton JS, Watson B, et al. Impact of rapid molecular diagnostic tests on time to treatment initiation and outcomes in patients with multidrug-resistant tuberculosis, Tamil Nadu, India. Trans R Soc Trop Med Hyg. 2016;110: 534–541. doi:10.1093/trstmh/trw060

67. Natarajan S, Singla R, Singla N, Gupta A, Caminero JA, Chakraborty A, et al. Treatment interruption patterns and adverse events among patients on bedaquiline containing regimen under programmatic conditions in India. Pulmonology. 2022;28: 203–209. doi:10.1016/j.pulmoe.2020.09.006

68. Parmar MM, Sachdeva KS, Dewan PK, Rade K, Nair SA, Pant R, et al. Unacceptable treatment outcomes and associated factors among India's initial cohorts of multidrug-resistant tuberculosis (MDR-TB) patients under the revised national TB control programme (2007–2011): Evidence leading to policy enhancement. PLoS ONE. 2018;13: e0193903. doi:10.1371/journal.pone.0193903

69. Patel SV, Nimavat KB, Patel AB, Mehta KG, Shringarpure K, Shukla LK. Sputum Smear and Culture Conversion in Multidrug Resistance Tuberculosis Patients in Seven Districts of Central Gujarat, India: A Longitudinal Study. Indian J Community Med. 2018;43: 117–119. doi:10.4103/ijcm.IJCM_152_17

70. Rupani M, Dave J, Parmar V, Singh M, Parikh K. Adverse drug reactions and risk factors for discontinuation of multidrug-resistant tuberculosis regimens in Gujarat, western India. Natl Med J India. 2020;33: 10–14. doi:10.4103/0970-258X.308234

71. Saha A, Vaidya PJ, Chavhan VB, Pandey KV, Kate AH, Leuppi JD, et al. Factors affecting outcomes of individualised treatment for drug resistant tuberculosis in an endemic region. Indian J Tuberc. 2019;66: 240–246. doi:10.1016/j.ijtb.2017.04.001

72. Shringarpure KS, Isaakidis P, Sagili KD, Baxi RK. Loss-To-Follow-Up on Multidrug Resistant Tuberculosis Treatment in Gujarat, India: The WHEN and WHO of It. Mistry N, editor. PLoS ONE. 2015;10: e0132543. doi:10.1371/journal.pone.0132543

73. Velayutham B, Shah V, V. Mythily, Gopalaswamy R, Kumar N, Mandal S, et al. Factors influencing treatment outcomes in patients with isoniazid-resistant pulmonary TB. Int J Tuberc Lung Dis. 2022;26: 1033–1040. doi:10.5588/ijtld.21.0701

74. Ahmed M, Mohan R. A comparative study of factors for interruption of antitubercular treatment among defaulters in urban and rural areas of Kamrup District, Assam. J Family Med Prim Care. 2021;10: 127–131. doi:10.4103/jfmpc.jfmpc_1027_20

75. Banerjee S, Bandyopadhyay K, Taraphdar P, Dasgupta A. Perceived discrimination among tuberculosis patients in an urban area of Kolkata City, India. J Global Infect Dis. 2020;12: 144–148. doi:10.4103/jgid.jgid_146_19

76. Bhagyalaxmi A, Jain S, Kadri A. Effectiveness of different models of DOTS providers under RNTCP in Ahmedabad City, Gujarat. Indian J Community Med. 2010;35: 495-497. doi:10.4103/0970-0218.74356

77. Bhargava A, Chatterjee M, Jain Y, Chatterjee B, Kataria A, Bhargava M, et al. Nutritional status of adult patients with pulmonary tuberculosis in rural central India and its association with mortality. PLoS One. 2013;8: e77979. doi:10.1371/journal.pone.0077979

78. Brahmapurkar K, Brahmapurkar V, Zodpey S. Sputum smear grading and treatment outcome among directly observed treatment-short course patients of tuberculosis unit, Jagdalpur, Bastar. J Family Med Prim Care. 2017;6: 293-296. doi:10.4103/jfmpc.jfmpc_24_16

79. Cox SR, Gupte AN, Thomas B, Gaikwad S, Mave V, Padmapriyadarsini C, et al. Unhealthy alcohol use independently associated with unfavorable TB treatment outcomes among Indian men. Int J Tuberc Lung Dis. 2021;25: 182–190. doi:10.5588/ijtld.20.0778

80. Dandona R, Dandona L, Mishra A, Dhingra S, Venkatagopalakrishna K, Chauhan LS. Utilization of and barriers to public sector tuberculosis services in India. Natl Med J India. 2004;17: 292–299.

81. Das M, Isaakidis P, Shenoy R, Anicete R, Sharma HK, Ao I, et al. Self-Administered Tuberculosis Treatment Outcomes in a Tribal Population on the Indo-Myanmar Border, Nagaland, India. PLoS ONE. 2014;9: e108186. doi:10.1371/journal.pone.0108186

82. Dey A, Lahiri A, Jha SS, Sharma V, Shanmugam P, Chakrabartty AK. Treatment adherence status of the TB patients notified from private sector and its associated factors: Findings of a secondary data analysis from West Bengal, India. Indian J Tuberc. 2022;69: 334–340. doi:10.1016/j.ijtb.2021.06.001

83. Gopi PG, Vasantha M, Muniyandi M, Chandrasekaran V, Balasubramanian R, Narayanan PR. Risk factors for non-adherence to directly observed treatment (DOT) in a rural tuberculosis unit, South India. Indian Journal of Tuberculosis. 2007;54: 66–70.

84. Gupta S, Gupta S, Behera D. Reasons for interruption of anti-tubercular treatment as reported by patients with tuberculosis admitted in a tertiary care institute. Indian J Tuberc. 2011;58: 11–17.

85. Huddart S, Singh M, Jha N, Benedetti A, Pai M. Case fatality and recurrent tuberculosis among patients managed in the private sector: A cohort study in Patna, India. PLOS One. 2021;16: e0249225. doi:10.1371/journal.pone.0249225

86. Islam S, Das S, Das DK. Nutritional status and adherence to anti-tubercular treatment among tuberculosis patients in a community development block of Eastern India. Indian J Tuberc. 2023. Epub 2023 Apr 18. doi:10.1016/j.ijtb.2023.04.005

87. Jaggarajamma K, Sudha G, Chandrasekaran V, Nirupa C, Thomas A, Santha T, et al. Reasons for non-compliance among patients treated under Revised National Tuberculosis Control Programme (RNTCP), Tiruvallur district, south India. Indian J Tuberc. 2007;54: 130–135.

88. Jaiswal S, Sharma H, Joshi U, Agrawal M, Sheohare R. Non-adherence to anti-tubercular treatment during COVID-19 pandemic in Raipur district Central India. Indian J Tuberc. 2022;69: 558–564. doi:10.1016/j.ijtb.2021.08.033

89. Secretary of Jan Swasthya Sahyog, Laux TS, Patil S. Predictors of tuberculosis treatment outcomes among a retrospective cohort in rural, Central India. Journal of Clinical Tuberculosis and Other Mycobacterial Diseases. 2018;12: 41–47. doi:10.1016/j.jctube.2018.06.005

90. Jonnalagada S, Harries AD, Zachariah R, Satyanarayana S, Tetali S, Keshav Chander G, et al. The timing of death in patients with tuberculosis who die during anti-tuberculosis treatment in Andhra Pradesh, South India. BMC Public Health. 2011;11: 921. doi:10.1186/1471-2458-11-921

91. Kamble BD, Malhotra S. Profile and treatment outcomes among young patients with tuberculosis aged 15-24 years in Faridabad district of Haryana, India. BMJ Open. 2022;12: e060363. doi:10.1136/bmjopen-2021-060363

92. Karanjekar V, Gujarati V, Lokare P. Sociodemographic factors associated with health seeking behavior of chest symptomatics in urban slums of Aurangabad city, India. International Journal of Basic and Applied Medical Sciences. 2014;4: 173–179.

93. Kumar R, Ahirwar RK, Dave L, Srivastava N, Bajpai A, Jain S, et al. Treatment outcome and efficacy of anti-tuberculosis treatment in tuberculosis patients put on DOTS in RNTCP in Central India. J Evol Med Dent Sci. 2018;7: 1840–1844. doi:10.14260/jemds/2018/416

94. Kuruva P, Kandi SR, Kandi S. Clinico-radiological profile and treatment outcome of pulmonary tuberculosis with and without type 2 diabetes mellitus. Indian J Tuberc. 2021;68: 249–254. doi:10.1016/j.ijtb.2020.09.020

95. Lata S, Khajuria V, Sawhney V, Kumari K. Evaluation of non-adherence to antitubercular drugs among tuberculosis patients: a prospective study. Int J Curr Pharm Res. 2021;13: 26–28. doi:10.22159/ijcpr.2021v13i2.41550

96. Maroof M, Pamei G, Bhatt M, Awasthi S, Bahuguna SC, Singh P. Drug adherence to anti-tubercular treatment during COVID-19 lockdown in Haldwani block of Nainital district. Indian Journal of Community Health. 2022;34: 535–541. doi:10.47203/IJCH.2022.v34i04.016

97. Mittal C, Gupta S. Noncompliance to DOTS: How it can be decreased. Indian J Community Med. 2011;36: 27–30. doi:10.4103/0970-0218.80789

98. Mittal C, Gupta SC. Effect of Disease Related Variables on Treatment Outcome Under DOTS. JK Science: Journal of Medical Education & Research. 2011;13: 15-18.

99. Motappa R, Fathima T, Kotian H. Appraisal on patient compliance and factors influencing the daily regimen of anti-tubercular drugs in Mangalore city: A cross-sectional study. F1000Res. 2022;11: 462. doi:10.12688/f1000research.109006.2

100. Mukhopadhyay S, Sarkar A. Comparative analysis of RNTCP indicators in a rural and an urban tuberculosis unit of Burdwan district in West Bengal. Indian J Community Med. 2011;36: 146. doi:10.4103/0970-0218.84136

101. Mundra A, Deshmukh PR, Dawale A. Magnitude and determinants of adverse treatment outcomes among tuberculosis patients registered under Revised National Tuberculosis Control Program in a Tuberculosis Unit, Wardha, Central India: A record-based cohort study. J Epidemiol Glob Health. 2017;7: 111–118. doi:10.1016/j.jegh.2017.02.002

102. Mundra A, Deshmukh P, Dawale A. Determinants of adverse treatment outcomes among patients treated under Revised National Tuberculosis Control Program in Wardha, India: Case–control study. Med J Armed Forces India. 2018;74: 241–249. doi:10.1016/j.mjafi.2017.07.008

103. Nahar N, Phadnis S, Joshi A, Lodha R, Tiwari SC, Nandeshwar S, et al. A Study on Non-Adherence to Anti Tubercular Treatment in RNTCP. Indian Journal of Public Health Research & Development. 2014;5: 113-116. doi:10.5958/j.0976-5506.5.1.027

104. Nandakumar K, Duraisamy K, Balakrishnan S, M S, S JS, Sagili KD, et al. Outcome of Tuberculosis Treatment in Patients with Diabetes Mellitus Treated in the Revised National Tuberculosis Control Programme in Malappuram District, Kerala, India. PLoS ONE. 2013;8: e76275. doi:10.1371/journal.pone.0076275

105. Nandi C, Mitra K, Bhaumik D. Determinants of treatment interruption and outcome among smear-positive pulmonary tuberculosis patients in a tuberculosis unit of Purba Bardhaman district of West Bengal. J Family Med Prim Care. 2022;11: 1134–1139. doi:10.4103/jfmpc.jfmpc_1105_21

106. Panati D, Chittooru CS, Madarapu YR, Gorantla AK. Effect of depression on treatment adherence among elderly tuberculosis patients: A prospective interventional study. Clinical Epidemiology and Global Health. 2023;22: 101338. doi:10.1016/j.cegh.2023.101338

107. Pardeshi GS, Deshmukh D. A comparison of treatment outcome in re-treatment versus new smear positive cases of tuberculosis under RNTCP. Indian J Public Health. 2007;51: 237–239.

108. Patra S, Lukhmana S, Tayler Smith K, Kannan AT, Satyanarayana S, Enarson DA, et al. Profile and treatment outcomes of elderly patients with tuberculosis in Delhi, India: implications for their management. Transactions of the Royal Society of Tropical Medicine and Hygiene. 2013;107: 763–768. doi:10.1093/trstmh/trt094

109. Paunikar AP, Khadilkar HA, Doibale MK, Lamb AR. Survival Analysis of Treatment Defaulters among Tuberculosis Patients in Government Medical College and Hospital, Aurangabad. Indian Journal of Community Medicine. 2019;44: 44-47.

110. Pore P, Kumar A, Farooqui I. Noncompliance to directly observed treatment short course in Mulshi block, Pune district. Indian J Community Med. 2020;45: 291-294. doi:10.4103/ijcm.IJCM_137_19

111. Potty RS, Kumarasamy K, Adepu R, Reddy RC, Singarajipura A, Siddappa PB, et al. Community health workers augment the cascade of TB detection to care in urban slums of two metro cities in India. J Glob Health. 2021;11: 04042. doi:10.7189/jogh.11.04042

112. Potty RS, Kumarasamy K, Munjattu JF, Reddy RC, Adepu R, Singarajipura A, et al. Tuberculosis treatment outcomes and patient support groups, southern India. Bull World Health Organ. 2023;101: 28-35A. doi:10.2471/BLT.22.288237

113. Prudhivi R, Challa SR, Rao MV B, Veena G V, Rao N B, Manogna Narne H. Assessment of Success Rate of Directly Observed Treatment Short-Course (DOTS) in Tuberculosis Patients of South India. J Young Pharm. 2018;11: 67–72. doi:10.5530/jyp.2019.11.14

114. Ratnesh, Umashankar H, Sudeepa D. Treatment Seeking Behaviour among TB Patients Registered Under RNTCP in District Bareilly. Indian Journal of Public Health Research & Development. 2020;11: 79-83. doi:10.37506/v11/i2/2020/ijphrd/194757

115. Saini S, Singh M. Treatment outcome among HIV positive and HIV negative TB patients in Chandigarh, India: A retrospective cohort study. European Respiratory Journal. 2015;46:PA2741. doi:10.1183/13993003.congress-2015.PA2741

116. Shabil M, Rajesh V, Raj KCB, Rajesh KS, Shama KP, Gururaja MP, et al. A Study on Treatment Defaulters in Tuberculosis Patients on DOTS Therapy. Res J Pharm Technol. 2019;12: 2245–53. doi:10.5958/0974-360X.2019.00374.3

117. Sharma SK, Lawaniya S, Lal H, Singh UB, Sinha PK. DOTS Centre at a Tertiary Care Teaching Hospital: Lessons Learned and Future Directions. Indian Journal Chest Dis Allied Sci. 2004;46: 251-256.

118. Sharma V, Thekkur P, Naik PR, Saha BK, Agrawal N, Dinda MK, et al. Treatment success rates among tuberculosis patients notified from the private sector in West Bengal, India. Monaldi Arch Chest Dis. 2021;91. doi:10.4081/monaldi.2021.1555

119. Siddiqui AN, Khayyam KU, Sharma M. Effect of Diabetes Mellitus on Tuberculosis Treatment Outcome and Adverse Reactions in Patients Receiving Directly Observed Treatment Strategy in India: A Prospective Study. BioMed Res Int. 2016;2016: 1–11. doi:10.1155/2016/7273935

120. Singh M, Sagili KD, Tripathy JP, Kishore S, Bahurupi YA, Kumar A, et al. Are Treatment Outcomes of Patients with Tuberculosis Detected by Active Case Finding Different From Those Detected by Passive Case Finding? J Glob Infect Dis. 2020;12: 28–33. doi:10.4103/jgid.jgid_66_19

121. Sodhi R, Penkunas MJ, Pal A. Free drug provision for tuberculosis increases patient follow-ups and successful treatment outcomes in the Indian private sector: a quasi experimental study using propensity score matching. BMC Infect Dis. 2023;23: 421. doi:10.1186/s12879-023-08396-5

122. Subbaraman R, Thomas BE, Kumar JV, Thiruvengadam K, Khandewale A, Kokila S, et al. Understanding Nonadherence to Tuberculosis Medications in India Using Urine Drug Metabolite Testing: A Cohort Study. Open Forum Infect Dis. 2021;8: ofab190. doi:10.1093/ofid/ofab190

123. Vasantha M, Gopi PG, Subramani R. Survival of tuberculosis patients treated under DOTS in a rural tuberculosis unit (TU), South India. Indian Journal of Tuberculosis. 2008;55: 64–69.

124. Vasudevan K, Jayakumar N, Gnanasekaran D. Smear Conversion, Treatment Outcomes and the Time of Default in Registered Tuberculosis Patients on RNTCP DOTS in Puducherry, Southern India. J Clin Diagn Res. 2014;8: JC05-8. doi:10.7860/JCDR/2014/8421.4984

125. Washington R, Potty RS, Rajesham A, Seenappa T, Singarajipura A, Swamickan R, et al. Is a differentiated care model needed for patients with TB? A cohort analysis of risk factors contributing to unfavourable outcomes among TB patients in two states in South India. BMC Public Health. 2020;20: 1158. doi:10.1186/s12889-020-09257-5

126. Yadav GS, Jangid VK, Mathur BB. Study of various reasons for interruption of anti-tubercular treatment in patients of tuberculosis reporting to tertiary care center of west Rajasthan. Int J Res Med Sci. 2019;7: 2220-2226. doi:10.18203/2320-6012.ijrms20192542

127. Ambadekar NN, Zodpey SP, Soni RN, Lanjewar SP. Treatment outcome and its attributes in TB-HIV co-infected patients registered under Revised National TB Control Program: a retrospective cohort analysis. Public Health. 2015;129: 783–789. doi:10.1016/j.puhe.2015.03.006

128. Madan C, Chopra KK, Satyanarayana S, Surie D, Chadha V, Sachdeva KS, et al. Developing a model to predict unfavourable treatment outcomes in patients with tuberculosis and human immunodeficiency virus co-infection in Delhi, India. PLoS One. 2018;13: e0204982. doi:10.1371/journal.pone.0204982

129. Maji D, Agarwal U, Kumar L, V V, Sharma A. Clinicodemographic profile and outcome of tuberculosis treatment in TB-HIV co-infected patients receiving daily ATT under a single window TB/HIV services delivery initiative. Monaldi Arch Chest Dis. 2022;93. doi:10.4081/monaldi.2022.2405

130. Ranganath TS, Kishore SG, Reddy R, Murthy HJD, Vanitha B, Sharath BN, et al. Risk factors for non-adherence among people with HIV-associated TB in Karnataka, India: A case-control study. Indian J Tuberc. 2022;69: 65–72. doi:10.1016/j.ijtb.2021.03.003

131. Sharma SK, Soneja M, Prasad KT, Ranjan S. Clinical profile & predictors of poor outcome of adult HIV-tuberculosis patients in a tertiary care centre in north India. Indian J Med Res. 2014;139: 154–160.

132. Shastri S, Naik B, Shet A, Rewari B, De Costa A. TB treatment outcomes among TB-HIV co-infections in Karnataka, India: how do these compare with non-HIV tuberculosis outcomes in the province? BMC Public Health. 2013;13: 838. doi:10.1186/1471-2458-13-838

133. Dhakulkar S, Das M, Sutar N, Oswal V, Shah D, Ravi S, et al. Treatment outcomes of children and adolescents receiving drug-resistant TB treatment in a routine TB programme, Mumbai, India. PLoS ONE. 2021;16: e0246639. doi:10.1371/journal.pone.0246639

134. Raizada N, Khaparde SD, Salhotra VS, Rao R, Kalra A, Swaminathan S, et al. Accelerating access to quality TB care for pediatric TB cases through better diagnostic strategy in four major cities of India. PLoS One. 2018;13: e0193194. doi:10.1371/journal.pone.0193194

135. Sadana P, Verma V, Nagpal M. A study on predictors of treatment outcome among children registered under DOTS in district Tarn Taran, Punjab. Indian J Community Health. 2020;32: 399–403. doi:10.47203/IJCH.2020.v32i02.017

136. Satyanarayana S, Shivashankar R, Vashist RP, Chauhan LS, Chadha SS, Dewan PK, et al. Characteristics and Programme-Defined Treatment Outcomes among Childhood Tuberculosis (TB) Patients under the National TB Programme in Delhi. PLoS ONE. 2010;5: e13338. doi:10.1371/journal.pone.0013338

137. Vijay S, Kumar P, Chauhan LS, Narayan Rao SV, Vaidyanathan P. Treatment Outcome and Mortality at One and Half Year Follow-Up of HIV Infected TB Patients Under TB Control Programme in a District of South India. Pai M, editor. PLoS ONE. 2011;6: e21008. doi:10.1371/journal.pone.0021008
