# Supplementary material for: Barriers to engagement in the care cascade for tuberculosis disease in India: A systematic review of quantitative studies
Source: PLoS Med. 2024 May 28;21(5):e1004409. doi: 10.1371/journal.pmed.1004409 (PMC11166313; doi:10.1371/journal.pmed.1004409)
Supplement: S6 Appendix — (PDF) [file pmed.1004409.s006.pdf]

S6 Appendix. Extended results for all care cascade gaps

Supplement to:
Barriers to engagement in the care cascade for tuberculosis disease in India: a systematic review of quantitative studies

Authors:
Tulip A. Jhaveri, Disha Jhaveri, Amith Galivanche, Maya Lubeck-Schricker, Dominic Voehler, Mei Chung, Pruthu Thekkur, Vineet Chadha, Ruvandhi Nathavitharana, Ajay M.V. Kumar, Hemant Deepak Shewade, Katherine Powers, Kenneth H. Mayer, Jessica E. Haberer, Paul Bain, Madhukar Pai, Srinath Satyanarayana, Ramnath Subbaraman

Correspondence:
Ramnath Subbaraman, MD, MSc, FACP, FIDSA
Tufts University School of Medicine
Department of Public Health and Community Medicine
136 Harrison Ave., MV120
Boston, MA 02130, USA
Email: ramnath.subbaraman@tufts.edu

Table of Contents

Summary of characteristics and quality of the included studies across all care cascade gaps... 3

Table A. Characteristics and quality of all studies included in the review for each care cascade gap 4

Gap 1—Barriers contributing to people with TB symptoms not having sought care................ 5

Characteristics and quality of the included studies.. 5

Gap 2—Barriers to completing the TB diagnostic workup... 7

Characteristics and quality of the included studies.. 7

Fig A. Factors associated with not pursuing the TB diagnostic workup despite referral among people with TB symptoms (Gap 2). 9

Fig B. Factors associated with non-completion of sputum microscopy evaluation in a multi-site study in India (Gap 2). 10

Fig C. Reasons for non-completion of sputum microscopy evaluation (Gap 2)... 11

Fig D. Factors associated with chest X-ray non-completion (Gap 2). 12

Fig E. Reasons for chest X-ray non-completion (Gap 2). 13

Fig F. Factors associated with nucleic acid amplification testing (NAAT) or mycobacterial culture non-completion (Gap 2).. 14

Fig G. Reasons for NAAT or mycobacterial culture non-completion (Gap 2). 15

Gap 3—Barriers contributing to pretreatment loss to follow-up (PTLFU) 16

Characteristics and quality of the included studies... 16

Fig H. Reasons for pretreatment loss to follow-up among people with drug-resistant tuberculosis (Gap 3). 18

Gap 4—Barriers to treatment success among people who start TB treatment...................... 19

Characteristics and quality of the included studies... 19

Fig I. Demographic factors contributing to unfavorable treatment outcomes in people with drug-susceptible TB (Gap 4). 21

Fig J. TB clinical factors contributing to unfavorable treatment outcomes in people with drug-susceptible TB (Gap 4). 22

Fig K. Other clinical factors associated with unfavorable treatment outcomes in people with drug-susceptible TB (Gap 4). 23

Fig L. Clinical reasons contributing to unfavorable treatment outcomes in people with drug-susceptible TB (Gap 4). 24

Fig M. Reasons reported by people with rifampin-resistant or multidrug-resistant (MDR) TB for experiencing unfavorable outcomes during treatment (Gap 4).. 25

Fig N. Factors associated with unfavorable TB treatment outcomes among people with HIV (Gap 4). 26

Gap 5—Barriers to achieving recurrence-free survival after TB treatment........................... 27

Characteristics and quality of the included studies... 27

Summary of common findings across care cascade gaps 29

Table B. Statistically significant factors associated with unfavorable outcomes across multiple TB care cascade gaps in adjusted analyses. 30

Table C. Reasons reported by people with TB or TB symptoms for not seeking care or being lost from care across multiple care cascade gaps.. 31

References 32

Summary of characteristics and quality of the included studies across all care cascade gaps

Table A below summarizes the characteristics and quality of all studies, including studies that only reported findings from unadjusted analyses.

Table A. Characteristics and quality of all studies included in the review for each care cascade gap

Study characteristics	Gap 1	Gap 2	Gap 3	Gap 4	Gap 5
**Studies contributing to the systematic review**					
Total studies reported in the systematic review	15[a]	21[b]	27[e]	144[f]	19[g]
Studies reporting factors associated with unfavorable outcomes from regression analyses (including those with unadjusted or adjusted analyses)	13	17[c]	20	135	19
Studies reporting reasons from surveys of people who experienced unfavorable outcomes	9	10[d]	11	18	0
**Study setting**					
States or union territories covered by included studies	7	11	11	25	8
Studies conducted in a rural setting only	5	6	7	27	4
Studies conducted in an urban setting only	8	7	10	74	10
Studies conducted in both rural and urban settings	3	9	10	43	5
**Quality or risk of bias criteria**					
Studies that were medium or low quality for sampling strategy	0	0	0	0	0
Studies that were medium or low quality for sample size	2	2	4	37	0
Studies that were medium or low quality for the proportion of the estimated population screened for TB symptoms (Gap 1 only)	10	NA	NA	NA	NA
Studies that were medium or low quality for the proportion of individuals with TB symptoms who were interviewed (Gap 1 only)	4	NA	NA	NA	NA
Studies that were medium or low quality for the time frame of research fieldwork after start of diagnostic evaluation (Gaps 2 and 3 only)	NA	13	20	NA	NA
Studies that were medium or low quality for only using outcomes self-reported by the government TB program (Gaps 2 and 3 only)	NA	5	7	NA	NA
Studies that were medium or low quality for retrospective assessment of both exposures and outcomes (Gap 4 only)	NA	NA	NA	59	NA
Studies that were medium or low quality for only conducting passive surveillance for post-treatment TB recurrence or mortality (Gap 5 only)	NA	NA	NA	NA	9
Studies that were medium or low quality for only diagnosing TB recurrence clinically or only testing people with symptoms (Gap 5 only)	NA	NA	NA	NA	9

[a]Of 15 studies, 1 study reported data from 2 locations, so we present characteristics across 16 locations.
[b]Of 21 studies, 1 study reported 2 different outcomes, so we present characteristics across 22 analyses.
[c]Of 17 studies, 3 studies reported on non-pursual of the diagnostic workup; 4 studies reported on sputum microscopy evaluation non-completion; 2 studies reported on chest X-ray non-completion; and 8 studies reported on NAAT, line probe assay, or culture non-completion.
[d]Of 10 studies, 2 studies reported on sputum microscopy evaluation non-completion; 2 studies reported on chest X-ray non-completion; and 6 studies reported on NAAT non-completion.
HIV, human immunodeficiency virus; NA, not applicable (i.e., quality indicator not relevant to a specific gap); NAAT, nucleic acid amplification test; TB, tuberculosis.
[e]Of 27 studies assessing pretreatment loss to follow-up; 20 studies evaluated adults with drug-susceptible TB; 6 studies evaluated adults with drug-resistant TB; and 1 study evaluated children with drug-susceptible or drug-resistant TB.
[f]Of 144 studies, 108 studies evaluated people with drug-susceptible TB (34 studies in people with new TB, 19 studies in people with a prior TB treatment history, and 55 studies in people with new TB or a prior TB history); 25 studies evaluated people with drug-resistant TB; 7 studies evaluated people with HIV being treated for TB; and 4 studies evaluated children with TB.
[g]Of 19 studies, 14 studies reported on TB recurrence as a single outcome or part of a composite outcome; and 5 studies reported on post-treatment mortality as a single outcome or part of a composite outcome with on-treatment mortality.

Gap 1—Barriers contributing to people with TB symptoms not having sought care

Characteristics and quality of the included studies

Across 3 searches spanning January 1, 2000, to August 14, 2023, we screened titles and abstracts of 6,262 unique reports and identified 323 reports for full text review, of which 12 met inclusion criteria (Fig A in S1 Appendix). 1 additional study meeting inclusion criteria was identified by reviewing references of other articles [1]. 3 datasets—1 of which was linked to 1 of the 12 studies meeting inclusion criteria—were identified by outreach to experts. At our request, the authors conducted secondary analyses of those datasets to evaluate factors associated with not having sought care for TB symptoms [2–4].

As such, 15 total articles or analyses were included in the Gap 1 review (Table A above in the S6 Appendix). 1 study reported data from 2 geographic locations [5], so we present characteristics across 16 studies or locations. Of these, 13 presented findings on factors associated with not having sought care (of which 7 included adjusted analyses) and 9 presented findings on reasons for not seeking care reported by people with presumptive TB (Table C in S1 Appendix). Studies were conducted in 7 of India's 28 states and 8 union territories, including Uttar Pradesh and Madhya Pradesh, 2 of India's high-population and low-income states by gross domestic product (GDP) per capita, and 3 studies were conducted in multiple states. 5 studies were conducted in rural areas, 8 in urban areas, and 3 in both (Table C in the S1 Appendix).

All studies involved a high-quality sampling strategy (i.e., random or comprehensive sampling). 1 study was low quality with regard to sample size [6]. 10 studies did not report the proportion of people screened for presumptive TB during population-based data collection (low quality). 3 studies did not report the proportion of individuals with presumptive TB who completed an interview [32] (low quality), while, in another study, only 68% of individuals with presumptive TB were interviewed [7] (medium quality) (Table C in S1 Appendix).

Gap 2—Barriers to completing the TB diagnostic workup

Characteristics and quality of the included studies

Across searches spanning January 1, 2000, to August 14, 2023, we screened titles and abstracts of 4,262 unique reports and identified 390 for full text review, of which 21 met inclusion criteria (Fig A in S2 Appendix and Table A above in the S6 Appendix). Of these, 17 studies presented findings on factors associated with not completing steps of the diagnostic workup, 9 of which included adjusted analyses. Of the 17 studies presenting findings on factors from regression analyses, 3 reported on non-pursual of diagnostic workup despite referral [8–10]; 4 reported on non-completion of sputum microscopy evaluation [11–14]; 2 reported on chest X-ray non-completion [15,16]; and 8 reported on non-completion of nucleic acid amplification test (NAAT), line probe assay, or mycobacterial culture among people at higher risk for drug-resistant TB [15,17–23]. 10 studies described reasons for not completing the diagnostic workup, of which 2 reported on non-completion of sputum microscopy evaluation from patient interviews [12,24], 2 reported on chest X-ray non-completion from patient interviews [25,26], and 6 reported on NAAT non-completion from evaluation of health records [17–20,27,28] (Table C in S2 Appendix).

Studies were conducted in 11 of India's states and union territories (Table C in S2 Appendix). In addition, 1 study reported findings from multiple states. 4 of the studies reported findings from Bihar, Madhya Pradesh, and Chhattisgarh, which are some of India's poorest states by GDP per capita [9,13,19,28]. 7 studies were conducted in urban areas, 6 in rural areas, and 9 in both.

All studies involved high-quality sampling of patients (i.e., random or comprehensive patient sampling). 2 studies were low quality for sample size [21,26]. 9 studies did not report the time frame of research fieldwork, while 4 studies collected data or followed-up patients >3 months after presentation (low quality). 2 studies collected data or followed-up patients 1 to 3 months after presentation (medium quality). 5 studies relied on self-report by the government TB program to determine outcomes (medium to low quality) (Table C in S2 Appendix).

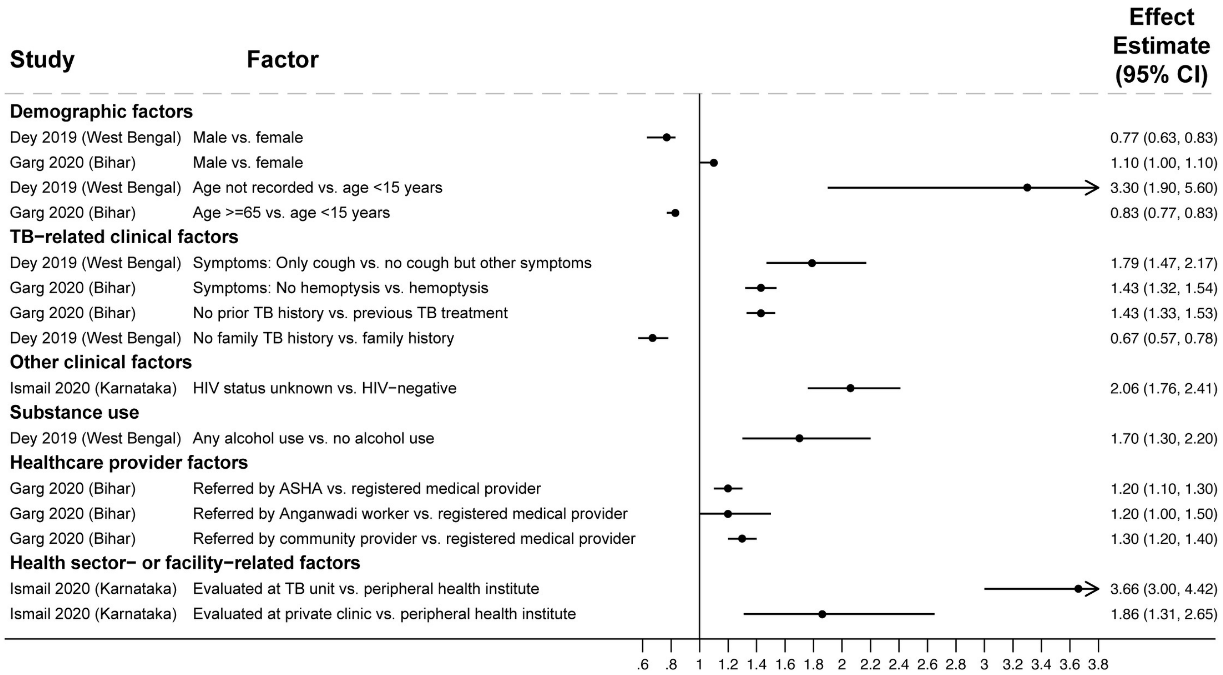

Fig A. Factors associated with not pursuing the TB diagnostic workup despite referral among people with TB symptoms (Gap 2). All studies used multivariable relative risk regression, with findings reported as adjusted risk ratios [8–10]. Effect estimates greater than 1 represent increased risk of non-pursual of the TB diagnostic workup; effect estimates less than 1 represent decreased risk of non-pursual of the TB diagnostic workup. Only statistically significant findings are presented. Some studies in the review with adjusted analyses reported non-significant findings for sex [10], age [10], and alcohol use [9]. ASHA, accredited social health activist; CI, confidence interval; HIV, human immunodeficiency virus; TB, tuberculosis.

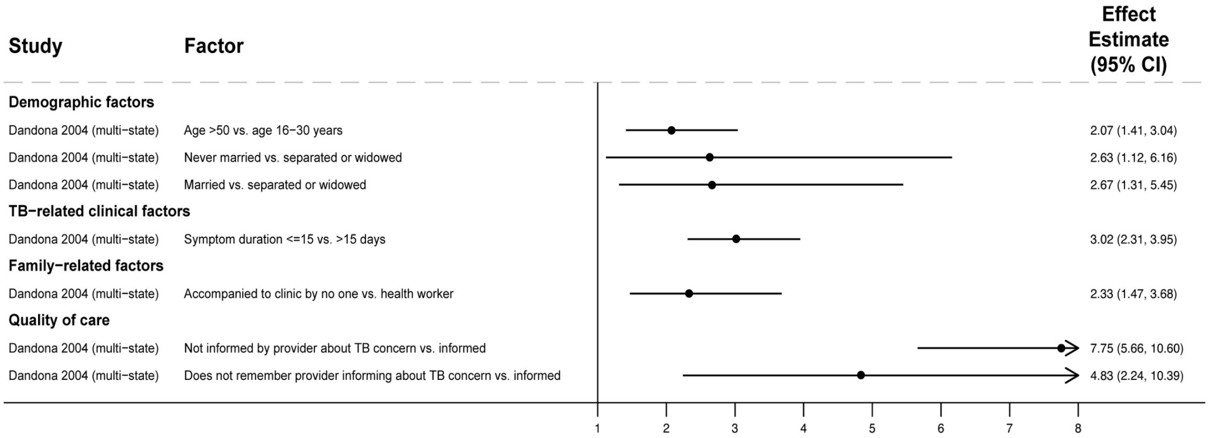

Fig B. Factors associated with non-completion of sputum microscopy evaluation in a multi-site study in India (Gap 2). The study used multivariable logistic regression, with findings reported as adjusted odds ratios [29]. Effect estimates greater than 1 represent increased odds of non-completion of sputum microscopy evaluation; effect estimates less than 1 represent decreased odds of non-completion of sputum microscopy evaluation. Only statistically significant findings are presented. CI, confidence interval; TB, tuberculosis.

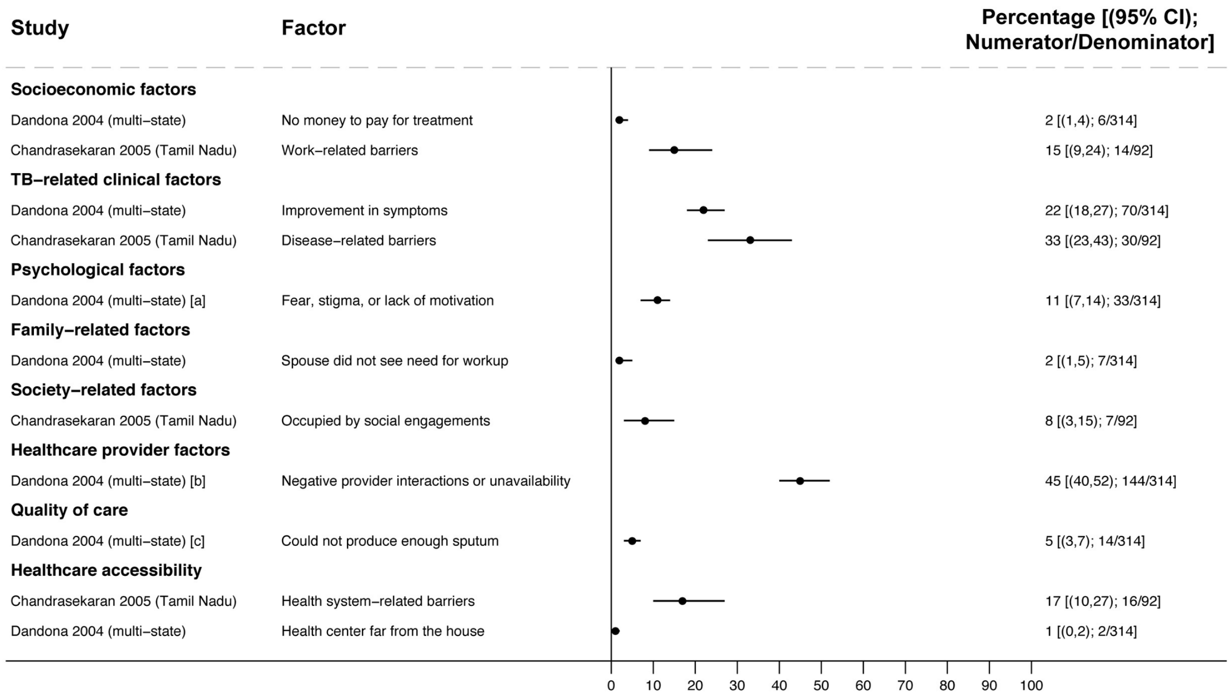

Fig C. Reasons for non-completion of sputum microscopy evaluation (Gap 2). Both studies [24,29] report estimates of the percentage of people who reported a given reason for non-completion of sputum microscopy evaluation. Study or variable labels indicate: [a] "fear, stigma, or lack of motivation" summarizes the following reasons: no time, too busy, scared of TB, did not think further workup was necessary, could not go due to ill health, did not go because knew of other patients who were not cured by government care; [b] "negative provider interactions or unavailability" summarizes: provider not aware 3 sputum samples were needed, lab personnel did not behave well towards the patient, had to wait too long at the center, lab personnel or doctor was not available; [c] "could not produce sputum" summarizes: could not produce enough sputum, referred for x-ray. CI, confidence interval.

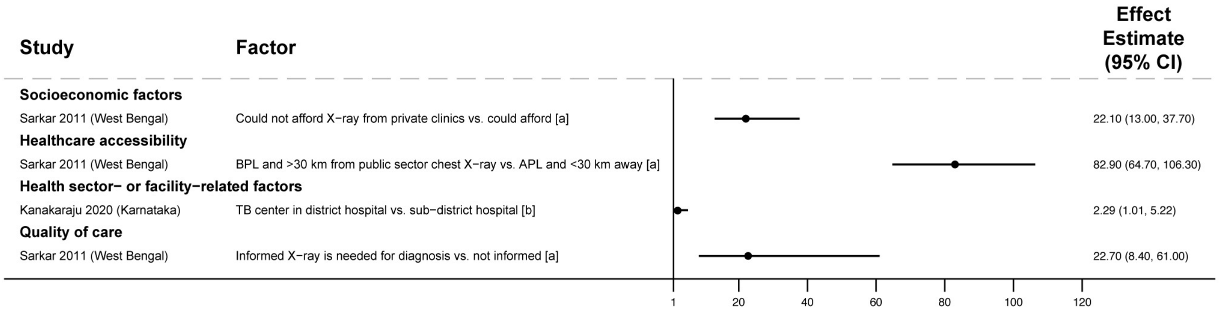

Fig D. Factors associated with chest X-ray non-completion (Gap 2). Both studies used multivariable regression, with findings reported as adjusted odds ratios [16] or relative risk ratios [15]. Effect estimates greater than 1 represent increased adjusted risk of chest X-ray non-completion. Variables labeled [a] reported effect estimates as odds ratios, while those labeled [b] reported effect estimates as relative risk ratios. Only statistically significant findings are presented. APL, above poverty line; BPL, below poverty line; CI, confidence interval; TB, tuberculosis.

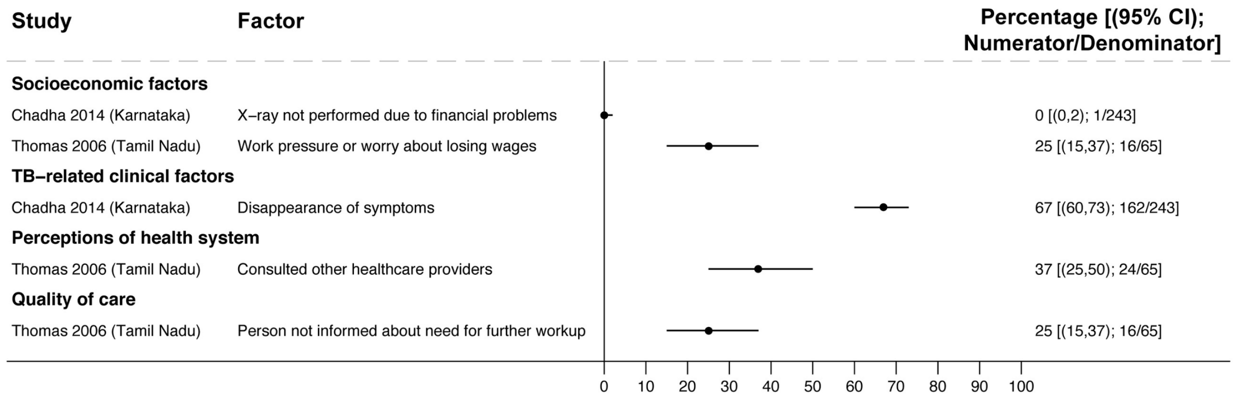

Fig E. Reasons for chest X-ray non-completion (Gap 2). Both studies [25,26] report the estimated percentage of people who reported a given reason for non-completion of chest X-ray as part of the TB diagnostic workup. CI, confidence interval.

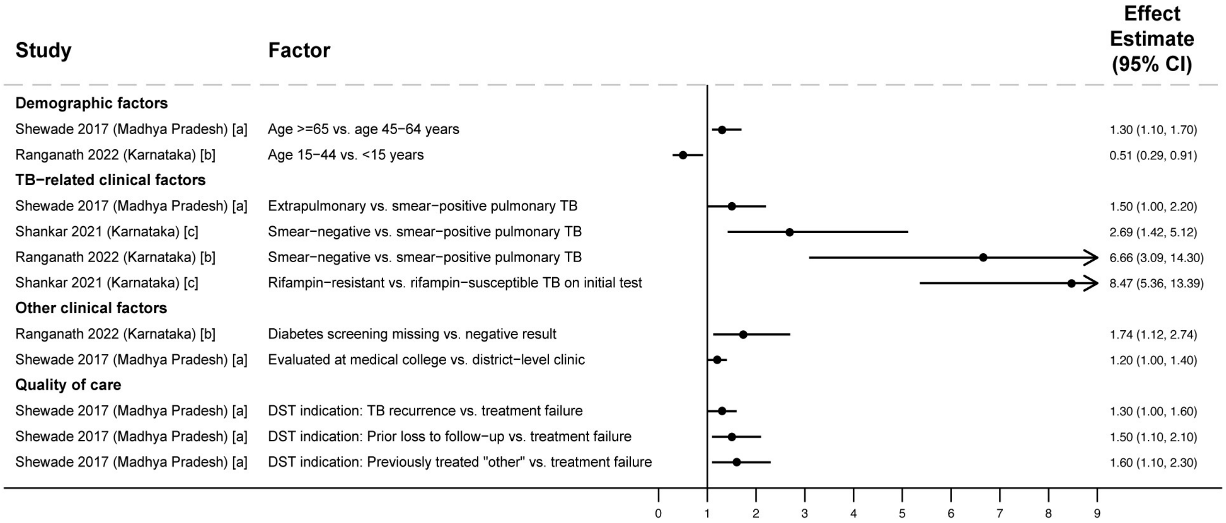

Fig F. Factors associated with nucleic acid amplification testing (NAAT) or mycobacterial culture non-completion (Gap 2). Studies used multivariable regression, with findings reported as adjusted relative risk ratios [19,22,23]. Effect estimates greater than 1 represent increased adjusted risk of test non-completion; effect estimates less than 1 represent decreased adjusted risk of test non-completion. Study labels indicate: [a] outcome is NAAT (i.e., Xpert MTB/RIF) non-completion; [b] outcome is non-completion of NAAT or line probe assay for first line drugs for initial drug-susceptibility testing; and [c] outcome is non-completion of line probe assay and/or mycobacterial culture for drug-susceptibility testing. Only statistically significant findings are presented. 1 study in the review with an adjusted analysis reported non-significant findings for extrapulmonary TB [23]. CI, confidence interval; DST, drug susceptibility testing; NAAT, nucleic acid amplification test; TB, tuberculosis.

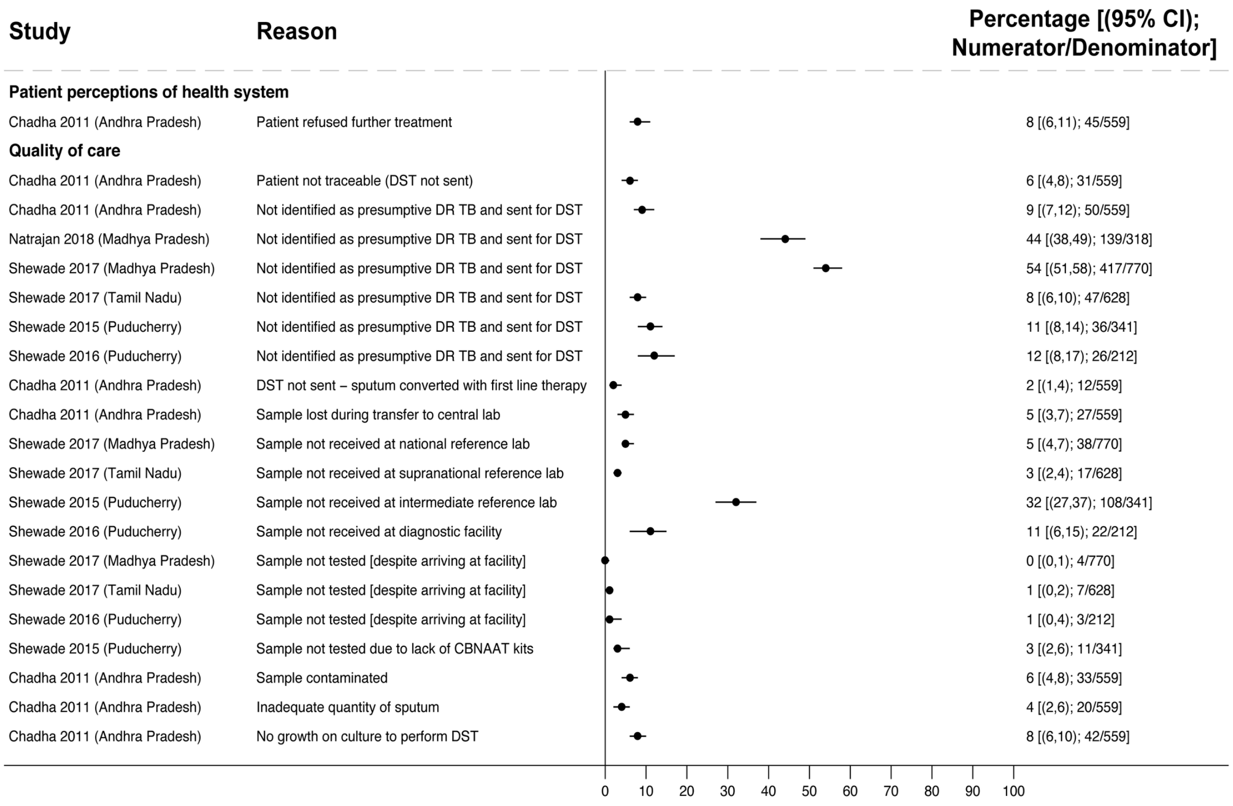

Fig G. Reasons for NAAT or mycobacterial culture non-completion (Gap 2). All studies reported the estimated percentage of all people who were eligible for drug susceptibility testing who experienced a given reason for NAAT or mycobacterial culture noncompletion [17–19,27]. All studies evaluated NAAT non-completion as the outcome except for Chadha et al. 2011 [27], which evaluated non-completion of mycobacterial culture. CBNAAT, cartridge based nucleic acid amplification testing; CI, confidence interval; DR, drug resistant; DST, drug susceptibility testing; TB, tuberculosis.

Gap 3—Barriers contributing to pretreatment loss to follow-up (PTLFU)

Characteristics and quality of the included studies

Across searches for studies spanning January 1, 2000, to August 14, 2023, we screened titles and abstracts of 4,262 unique reports and identified 390 reports for full text review (Fig A in S3 Appendix). Of these, 27 studies met the inclusion criteria of evaluating patients diagnosed with TB disease who did not start on, or get registered in, treatment, which we also refer to as pretreatment loss to follow-up (PTLFU) (Table A above in this S6 Appendix). Note that the definitions varied across studies, with most (though not all [30]) studies defined PTLFU as including patients who left the public sector for private sector TB care. Among included studies, 20 studies reported factors associated with PTLFU (including 5 studies reporting findings from adjusted analyses), and 11 studies reported reasons for PTLFU (Table C in S3 Appendix). Studies were conducted in 11 of India’s states and union territories. In addition, 4 studies collected data from more than 1 state [12,31–33], and 1 study reported findings from a nationally-representative sample of households [32]. 10 studies were conducted in urban areas, 7 in rural areas, and 10 in both.

All studies involved a high-quality (i.e., random or comprehensive) sampling strategy. 11 studies were low quality with regard to sample size [20,21,28,34–41]. 14 studies did not report the time frame of research fieldwork while 3 studies assessed outcomes >3 months after presentation (low quality). 3 studies assessed outcomes 1-3 months after presentation (medium quality). 7 studies relied on self-reported outcomes by the government TB program,

without verification through patient tracking by the research team (low quality) (Table C in S3 Appendix).

20 studies evaluated PTLFU in adults with confirmed or presumed drug-susceptible TB. 6 studies evaluated adults with drug-resistant TB, while 1 study evaluated children with drug-susceptible or drug-resistant TB (Table C in S3 Appendix). We present findings separately for adults with drug-susceptible TB, adults with drug-resistant TB, and children with TB.

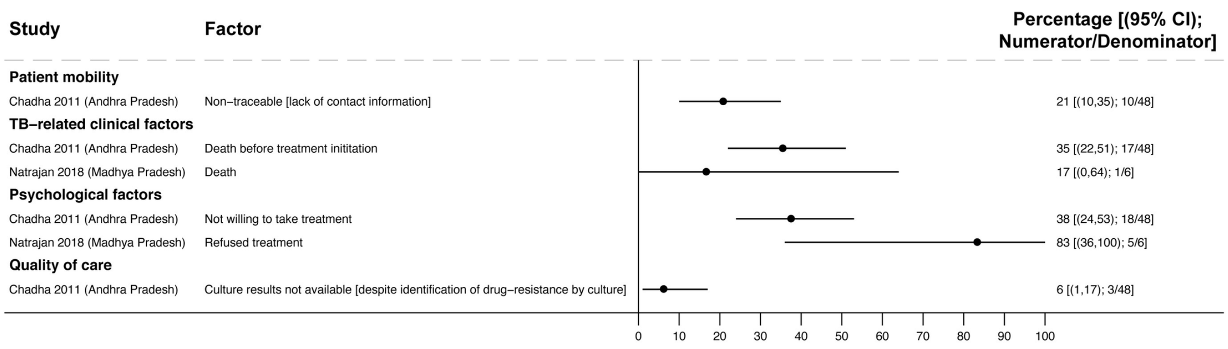

Fig H. Reasons for pretreatment loss to follow-up among people with drug-resistant tuberculosis (Gap 3). Both studies described the estimated percentage of individuals interviewed who reported a given reason for not starting on treatment for drug-resistant TB [27,28]. CI, confidence interval; TB, tuberculosis.

Gap 4—Barriers to treatment success among people who start TB treatment

Characteristics and quality of the included studies

Across searches spanning January 1, 2000, to August 14, 2023, we screened titles and abstracts of 4,262 unique reports and identified 390 reports that underwent full text review, of which 144 met Gap 4 inclusion criteria (Fig A in S4 Appendix and Table A above in this S6 Appendix).

As such, 144 articles were included in the Gap 4 analysis. Of these, 135 presented findings on factors associated with unfavorable treatment outcomes (including 71 reporting adjusted analyses), and 18 presented findings on reasons for unfavorable treatment outcomes (Table C in S4 Appendix). Studies were conducted in 25 of India's states and union territories. In addition, 18 studies collected data from multiple states [2,12,31,42–56], including 1 study comprising a nationally-representative sample from India's 2006 TB register [46]. 27 studies were conducted in rural areas, 74 in urban areas, and 36 in both. Except for 7 studies [53,57–62] where TB was treated in the private sector, all other studies evaluated patients in the public sector (Table C in S4 Appendix).

All included studies involved high-quality (i.e., random or comprehensive) sampling. 37 were low quality with regard to sample size. 59 assessed exposures and outcomes retrospectively from medical records without data collection from TB patients (low quality) (Table C in S4 Appendix).

Given the large number of studies identified, we present findings separately for subpopulations of people with TB: people with drug-susceptible TB (including people with new TB [n=34], people with a previous TB treatment history [n=19], and people with either new or a previous TB treatment history [n=62]), people with drug-resistant TB (n=25), people with human immunodeficiency virus (HIV) being treated for TB (n=7), and children with TB (n=4). (Table C in S4 Appendix).

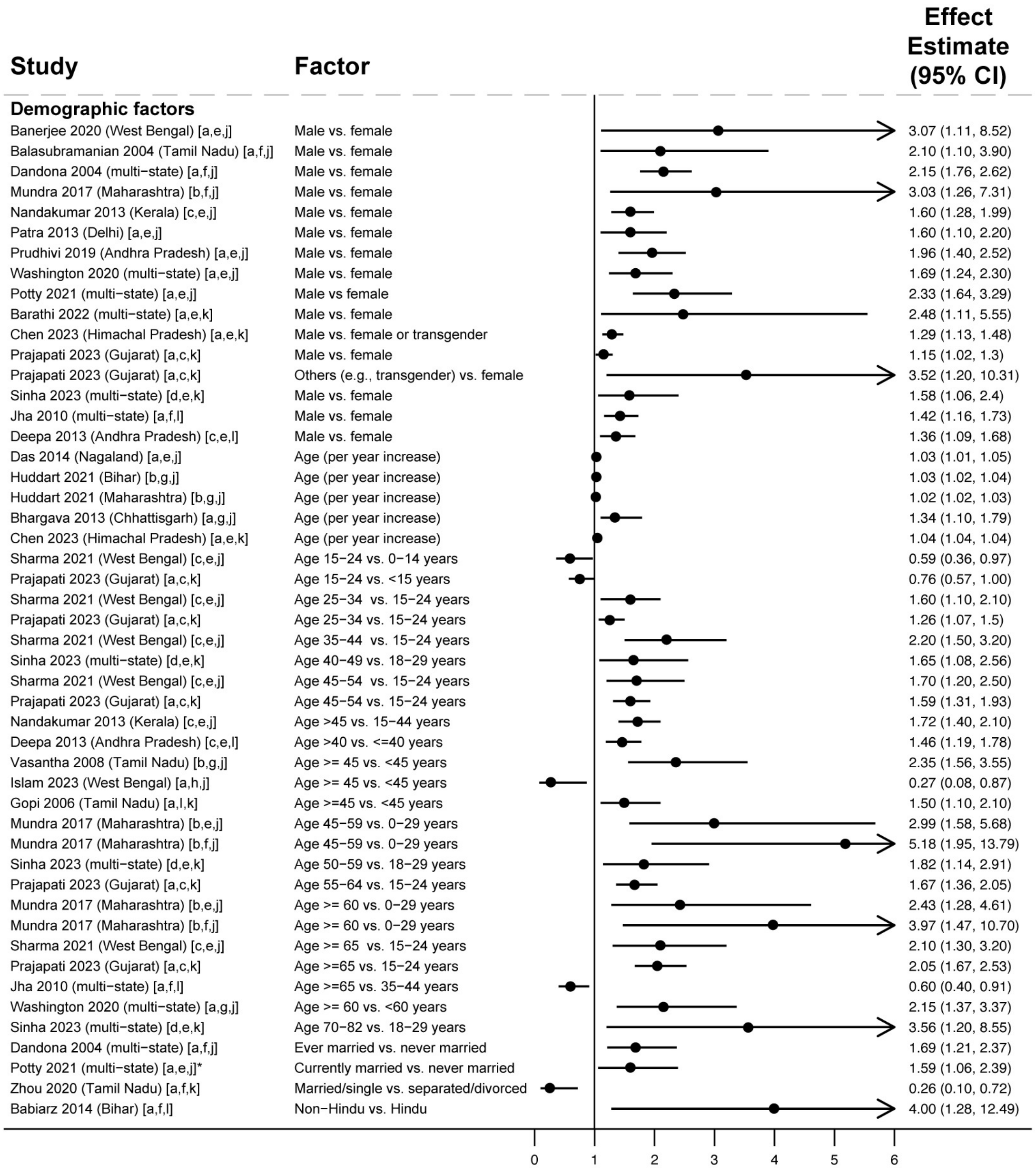

Fig I. Demographic factors contributing to unfavorable treatment outcomes in people with drug-susceptible TB (Gap 4). All studies used multivariable regression and report adjusted effect estimates [29,45,46,49,55,59,63–81]. Estimates greater than 1 represent increased adjusted odds of unfavorable outcomes; estimates less than 1 represent decreased adjusted odds of unfavorable outcomes. Arrowheads means that the upper limits of the CI extends beyond the end of x-axis. Study labels indicate effect estimates are: [a] adjusted odds ratios; [b] adjusted hazard ratios; [c] adjusted relative risk ratios; [d] adjusted incidence rate ratios. Study or variable labels indicate outcomes are: [e] any unfavorable treatment outcome; [f] loss to follow-up; [g] death; [h] medication nonadherence; [i] treatment failure. Study or variable labels indicate subpopulations are: [j] peope with new or previously treated TB; [k] people with new TB only; [l] people with prior TB treatment history only. Only statistically significant findings are presented. Some studies in the review with adjusted analyses reported non-significant findings for sex [49,54,55,64,65,74–76,78–90], age [29,45,46,49,54,55,61,65,68,72,81,83,85–87,90–92], marital status [49,69,85,87], and religion [49,81,85]. CI, confidence interval.

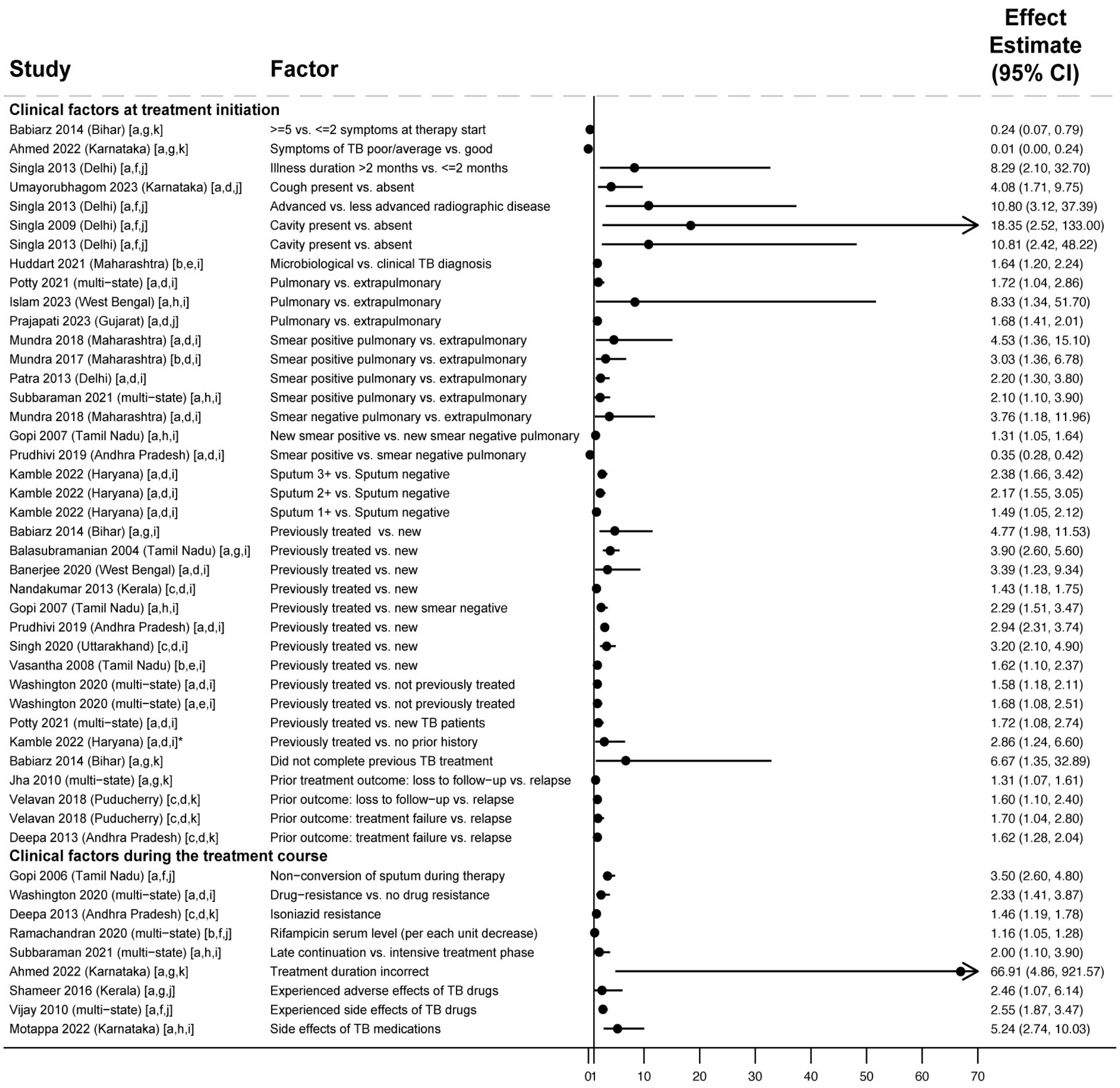

Fig J. TB clinical factors contributing to unfavorable treatment outcomes in people with drug-susceptible TB (Gap 4). All studies used multivariable regression and report adjusted effect estimates [44,46,49,52,54,63,65–68,73,75,78,80–83,88,93–104]. Estimates greater than 1 represent increased adjusted odds of unfavorable outcomes; estimates less than 1 represent decreased adjusted odds of unfavorable outcomes. The arrowhead means that the upper and lower limits of CI extend beyond the end of x-axis. Study or variable labels indicate effect estimates are: [a] adjusted odds ratio; [b] adjusted hazard ratio; [c] adjusted relative risk ratio. Study or variable labels indicate outcomes are: [d] any unfavorable treatment outcome; [e] death; [f] treatment failure; [g] loss to follow-up; [h] medication non-adherence. Study or variable labels indicate patient populations are: [i] multiple tuberculosis patient populations; [j] new TB patients; [k] patients with prior TB treatment history. Only statistically significant findings are presented. Some studies in the review with adjusted analyses reported non-significant findings for number of TB symptoms at treatment initiation [80,81], increased duration of illness [81], cavitary disease, smear positive pulmonary disease [49,63,65–67,71,75,76,82,88,89], smear grade [55,66,80,101,105], non-conversion of sputum [106], TB drug levels [102], TB drug side effects [90,98], drug-resistant TB [43], and previous TB treatment history [46,54,61,65,74–76,78,81,84,86–91]. CI, confidence interval; TB, tuberculosis.

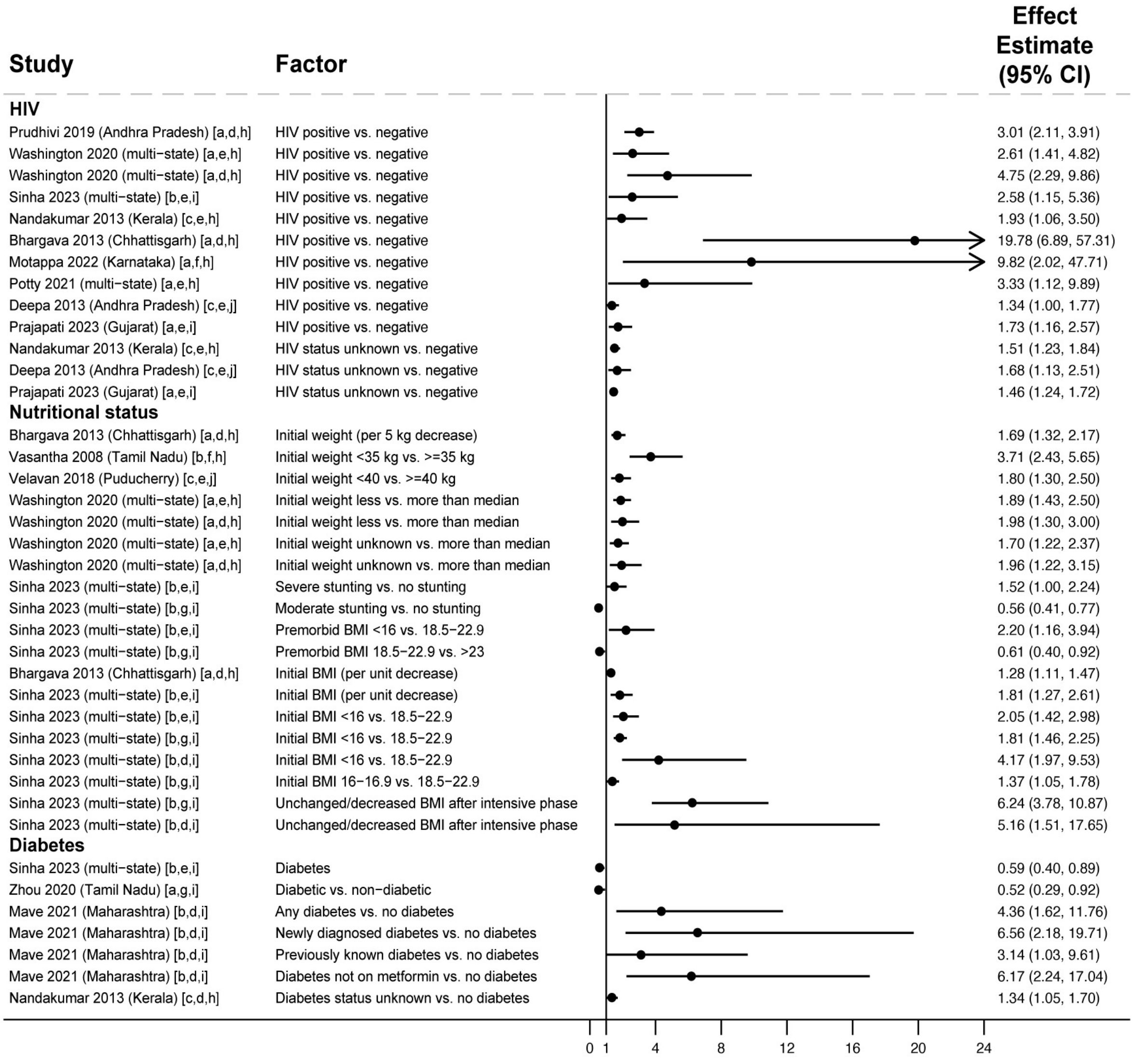

Fig K. Other clinical factors associated with unfavorable treatment outcomes in people with drug-susceptible TB (Gap 4). All studies used multivariable regression and report adjusted effect estimates [45,49,52,55,66,68,73,78,88,94,104,105,107]. Estimates greater than 1 represent increased adjusted odds of unfavorable outcomes; estimates less than 1 represent decreased adjusted odds of unfavorable outcomes. The arrowhead means that the upper and lower limits of CI extend beyond the end of x-axis. Study or variable labels indicate effect estimates are: [a] adjusted odds ratio; [b] adjusted incidence rate ratio; [c] adjusted relative risk ratio. Study or variable labels indicate outcomes are: [d] death; [e] any unfavorable treatment outcome; [f] medication non-adherence [g] loss to follow-up. Study or variable labels indicate patient populations are: [h] people with new TB or a prior TB treatment history; [i] people with new TB; [j] peoople with a prior TB treatment history. Only statistically significant findings are presented. Some studies in the review with adjusted analyses reported non-significant findings for HIV status [43,54,55,65,69,73,86], low BMI [90], and diabetes [49,65,86,90]. CI, confidence interval; BMI, body mass index; HIV, human immunodeficiency virus; TB, tuberculosis.

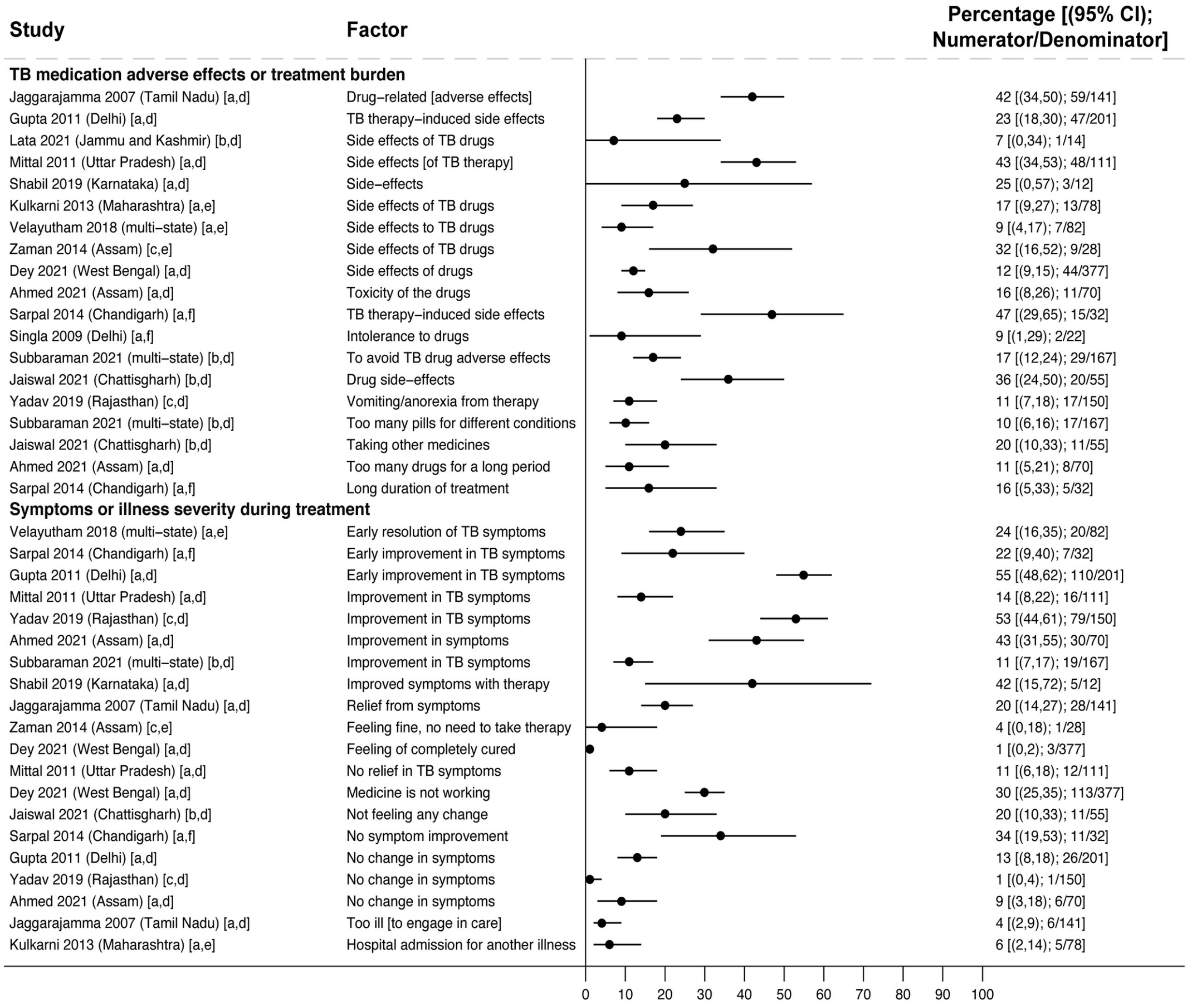

Fig L. Clinical reasons contributing to unfavorable treatment outcomes in people with drug-susceptible TB (Gap 4). This figure cites the following papers: [43,54,62,85,99,108–117]. Estimates represent the percentage of individuals interviewed who reported a given reason for experiencing unfavorable outcomes. Study labels indicate outcomes are: [a] loss to follow-up; [b] medication non-adherence [c] any interruption (defined as a combination of medication non-adherence and loss to follow-up). Study labels indicate subpopulations are: [d] people with new TB or a prior TB treatment history; [e] people with new TB; [f] people with a prior TB treatment history. CI, confidence interval; TB, tuberculosis.

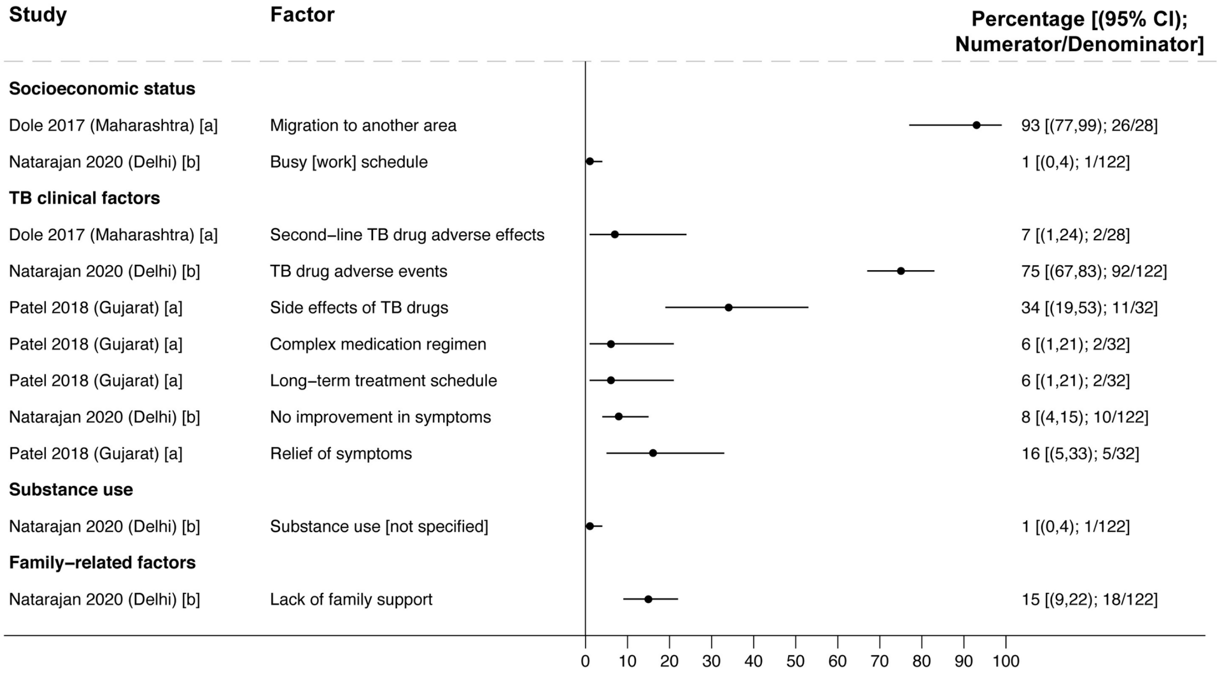

Fig M. Reasons reported by people with rifampin-resistant or multidrug-resistant (MDR) TB for experiencing unfavorable outcomes during treatment (Gap 4). This figure cites the following papers: [118–120]. Estimates represent the percentage of individuals interviewed who reported a given reason for experiencing unfavorable outcomes. Study labels indicate outcomes are: [a] loss to follow-up; [b] any interruption (defined as a combination of medication non-adherence and loss to follow-up). CI, confidence interval; TB, tuberculosis.

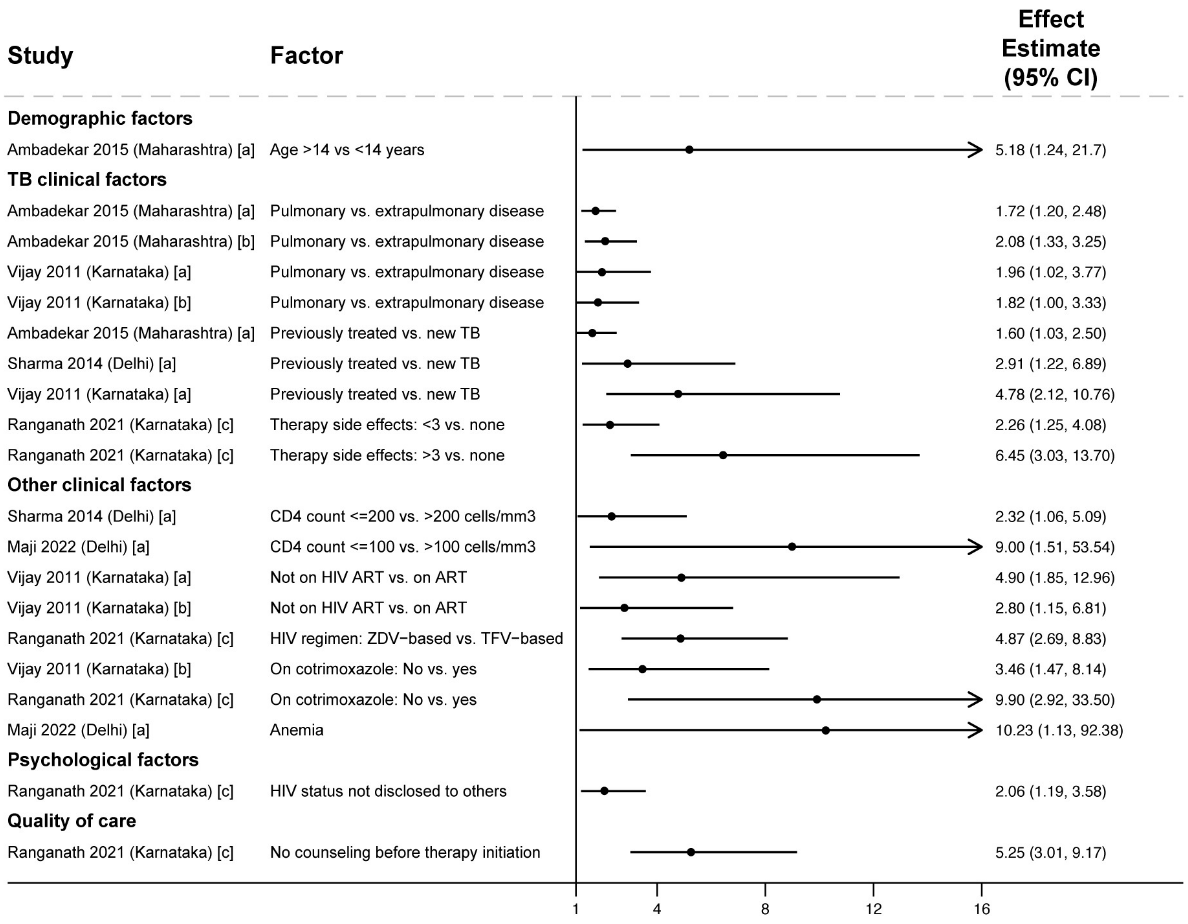

Fig N. Factors associated with unfavorable TB treatment outcomes among people with HIV (Gap 4). All studies used multivariable regression and report adjusted odds ratios [121–125]. Ratios greater than 1 represent increased odds of unfavorable outcomes; ratios less than 1 represent decreased odds of unfavorable outcomes. The arrowhead means that the upper and limit of the CI extends beyond the end of the x-axis. Study or variable labels indicate outcomes are: [a] any unfavorable outcome (loss to follow-up, treatment failure, or death); [b] death; or [c] non-adherence. Only statistically significant findings are presented. Some studies in the review with adjusted analyses reported non-significant findings for age [123], previous TB treatment history [121,123], status of HIV ART [122] and of cotrimoxazole therapy [126]. ART, antiretroviral therapy; BMI, body mass index; CD4, clusters of differentiation; CI, confidence interval; HIV, human immunodeficiency virus; TB, tuberculosis; TFV, tenofovir; ZDV, zidovudine.

Gap 5—Barriers to achieving recurrence-free survival after TB treatment

Characteristics and quality of the included studies

Across searches spanning January 1, 2000, to August 14, 2023, we screened titles and abstracts of 4,660 unique reports and identified 117 for full text review, of which 19 met inclusion criteria (Fig A in S5 Appendix and Table A above in this S6 Appendix). Of these, 14 studies reported findings on TB recurrence as a single outcome or part of a composite outcome, and 5 studies reported findings on post-treatment mortality as a single outcome or part of a composite outcome with on-treatment mortality (Table C in S5 Appendix). Notably, 14 studies reported findings from adjusted regression analyses. Studies were conducted in 9 of India's states and union territories, including Bihar and Madhya Pradesh, 2 of India's high-population and low-income states. 5 studies were conducted in multiple states. 4 studies were conducted in rural areas, 10 in urban areas, and 5 in both (Table C in S5 Appendix).

All studies involved a high-quality random or comprehensive sampling (Table C in S5 Appendix). 4 studies were medium quality with regard to sample size and distribution, because they were conducted at a single facility [127–130]. 7 studies did not follow patients prospectively and relied on a lower-quality passive surveillance for assessing post-treatment outcomes [59,75,128,131–134], while 2 studies did not report the surveillance approach [135,136]. For studies evaluating TB recurrence, 5 studies had at least 1 post-treatment follow-up visit with microbiological testing to identify recurrence [42,43,55,128,137] (high quality), while 6 studies performed microbiological testing only for patients with persistent symptoms

[48,107,127,130,131,138] (medium quality). 2 studies evaluated TB recurrence through self-enrollment of patients in treatment [59,75] (low quality), and 1 did not clearly report the approach to detecting recurrence [129] (low quality).

All studies evaluated adult patients. 5 studies included adolescents (usually >14 years of age) [128,129,134–136] and 1 study included younger children [131]. Except for 2 studies of the private sector [59,75], studies evaluated patients in the public sector. (Table C in S5 Appendix). We present findings separately for studies evaluating TB recurrence (as a single outcome or part of a composite outcome) and post-treatment mortality (as a single outcome or part of a composite outcome along with on-treatment mortality).

Summary of common findings across care cascade gaps

We summarize factors that were statistically significantly associated with unfavorable outcomes for more than 1 care cascade gap, and the number of studies contributing findings, in Table B below. We summarize reasons for unfavorable outcomes that were reported across more than 1 gap, and the number of studies contributing findings, in Table C below.

Table B. Statistically significant factors associated with unfavorable outcomes across multiple TB care cascade gaps in adjusted analyses.

Numbers indicate the number of studies contributing data to each factor for a given gap. Footnotes identify discordant (protective) findings for some factors.

**Subcategory and factors**<br>N (total studies with findings across all gaps)	**Gap 1**<br>*(did not seek care for TB symptoms)*	**Gap 2**<br>*(did not complete the diagnostic workup)*	**Gap 3**<br>*(did not start TB treatment)*	**Gap 4**<br>*(did not complete TB treatment)*	**Gap 5**<br>*(post-treatment TB recurrence or death)*
**Demographic factors**					
Male sex (N=29)	**3** [5,139][d,e]	**2** [8,9][i]		**20** [11,12,46,47,49,52,55,56,63,65–68,73,94,140–144]	**4** [43,75,132,134]
Older age (N=30)	**1** [139][f]	**4** [9,12,19,22][j]	**2** [30,32]	**19** [46,49,55,58,59,61,65,66,73,76,78,80,94,123,140,141, 143,145,146]	**4** [59,132,134,135]
**Socioeconomic status**					
Lower socioeconomic class[a] (N=5)	**2** [4,5]	**1** [16]	**1** [32]	**1** [147]	
Lower education (N=8)	**1** [3][g]		**1** [32]	**5** [44,49,81,84,100]	**1** [134]
Unemployed or daily wages (N=7)	**2** [2,139]			**3** [11,54,84][l]	**2** [132,134]
Rural location (N=3)	**1** [148]		**1** [30]		**1** [59]
**TB clinical factors**					
Lower symptom severity[b] (N=4)	**2** [7,139]	**1** [12]		**1** [81]	
Previous TB history (N=23)	**1** [7][h]	**2** [9,19][k]	**2** [10,30]	**17** [11,47,49,52,63,66,68,78,81,82,89,100,121–123,149,150]	**1** [135]
Drug-resistant TB (N=5)		**1** [23]		**3** [49,73,144]	**1** [137]
Medication non-adherence (N=9)				**7** [44,47,52,56,97,99,145]	**2** [75,137]
**Other clinical factors**					
Undernutrition (N=16)				**12** [47,49,55,76,78,88,141,143,145,147,149,151]	**4** [48,55,107,132]
HIV (N=12)		**1** [10]		**11** [49,55,66,68,69,73,76,94,104,144,151]	
**Substance use**					
Alcohol use (N=21)	**1** [139]	**1** [8]		**15** [11,44,45,49,54,68,78,80,84,85,103,144,151–153]	**4** [48,107,135,136]
Smoking (N=13)	**1** [139]		**1** [41]	**6** [55,68,85,98,151,152]	**5** [129,134–137]
**Knowledge-related factors**					
Low TB-related knowledge (N=7)	**3** [3,5,139]			**4** [44,98,154,155]	
**Health system factors**					
Healthcare accessibility (N=6)		**1** [16]		**5** [81,98,100,152,154]	
Evaluation at private facility (N=5)		**1** [10]	**2** [10,32]	**2** [46,94][m]	
Negative provider interactions (N=6)		**2** [12,16]		**4** [44,81,98,156]	
Negative views of health system[c] (N=5)	**1** [5]			**4** [12,44,83,155]	

[a]Includes lower household income; [b]includes lower symptom duration; [c]includes distrust of the health system; [d][5] found male sex was associated with lower care seeking in 2 Indian states and is reported here as 2 studies; [e][139] found male sex was associated with lower care-seeking in the unadjusted analysis, and, while the direction of this association switched in the multivariable analysis, this was after adjusting for alcohol use, which was only reported among men; [f][139] found increasing age was associated with higher care seeking; [g][3] found lower education was associated with higher care seeking; [h][7] found previously treated individuals were more likely to seek care; [i][8] found men were more likely to pursue further diagnostic workup; [j][9,22] found older people were more likely to pursue further diagnostic workup; [k][9] found previously treated people were more likely to pursue further diagnostic workup; [l][11,84] found unemployed individuals were more likely to have favorable treatment outcomes; [m][94] found evaluation at private facility were more likely to have favorable treatment outcomes. HIV, human immunodeficiency virus; TB, tuberculosis.

Table C. Reasons reported by people with TB or TB symptoms for not seeking care or being lost from care across multiple care cascade gaps. Numbers indicate studies contributing data to each reason for a given gap. Only findings in which 15% or more of people reported a given reason are presented.

**Subcategory and reasons**<br>N (total studies with findings across all gaps)	**Gap 1**<br>*(did not seek care for TB symptoms)*	**Gap 2**<br>*(did not complete the diagnostic workup)*	**Gap 3**<br>*(did not start TB treatment)*	**Gap 4**<br>*(did not complete TB treatment)*
**Socioeconomic status**				
Work constraints or pressure (N=10)	**4** [5,157–159]	**1** [26]	**1** [34]	**4** [62,85,111,112]
Patient mobility (N=9)			**1** [34]	**8** [43,54,62,99,109,112]
**TB clinical factors**				
Symptoms not severe or resolved early (N=20)	**9** [1,5,6,157–161][a]	**2** [12,25]		**9**[43,54,62,85,108,110,112,115,116]
High severity of TB illness[b] (N=5)		**1** [24]	**4** [27,30,33,162]	
Adverse effects of TB therapy[c] (N=13)			**1** [38]	**12**[54,62,85,108,110–113,115,117,119,120]
**Psychological factors**				
Stigma related to TB (N=4)			**2** [12,163]	**2** [54,85]
**Substance use**				
Alcohol use (N=2)			**1** [163]	**1** [43]
**Health system factors**				
Long distance to health facility (N=4)	**2** [6,157]		**1** [39]	**1** [112]
Negative views of health system (N=3)			**2** [12,38]	**1** [85]
Negative health provider interactions (N=2)		**1** [12]		**1** [111]
Quality of care: Patient not told or referred for next steps in care or patient untrackable because of poor recording of contact information (N=9)		**4** [19,20,26,28]	**5** [27,30,33,162,164]	

[a][5] found 'symptoms not severe' as 1 of the key reasons given by chest symptomatics for not seeking care in 2 Indian states and is reported here as 2 studies
[b]includes disease-related barriers and early deaths; [c]includes experiences of adverse effects of prior TB therapy as contributing to pretreatment loss to follow-up. TB, tuberculosis.

References

1. Karanjekar V, Gujarati V, Lokare P. Sociodemographic factors associated with health seeking behavior of chest symptomatics in urban slums of Aurangabad city, India. International Journal of Basic and Applied Medical Sciences. 2014;4: 173–179.

2. Shewade HD, Gupta V, Satyanarayana S, Pandey P, Bajpai UN, Tripathy JP, et al. Patient characteristics, health seeking and delays among new sputum smear positive TB patients identified through active case finding when compared to passive case finding in India. PLoS One. 2019;14: e0213345. doi:10.1371/journal.pone.0213345

3. Karnataka Health Promotion Trust (KHPT). Knowledge about TB and Health Seeking Behaviour among Adult Chest Symptomatic (CS) Population Living in Urban Slums of Hyderabad City. A Baseline Study Report: 2016-17. Karnataka Health Promotion Trust (KHPT) Tuberculosis Health Action Learning Initiative (THALI); 2018.

4. Karnataka Health Promotion Trust (KHPT). Knowledge about TB and Health Seeking Behaviour among Adult Chest Symptomatic (CS) Population Living in Urban Slums of Bengaluru City. A Baseline Study Report: 2016-17. Karnataka Health Promotion Trust (KHPT) Tuberculosis Health Action Learning Initiative (THALI); 2018.

5. George O, Sharma V, Sinha A, Bastian S, Santha T. Knowledge and behaviour of chest symptomatics in urban slum populations of two states in India towards care-seeking. Indian Journal of Tuberculosis. 2013;60: 95–106.

6. Shriraam V, Srihari R, Gayathri T, Murali L. Active case finding for Tuberculosis among migrant brick kiln workers in South India. Indian J Tuberc. 2020;67: 38–42. doi:10.1016/j.ijtb.2019.09.003

7. Fochsen G, Deshpande K, Diwan V, Mishra A, Diwan VK, Thorson A. Health care seeking among individuals with cough and tuberculosis: a population-based study from rural India. Int J Tuberc Lung Dis. 2006;10: 995–1000.

8. Dey A, Thekkur P, Ghosh A, Dasgupta T, Bandopadhyay S, Lahiri A, et al. Active Case Finding for Tuberculosis through TOUCH Agents in Selected High TB Burden Wards of Kolkata, India: A Mixed Methods Study on Outcomes and Implementation Challenges. Trop Med Infect Dis. 2019;4: 134. doi:10.3390/tropicalmed4040134

9. Garg T, Gupta V, Sen D, Verma M, Brouwer M, Mishra R, et al. Prediagnostic loss to follow-up in an active case finding tuberculosis programme: a mixed-methods study from rural Bihar, India. BMJ Open. 2020;10: e033706. doi:10.1136/bmjopen-2019-033706

10. Ismail IM, Kibballi Madhukeshwar A, Naik PR, Nayarmoole BM, Satyanarayana S. Magnitude and Reasons for Gaps in Tuberculosis Diagnostic Testing and Treatment

Initiation: An Operational Research Study from Dakshina Kannada, South India. J Epidemiol Glob Health. 2020;10: 326–336. doi:10.2991/jegh.k.200516.001

11. Balasubramanian R, Garg R, Santha T, Gopi PG, Subramani R, Chandrasekaran V, et al. Gender disparities in tuberculosis: report from a rural DOTS programme in south India. Int J Tuberc Lung Dis. 2004;8: 323–332.

12. Dandona R, Dandona L, Mishra A, Dhingra S, Venkatagopalakrishna K, Chauhan LS. Utilization of and barriers to public sector tuberculosis services in India. Natl Med J India. 2004;17: 292–299.

13. Das M, Pasupuleti D, Rao S, Sloan S, Mansoor H, Kalon S, et al. GeneXpert and Community Health Workers Supported Patient Tracing for Tuberculosis Diagnosis in Conflict-Affected Border Areas in India. Trop Med Infect Dis. 2019;5: 1. doi:10.3390/tropicalmed5010001

14. Tripathy JP, Srinath S, Naidoo P, Ananthakrishnan R, Bhaskar R. Is physical access an impediment to tuberculosis diagnosis and treatment? A study from a rural district in North India. Public Health Action. 2013;3: 235–239. doi:10.5588/pha.13.0044

15. Kanakaraju M, Nagaraja SB, Satyanarayana S, Babu YR, Madhukeshwar AK, Narasimhaiah S. Chest Radiography and Xpert MTB/RIF® Testing in Persons with Presumptive Pulmonary TB: Gaps and Challenges from a District in Karnataka, India. Tuberc Res Treat. 2020;2020: 5632810. doi:10.1155/2020/5632810

16. Sarkar J, Murhekar MV. Factors associated with low utilization of x-ray facilities among the sputum negative chest symptomatics in Jalpaiguri district (West Bengal) 2009. Indian J Tuberc. 2011;58: 208–211.

17. Shewade HD, Govindarajan S, Sharath BN, Tripathy JP, Chinnakali P, Kumar AMV, et al. MDR-TB screening in a setting with molecular diagnostic techniques: who got tested, who didn't and why? Public Health Action. 2015;5: 132–139. doi:10.5588/pha.14.0098

18. Shewade HD, Govindarajan S, Thekkur P, Palanivel C, Muthaiah M, Kumar AMV, et al. MDR-TB in Puducherry, India: reduction in attrition and turnaround time in the diagnosis and treatment pathway. Public Health Action. 2016;6: 242–246. doi:10.5588/pha.16.0075

19. Shewade HD, Kokane AM, Singh AR, Verma M, Parmar M, Chauhan A, et al. High pre-diagnosis attrition among patients with presumptive MDR-TB: an operational research from Bhopal district, India. BMC Health Serv Res. 2017;17: 249. doi:10.1186/s12913-017-2191-6

20. Shewade HD, Nair D, Klinton JS, Parmar M, Lavanya J, Murali L, et al. Low pre-diagnosis attrition but high pre-treatment attrition among patients with MDR-TB: An operational research from Chennai, India. J Epidemiol Glob Health. 2017;7: 227–233. doi:10.1016/j.jegh.2017.07.001

21. Singla N, Satyanarayana S, Sachdeva KS, Van den Bergh R, Reid T, Tayler-Smith K, et al. Impact of introducing the line probe assay on time to treatment initiation of MDR-TB in Delhi, India. PLoS One. 2014;9: e102989. doi:10.1371/journal.pone.0102989

22. Ranganath R, Shewade HD, Bahadur AK, Naik V, Nagaraja SB, Kumar AMV, et al. Uptake of universal drug susceptibility testing among people with TB in a south Indian district: How are we faring? Trans R Soc Trop Med Hyg. 2022;116: 43–49. doi:10.1093/trstmh/trab051

23. Shankar S U, Kumar AMV, Venkateshmurthy NS, Nair D, Kingsbury R, R P, et al. Implementation of the new integrated algorithm for diagnosis of drug-resistant tuberculosis in Karnataka State, India: How well are we doing? PLoS One. 2021;16: e0244785. doi:10.1371/journal.pone.0244785

24. Chandrasekaran V, Ramachandran R, Cunningham J, Balasubramanian R, Thomas A, Sudha G, et al. Factors leading to tuberculosis diagnostic drop-out and delayed treatment initiation in Chennai, India. Int J Tuberc Lung Dis. 2005;9: S172.

25. Chadha VK, Praseeja P, Hemanthkumar NK, Shivshankara BA, Sharada MA, Nagendra N, et al. Implementation efficiency of a diagnostic algorithm in sputum smear-negative presumptive tuberculosis patients. Int J Tuberc Lung Dis. 2014;18: 1237–1242. doi:10.5588/ijtld.14.0218

26. Thomas A, Gopi PG, Santha T, Jaggarajamma K, Charles N, Prabhakaran E, et al. Course of action taken by smear negative chest symptomatics: A report from a rural area in South India. Indian Journal of Tuberculosis. 2006;53: 4–6.

27. Chadha SS, Sharath BN, Reddy K, Jaju J, Vishnu PH, Rao S, et al. Operational challenges in diagnosing multi-drug resistant TB and initiating treatment in Andhra Pradesh, India. PLoS One. 2011;6: e26659. doi:10.1371/journal.pone.0026659

28. Natrajan S, Singh AR, Shewade HD, Verma M, Bali S. Pre-diagnosis attrition in patients with presumptive MDR-TB in Bhopal, India, 2015: a follow-up study. Public Health Action. 2018;8: 95–96. doi:10.5588/pha.18.0015

29. Dandona R, Dandona L, Mishra A, Dhingra S, Venkatagopalakrishna K, Chauhan LS. Utilization of and barriers to public sector tuberculosis services in India. Natl Med J India. 2004;17: 292–299.

30. Thomas BE, Subbaraman R, Sellappan S, Suresh C, Lavanya J, Lincy S, et al. Pretreatment loss to follow-up of tuberculosis patients in Chennai, India: a cohort study with implications for health systems strengthening. BMC Infect Dis. 2018;18: 142. doi:10.1186/s12879-018-3039-3

31. Raizada N, Khaparde SD, Salhotra VS, Rao R, Kalra A, Swaminathan S, et al. Accelerating access to quality TB care for pediatric TB cases through better diagnostic strategy in four major cities of India. PLoS One. 2018;13: e0193194. doi:10.1371/journal.pone.0193194

32. Pardeshi G, Deluca A, Agarwal S, Kishore J. Tuberculosis patients not covered by treatment in public health services: findings from India's National Family Health Survey 2015-16. Trop Med Int Health. 2018;23: 886–895. doi:10.1111/tmi.13086

33. Sai Babu B, Satyanarayana AVV, Venkateshwaralu G, Ramakrishna U, Vikram P, Sahu S, et al. Initial default among diagnosed sputum smear-positive pulmonary tuberculosis patients in Andhra Pradesh, India. Int J Tuberc Lung Dis. 2008;12: 1055–1058.

34. Mandal A, Basu M, Das P, Mukherjee S, Das S, Roy N. Magnitude and reasons of initial default among new sputum positive cases of pulmonary tuberculosis under RNTCP in a district of West Bengal, India. South East Asia Journal of Public Health. 2014;4: 41–47. doi:10.3329/seajph.v4i1.21839

35. Jain S, Varudkar HG, Julka A, Singapurwala M, Khosla S, Shah B. Socio-economical and Clinico-Radiological Profile of 474 MDR TB Cases of a Rural Medical College. J Assoc Physicians India. 2018;66: 14–18.

36. Khandekar J, Acharya AS, R TH, Sharma A. Do patients with tuberculosis referred from a tertiary care referral centre reach their peripheral health institution? Natl Med J India. 2013;26: 332–334.

37. Kumar D, Goel C, Bansal AK, Bhardwaj AK. Delineating the factors associated with recurrence of tuberculosis in programmatic settings of rural health block, Himachal Pradesh, India. Indian J Tuberc. 2018;65: 303–307. doi:10.1016/j.ijtb.2018.07.001

38. Mehra D, Kaushik RM, Kaushik R, Rawat J, Kakkar R. Initial default among sputum-positive pulmonary TB patients at a referral hospital in Uttarakhand, India. Trans R Soc Trop Med Hyg. 2013;107: 558–565. doi:10.1093/trstmh/trt065

39. Rawat J, Biswas D, Sindhwani G, Kesharwani V, Masih V, Chauhan BS. Diagnostic defaulters: an overlooked aspect in the Indian Revised National Tuberculosis Control Program. J Infect Dev Ctries. 2012;6: 20–22. doi:10.3855/jidc.1895

40. Selvaraju S, Malaisamy M, Dolla CK, Murali L, Karikalan N, Saravanan B, et al. Application of mobile phone technology as intervention for the management of tuberculosis patients diagnosed through community survey. Indian J Med Res. 2022;155: 301–305. doi:10.4103/ijmr.IJMR_75_20

41. Majella MG, Thekkur P, Kumar AM, Chinnakali P, Saka VK, Roy G. Effect of mobile voice calls on treatment initiation among patients diagnosed with tuberculosis in a tertiary care hospital of Puducherry: A randomized controlled trial. J Postgrad Med. 2021;67: 205–212. doi:10.4103/jpgm.JPGM_1105_20

42. Ramachandran G, Chandrasekaran P, Gaikwad S, Agibothu Kupparam HK, Thiruvengadam K, Gupte N, et al. Subtherapeutic Rifampicin Concentration Is Associated With Unfavorable

Tuberculosis Treatment Outcomes. Clinical Infectious Diseases. 2020;70: 1463–1470. doi:10.1093/cid/ciz380

43. Velayutham B, Chadha VK, Singla N, Narang P, Gangadhar Rao V, Nair S, et al. Recurrence of tuberculosis among newly diagnosed sputum positive pulmonary tuberculosis patients treated under the Revised National Tuberculosis Control Programme, India: A multi-centric prospective study. PLoS One. 2018;13: e0200150. doi:10.1371/journal.pone.0200150

44. Vijay S, Kumar P, Chauhan LS, Vollepore BH, Kizhakkethil UP, Rao SG. Risk Factors Associated with Default among New Smear Positive TB Patients Treated Under DOTS in India. PLoS ONE. 2010;5: e10043. doi:10.1371/journal.pone.0010043

45. Zhou TJ, Lakshminarayanan S, Sarkar S, Knudsen S, Horsburgh CR, Muthaiah M, et al. Predictors of Loss to Follow-Up among Men with Tuberculosis in Puducherry and Tamil Nadu, India. The American Journal of Tropical Medicine and Hygiene. 2020;103: 1050–1056. doi:10.4269/ajtmh.19-0415

46. Jha UM, Satyanarayana S, Dewan PK, Chadha S, Wares F, Sahu S, et al. Risk Factors for Treatment Default among Re-Treatment Tuberculosis Patients in India, 2006. PLoS ONE. 2010;5: e8873. doi:10.1371/journal.pone.0008873

47. Parmar MM, Sachdeva KS, Dewan PK, Rade K, Nair SA, Pant R, et al. Unacceptable treatment outcomes and associated factors among India's initial cohorts of multidrug-resistant tuberculosis (MDR-TB) patients under the revised national TB control programme (2007–2011): Evidence leading to policy enhancement. PLoS ONE. 2018;13: e0193903. doi:10.1371/journal.pone.0193903

48. Cox SR, Gupte AN, Thomas B, Gaikwad S, Mave V, Padmapriyadarsini C, et al. Unhealthy alcohol use independently associated with unfavorable TB treatment outcomes among Indian men. Int J Tuberc Lung Dis. 2021;25: 182–190. doi:10.5588/ijtld.20.0778

49. Washington R, Potty RS, Rajesham A, Seenappa T, Singarajipura A, Swamickan R, et al. Is a differentiated care model needed for patients with TB? A cohort analysis of risk factors contributing to unfavourable outcomes among TB patients in two states in South India. BMC Public Health. 2020;20: 1158. doi:10.1186/s12889-020-09257-5

50. B V, V S, V M, R G, N K, S M, et al. Factors influencing treatment outcomes in patients with isoniazid-resistant pulmonary TB. Int J Tuberc Lung Dis. 2022;26: 1033-1040. doi:10.5588/ijtld.21.0701

51. Potty RS, Kumarasamy K, Munjattu JF, Reddy RC, Adepu R, Singarajipura A, et al. Tuberculosis treatment outcomes and patient support groups, southern India. Bull World Health Organ. 2023;101: 28-35A. doi:10.2471/BLT.22.288237

52. Potty RS, Kumarasamy K, Adepu R, Reddy RC, Singarajipura A, Siddappa PB, et al. Community health workers augment the cascade of TB detection to care in urban slums of two metro cities in India. J Glob Health. 2021;11: 04042. doi:10.7189/jogh.11.04042

53. Sodhi R, Penkunas MJ, Pal A. Free drug provision for tuberculosis increases patient follow-ups and successful treatment outcomes in the Indian private sector: a quasi experimental study using propensity score matching. BMC Infect Dis. 2023;23: 421. doi:10.1186/s12879-023-08396-5

54. Subbaraman R, Thomas BE, Kumar JV, Thiruvengadam K, Khandewale A, Kokila S, et al. Understanding Nonadherence to Tuberculosis Medications in India Using Urine Drug Metabolite Testing: A Cohort Study. Open Forum Infect Dis. 2021;8: ofab190. doi:10.1093/ofid/ofab190

55. Sinha P, Ponnuraja C, Gupte N, Prakash Babu S, Cox SR, Sarkar S, et al. Impact of Undernutrition on Tuberculosis Treatment Outcomes in India: A Multicenter, Prospective, Cohort Analysis. Clin Infect Dis. 2023;76: 1483–1491. doi:10.1093/cid/ciac915

56. Barathi A, Krishnamoorthy Y, Sinha P, Horsburgh C, Hochberg N, Johnson E, et al. Effect of treatment adherence on the association between sex and unfavourable treatment outcomes among tuberculosis patients in Puducherry, India: a mediation analysis. J Public Health (Oxf). 2023;45: 304–311. doi:10.1093/pubmed/fdac062

57. Isaakidis P, Varghese B, Mansoor H, Cox HS, Ladomirska J, Saranchuk P, et al. Adverse Events among HIV/MDR-TB Co-Infected Patients Receiving Antiretroviral and Second Line Anti-TB Treatment in Mumbai, India. Wilkinson RJ, editor. PLoS ONE. 2012;7: e40781. doi:10.1371/journal.pone.0040781

58. Saha A, Vaidya PJ, Chavhan VB, Pandey KV, Kate AH, Leuppi JD, et al. Factors affecting outcomes of individualised treatment for drug resistant tuberculosis in an endemic region. Indian J Tuberc. 2019;66: 240–246. doi:10.1016/j.ijtb.2017.04.001

59. Huddart S, Singh M, Jha N, Benedetti A, Pai M. Case fatality and recurrent tuberculosis among patients managed in the private sector: A cohort study in Patna, India. PLOS One. 2021;16: e0249225. doi:10.1371/journal.pone.0249225

60. Secretary Of Jan Swasthya Sahyog, Laux TS, Patil S. Predictors of tuberculosis treatment outcomes among a retrospective cohort in rural, Central India. J Clin Tuberc Other Mycobact Dis. 2018;12: 41–47. doi:10.1016/j.jctube.2018.06.005

61. Sharma V, Thekkur P, Naik PR, Saha BK, Agrawal N, Dinda MK, et al. Treatment success rates among tuberculosis patients notified from the private sector in West Bengal, India. Monaldi Arch Chest Dis. 2021;91. doi:10.4081/monaldi.2021.1555

62. Dey A, Lahiri A, Jha SS, Sharma V, Shanmugam P, Chakrabartty AK. Treatment adherence status of the TB patients notified from private sector and its associated factors: Findings of

a secondary data analysis from West Bengal, India. Indian J Tuberc. 2022;69: 334–340. doi:10.1016/j.ijtb.2021.06.001

63. Banerjee S, Bandyopadhyay K, Taraphdar P, Dasgupta A. Perceived discrimination among tuberculosis patients in an urban area of Kolkata City, India. J Global Infect Dis. 2020;12: 144–148. doi:10.4103/jgid.jgid_146_19

64. Balasubramanian R, Garg R, Santha T, Gopi PG, Subramani R, Chandrasekaran V, et al. Gender disparities in tuberculosis: report from a rural DOTS programme in south India. Int J Tuberc Lung Dis. 2004;8: 323–332.

65. Mundra A, Deshmukh PR, Dawale A. Magnitude and determinants of adverse treatment outcomes among tuberculosis patients registered under Revised National Tuberculosis Control Program in a Tuberculosis Unit, Wardha, Central India: A record-based cohort study. J Epidemiol Glob Health. 2017;7: 111–118. doi:10.1016/j.jegh.2017.02.002

66. Nandakumar K, Duraisamy K, Balakrishnan S, M S, S JS, Sagili KD, et al. Outcome of Tuberculosis Treatment in Patients with Diabetes Mellitus Treated in the Revised National Tuberculosis Control Programme in Malappuram District, Kerala, India. PLoS ONE. 2013;8: e76275. doi:10.1371/journal.pone.0076275

67. Patra S, Lukhmana S, Tayler Smith K, Kannan AT, Satyanarayana S, Enarson DA, et al. Profile and treatment outcomes of elderly patients with tuberculosis in Delhi, India: implications for their management. Transactions of the Royal Society of Tropical Medicine and Hygiene. 2013;107: 763–768. doi:10.1093/trstmh/trt094

68. Prudhivi R, Challa SR, Rao MV B, Veena G V, Rao N B, Manogna Narne H. Assessment of Success Rate of Directly Observed Treatment Short-Course (DOTS) in Tuberculosis Patients of South India. J Young Pharm. 2018;11: 67–72. doi:10.5530/jyp.2019.11.14

69. Potty RS, Kumarasamy K, Adepu R, Reddy RC, Singarajipura A, Siddappa PB, et al. Community health workers augment the cascade of TB detection to care in urban slums of two metro cities in India. J Glob Health. 2021;11: 04042. doi:10.7189/jogh.11.04042

70. Barathi A, Krishnamoorthy Y, Sinha P, Horsburgh C, Hochberg N, Johnson E, et al. Effect of treatment adherence on the association between sex and unfavourable treatment outcomes among tuberculosis patients in Puducherry, India: a mediation analysis. J Public Health. 2023;45: 304–311. doi:10.1093/pubmed/fdac062

71. Chen AZ, Kumar R, Baria RK, Shridhar PK, Subbaraman R, Thies W. Impact of the 99DOTS digital adherence technology on tuberculosis treatment outcomes in North India: a pre-post study. BMC Infect Dis. 2023;23: 504. doi:10.1186/s12879-023-08418-2

72. Prajapati AC, Shah T, Panchal S, Joshi B, Shringarpure K, Jakasania A, et al. Treatment outcomes and associated factors among patients with drug-sensitive tuberculosis on daily

fixed-dose combination drugs: A cohort study from Ahmedabad, India. J Family Med Prim Care. 2023;12: 452–459. doi:10.4103/jfmpc.jfmpc_1331_22

73. Deepa D, Achanta S, Jaju J, Rao K, Samyukta R, Claassens M, et al. The Impact of Isoniazid Resistance on the Treatment Outcomes of Smear Positive Re-Treatment Tuberculosis Patients in the State of Andhra Pradesh, India. PLoS ONE. 2013;8: e76189. doi:10.1371/journal.pone.0076189

74. Das M, Isaakidis P, Shenoy R, Anicete R, Sharma HK, Ao I, et al. Self-Administered Tuberculosis Treatment Outcomes in a Tribal Population on the Indo-Myanmar Border, Nagaland, India. PLoS ONE. 2014;9: e108186. doi:10.1371/journal.pone.0108186

75. Huddart S, Ingawale P, Edwin J, Jondhale V, Pai M, Benedetti A, et al. TB case fatality and recurrence in a private sector cohort in Mumbai, India. Int J Tuberc Lung Dis. 2021;25: 738–746. doi:10.5588/ijtld.21.0266

76. Bhargava A, Chatterjee M, Jain Y, Chatterjee B, Kataria A, Bhargava M, et al. Nutritional status of adult patients with pulmonary tuberculosis in rural central India and its association with mortality. PLoS One. 2013;8: e77979. doi:10.1371/journal.pone.0077979

77. Sharma V, Thekkur P, Naik PR, Saha BK, Agrawal N, Dinda MK, et al. Treatment success rates among drug susceptible tuberculosis patients notified from the private sector in West Bengal, India. Monaldi Archives for Chest Disease. 2021;91: 1555.

78. Vasantha M, Gopi PG, Subramani R. Survival of tuberculosis patients treated under DOTS in a rural tuberculosis unit (TU), South India. Indian Journal of Tuberculosis. 2008;55: 64–69.

79. Islam S, Das S, Das DK. Nutritional status and adherence to anti-tubercular treatment among tuberculosis patients in a community development block of Eastern India. Indian J Tuberc. 2023. Epub 2023 Apr 18. doi:10.1016/j.ijtb.2023.04.005

80. Gopi PG, Chandrasekaran V, Subramani R, Santha T, Thomas A, Selvakumar N, et al. Association of conversion & cure with initial smear grading among new smear positive pulmonary tuberculosis patients treated with Category I regimen. Indian J Med Res. 2006;123: 807–814.

81. Babiarz KS, Suen S, Goldhaber-Fiebert JD. Tuberculosis treatment discontinuation and symptom persistence: an observational study of Bihar, India’s public care system covering >100,000,000 inhabitants. BMC Public Health. 2014;14: 418. doi:10.1186/1471-2458-14-418

82. Singh M, Sagili KD, Tripathy JP, Kishore S, Bahurupi YA, Kumar A, et al. Are Treatment Outcomes of Patients with Tuberculosis Detected by Active Case Finding Different From Those Detected by Passive Case Finding? J Glob Infect Dis. 2020;12: 28–33. doi:10.4103/jgid.jgid_66_19

83. Mundra A, Deshmukh P, Dawale A. Determinants of adverse treatment outcomes among patients treated under Revised National Tuberculosis Control Program in Wardha, India: Case–control study. Med J Armed Forces India. 2018;74: 241–249. doi:10.1016/j.mjafi.2017.07.008

84. Bhagat VM, Gattani PL. Factors affecting tuberculosis retreatment defaults in Nanded, India. Southeast Asian J Trop Med Public Health. 2010;41: 1153–1157.

85. Sarpal SS, Goel NK, Kumar D, Janmeja AK. Treatment Outcome Among the Retreatment Tuberculosis (TB) Patients under RNTCP in Chandigarh, India. J Clin Diagn Res. 2014;8: 53–56. doi:10.7860/JCDR/2014/6510.4006

86. Maroof M, Pamei G, Bhatt M, Awasthi S, Bahuguna SC, Singh P. Drug adherence to anti-tubercular treatment during COVID-19 lockdown in Haldwani block of Nainital district. Indian J Community Health. 2022;34: 535–541.

87. Panati D, Chittooru CS, Madarapu YR, Gorantla AK. Effect of depression on treatment adherence among elderly tuberculosis patients: A prospective interventional study. Clin Epidemiol Glob Health. 2023;22: 101338. doi:10.1016/j.cegh.2023.101338

88. Velavan A, Purty AJ, Shringarpure K, Sagili KD, Mishra AK, Selvaraj KS, et al. Tuberculosis retreatment outcomes and associated factors: a mixed-methods study from Puducherry, India. public health action. 2018;8: 187–193. doi:10.5588/pha.18.0038

89. Kamble BD, Malhotra S. Profile and treatment outcomes among young patients with tuberculosis aged 15-24 years in Faridabad district of Haryana, India. BMJ Open. 2022;12: e060363. doi:10.1136/bmjopen-2021-060363

90. Siddiqui AN, Khayyam KU, Sharma M. Effect of Diabetes Mellitus on Tuberculosis Treatment Outcome and Adverse Reactions in Patients Receiving Directly Observed Treatment Strategy in India: A Prospective Study. BioMed Res Int. 2016;2016: 1–11. doi:10.1155/2016/7273935

91. Umayorubhagom A, Baliga SS. Factors affecting tuberculosis treatment outcome among newly diagnosed tuberculosis patients – A longitudinal study. Indian J Tuberc. 2023. Epub 2023 Jun 7. doi:10.1016/j.ijtb.2023.06.007

92. Rouf A, Masoodi MA, Dar MM, Khan SMS, Bilquise R. Depression among Tuberculosis patients and its association with treatment outcomes in district Srinagar. J Clin Tuberc Other Mycobact Dis. 2021;25: 100281. doi:10.1016/j.jctube.2021.100281

93. Balasubramanian R, Garg R, Santha T, Gopi PG, Subramani R, Chandrasekaran V, et al. Gender disparities in tuberculosis: report from a rural DOTS programme in south India. Int J Tuberc Lung Dis. 2004;8: 323–332.

94. Prajapati AC, Shah T, Panchal S, Joshi B, Shringarpure K, Jakasania A, et al. Treatment outcomes and associated factors among patients with drug-sensitive tuberculosis on daily fixed-dose combination drugs: A cohort study from Ahmedabad, India. J Family Med Prim Care. 2023;12: 452–459. doi:10.4103/jfmpc.jfmpc_1331_22

95. Islam S, Das S, Das D kumar. Nutritional status and adherence to anti-tubercular treatment among tuberculosis patients in a community development block of Eastern India. Indian Journal of Tuberculosis. 2023. Epub 2023 Apr 18. doi:10.1016/j.ijtb.2023.04.005

96. Umayorubhagom A, Baliga SS. Factors affecting tuberculosis treatment outcome among newly diagnosed tuberculosis patients – A longitudinal study. Indian Journal of Tuberculosis. 2023. Epub 2023 Jun 7. doi:10.1016/j.ijtb.2023.06.007

97. Singla R, Bharty SK, Gupta UA, Khayyam KU, Vohra V, Singla N, et al. Sputum smear positivity at two months in previously untreated pulmonary tuberculosis patients. Int J Mycobacteriol. 2013;2: 199–205. doi:10.1016/j.ijmyco.2013.08.002

98. Ahmed MV, Nirgude AS, Naik PR, Mandolikar RY. Assessment of patient related risk factors pertaining to default and non-default among study population. Journal of Pharmaceutical Negative Results. 2022;13: 2410–2415. doi:10.47750/pnr.2022.13

99. Singla R, Sarin R, Khalid UK, Mathuria K, Singla N, Jaiswal A, et al. Seven-year DOTS-Plus pilot experience in India: results, constraints and issues. Int J Tuberc Lung Dis. 2009;13: 976–981.

100. Gopi PG, Vasantha M, Muniyandi M, Chandrasekaran V, Balasubramanian R, Narayanan PR. Risk factors for non-adherence to directly observed treatment (DOT) in a rural tuberculosis unit, South India. Indian Journal of Tuberculosis. 2007;54: 66–70.

101. Kamble BD, Malhotra S. Profile and treatment outcomes among young patients with tuberculosis aged 15-24 years in Faridabad district of Haryana, India. BMJ Open. 2022;12: e060363. doi:10.1136/bmjopen-2021-060363

102. Ramachandran G, Chandrasekaran P, Gaikwad S, Agibothu Kupparam HK, Thiruvengadam K, Gupte N, et al. Subtherapeutic Rifampicin Concentration Is Associated With Unfavorable Tuberculosis Treatment Outcomes. Clin Infect Dis. 2020;70: 1463–1470. doi:10.1093/cid/ciz380

103. M. S, K. M, Marconi S, V. K, S. R, Prasad J. A community based case control study on risk factors for treatment interruptions in people with tuberculosis in Kollam district, Kerala, southern India. Int J Community Med Public Health. 2016;3: 962–967. doi:10.18203/2394-6040.ijcmph20160937

104. Motappa R, Fathima T, Kotian H. Appraisal on patient compliance and factors influencing the daily regimen of anti-tubercular drugs in Mangalore city: A cross-sectional study. F1000Res. 2022;11: 462. doi:10.12688/f1000research.109006.2

105. Bhargava A, Chatterjee M, Jain Y, Chatterjee B, Kataria A, Bhargava M, et al. Nutritional status of adult patients with pulmonary tuberculosis in rural central India and its association with mortality. PLoS One. 2013;8: e77979. doi:10.1371/journal.pone.0077979

106. Panati D, Chittooru CS, Madarapu YR, Gorantla AK. Effect of depression on treatment adherence among elderly tuberculosis patients: A prospective interventional study. Clinical Epidemiology and Global Health. 2023;22: 101338. doi:10.1016/j.cegh.2023.101338

107. Mave V, Gaikwad S, Barthwal M, Chandanwale A, Lokhande R, Kadam D, et al. Diabetes Mellitus and Tuberculosis Treatment Outcomes in Pune, India. Open Forum Infectious Diseases. 2021;8: ofab097. doi:10.1093/ofid/ofab097

108. Jaggarajamma K, Sudha G, Chandrasekaran V, Nirupa C, Thomas A, Santha T, et al. Reasons for non-compliance among patients treated under Revised National Tuberculosis Control Programme (RNTCP), Tiruvallur district, south India. Indian J Tuberc. 2007;54: 130–135.

109. Kulkarni P, Akarte S, Mankeshwar R, Bhawalkar J, Banerjee A, Kulkarni A. Non-adherence of new pulmonary tuberculosis patients to anti-tuberculosis treatment. Ann Med Health Sci Res. 2013;3: 67–74. doi:10.4103/2141-9248.109507

110. Gupta S, Gupta S, Behera D. Reasons for interruption of anti-tubercular treatment as reported by patients with tuberculosis admitted in a tertiary care institute. Indian J Tuberc. 2011;58: 11–17.

111. Zaman F, Sheikh S, Das K, Zaman G, Pal R. An epidemiological study of newly diagnosed sputum positive tuberculosis patients in Dhubri district, Assam, India and the factors influencing their compliance to treatment. J Nat Sc Biol Med. 2014;5: 415–420. doi:10.4103/0976-9668.136213

112. Ahmed M, Mohan R. A comparative study of factors for interruption of antitubercular treatment among defaulters in urban and rural areas of Kamrup District, Assam. J Family Med Prim Care. 2021;10: 127–131. doi:10.4103/jfmpc.jfmpc_1027_20

113. Mittal C, Gupta S. Noncompliance to DOTS: How it can be decreased. Indian J Community Med. 2011;36: 27–30. doi:10.4103/0970-0218.80789

114. Lata S, Khajuria V, Sawhney V, Kumari K. Evaluation of non-adherence to antitubercular drugs among tuberculosis patients: a prospective study. Int J Curr Pharm Res. 2021;13: 26–28. doi:10.22159/ijcpr.2021v13i2.41550

115. Shabil M, Rajesh V, Raj KCB, Rajesh KS, Shama KP, Gururaja MP, et al. A Study on Treatment Defaulters in Tuberculosis Patients on DOTS Therapy. Res J Pharm Technol. 2019;12: 2245–53. doi:10.5958/0974-360X.2019.00374.3

116. Yadav GS, Jangid VK, Mathur BB. Study of various reasons for interruption of anti-tubercular treatment in patients of tuberculosis reporting to tertiary care center of west Rajasthan. Int J Res Med Sci. 2019;7: 2220. doi:10.18203/2320-6012.ijrms20192542

117. Jaiswal S, Sharma H, Joshi U, Agrawal M, Sheohare R. Non-adherence to anti-tubercular treatment during COVID-19 pandemic in Raipur district Central India. Indian J Tuberc. 2022;69: 558–564. doi:10.1016/j.ijtb.2021.08.033

118. Dole SS, Waghmare VN, Shaikh AM. Clinical Profile and Treatment Outcome of Drug Resistant Tuberculosis Patients of Western Maharashtra, India. J Assoc Physicians India. 2017;65: 18–21.

119. Natarajan S, Singla R, Singla N, Gupta A, Caminero JA, Chakraborty A, et al. Treatment interruption patterns and adverse events among patients on bedaquiline containing regimen under programmatic conditions in India. Pulmonology. 2022;28: 203–209. doi:10.1016/j.pulmoe.2020.09.006

120. Patel SV, Nimavat KB, Patel AB, Mehta KG, Shringarpure K, Shukla LK. Sputum Smear and Culture Conversion in Multidrug Resistance Tuberculosis Patients in Seven Districts of Central Gujarat, India: A Longitudinal Study. Indian J Community Med. 2018;43: 117–119. doi:10.4103/ijcm.IJCM_152_17

121. Vijay S, Kumar P, Chauhan LS, Narayan Rao SV, Vaidyanathan P. Treatment Outcome and Mortality at One and Half Year Follow-Up of HIV Infected TB Patients Under TB Control Programme in a District of South India. PLoS ONE. 2011;6: e21008. doi:10.1371/journal.pone.0021008

122. Sharma SK, Soneja M, Prasad KT, Ranjan S. Clinical profile & predictors of poor outcome of adult HIV-tuberculosis patients in a tertiary care centre in north India. Indian J Med Res. 2014;139: 154–160.

123. Ambadekar NN, Zodpey SP, Soni RN, Lanjewar SP. Treatment outcome and its attributes in TB-HIV co-infected patients registered under Revised National TB Control Program: a retrospective cohort analysis. Public Health. 2015;129: 783–789. doi:10.1016/j.puhe.2015.03.006

124. Ranganath TS, Kishore SG, Reddy R, Murthy HJD, Vanitha B, Sharath BN, et al. Risk factors for non-adherence among people with HIV-associated TB in Karnataka, India: A case-control study. Indian J Tuberc. 2022;69: 65–72. doi:10.1016/j.ijtb.2021.03.003

125. D M, U A, L K, V V, A S. Clinicodemographic profile and outcome of tuberculosis treatment in TB-HIV co-infected patients receiving daily ATT under a single window TB/HIV services delivery initiative. Monaldi Arch Chest Dis. 2022;93. doi:10.4081/monaldi.2022.2405

126. Ambadekar NN, Zodpey SP, Soni RN, Lanjewar SP. Treatment outcome and its attributes in TB-HIV co-infected patients registered under Revised National TB Control Program: a

retrospective cohort analysis. Public Health. 2015;129: 783–789. doi:10.1016/j.puhe.2015.03.006

127. Vashishtha R, Mohan K, Singh B, Devarapu SK, Sreenivas V, Ranjan S, et al. Efficacy and safety of thrice weekly DOTS in tuberculosis patients with and without HIV co-infection: an observational study. BMC Infect Dis. 2013;13: 468. doi:10.1186/1471-2334-13-468

128. Lisha PV, James PT, Ravindran C. Morbidity and mortality at five years after initiating Category I treatment among patients with new sputum smear positive pulmonary tuberculosis. Indian J Tuberc. 2012;59: 83–91.

129. Mahishale V, Patil B, Lolly M, Eti A, Khan S. Prevalence of Smoking and Its Impact on Treatment Outcomes in Newly Diagnosed Pulmonary Tuberculosis Patients: A Hospital-Based Prospective Study. Chonnam Med J. 2015;51: 86–90. doi:10.4068/cmj.2015.51.2.86

130. Tripathy S, Anand A, Inamdar V, Manoj MM, Khillare KM, Datye AS, et al. Clinical response of newly diagnosed HIV seropositive & seronegative pulmonary tuberculosis patients with the RNTCP Short Course regimen in Pune, India. Indian J Med Res. 2011;133: 521–528.

131. Dandekar R, Dixit J, Srinivasan D. The fate of tuberculosis cases after two years of DOTS chemotherapy in Aurangabad city, Maharashtra. National Journal of Community Medicine. 2014;5: 174–178.

132. Sadacharam K, Gopi PG, Chandrasekaran V, Eusuff SI, Subramani R, Santha T, et al. Status of smear-positive TB patients at 2-3 years after initiation of treatment under a DOTS programme. Indian J Tuberc. 2007;54: 199–203.

133. Sharma R, Prajapati S, Patel P, Patel B, Gajjar S, Bapat N. An Outcome-Based Follow-up Study of Cured Category I Pulmonary Tuberculosis Adult Cases from Various Tuberculosis Units under Revised National Tuberculosis Control Program from a Western Indian City. Indian J Community Med. 2019;44: 48–52. doi:10.4103/ijcm.IJCM_310_18

134. Selvaraju S, Thiruvengadam K, Watson B, Thirumalai N, Malaisamy M, Vedachalam C, et al. Long-term Survival of Treated Tuberculosis Patients in Comparison to a General Population In South India: A Matched Cohort Study. Int J Infect Dis. 2021;110: 385–393. doi:10.1016/j.ijid.2021.07.067

135. Kolappan C, Subramani R, Kumaraswami V, Santha T, Narayanan PR. Excess mortality and risk factors for mortality among a cohort of TB patients from rural south India. Int J Tuberc Lung Dis. 2008;12: 81–86.

136. Kolappan C, Subramani R, Karunakaran K, Narayanan PR. Mortality of tuberculosis patients in Chennai, India. Bull World Health Organ. 2006;84: 555–560. doi:10.2471/blt.05.022087

137. Thomas A, Gopi PG, Santha T, Chandrasekaran V, Subramani R, Selvakumar N, et al. Predictors of relapse among pulmonary tuberculosis patients treated in a DOTS programme in South India. Int J Tuberc Lung Dis. 2005;9: 556–561.

138. Gupte AN, Selvaraju S, Paradkar M, Danasekaran K, Shivakumar SVBY, Thiruvengadam K, et al. Respiratory health status is associated with treatment outcomes in pulmonary tuberculosis. Int J Tuberc Lung Dis. 2019;23: 450–457. doi:10.5588/ijtld.18.0551

139. Helfinstein S, Engl E, Thomas BE, Natarajan G, Prakash P, Jain M, et al. Understanding why at-risk population segments do not seek care for tuberculosis: a precision public health approach in South India. BMJ Glob Health. 2020;5: e002555. doi:10.1136/bmjgh-2020-002555

140. Chen AZ, Kumar R, Baria RK, Shridhar PK, Subbaraman R, Thies W. Impact of the 99DOTS digital adherence technology on tuberculosis treatment outcomes in North India: a pre-post study. BMC Infect Dis. 2023;23: 504. doi:10.1186/s12879-023-08418-2

141. Sharma N, Khanna A, Chandra S, Basu S, Chopra K, Singla N, et al. Trends & treatment outcomes of multidrug-resistant tuberculosis in Delhi, India (2009-2014): A retrospective record-based study. Indian J Med Res. 2020;151: 598–603. doi:10.4103/ijmr.IJMR_1048_18

142. Nair D, Navneethapandian PD, Tripathy JP, Harries AD, Klinton JS, Watson B, et al. Impact of rapid molecular diagnostic tests on time to treatment initiation and outcomes in patients with multidrug-resistant tuberculosis, Tamil Nadu, India. Trans R Soc Trop Med Hyg. 2016;110: 534–541. doi:10.1093/trstmh/trw060

143. Bhatt R, Chopra K, Vashisht R. Impact of integrated psycho-socio-economic support on treatment outcome in drug resistant tuberculosis – A retrospective cohort study. Indian Journal of Tuberculosis. 2019;66: 105–110. doi:10.1016/j.ijtb.2018.05.020

144. Velayutham B, Shah V, V. Mythily, Gopalaswamy R, Kumar N, Mandal S, et al. Factors influencing treatment outcomes in patients with isoniazid-resistant pulmonary TB. Int J Tuberc Lung Dis. 2022;26: 1033–1040. doi:10.5588/ijtld.21.0701

145. Janmeja AK, Aggarwal D, Dhillon R. Factors predicting treatment success in multi-drug resistant tuberculosis patients treated under programmatic conditions. Indian J Tuberc. 2018;65: 135–139. doi:10.1016/j.ijtb.2017.12.015

146. Das M, Isaakidis P, Armstrong E, Gundipudi NR, Babu RB, Qureshi IA, et al. Directly-Observed and Self-Administered Tuberculosis Treatment in a Chronic, Low-Intensity Conflict Setting in India. Wilkinson RJ, editor. PLoS ONE. 2014;9: e92131. doi:10.1371/journal.pone.0092131

147. Maji D, Agarwal U, Kumar L, V V, Sharma A. Clinicodemographic profile and outcome of tuberculosis treatment in TB-HIV co-infected patients receiving daily ATT under a single

window TB/HIV services delivery initiative. Monaldi Arch Chest Dis. 2022;93. doi:10.4081/monaldi.2022.2405

148. Satyanarayana S, Nair SA, Chadha SS, Sharma G, Yadav S, Mohanty S, et al. Health-care seeking among people with cough of 2 weeks or more in India. Is passive TB case finding sufficient? Public Health Action. 2012;2: 157–161. doi:10.5588/pha.12.0019

149. Lohiya S, Tripathy JP, Sagili K, Khanna V, Kumar R, Ojha A, et al. Does Drug-Resistant Extrapulmonary Tuberculosis Hinder TB Elimination Plans? A Case from Delhi, India. Trop Med Infect Dis. 2020;5: 109. doi:10.3390/tropicalmed5030109

150. Johnson JM, Mohapatra AK, Velladath SU, Shettigar KS. Predictors of treatment outcomes in drug resistant tuberculosis-observational retrospective study. Int J Mycobacteriol. 2022;11: 38–46. doi:10.4103/ijmy.ijmy_244_21

151. Kalagani Y, Chary VG. Predictors of Unfavorable Treatment Outcome in Patients with Multidrug-Resistant Tuberculosis: A Prospective Study. European Journal of Molecular and Clinical Medicine. 2022;9: 4662–4668.

152. Bagchi S, Ambe G, Sathiakumar N. Determinants of Poor Adherence to Anti-Tuberculosis Treatment in Mumbai, India. Int J Prev Med. 2010;1: 223–32.

153. Duraisamy K, Mrithyunjayan S, Ghosh S, Nair SA, Balakrishnan S, Subramoniapillai J, et al. Does Alcohol Consumption during Multidrug-resistant Tuberculosis Treatment Affect Outcome?. A Population-based Study in Kerala, India. Ann Am Thorac Soc. 2014;11: 712–718. doi:10.1513/AnnalsATS.201312-447OC

154. Maroof M, Pamei G, Bhatt M, Awasthi S, Bahuguna SC, Singh P. Drug adherence to anti-tubercular treatment during COVID-19 lockdown in Haldwani block of Nainital district. Indian Journal of Community Health. 2022;34: 535–541. doi:10.47203/IJCH.2022.v34i04.016

155. Motappa R, Fathima T, Kotian H. Appraisal on patient compliance and factors influencing the daily regimen of anti-tubercular drugs in Mangalore city: A cross-sectional study. F1000Res. 2022;11: 462. doi:10.12688/f1000research.109006.2

156. Shringarpure KS, Isaakidis P, Sagili KD, Baxi RK. Loss-To-Follow-Up on Multidrug Resistant Tuberculosis Treatment in Gujarat, India: The WHEN and WHO of It. PLoS One. 2015;10: e0132543. doi:10.1371/journal.pone.0132543

157. Thomas BE, Thiruvengadam K, S R, Rani S, S V, Gangadhar Rao V, et al. Understanding health care-seeking behaviour of the tribal population in India among those with presumptive TB symptoms. PLoS One. 2021;16: e0250971. doi:10.1371/journal.pone.0250971

158. Thomas BE, Charles N, Watson B, Chandrasekaran V, Senthil Kumar R, Dhanalakshmi A, et al. Prevalence of chest symptoms amongst brick kiln migrant workers and care seeking behaviour: a study from South India. J Public Health. 2015;37: 590–596. doi:10.1093/pubmed/fdu104

159. Ghosh S, Sinhababu A, Taraphdar P, Mukhopadhyay DK, Mahapatra BS, Biswas AB. A study on care seeking behavior of chest symptomatics in a slum of Bankura, West Bengal. Indian J Public Health. 2010;54: 42–44. doi:10.4103/0019-557X.70553

160. Charles N, Thomas B, Watson B, Raja Sakthivel M, Chandrasekeran V, Wares F. Care seeking behavior of chest symptomatics: a community based study done in South India after the implementation of the RNTCP. PLoS One. 2010;5: e12379. doi:10.1371/journal.pone.0012379

161. Suganthi P, Chadha VK, Ahmed J, Umadevi G, Kumar P, Srivastava R, et al. Health seeking and knowledge about tuberculosis among persons with pulmonary symptoms and tuberculosis cases in Bangalore slums. Int J Tuberc Lung Dis. 2008;12: 1268–1273.

162. Dave P, Nimavat P, Shah A, Pujara K, Patel P, Modi B. Knowing more about initial default among diagnosed sputum smear-positive pulmonary tuberculosis patients in Gujarat, India. Int J Tuberc Lung Dis. 2013;17: S469.

163. Pillai D, Purty A, Prabakaran S, Singh Z, Soundappan G, Anandan V. Initial default among tuberculosis patients diagnosed in selected medical colleges of Puducherry: issues and possible interventions. Int J Med Sci Public Health. 2015;4: 957–960. doi:10.5455/IJMSPH.2015.30012015196

164. Gopi P, Chandrasekaran V, Subramani R, Narayanan P. Failure to initiate treatment for tuberculosis patients diagnosed in a community survey and at health facilities under a DOTS program in a district of south India. Indian J Tuberc. 2005;52: 153–156.
