# Supplementary material for: Barriers to engagement in the care cascade for tuberculosis disease in India: A systematic review of quantitative studies
Source: PLoS Med. 2024 May 28;21(5):e1004409. doi: 10.1371/journal.pmed.1004409 (PMC11166313; doi:10.1371/journal.pmed.1004409)
Supplement: S7 Appendix — (PDF) [file pmed.1004409.s007.pdf]

S7 Appendix. PRISMA checklist for all care cascade gaps

Supplement to:
Barriers to engagement in the care cascade for tuberculosis disease in India: a systematic review of quantitative studies

Authors:
Tulip A. Jhaveri, Disha Jhaveri, Amith Galivanche, Maya Lubeck-Schricker, Dominic Voehler, Mei Chung, Pruthu Thekkur, Vineet Chadha, Ruvandhi Nathavitharana, Ajay M.V. Kumar, Hemant Deepak Shewade, Katherine Powers, Kenneth H. Mayer, Jessica E. Haberer, Paul Bain, Madhukar Pai, Srinath Satyanarayana, Ramnath Subbaraman

Correspondence:
Ramnath Subbaraman, MD, MSc, FACP, FIDSA
Tufts University School of Medicine
Department of Public Health and Community Medicine
136 Harrison Ave., MV120
Boston, MA 02130, USA
Email: ramnath.subbaraman@tufts.edu

PRISMA 2020 Abstract Checklist

Section and Topic	Item #	Checklist item	Reported (Yes/No)
**TITLE**			
Title	1	Identify the report as a systematic review.	Yes
**BACKGROUND**			
Objectives	2	Provide an explicit statement of the main objective(s) or question(s) the review addresses.	Yes, last sentence of “Background”
**METHODS**			
Eligibility criteria	3	Specify the inclusion and exclusion criteria for the review.	Yes, third sentence of “Methods and Findings”
Information sources	4	Specify the information sources (e.g. databases, registers) used to identify studies and the date when each was last searched.	Yes, second sentence of “Methods and Findings”
Risk of bias	5	Specify the methods used to assess risk of bias in the included studies.	Yes, third sentence of the “Methods and Findings”
Synthesis of results	6	Specify the methods used to present and synthesise results.	Yes, fourth sentence of “Methods and Findings”
**RESULTS**			
Included studies	7	Give the total number of included studies and participants and summarise relevant characteristics of studies.	Yes, in the third paragraph of the “Methods and Findings,” with total studies reported per care care cascade gap

Section and Topic	Item #	Checklist item	Reported (Yes/No)
Synthesis of results	8	Present results for main outcomes, preferably indicating the number of included studies and participants for each. If meta-analysis was done, report the summary estimate and confidence/credible interval. If comparing groups, indicate the direction of the effect (i.e. which group is favoured).	Yes, in the second and third paragraph of the "Methods and Findings"
**DISCUSSION**			
Limitations of evidence	9	Provide a brief summary of the limitations of the evidence included in the review (e.g. study risk of bias, inconsistency and imprecision).	Yes, last sentence of the "Methods and Findings"
Interpretation	10	Provide a general interpretation of the results and important implications.	Yes, in the "Conclusions"
**OTHER**			
Funding	11	Specify the primary source of funding for the review.	This is included in a separate statement in the online journal system.
Registration	12	Provide the register name and registration number.	PROSPERO IDs are included in the S1 to S5 Appendices.

From: Page MJ, McKenzie JE, Bossuyt PM, Boutron I, Hoffmann TC, Mulrow CD, et al. The PRISMA 2020 statement: an updated guideline for reporting systematic reviews. BMJ 2021;372:n71. doi: 10.1136/bmj.n71

PRISMA 2020 Manuscript Checklist

Section and Topic	Item #	Checklist item	Location where item is reported
**TITLE**			
Title	1	Identify the report as a systematic review.	Title page
**ABSTRACT**			
Abstract	2	See the PRISMA 2020 for Abstracts checklist.	Abstract page; see the separate PRISMA abstract checklist included on the previous two pages of this document
**INTRODUCTION**			
Rationale	3	Describe the rationale for the review in the context of existing knowledge.	Introduction, paragraphs 2, 3
Objectives	4	Provide an explicit statement of the objective(s) or question(s) the review addresses.	Introduction, paragraph 3
**METHODS**			
Eligibility criteria	5	Specify the inclusion and exclusion criteria for the review and how studies were grouped for the syntheses.	Methods section, subsection on 'Search strategy and study selection', paragraph 2<br>Appendices S1-S5, section on 'Inclusion and exclusion criteria'
Information sources	6	Specify all databases, registers, websites, organisations, reference lists and other sources searched or consulted to identify studies. Specify the date when each source was last searched or consulted.	Methods section, Subsection on 'Search strategy and study selection', paragraph 1<br>Appendices S1-S5, Section on 'Search strategy'
Search strategy	7	Present the full search strategies for all databases, registers and websites, including any filters and limits used.	Methods section, subsection on 'Search strategy and study selection', paragraph 1<br>Appendices S1-S5, section on 'Search strategy' and Table A in each appendix
Selection process	8	Specify the methods used to decide whether a study met the inclusion criteria of the review, including how many reviewers screened each record and each report retrieved, whether they worked independently, and if applicable, details of automation tools used in the process.	Methods section, subsection on 'Search strategy and study selection', paragraph 2<br>Appendices S1-S5, section on 'Study selection' in each appendix
Data collection process	9	Specify the methods used to collect data from reports, including how many reviewers collected data from each report, whether they worked independently, any processes for obtaining or confirming data from study investigators, and if applicable, details of automation tools used in the process.	Methods section, subsection on 'Data extraction', paragraph 1

Section and Topic	Item #	Checklist item	Location where item is reported
			Appendices S1-S5, section on 'Data extraction and analysis', paragraph 1
Data items	10a	List and define all outcomes for which data were sought. Specify whether all results that were compatible with each outcome domain in each study were sought (e.g. for all measures, time points, analyses), and if not, the methods used to decide which results to collect.	Methods section, subsection on 'TB care cascade framework', Table 1<br>Methods section, subsection on 'PECO framework', Paragraph 4<br>Methods section, subsection on 'Data extraction', paragraph 1<br>Appendices S1-S5, entire sections on 'Objectives' in each appendix and first two paragraphs on 'Data extraction and analysis' in each appendix
	10b	List and define all other variables for which data were sought (e.g. participant and intervention characteristics, funding sources). Describe any assumptions made about any missing or unclear information.	Methods section, subsection on 'TB care cascade framework', Table 1<br>Methods section, subsection on 'PECO framework', paragraphs 1, 2, 3<br>Methods section, subsection on 'Data extraction', paragraphs 1 and 2<br>Appendices S1-S5, section on 'Data extraction and analysis' in each appendix, especially paragraph 1
Study risk of bias assessment	11	Specify the methods used to assess risk of bias in the included studies, including details of the tool(s) used, how many reviewers assessed each study and whether they worked independently, and if applicable, details of automation tools used in the process.	Methods section, subsection on 'Quality assessment'<br>Appendices S1-S5, section on 'Quality assessment of quantitative studies', Table B
Effect measures	12	Specify for each outcome the effect measure(s) (e.g. risk ratio, mean difference) used in the synthesis or presentation of results.	Methods section, subsection on 'PECO framework,' paragraphs 2 and 3<br>Methods section, subsection on 'Data extraction', paragraphs 1, 2

Section and Topic	Item #	Checklist item	Location where item is reported
			Appendices S1-S5, section on ‘Data extraction and analysis’ and Table D (which presents original effect measures from all studies) in each appendix
Synthesis methods	13a	Describe the processes used to decide which studies were eligible for each synthesis (e.g. tabulating the study intervention characteristics and comparing against the planned groups for each synthesis (item #5)).	Methods section, subsection on ‘Framework for organizing and visualizing findings’, paragraphs 1, 2, Table 2<br>Methods section, subsection on ‘Quality assessment’, last sentence<br>Appendices S1-S5, section on ‘Data extraction and analysis’ in each appendix
	13b	Describe any methods required to prepare the data for presentation or synthesis, such as handling of missing summary statistics, or data conversions.	Methods section, Subsection on ‘Framework for organizing and visualizing findings’, paragraphs 1, 2<br>Appendices S1-S5, section on ‘Data extraction and analysis’ in each appendix
	13c	Describe any methods used to tabulate or visually display results of individual studies and syntheses.	Methods section, Subsection on ‘Framework for organizing and visualizing findings’, paragraphs 1, 2 and Table 2<br>Appendices S1-S5, section on ‘Data extraction and analysis’ in each appendix
	13d	Describe any methods used to synthesize results and provide a rationale for the choice(s). If meta-analysis was performed, describe the model(s), method(s) to identify the presence and extent of statistical heterogeneity, and software package(s) used.	Methods section, Subsection on ‘Framework for organizing and visualizing findings’, paragraph 1 (especially sentence 3)<br>Appendices S1-S5, section on ‘Data extraction and analysis’ in each appendix
	13e	Describe any methods used to explore possible causes of heterogeneity among study results (e.g. subgroup analysis, meta-regression).	Methods section, Subsection on ‘Framework for organizing and visualizing findings’, paragraphs 1, 2, Table 2<br>Appendices S1-S5, section

Section and Topic	Item #	Checklist item	Location where item is reported
			on 'Data extraction and analysis'
	13f	Describe any sensitivity analyses conducted to assess robustness of the synthesized results.	N/A
Reporting bias assessment	14	Describe any methods used to assess risk of bias due to missing results in a synthesis (arising from reporting biases).	Methods section, Subsection on 'Quality assessment'<br>Appendices S1-S5, Section on 'Quality assessment of quantitative studies', Table B<br>Discussion section, subsection on 'Strengths and limitations of the review', paragraph 2 (regarding rationale for not exploring publication bias)
Certainty assessment	15	Describe any methods used to assess certainty (or confidence) in the body of evidence for an outcome.	Methods section, Subsection on 'Quality assessment'<br>Appendices S1-S5, Section on 'Quality assessment of quantitative studies', table B
**RESULTS**			
Study selection	16a	Describe the results of the search and selection process, from the number of records identified in the search to the number of studies included in the review, ideally using a flow diagram.	Appendices S1-S5, Fig A (PRISMA flowchart for each care cascade gap).<br>Appendix S6, Section on 'Characteristics and quality of the included studies', under each gap
	16b	Cite studies that might appear to meet the inclusion criteria, but which were excluded, and explain why they were excluded.	Appendices S1-S5, Fig A.<br>Appendix S6, Section on 'Characteristics and quality of the included studies', under each gap
Study characteristics	17	Cite each included study and present its characteristics.	Appendices S1-S5, Table C (report of all individual included studies for each care cascade gap)<br>Results section, Table 3 (summarizes characteristics of included studies by care cascade gap)<br>Appendix S6, Section on 'Characteristics and quality of

Section and Topic	Item #	Checklist item	Location where item is reported
			the included studies', under each gap
Risk of bias in studies	18	Present assessments of risk of bias for each included study.	Results section, Table 3<br>Appendices S1-S5, Table C<br>Appendix S6, Section on 'Characteristics and quality of the included studies,' under each gap
Results of individual studies	19	For all outcomes, present, for each study: (a) summary statistics for each group (where appropriate) and (b) an effect estimate and its precision (e.g. confidence/credible interval), ideally using structured tables or plots.	Results section, entire text, Figs 1 to 11 (which report confidence intervals for each finding)<br>Appendices S1-S5, Table D (which reports effect estimates and confidence intervals for all findings)
Results of syntheses	20a	For each synthesis, briefly summarise the characteristics and risk of bias among contributing studies.	Results section, entire text, Figs 1 to 11 (which show directionality for findings from regression analyses and present some study characteristics)<br>Results section, Table 3 (which summarizes study characteristics)<br>Appendix S6, Section on 'Characteristics and quality of the included studies,' under each gap
	20b	Present results of all statistical syntheses conducted. If meta-analysis was done, present for each the summary estimate and its precision (e.g. confidence/credible interval) and measures of statistical heterogeneity. If comparing groups, describe the direction of the effect.	Results section, entire text, Figs 1 to 11 (which show effect estimates and directionality for findings from regression analyses)<br>Appendices S1-S5, Table D in each appendix (which presents all effect estimates)
	20c	Present results of all investigations of possible causes of heterogeneity among study results.	N/A
	20d	Present results of all sensitivity analyses conducted to assess the robustness of the synthesized results.	N/A
Reporting biases	21	Present assessments of risk of bias due to missing results (arising from reporting biases) for each synthesis assessed.	Discussion section, subsection on 'Strengths and limitations of the review',

Section and Topic	Item #	Checklist item	Location where item is reported
			paragraph 2 (regarding rationale for not exploring publication bias)
Certainty of evidence	22	Present assessments of certainty (or confidence) in the body of evidence for each outcome assessed.	Results section, entire text, Figs 1 to 11 (which report confidence intervals for each finding)<br>Appendices S1-S5, Table D
**DISCUSSION**			
Discussion	23a	Provide a general interpretation of the results in the context of other evidence.	Discussion section, subsections on 'Continuities in risk across care cascade stages' and 'Findings specific to each care cascade gap'
	23b	Discuss any limitations of the evidence included in the review.	Discussion section, subsection on 'Strengths and limitations of the review', paragraphs 2, 3<br>Discussion section, paragraph 1 (discusses dearth of studies for some subpopulations of people with TB)
	23c	Discuss any limitations of the review processes used.	Discussion section, subsection on 'Strengths and limitations of the review', paragraphs 2, 3
	23d	Discuss implications of the results for practice, policy, and future research.	Discussion section, paragraph 2<br>Discussion section, subsection on 'Continuities in risk across care cascade stages' and 'Findings specific to each care cascade gap' all paragraphs<br>Conclusion section
**OTHER INFORMATION**			
Registration and protocol	24a	Provide registration information for the review, including register name and registration number, or state that the review was not registered.	Methods section, subsection on 'TB care cascade framework', paragraph 1<br>Appendices S1-S5, section on 'Objectives' in each appendix, paragraph 1

Section and Topic	Item #	Checklist item	Location where item is reported
	24b	Indicate where the review protocol can be accessed, or state that a protocol was not prepared.	Methods section, subsection on 'TB care cascade framework', paragraph 1<br>Appendices S1-S5, section on 'Objectives' in each appendix, paragraph 1
	24c	Describe and explain any amendments to information provided at registration or in the protocol.	N/A
Support	25	Describe sources of financial or non-financial support for the review, and the role of the funders or sponsors in the review.	Funding section
Competing interests	26	Declare any competing interests of review authors.	Competing interests section
Availability of data, code and other materials	27	Report which of the following are publicly available and where they can be found: template data collection forms; data extracted from included studies; data used for all analyses; analytic code; any other materials used in the review.	S1 to S5 Appendices, Table D contains all data from regression analyses that informed Forest Plots<br>References sections of the main manuscript and of the S1 to S5 Appendices provides information on all included studies<br>Methods section, subsection on 'Data extraction,' paragraph 1 notes that the data extraction form is available upon request from the corresponding author<br>Methods section, subsection on 'Framework for organizing and visualizing findings', paragraph 1 notes that Stata code for Forest plots is available upon request from the corresponding author.
