# Supplementary material for: Barriers to engagement in the care cascade for tuberculosis disease in India: A systematic review of quantitative studies
Source: PLoS Med. 2024 May 28;21(5):e1004409. doi: 10.1371/journal.pmed.1004409 (PMC11166313; doi:10.1371/journal.pmed.1004409)
Supplement: Attachment — Submitted filename: jhaveri.pdf [file pmed.1004409.s008.pdf]

This paper presents a review of studies of engagement in the care cascade in India.

I was asked for a statistical report and I interpret that to include all aspects of the design and conduct of the study.

Points of detail

Page 11 and 12 The final search date is now just over two years ago which does seem rather a long delay. An update would improve matters I appreciate that this review took a very considerable amount of work but there are eighteen authors to share the burden of an update.

Page 12 I suppose the treatment as usual arms of clinical trials might be thought relevant. The treatment arms are clearly not relevant.

Figure 7 The confidence intervals are asymmetric, which is correct, but I did not see anywhere what method was adopted to generate them

Point of more substance

Page 14 informs us that only statistically significant results are presented and this is confirmed in the captions of the figures. I find it hard to see why, in 2023, anyone thought this was a good idea. The point of a systematic review is to present to us the totality of evidence and then to draw from it some form of conclusion. It is interesting to look at the example of the use of streptokinase in acute myocardial infarction (Lau et al., 1992) where the first 20 trials include 17 trials which the authors would not have presented and yet the meta–analysis up to that point produced an almost identical result to that following the first large trial (GISSI–1)

Summary

I would regard presenting all the evidence as a mandatory improvement.

Michael Dewey

References

J Lau, E M Antman, J Jimenez-Silva, B Kupelnick, F Mosteller, and T C Chalmers. Cumulative meta-analysis of therapeutic trials for myocardial infarction. *New England Journal of Medicine*, 327:248–254, 1992.
