# Supplementary material for: Barriers to engagement in the care cascade for tuberculosis disease in India: A systematic review of quantitative studies
Source: PLoS Med. 2024 May 28;21(5):e1004409. doi: 10.1371/journal.pmed.1004409 (PMC11166313; doi:10.1371/journal.pmed.1004409)
Supplement: Attachment — Submitted filename: Response to Reviewers comments PMED 3.4.24.docx [file pmed.1004409.s009.docx]

**RESPONSES TO THE EDITORS**

**General comments**

*Please respond to all editor and reviewer comments detailed below in full.

Please cite the reference numbers in square brackets (e.g., “We used the techniques developed by our colleagues [19] to analyze the data”). Citations should be preceding punctuation.*

We have ensured that all references are cited in square brackets before the punctuation.

*Please cite your Supporting Information as outlined here:* [*https://journals.plos.org/plosmedicine/s/supporting-information*](https://journals.plos.org/plosmedicine/s/supporting-information)

We have cited supporting information as outlined in the PLOS Medicine guidelines and have now included a listing of supporting information (with titles for each supporting information document) at the end of the main manuscript.

*Please ensure to be consistent in the use of numbers as words or numerals (e.g., ll.751-756).*

We have now modified the manuscript to report all numbers as numerals (rather than words) throughout.

*Please remove the Financial disclosure statement and Competing interest statement following your ‘Acknowledgements’. This information should only be added in the according section in the online submission form.*

We have removed the financial disclosure and competing interest statement. We will add this in the online submission form.

**Academic editor comments**

*Clearly an ambitious article with some work remaining but has lots of potential.*

We thank the academic editor for this feedback and hope that we have sufficiently improved the manuscript for its full potential to be realized.

**Editorial comments**

*We appreciate your efforts to streamline the manuscript. However, we still feel that the manuscript is quite long and that the manuscripts could benefit from an even greater focus on reader accessibility by possibly placing some data and information in the appendix.*

*We noticed that the Methods and Results sections contain quite a lot of discussion of methodological approaches - we appreciate that the study is very complex, but wonder how much of this could be included/reported as supporting information as part of an analysis plan? This could potentially provide more focus and further streamline your manuscript.*

We appreciate this feedback from the editorial team, and we have cut the Methods substantially from 1,872 words to 1,136 words (reduction of 736 words). The S1 to S5 Appendices already serve as standalone methods sections for each of the Gap 1 to Gap 5 systematic reviews included in this paper. To remove some text from the Methods section of the main manuscript, we did end up including additional details in these existing appendices, such as the PROSPERO IDs for the original registration of these systematic review protocols in 2020. We believe that the Methods section has been streamlined considerably and provides high-level information to help readers understand our approach.

We also cut down the text in the Results section as much as we possibly could, even as our refresher search, which updated the review to August 2023, identified 47 new papers for inclusion. Based on this extensive new data, which largely fit into Gap 4, we took the advice of Reviewer 2 below to reorganize presentation of our Gap 4 findings. We also now present relevant non-significant findings throughout the paper, as this was the primary scientific critique by Reviewers 1 and 2, with which we strongly agreed. *At the same time, given the editorial team’s appropriate concerns about the length of the manuscript, we removed presentation of findings from unadjusted (i.e., univariate) analyses in the main manuscript. After removal of reporting of findings from studies with unadjusted analyses from the main manuscript, we decided to modify the total number of studies reported for each gap in the Abstract, and in Table 3, to reflect the primary findings presented in the main manuscript.*

As such, despite extensive editing, the word count for the Results section has been reduced more modestly from 4,705 to 4,123 words (reduction of 582 words).

We have also removed two tables that summarized findings and put these in the S6 Appendix, while opting for a single more reader-friendly figure (Fig 12) to summarize findings across care cascade gaps at the end of the Results section. We have also cut the word count for the Discussion and Conclusion sections from 2,364 to 1,949 words (reduction of 415 words).

Overall, the manuscript length has been reduced from 9,505 words to 7,505 words, excluding tables and figure captions (overall reduction of 2,000 words, despite the addition of findings from 47 new papers). We also trimmed the length of text in the tables wherever possible and cut the word count for the Abstract and Author Summary slightly.

*Finally, we noticed that the figures look very 'busy', especially Figure 6. Could the data presented in Figure 6 be presented in a table instead? What information value do the forest plots add, given that the confidence intervals are shown next to them? Please consider these points while we leave the modification of your figures to your discretion.*

We greatly appreciate this advice. We definitely considered alternative approaches to presenting the central study findings; however, presenting the findings as Forest plots has important advantages for readers by allowing them to quickly identify trends in the data or discordant findings. For example, in situations where most studies show that a variable is associated with unfavorable outcomes (e.g., male sex vs. female sex with effect estimates greater than 1), readers can quickly identify studies in which there was the opposite directionality (e.g., male sex vs. female sex with an effect estimate less than 1). We took great pains to “flip” outcomes and to “flip” reference groups where needed to ensure similar variables could be compared visually on the Forest plots so that readers can quickly identify trends or discordant findings. In addition, with Forest Plots presenting proportions, readers are quickly able to identify which findings were experiences by a high proportion vs. a very low proportion of people with TB. We also felt it was important to present the effect estimates themselves so that all findings are transparent to the reader.

Our concern with using tables for some of the data-rich plots is that: (1) These are precisely the Forest plots that benefit the reader the most, by allowing them to rapidly visualize trends or discordant findings. For example, Figs 5, 6, and 9 (now the “busiest” Forest plots in the revised manuscript) allow readers to quickly visualize trends in educational status, work-related barriers, and male sex across several studies (to note a few key trends) as barriers to achieving TB treatment success. (2) We also felt it would be inconsistent and confusing to readers to have only selected findings presented tables when all other presentation of adjusted effect estimates were using Forest plots.

*All authors must declare their relevant competing interests per the PLOS policy, which can be seen here:* [*https://journals.plos.org/plosmedicine/s/competing-interests*](https://journals.plos.org/plosmedicine/s/competing-interests) *For authors with ties to industry, please indicate whether any of the interests has a financial stake in the results of the current study."*

We have now included a competing interest statement in the online submission system, which notes the statement regarding Professor Pai’s competing interest while clarifying that all other authors do not have any competing interests.

*Please add this statement to the manuscript's Competing Interests: "MP is an Academic Editor on PLOS Medicine's editorial board."*

We have added this statement in the appropriate place in the online submission system.

**Abstract**

*Please report your abstract according to PRISMA for abstracts, following the PLOS Medicine abstract structure (Background, Methods and Findings, Conclusions)* [*http://www.plosmedicine.org/article/info:doi/10.1371/journal.pmed.1001419*](http://www.plosmedicine.org/article/info:doi/10.1371/journal.pmed.1001419) *.

Please provide the dates of search, data sources, types of study designs included, eligibility criteria, and synthesis/appraisal methods.*

We have revised the abstract to include all of this required information, while also adhering to PLOS Medicine abstract guidelines and keeping it within the maximum word count for abstracts. Notable additions after checklist review include the specific start and end month/days of the searches and general communication of eligibility (inclusion/exclusion criteria), as specific criteria vary by care cascade gap. We have also completed the PRISMA for Abstracts checklist and included this as part of our larger PRISMA checklist in the S7 Appendix for transparency for readers regarding our adherence to the PRISMA guidelines.

**Abstract Methods and Findings:**

*Please ensure that all numbers presented in the abstract are present and identical to numbers presented in the main manuscript text.*

Numbers presented in the abstract are identical to numbers in main manuscript text or in the supplementary information. For example, the numbers of studies cited in the Abstract correspond directly to the total studies for each gap listed in the new Table 3, which summarizes the number and characteristics of included studies across care cascade gaps for findings presented in the main manuscript.

*PLOS Medicine requests that main results are quantified with 95% CIs as well as p values.  Please include.  When reporting p values please report as p<0.001 and where higher as the exact p value p=0.002, for example.  For the purposes of transparent data reporting, if not including the aforementioned please clearly state the reasons why not.*

In the main manuscript, all results have 95% confidence intervals reported in the Forest plots. p-values for these results are included when reported by the original studies in Table D in each of the S1—S5 Appendices, and we have referred readers to Table D in the appropriate supplementary information throughout the Results section. Please note that we reported p-values as they were reported in the original papers included in the systematic review. Unfortunately, we are limited by how these p-values were reported in the original studies. For transparency, we have now clarified that the p-values reported in the supplementary information represent the p-values reported in the original studies in the Methods section, subsection on Data extraction, second paragraph:

“We reported all unadjusted and adjusted effect estimates, including 95%CIs and p-values as reported in the original papers, in Table D in each of the S1—S5 Appendices.”

*Please include any important dependent variables that are adjusted for in the analyses.*

In Table D in each of the S1—S5 Appendices, we have included all dependent variables that were adjusted for in every analysis or study. Table D in each of the S1-S5 Appendices additionally provides the effect estimates for all of these dependent variables that were adjusted for to provide maximal transparency for readers. We have made this point very clear in the revised manuscript in the Methods section, subsection on Data extraction, paragraph 2:

“Dependent variables that were adjusted for can be found in Table D in each of the S1—S5 Appendices.”

*Throughout, suggest reporting statistical information as follows to improve clarity for the reader “22% (95% CI [13%,28%]; p</=)”. Please amend throughout the abstract and main manuscript. Please note the use of commas to separate upper and lower bounds, as opposed to hyphens as these can be confused with reporting of negative values.*

There is no statistical information reported in the abstract. Nearly all findings involving reporting of 95% confidence intervals are in the figures. For reporting of these 95% confidence intervals, we have ensured that these are all reported using commas to separate upper and lower bounds, and we have eliminated any use of hyphens. We have also edited Table D in the S1-S5 Appendices, as we had previously reported confidence intervals with hyphens instead of commas. In the main text, the only place where we report confidence intervals is in the Results section, subsection entitled “Gap 3—Barriers to treatment initiation for people diagnosed with TB disease”, sub-subsection entitled “Factors associated with PTLFU in adults with drug-resistant TB.” These 95% confidence intervals are now separated by commas, and notably, the original study did not report p-values.

Note that, due to space limitations, we have not reported p-values in the main manuscript figures (as noted above), but these p-values are all clearly reported (when available and reported by the original studies) in Table D in each of the S1-S5 Appendices.

*In the last sentence of the Abstract Methods and Findings section, please describe the main limitation(s) of the study's methodology.*

We have added the following sentence as the last sentence of the Methods and Findings section of the Abstract:

“Limitations include lack of meta-analyses due to the heterogeneity of findings and limited generalizability to some Indian regions, given the country’s diverse population.”

**Author summary**

*Thank you for providing an Author Summary. Please shorten the individual bullet points. The summary should include 2-3 single sentence, individual bullet points under each of the questions. This text is subject to editorial change and should be distinct from the scientific abstract. In the final bullet point of ‘What Do These Findings Mean?’, please describe the main limitations of the study in non-technical language.*

Thank you for this feedback. We have edited down the Author Summary from 454 words to 355 words (reduction of 99 words), while also trying to use more non-technical language. We included the following final bullet point in the Author Summary to highlight study limitations:

“Study limitations include lack of meta-analyses (i.e., estimation of the average effect of each risk factor by combining findings across studies), and caution is required when applying findings across India’s large and diverse population.”

**Introduction**

*Please address past research and explain the need for and potential importance of your study. Indicate whether your study is novel and how you determined that. If there has been a systematic review of the evidence related to your study (or you have conducted one), please refer to and reference that review and indicate whether it supports the need for your study. Please conclude the Introduction with a clear description of the study question or hypothesis.*

We address past research on the TB care cascade in India and globally in the first two paragraphs of the introduction, with citations 3 to 9. We then indicate our study is novel in the final sentence of the second paragraph of the Introduction, based on the studies previously cited:

“While prior TB care cascade analyses quantified gaps in care, few analyses have mapped findings from studies across the care cascade to understand *who* is lost and *why* people are lost from care [12].”

Note that this sentence also serves a description of the study question that we are trying to answer with this systematic review.

**Methods and Results**

*Please report your SR/MA according to the PRISMA guidelines provided at the EQUATOR site.* [*http://www.equator-network.org/reporting-guidelines/prisma/*](http://www.equator-network.org/reporting-guidelines/prisma/) *Please provide the completed PRISMA checklist and upload it as Supporting Information. When completing the checklist, please use section and paragraph numbers, rather than page numbers.*

*Please add the following statement, or similar, to the Methods: "This study is reported as per the Preferred Reporting Items for Systematic Reviews and Meta-*

*Analyses (PRISMA) guideline (S1 Checklist)."*

We have reported our systematic review using the PRISMA guidelines, and we have included both the general PRISMA checklist and the PRISMA abstract checklist in the S7 Appendix. These checklists were completed using section and paragraph numbers.

We have included this sentence in the main manuscript Methods section, subsection entitled “TB care cascade framework,” last sentence of the first paragraph:

“Protocols for each gap are provided in the S1—S5 Appendices, following the Preferred Reporting Items for Systematic Reviews and Meta-Analyses (PRISMA) guideline (S7 Appendix Checklist).”

*Please update your search to the present time.*

We conducted a refresher search which has updated this review with almost two years of data for all care cascade gaps through August 14, 2023. This search identified 47 new papers, including more than 40 new papers for Gap 4 alone. We appreciate the patience from the editorial team as we carefully extracted the information from this refresher search. The very large volume of new findings from this refresher search resulted in reorganization of our reporting of Gap 4 findings, based on advice from Reviewer 2, as noted below in our response to that reviewer’s feedback.

*Please evaluate evidence of publication bias.*

We appreciate this advice, but we have decided not to conduct analyses for evidence of publication bias for both scientific and practical reasons. From a scientific perspective, our research approach was not evaluating a set of studies that were all designed to answer the same research question or hypothesis. Instead, we extracted *all exposures* across a range of observational studies, including exposures that were not necessarily related to the hypothesis being tested in each given study. Approaches to evaluating for publication bias, such as funnel plots, are usually premised on trying to identify “holes” or asymmetry in the funnel plot that suggest that small studies with negative findings may not have been published. The underlying assumptions is that all of the included studies were aiming to address the same defined hypothesis. Given that we extracted all exposures from diverse studies with different hypotheses, we do not see a clear rationale for funnel plots. In addition, our approach of extracting all exposures across studies partly guards against the risk of publication bias. From a practical perspective, creating numerous funnel plots would be extremely challenging, while not addressing the scientific problem that these plots are meant to address.

We provide a justification for not exploring publication bias in the Discussion, section on “Strengths and limitations of the review,” paragraph 2:

“We also did not assess publication bias, as meta-analyses were not performed in this review and publication bias is premised on the assumption that included studies were designed to assess the same pre-specified hypotheses.”

*Please mention the language of studies included in your systematic review and if not done so, please include non-English language sources of studies.*

We have clarified that, while we did not restrict our search by language, all studies that have been included in the review happen to have been published in the English language. We have included the following sentence in the Methods section, subsection on “Search strategy and study selection”, first paragraph, last sentence:

“While searches were not restricted by language, all studies meeting inclusion criteria were published in English.”

The fact that all included studies were published in English may reflect that most Indian medical journals are published in English.

*PLOS Medicine requests that main results are quantified with 95% CIs as well as p values.  Please include.  When reporting p values please report as p<0.001 and where higher as the exact p value p=0.002, for example.  For the purposes of transparent data reporting, if not including the aforementioned please clearly state the reasons why not.*

In the main manuscript, all results have 95% confidence intervals reported in the Forest plots. p-values for these results are included when reported by the original studies in Table D in each of the S1—S5 Appendices, and we have referred readers to Table D in the appropriate supplementary information throughout the Results section. Please note that we reported p-values as they were reported in the original papers included in the systematic review. Unfortunately, we are limited by how these p-values were reported in the original studies. For transparency, we have now clarified that the p-values reported in the supplementary information represent the p-values reported in the original studies in the Methods section, subsection on Data extraction, second paragraph:

“We reported all unadjusted and adjusted effect estimates, including 95%CIs and p-values as reported in the original papers, in Table D in each of the S1—S5 Appendices.”

*Please include any important dependent variables that are adjusted for in the analyses.*

In Table D in each of the S1—S5 Appendices, we have included all dependent variables that were adjusted for in every analysis or study. Table D in each of the S1-S5 Appendices additionally provides the effect estimates for all of these dependent variables that were adjusted for to provide maximal transparency for readers. We have made this point very clear in the revised manuscript in the Methods section, subsection on Data extraction, paragraph 2:

“Dependent variables that were adjusted for can be found in Table D in each of the S1—S5 Appendices.”

*Suggest reporting statistical information as detailed above – see under ABSTRACT*

*ll.169-171: For the current manuscript, and since the focus is on the quantitative data reported in this study, should mention of the qualitative data be reserved for the future paper? We suggest changing these statements to "In the current study, we only report quantitative findings. Qualitative findings will be reported elsewhere. We discuss methodological considerations common to both the quantitative and qualitative elements of our study" or similar.*

During the process of editing down the Methods section, all reference to qualitative studies has now been cut. This also addresses the concern raised above about unnecessarily mentioning qualitative data and methodological considerations for qualitative data.

*l.305: Please change to “This allows readers to identify trends (or discordant findings) across studies.”*

We actually edited out this sentence in the process of reducing the length of the Methods section; however, we have now included this phrase as you have suggested in the Methods section, subsection on “Framework for organizing and visualizing findings,” paragraph 2, first sentence:

“To visualize trends (or discordant findings), we combined different unfavorable outcomes (e.g., death, loss to follow-up) or effect estimates (i.e., odds, relative risk, hazard ratios) in the same Forest plots while denoting this in footnotes.”

*l.308: Please define ‘HIV’ at first use.*

We have now defined “HIV” in its first use in the text on in the Results section, subsection entitled “Gap 2—Barriers to completing the TB diagnostic workup,” sub-subsection entitled “Factors associated with non-pursual of the TB diagnostic workup despite referral in regression analyses,” paragraph 1. Note that we also use “HIV” in tables or figures preceding this initial mention in the text; however, this abbreviation is defined in the table or figure caption.

*ll.308-311: Here, you describe that Table 2 summarizes the framework organizing findings into “people-, family, or society-related factors” and “health system-related factors,” with relevant subcategories. In the column header of Table 2, the specific factors listed are described as “Examples of factors that might be included in a subcategory”. Please provide a full overview of the specific factors included in the subcategories or adjust the column header if Table 2 provides the full set of factors.*

We have tried to provide clarity in this table as follows. First, the header for column 1 is now described as “Subcategories for organizing review findings” and the broader categories into which the various subcategories are organized (i.e., “Patient-, family-, or society-related factors” and “Health system factors”) are included as their own rows and highlighted to indicate that they are larger organizing categories.

Second, the header for column is now described as “Examples of factors included in each subcategory.” We have clarified that this is not an exhaustive list of all possible factors that could be in each subcategory in a footnote: “The factors included in this column are meant to be representative of the types of factors that might be included in each subcategory; they do not represent an exhaustive list of all possible factors within each subcategory.”

We hope this provides clarity for readers.

*l.453: Please define ‘DR’.*

We have opted not to use the abbreviation “DR” for “drug-resistant.” Instead, we have used the expanded term throughout the main text, although we do use an abbreviation for multidrug-resistant (MDR), which is defined at first use.

*l.471: Please define ‘PTLFU’ at first use.*

We now define PTLFU at first use in the Results section, sub-section title: “Gap 3—Barriers contributing to pretreatment loss to follow-up (PTLFU).”

*ll.474-475: Please add the according citation for the different age definitions.*

Note that this sentence is now located in the Results section, subsection title entitled “Gap 3—Barriers contributing to pretreatment loss to follow-up (PTLFU),” sub-subsection entitled “Factors associated with pretreatment loss to follow-up (PTLFU) in adults with drug-susceptible TB.”

We have cut reference to the age definition in the unadjusted analysis, as we now no longer report unadjusted analysis findings in the main manuscript text, as outlined further below in our response to reviewers.

*l.511: Please define ‘DOT’.*

We have now defined “DOT” at first use in the Results section, subsection entitled “Gap 3—Barriers to treatment initiation for people diagnosed with TB disease,” sub-subsection entitled “Reasons for PTLFU in adults with drug-susceptible TB,” paragraph 2.

*ll.554-556: Please add the according citation for the different age definitions.*

After our extensive re-organization of Gap 4 in the Results section, this request is no longer relevant. These citations are now part of a larger set of citations in the Results section under the sub-section entitled “Gap 4—Barriers to treatment success among people who start TB treatment” and the sub-subsection entitled “Demographic factors contributing to unfavorable treatment outcomes in people with drug-susceptible TB.” Specifically, there are now 15 studies that compare older age categories to younger age categories, and we have cited these studies and refer readers to the Forest plot in the supplemental appendix to explore these findings further:

“Across 51 studies reporting adjusted analyses including people with drug-susceptible TB, male sex and older age were associated with unfavorable treatment outcomes in 15 [41,66–79] and 15 [68,69,72,75–86] studies, respectively (Fig I in the S6 Appendix and Table D in the S4 Appendix).”

*ll.682-684: Please change “People with HIV, or with unknown HIV status, had higher unfavorable outcomes in three studies [93,96,98], as was unknown diabetes status (versus not having diabetes) [96].” to “People with HIV or unknown HIV status had higher unfavorable outcomes in three studies [93,96,98], as had people with unknown diabetes status (versus not having diabetes) [96].”.*

We have made this change in the Results section under the sub-section entitled “Gap 4—Barriers to treatment success among people who start TB treatment,” sub-section entitled “Clinical factors contributing to unfavorable treatment outcomes in people with drug-susceptible TB,” paragraph 4. Notably, given an increased number of studies, we have separated these findings regarding people with HIV and diabetes into two different sentences in that paragraph.

*l.754: Please ensure to consistently use ‘kilograms’ or ‘kg’ throughout your manuscript.*

We now consistently use the full work “kilograms” throughout the manuscript, except in Forest plots. For Forest plots, the “kg” is defined in the figure captions.

*l.810: Some readers may not be familiar with the Saint George's Respiratory Questionnaire, please add some details to make it accessible to a wide audience.*

We have now provided details in the Results section, sub-section entitled “Gap 5—Barriers to achieving recurrence-free survival after TB treatment,” sub-subsection entitled “Factors associated with post-treatment TB recurrence,” paragraph 1. In that paragraph, we describe the Saint George’s Respiratory Questionnaire as a questionnaire that “assesses respiratory health in obstructive airways disease.”

**Discussion**

**Please present and organize the Discussion as follows: a short, clear summary of the article's findings; what the study adds to existing research and where and why the results may differ from previous research; strengths and limitations of the study; implications and next steps for research, clinical practice, and/or public policy; one-paragraph conclusion. Please remove any subheadings.**

We have revised the Discussion to ensure that includes all of these recommendations above. In particular, we had not described “strengths” of the study, and we have also expanded on the limitations of the study. At the same time, we also shortened the Discussion section considerably to make it more accessible to the reader.

We should note that, given range of findings we discuss in this section, we felt that the subheadings make the Discussion section more accessible and reader-friendly. However, the way that it is currently written, these subheadings can easily be removed if the editors feel strongly, and we would be happy to do so if requested. **ll.870-872 suggest: “Of concern across all gaps in the care cascade is the dearth of studies on children and the private sector, where about half of Indians with TB receive care.”**

We have made this change in the Discussion section, paragraph 1.

**ll.887-888 suggest: “Some TB subpopulations are at higher risk of unfavorable outcomes across multiple stages of the care cascade.”
ll.887-888: At the end of the statement, it seems you mistakenly cited Table 3 and Table 4 instead of Table 4 and Table 5. Please revise.**

Thank you for pointing that out. We have updated this in the Discussion section, sub-section entitled “Continuities in risk across care cascade stages,” paragraph 1 by taking out reference to these tables. Reference to these tables has now been moved to the Results section, and the tables themselves are no longer in the main manuscript. Based on the feedback from the editors and other reviewers as well, we have moved these tables to the S6 Appendix as extended results to provide a high-level summary of findings.

**l.1074: change ‘heath system’ to ‘health system’.**

We have changed this.

**Table 4/5: Please remove Tables 4 and 5 from the Discussion, as new results should not be discussed first in the Discussion. We suggest moving these tables to the Appendix, given the length of your manuscript and the fact that the data presented appear to have been "summarized" earlier in your manuscript. In addition, we have found that the colors make the tables difficult to read. We suggest using asterisks instead of colors to indicate the study numbers included.**

We appreciate this feedback and have done the following:

(1) We have created a new (very brief) sub-section in the Results section after presenting findings across all 5 care cascade gaps. This sub-section is entitled “Summary of common or important findings across care cascade gaps.”
(2) In that section, we reference the prior Tables 4 and 5, which are now labeled Tables B and C in the S6 Appendix. As such, these tables are no longer presented in the discussion section, and they are no longer in the main manuscript, though they are still available to readers as useful summary tables.
(3) We have removed the colors from these two tables.
(4) In response to Reviewer 3’s feedback, we have included a figure that provides a unified conceptual framework and qualitative summary of findings to disseminate these findings in a manner that is easy for readers to understand. This is placed in the Results section, under the sub-section entitled “Summary of common or important findings across care cascade gaps.” We believe that this figure is a much more reader-friendly summary, and greatly enhances the accessibility of findings from this manuscript.

FIGURES

**Please provide titles and legends for all figures (including those in Supporting Information files).**

**For all Figures, please ensure that you have complied with our figures requirements** [**http://journals.plos.org/plosmedicine/s/figures**](http://journals.plos.org/plosmedicine/s/figures)**.**

We have addressed this, including running these images through the PACE system.

**Please consider avoiding the use of red and green in order to make your figure more accessible to those with colour blindness.**

We have taken out the colors.

**Please ensure to define abbreviations used in your figures.**

**Please indicate in the figure caption the meaning of the whiskers and the arrowhead.**

We have addressed this.

**For some figures, we noted that there is an overlap of text and whiskers (e.g., Figure 9). Please revise and ensure to have enough space for each column and/or add a line break when necessary (including the Figures in the Supporting Information files).**

We have removed all overlaps of text and whiskers in all figures, including those in the supplementary appendices. All figures were reproduced for this revision.

**Figure 3: Please define ‘PTLFU’.**

PTLFU is now defined in the title to this figure and in the figure caption (expansion of abbreviations used in the figure).

**Figure 3/5/6/8/9/10: Please mark the vertical line as ‘1’ in your Figure or add ‘(vertical line)’ behind ‘1’ in your Figure description.**

Vertical lines have been included behind “1” in all Forest plots.

**Figure 3/4/5/6/7/8/9/10: Please indicate the meaning of the information in brackets following the study (Author, year).**

For all figures, the meaning of all information in brackets is now included in the figure captions, usually with language such as “Study labels indicate:…”

**Figure 4: Please add a header for the third column as “%” is not sufficient.**

For relevant Forest plots reporting percentages, we have now used the expanded word “Percent (95% CI)” for clarity.

**Figure 5: For Factor ‘Age >=45 vs. <45’, please add the unit ‘years’.**

This information is now in Fig I in the S6 Appendix. We have included “years” next to all age-related information in all Forest plots.

**Figure 5: You listed ‘[c] outcome is any unfavorable treatment outcome’ in your Figure description while it is not to be found in the Figure itself. Please revise.**

This has been corrected, though this information has been moved to a different figure as noted below.

**Figure 6: When age is listed, please add a unit e.g., ‘years’.**

We have corrected this, though the information is in a new figure.

**Figure 6: Please define ‘DOT’, ‘HIV’, ‘kg’. Please define the meaning of the asterisks.**

We have corrected this, though the information is in a new figure.

**Figure 7: Please define ‘DOT’.**

We have corrected this, though the information is in a new figure.

**Figure 7: Please explain the meaning of the ‘[a]’ and ‘[b] in your Figure description.**

We have corrected this, though the information is in a new figure.

**Figure 7: Please add a unit (%) for ‘Proportion’.**

We have corrected this, though the information is in a new figure. For all Forest plots reporting proportions, we have changes the last column title to “Percent” and included percentage signs along the x-axis for clarity.

**Figure 7: We noted that you used a vertical line to indicate ‘0%’ in your Figure. In**

**Figure 4, you did not use a vertical line to indicate ‘0%’. Please match the presentation style in both figures.**

We have now eliminated use of vertical lines at 0% for all figures reporting percentages.

**Figure 8: When age is listed, please add a unit e.g., ‘years’.**

We have corrected this, though the information is in a new figure.

**Figure 8: Please define ‘LPA’.**

We have corrected this, though the information is in a new figure.

**Figure 8: Please explain the meaning of the ‘[i]’ and ‘[j] in your Figure description.**

**Please note that you listed ‘[g]’ twice. If you want to add further details to ‘[g]’, please use an asterisk.**

We have corrected this, though the information is in a new figure.

**Figure 8: You listed ‘OR, odds ratio’ under the abbreviations in your Figure description, but it is not abbreviated in your Figure. Please remove.**

We have corrected this, though the information is in a new figure.

**Figure 9: Please define ‘CI’.**

We have corrected this, though the information is in a new figure. CI has been added as an abbreviation to all figures.

**Figure 10: Please define ‘kg’.**

We have corrected this, though the information is in a new figure.

**Figure 10: The labels ‘[d]’ and ‘[e]’ are not present in the Figure itself. Please check and revise.**

We have spelled out these abbreviations and defined the meaning of the asterisks.

Please note that figures for gap 4 (Fig 5- 9) have been completely modified per reviewers and editors’ feedback. Particularly after our updates of the search to August 2023 resulted in the inclusion of about 40 additional studies in Gap 4, we realized that combining the analyses for these 3 populations (people with new TB, people with previously treated TB, and people with either new or previously treated TB) results in greater clarity and impact for the reader. All 3 of these populations are now collectively referred to as “people with drug-susceptible TB,” but we have still been very careful to designate findings from each specific population using footnotes to the study names in the Forest plots. This feedback resulted in substantial reorganization of the Gap 4 Results section. Given the large volume of findings across these 3 subpopulations of people with drug-susceptible TB, we have now divided reporting of Results for this population into the following sub-sections: (1) “Demographic factors contributing to unfavorable treatment outcomes in people with drug-susceptible TB”; (2) “Clinical factors contributing to unfavorable treatment outcomes in people with drug-susceptible TB”; (3) “Socioeconomic, psychosocial, and family- or society-related barriers contributing to unfavorable treatment outcomes in people with drug-susceptible TB”; (4) “Health system barriers contributing to unfavorable treatment outcomes in people with drug-susceptible TB.”

TABLES

**Please provide titles and legends for all tables (including those in Supporting Information files).**

We have addressed this.

**Please consider avoiding the use of red and green in order to make your tables more accessible to those with colour blindness.**

We have taken out the colors.

**Please ensure to define abbreviations used in your tables.**

We have addressed this.

**Table 1: Please define ‘HIV’, ‘NTEP’.**

We have defined HIV and removed “NTEP” from the table.

**Table 1: In the ‘Specific populations and outcomes assessed’ of Gap 5, we suggest deleting ‘[…], in Gap 5’ at the end of the sentence.**

We have corrected this. **Table 1: Please define ‘TB’, ‘HIV’.**

I believe you meant Table 2, we have defined these abbreviations under footnotes of Table 2.

**Table 4: Please define ‘TB’, ‘HIV’.**

Table 4 is now moved to S6 appendix as Table B. We have defined these abbreviations under footnotes.

**Table 4: Please add a definition or range for ‘Older Age (N=23)’.**

Table 4 is now moved to S6 appendix as Table B. Various cutoffs were mentioned for older age, including increase per year as a continuous variable. Hence, we cannot include a definition or range.

**Table 5: Please define ‘TB’.**

Table 5 is now moved to S6 appendix as Table C. We have defined this abbreviation under footnotes.

APPENDICES

**Suggest reporting statistical information as detailed above – see under ABSTRACT

S1 Appendix
l.151: Please change ‘citations’ to ‘citation’.**

We have corrected this.

**ll.256-257: Please change ‘We did not conduct meta-analyses of data, because we extracted findings represented a diverse set of variables from every study.’ to ‘We did not conduct meta-analyses of data, because we extracted findings representing a diverse set of variables from every study.’.**

We have addressed this. **Figure A: Please define ‘TB’ in the title.**

We have addressed this. **Table C: Please note that in some of the column headers you specify '(%)' as the unit (column 6), while in others you do not (column 9). If a unit is specified in the column header, the numbers in the corresponding column may stand alone. Please revise.**

We have updated n (%) in column 9. That way, the percentages in both columns were removed and numbers have stood alone.

**Table D: Please define ‘HIV/AIDS’.**

We have defined this abbreviation under footnotes.

**Table D: For the studies of ‘KHPT/THALI project 2016-17 (Telangana)f [9]’ and ‘KHPT/THALI project 2016-17 (Karnataka)f [10]’, please add a currency for ‘Personal monthly income’ and ‘Household monthly income’.**

We have added ‘in Indian rupees’.

**S2 Appendix

l.71: Please define ‘HIV’.**

We have defined this.

**Table A: Please define ‘TB’ in the title.**

We have addressed this and updated the title to ‘….individuals with presumptive tuberculosis (TB).’

**ll.193-194: Please change ‘As describe above,[…]’ to ‘As described above,[…]’.**

We have corrected this.

**Figure A: Please define ‘TB’ in the title.**

We have addressed this and updated the title to ‘…. people with tuberculosis disease’

**Table B: Please define ‘TB’ in the title.**

We have updated the title to ‘…. individuals with presumptive tuberculosis (TB)’

**ll.339-340: Please change ‘We did not conduct meta-analyses of data, because we extracted findings represented a diverse set of variables from every study.’ to ‘We did not conduct meta-analyses of data, because we extracted findings representing a diverse set of variables from every study.’.**

We have addressed this.

**Table C: Please introduce the abbreviation ‘TB’ in the title. Please define ‘DR’, ‘RR’.**

We have defined these abbreviations.

**Table D: Please define ‘HIV’, ‘DMC’, ‘km’, ‘MDR’, ‘LTFU’, ‘HIV’, ‘PHC/CHC’
Table D: For ‘Kanakaraju 2020 (Karnataka)b,d [12]’, ‘Shewade 2017 (Madhya Pradesh)a [16]’, ‘Shewade 2017 (Tamil Nadu)a [17]’, ‘Shewade 2016 (Puducherry)a [19]’, ‘Shewade 2015 (Puducherry)a [18]’ , please add a unit for age.
Table D: Please note that there is no definition for the asterisk.**

We have addressed this.

**S3 Appendix

Table A: Please introduce the abbreviation ‘TB’ in the title.**

We have introduced this abbreviation in the title.

**Figure A: Please introduce the abbreviation ‘TB’ in the title.**

We have addressed this and updated the title to ‘…. people with tuberculosis disease’

**l.213: Please define ‘DMC’.**

**l. 250: Please change ‘Cis’ to ‘CIs’.
ll.273-275: Please change ‘We did not conduct meta-analyses of data, because we extracted findings represented a diverse set of variables from every study.’ to ‘We did not conduct meta-analyses of data, because we extracted findings representing a diverse set of variables from every study.’.**

We have made these changes.

**Table C: Please introduce the abbreviation ‘TB’ in the title.**

We have addressed this.

**Table D: Please introduce the abbreviation ‘TB’ in the title.
Table D: Please define ‘PTLFU’, ‘N/A’, ‘HCP’, ‘KM’ (should be ‘km’), ‘HIV’, ‘MDR’.
Table D: If income is listed, please add a currency. If age is given, please add a unit.
Table D: Please note that there is no definition for the asterisk.**

We have made these changes.

**S4 Appendix

Please note that currently there are no references for S4 Appendix.**

Thank you for pointing this out. We have added references.

**l.79: Please define ‘HIV’.**

We have defined this.

**Figure A: Please introduce the abbreviation ‘TB’ in the title.**

We have addressed this and updated the title to ‘…. people with tuberculosis disease’

**Table B: Please define ‘TB’ in the title.**

We have updated the title to ‘…..evaluating barriers to treatment success in people diagnosed with tuberculosis (TB) in India’.

**ll.347-348: Please change ‘We did not conduct meta-analyses of data, because we extracted findings represented a diverse set of variables from every study.’ to ‘We did not conduct meta-analyses of data, because we extracted findings representing a diverse set of variables from every study.’.**

We have made these changes.

**Table C: Please define ‘TB’, ‘HIV’**

We have defined these abbreviations.

**Table D: Please introduce the abbreviation ‘TB’ in the title.
Table D: Please define ‘km’, ‘OBC’, ‘kg’, ‘SES’, ‘DOT’, ‘PHI’, ‘SAMVAD’, ‘BMI’, ‘IP’, ‘LTFU’, ‘HIV’, ‘ATT’, ‘ART’, ‘INH’, ‘TAD’, ‘RNTCP’, ‘NGO’, ‘S’, ‘H’, ‘E’, ‘MDR’, ‘XDR’, ‘CBNAAT’, ‘CDST’, ‘CP’, ‘AUDIT-C’, ‘AFB’, ‘SC/ST/NT’, ‘IQR’, ‘STS’, ‘INP’, ‘DMC’, ‘CD4’, ‘CPT’.
Table D: Please use ‘Sex’ instead of ‘Gender’.
Table D: For ‘Bagchi, 2010a (Maharashtra)’, please change ‘earlly’ to ‘early’ (Category ‘Knows about problems with stopping treatment earlly’).
Table D: If income is listed, please add a currency. If age is given, please add a unit.**

We have addressed these changes.

**S5 Appendix

Table B: Please define ‘CBNAAT’.**

We have defined this.

**Table C: Please introduce the abbreviation ‘TB’ in the title.
Table C: Please define ‘ATT’, ‘HIV’
Table C: If age is described, please add a unit (e.g., the unit is missing for Kolappan (2006) [14] and Kolappan (2008) [15]).**

We have addressed these changes.

**Table D: Please introduce the abbreviation ‘TB’ in the title.
Table D: Please define ‘‘HIV’, ‘COPD’, ‘kg’, ‘H’, ‘HR’, ‘ART’, ‘DM’
Table D: Please use ‘Sex’ instead of ‘Gender’.
Table D: If income is listed, please add a currency. If age is given, please add a unit.**

We have addressed these changes.

**Table D: Please note that in Table D, ‘SGRQ4’ is not addressed in the footnotes. Please revise.**

The ‘4’ in SGRQ4 scores was written in error and we have updated it to SGRQ scores, thank you for pointing that.

**S6 Appendix

Please ensure to cite all figures in the supplementary text.**

We have ensured to cite all figures in the supplementary text under footnotes.

**Please define ‘TB’ at first use.**

We have defined this.

**l.109: Please define ‘NAAT’.
l.268: Please define ‘RR’, ‘MDR’ and ‘HIV’ at first use.**

We have defined these abbreviations.

**Figure A/B/F/J: Please add a unit for age.

Figure A/B/C/D/E/F/G/H/I/J/K/L/M:** **Please indicate in the figure caption the meaning of the whiskers and for clarity, add ‘(vertical line)’ behind ‘1’ (‘0’ for Figure I/K/L) in your Figure description if applicable.

Figure A/B/C/D/E/F/G/H/I/J/K/L/M:** **Please indicate the meaning of the information in brackets following the study (Author, year).

Figure C/E/G/H: Please add a header for the third column as “%” is not sufficient (e.g., ‘Proportion’).

Figure A: Please define ‘HIV’.**

We have addressed these changes.

**Figure A: Please note that there is still a comment from one of the authors (RS) left on Figure A.**

Thank you for pointing it out, we have rectified this.

**Figure B: Please note that for ‘adjusted odds ratio’ the abbreviation should be listed as ‘aOR’.**

We have addressed this.

**Figure C: Please introduce the abbreviation ‘TB’ in the title.**

We have defined this.

**Figure F: Please define ‘TB’.**

We have defined this.

**Figure G: Please define ‘NTEP’, ‘DST’, ‘DR’, ‘TB’, ‘CBNAAT’, ‘MDR’.**

We have defined these abbreviations.

**Figure I. Please define ‘DOTS’.
Figure I: Please define the meaning of the arrowhead.**

We have added the following sentence to the footnote: “The arrowhead means that the upper limit of CI extends beyond the end of x-axis.”

**Figure I: Below the heading ‘Socioeconomic status’ there appears to be a misplaced footnote.**

**Figure J: Please define ‘HIV’, ‘DOT’, ‘kg’.**

**Figure J: Please define the meaning of the arrowhead.**

**Figure J: Please check whether the abbreviation ‘OR, odds ratio’ is necessary.
Figure J: Please define ‘DOTS’.**

**Figure L: Please define ‘TB’.**

**Figure L: Please add the definitions for ‘[a]’, ‘[b]’, and ‘[c]’.**

**Figure M: Please define the meaning of the arrowhead.**

**Figure M: Please add the definitions for ‘[a]’ and ‘[b]’.**

**Figure M: Please define ‘HIV’, ‘ART’, ‘CD4’**

**Figure M: Please add a unit for the CD4 count.**

**Figure M: Please check whether the abbreviation ‘OR, odds ratio’ is necessary.**

**Figure M: Is there a reason, you decided to not add the information in brackets (study setting) following author and year?**

We have spelled out these abbreviations and made necessary changes as requested.

Please note that figures for gap 4 (Fig I, J, K, L, M and N) have been completely modified per reviewers and editors’ feedback. Particularly after our updates of the search to August 2023 resulted in the inclusion of about 40 additional studies in Gap 4, we realized that combining the analyses for these 3 populations (people with new TB, people with previously treated TB, and people with either new or previously treated TB) results in greater clarity and impact for the reader. All 3 of these populations are now collectively referred to as “people with drug-susceptible TB,” but we have still been very careful to designate findings from each specific population using footnotes to the study names in the Forest plots. This feedback resulted in substantial reorganization of the Gap 4 Results section. Given the large volume of findings across these 3 subpopulations of people with drug-susceptible TB, we have now divided reporting of Results for this population into the following sub-sections: (1) “Demographic factors contributing to unfavorable treatment outcomes in people with drug-susceptible TB”; (2) “Clinical factors contributing to unfavorable treatment outcomes in people with drug-susceptible TB”; (3) “Socioeconomic, psychosocial, and family- or society-related barriers contributing to unfavorable treatment outcomes in people with drug-susceptible TB”; (4) “Health system barriers contributing to unfavorable treatment outcomes in people with drug-susceptible TB.”

**For Gap 5, there are currently no additional Figures presented – is this correct?**

Yes, this is correct, there were no figures in the supplementary appendices for Gap 5.

REFERENCES

**PLOS uses the numbered citation (citation-sequence) method and first six authors, et al.

Please ensure that journal name abbreviations match those found in the National Center for Biotechnology Information (NCBI) databases (**[**http://www.ncbi.nlm.nih.gov/nlmcatalog/journals**](http://www.ncbi.nlm.nih.gov/nlmcatalog/journals)**), and are appropriately formatted and capitalised.

Please also see** [**https://journals.plos.org/plosmedicine/s/submission-guidelines#loc-references**](https://journals.plos.org/plosmedicine/s/submission-guidelines#loc-references) **for further details on reference formatting.

Where website addresses are cited, please specify the date of access.**

We have addressed this.

**RESPONSES TO REVIEWER COMMENTS**

**Reviewer #1:**

*This paper presents a review of studies of engagement in the care cascade in*

*India. I was asked for a statistical report and I interpret that to include all aspects*

*of the design and conduct of the study.*

**Points of detail.**

*Page 11 and 12 The final search date is now just over two years ago which does seem rather a long delay. An update would improve matters I*

*appreciate that this review took a very considerable amount of work*

*but there are eighteen authors to share the burden of an update.*

We appreciate and agree with this feedback. We have conducted a refresher search that has updated the manuscript through August 14, 2024. This refresher search identified 47 new papers that were eligible for inclusion, which now contribute to the review findings. The inclusion of these papers involved considerable work over the last few months, but we believe it has improved the systematic review as suggested by Reviewer 1.

*Page 12 I suppose the treatment as usual arms of clinical trials might be thought relevant. The treatment arms are clearly not relevant.*

We agree that the treatment arms of clinical trials are not relevant for this review; however, we also have concerns about the control arms of clinical trials. Clinical drug trials in TB are meant to isolate the potential benefits of a new drug regimen. As such, under trial conditions, high retention is usually a goal in both study arms, and this usually involves efforts (e.g., financial compensation, social work support, etc.) that are not available in routine programmatic care. As the goal of this review is to understand factors associated with unfavorable TB outcomes in “real world” conditions, we feel that the control arms of clinical trials also vary substantially from what happens in routine care. As such, we have still excluded these studies in the revised manuscript.

*Figure 7 The confidence intervals are asymmetric, which is correct, but I*

*did not see anywhere what method was adopted to generate them.*

We apologize for this oversight in providing details regarding how we estimated the confidence intervals for proportions in these Forest plots. In the Methods section (subsection on “Data extraction,” paragraph 1, last sentence), we have now clarified that, when confidence intervals were not reported by the source paper, the approach to generating confidence intervals for Figure 7 (and similar figures) was the binomial “exact” method, assuming an infinite population size. We have also cited the online calculator that we used to estimate confidence intervals for proportions throughout the paper:

“When 95%CIs were not reported, we estimated these using the binomial “exact” method, assuming an infinite population size [20].”

**Point of more substance**

*Page 14 informs us that only statistically significant results are presented*

*and this is confirmed in the captions of the figures. I find it hard to see why,*

*in 2023, anyone thought this was a good idea. The point of a systematic*

*review is to present to us the totality of evidence and then to draw from it*

*some form of conclusion. It is interesting to look at the example of the use of*

*streptokinase in acute myocardial infarction (Lau et al., 1992) where the first*

*20 trials include 17 trials which the authors would not have presented and*

*yet the meta-analysis up to that point produced an almost identical result*

*to that following the first large trial (GISSI-1). I would regard presenting all the evidence as a mandatory improvement.*

*References*

*J Lau, E M Antman, J Jimenez-Silva, B Kupelnick, F Mosteller, and T C*

*Chalmers. Cumulative meta-analysis of therapeutic trials for myocardial*

*infarction. New England Journal of Medicine, 327:248{254, 1992.*

We greatly appreciate this critical feedback from the reviewer. We are in full agreement with this feedback, which was also provided by Reviewer 2, and we feel this feedback has been critical to improve the scientific quality of the manuscript.

In response, for any variables with statistically significant findings in any adjusted analyses, we have now also reported all studies that provide non-significant findings for those same variables in any adjusted analyses in the Forest plot captions and in the main text of the Results section. This has resulted in changes throughout the Results section and in the captions of nearly all Forest plots reporting findings from regression analyses. Please note that, for a few Forest plots, especially in Gaps 2 or 3, there were no studies reporting non-significant findings for variables included in the Forest plot, so these additions have not been made to these Forest plot captions.

We have also clarified this modification in our reporting approach in the Methods section (subsection on “Data extraction,” paragraph 2):

“For exposure variables with statistically significant findings, we also reported any non-significant adjusted effect estimates from included studies for these same variables in the main text and Forest plot captions.”

The inclusion of non-significant findings not only provides readers with the totality of the evidence for relevant exposures, but it could also facilitate meta-analyses of findings for selected exposures in the future, if readers wish to pursue such analyses. We did not perform meta-analyses in this manuscript for scientific reasons (e.g., heterogeneity was high for most exposures) and because there are active debates in the field of implementation science regard the extent to which findings can be meta-analyzed from diverse real world settings, even as the common trends are very helpful for intervention development within TB programs.

We are grateful to Reviewers 1 and 2 for this feedback, as we strongly agree that this was an important methodological issue. We hope that this concern has been addressed sufficiently in the revised manuscript.

**Reviewer #2:**

*Thank you for the invitation to review this very interesting article on the TB treatment cascade in India. The authors have used a novel, ambitious approach to describing the risk factors for negative outcomes at all stages of the care cascade. This has clearly been a significant undertaking and the results are comprehensive and revealing. Major and minor comments are listed below.*

We appreciate this feedback from Reviewer 2. Our goal was indeed to use systematic review methods but to use a novel approach and frameworks to understand barriers experienced by people with TB across all stages of India’s care cascade. Our attempt at a comprehensive approach is aimed at providing information that would be useful to TB program managers, researchers, and funders.

**Major comments**

*1. The authors have decided to only present and report on "statistically significant adjusted effect estimates, as these may represent associations from higher-quality analyses" (lines 298-300). I am not sure I agree with the justifying assertion. I appreciate the difficulties in synthesizing and presenting large amounts of information, but only including significant effects will misleadingly inflate effect size. For example, hypothetically if three studies have shown that male gender increases the risk of an adverse outcome, but several further studies have shown no significant effect of gender on outcome, this would be a relevant negative finding to report. This seems particularly relevant for studies with conflicting effect directions seen (e.g. diabetes, employment), but applies to all gap analyses and all Forest plots.*

We greatly appreciate this critical feedback from the reviewer. We are in full agreement with this feedback, which was also provided by Reviewer 1, and we feel this feedback has been critical to improve the scientific quality of the manuscript.

In response, for any variables with statistically significant findings in any adjusted analyses, we have now also reported all studies that provide non-significant findings for those same variables in any adjusted analyses in the Forest plot captions and in the main text of the Results section. This has resulted in changes throughout the Results section and in the captions of nearly all Forest plots reporting findings from regression analyses. Please note that, for a few Forest plots, especially in Gaps 2 or 3, there were no studies reporting non-significant findings for variables included in the Forest plot, so these additions have not been made to these Forest plot captions.

We have also clarified this modification in our reporting approach in the Methods section (subsection on “Data extraction,” paragraph 2):

“For exposure variables with statistically significant findings, we also reported any non-significant adjusted effect estimates from included studies for these same variables in the main text and Forest plot captions.”

The inclusion of non-significant findings not only provides readers with the totality of the evidence for relevant exposures, but it could also facilitate meta-analyses of findings for selected exposures in the future, if readers wish to pursue such analyses. We did not perform meta-analyses in this manuscript for scientific reasons (e.g., heterogeneity was high for most exposures) and because there are active debates in the field of implementation science regard the extent to which findings can be meta-analyzed from diverse real world settings, even as the common trends are very helpful for intervention development within TB programs.

We are grateful to Reviewers 1 and 2 for this feedback, as we strongly agree that this was an important methodological issue. We hope that this concern has been addressed sufficiently in the revised manuscript.

*Similarly, throughout the text of the results section it is often unclear whether an effect has been observed consistently across all studies, in the majority of the studies where it has been examined, or just in a single study (e.g. lines 406-408 & throughout.)*

*Where the number of studies is quoted (e.g. lines 474-475, "In one adjusted and two unadjusted analyses, older age […] was associated with higher PTLFU") it is not clear whether the remaining studies did not report on age, or they did but found no effect. It would be clearer to say "Of XX analyses which reported on age, one adjusted and two unadjusted analyses found that older age was associated with higher PTLFU, while the remaining YY found no significant association of age with LTFU" (and to show this in the forest plot.) It might also be informative to start each results section with "overall, data from ZZ studies contributed to this analysis." Again, this comment applies across all results sections.*

Thank you for this important feedback, which we have now addressed in a few different ways with meaningful modifications to the manuscript. First, given the reviewer’s concerns about understanding the total number of studies contributing to a given finding, we have included Table 3, which summarizes the total studies included in each gap. This table also summarizes the number of studies addressing each subpopulation within each care cascade gap in the footnotes. Note that, given the length of the manuscript, and strong encouragement by the editors to reduce the word count, we shifted narrative information on the characteristics of the included studies to the S6 Appendix; however, we feel that the new Table 3 provides a more efficient and reader-friendly snapshot regarding characteristics of the included studies.

Second, due the manuscript’s length and scientific considerations, we have now removed reporting of unadjusted findings from the main text of the manuscript. While unadjusted findings can sometimes provide supportive evidence on important trends in the findings, we felt that studies presenting only unadjusted findings generally were of lower quality and the analyses themselves are less likely to be identifying causal associations. Note that the total number of studies with findings reported in the main manuscript has been revised in the Abstract and Table 3 to reflect the exclusion of unadjusted findings from the main manuscript. However, we still report all unadjusted findings in the supplementary appendices and summarize characteristics of all of these studies in Table A in the S6 Appendix.

Third, in response to the reviewer’s first concern raised above, we now report and cite all non-significant findings in the text for any exposure variable that had significant findings (and therefore got reported in a Forest plot). By reporting both significant and non-significant findings on specific exposures in the text, this also provides information to the reader regarding the total number of studies that conducted adjusted analyses that included a given exposures variable. We also start each subsection with a summary of the total number of adjusted analyses that contributed findings to a given subsection or Forest plot.

*2. The conceptualization of the PECO for some of the Gaps was unclear to me, and from Table 1 and the text I found it hard to understand exactly what kinds of studies were being synthesized.*

*Gap 1 - The population was difficult to understand without reading the appendix ("People with confirmed TB or presumptive TB in the community who do not reach care at facilities" - how were they identified then?). It would be useful to explicitly state e.g. in Table 1 that (if I understand correctly!) these are people identified through cross-sectional population-based surveys, who either 1) have TB symptoms, or 2) are ultimately diagnosed with confirmed TB, and report that they have not previously sought care. For people with confirmed TB identified through prevalence surveys/ACF, clarify whether only symptomatic participants were included, or would participants with subclinical TB (who have no reason to seek care) also be captured?*

Your feedback made us realize that we needed to communicate details about the population included in Gap 1 with greater clarity in the main manuscript. In our systematic search process, our goal was to identify cross-sectional, population-based studies that either: (1) identified people with symptoms concerning for TB disease OR (2) that identified people with symptoms, confirmed those that had bacteriological TB, and then reported information on individuals with bacteriological TB who had or had not sought care at the time of the initial survey. The latter type of study is possible and can be implemented during population-based TB prevalence surveys, for example.

However, after our systematic search, all 15 studies that ultimately got included in Gap 1 comprised cross-sectional, population-bases surveys that identified people with TB symptoms. As such, for simplicity and to provide clarity for the reader, we have simplified our description of the population included in Gap 1 in the main manuscript Table 1, with the following modified text, which also clarified that all individuals included in Gap 1 had TB symptoms (i.e., people with subclinical TB were excluded):

Gap 1 Definitions: “People with symptoms of TB disease in the community who did not reach care and start the diagnostic workup.” (Table 1, Column 2)

Gap 1 Populations and outcomes assessed: “We included studies of people in the community identified through cross-sectional, population-based surveys, who had TB symptoms. Because these surveys started with symptom screening, studies only included symptomatic individuals.” (Table 1, Column 3)

*Gap 4 - The designation "multiple populations of people with TB" (Table 1 and in text) could be defined earlier ("undifferentiated"? - not immediately clear that it includes both those with new and previous TB). The authors have divided studies into those dealing exclusively with new DS-TB, those only including people previously-treated DS-TB, and those including both new and previous cases. This section (lines 547-713) is lengthy and the majority of risk factors are identical across all three analyses (male sex, older age, lower SES, lower education, employment, stigma, advanced disease, low BMI, alcohol, smoking, poor healthcare experience, logistical barriers to accessing care) which leads to repetition and obscures the consistency of key findings. Reported reasons (work constraints, mobility, side effects, early symptom resolution and health system factors) are similarly shared. For clarity and impact, have the authors have considered combining these three analyses, or at least "multiple populations" - most of whom will have new DS-TB - with those with new DS-TB? (Disaggregated subgroup results could be moved to an appendix.)*

We greatly appreciate this feedback and have taken the reviewer’s advice. Particularly after our updates of the search to August 2023 resulted in the inclusion of about 40 additional studies in Gap 4, we realized that combining the analyses for these 3 populations (people with new TB, people with previously treated TB, and people with either new or previously treated TB) results in greater clarity and impact for the reader. All 3 of these populations are now collectively referred to as “people with drug-susceptible TB,” but we have still been very careful to designate findings from each specific population using footnotes to the study names in the Forest plots.

This feedback resulted in substantial reorganization of the Gap 4 Results section. Given the large volume of findings across these 3 subpopulations of people with drug-susceptible TB, we have now divided reporting of Results for this population into the following sub-sections: (1) “Demographic factors contributing to unfavorable treatment outcomes in people with drug-susceptible TB”; (2) “Clinical factors contributing to unfavorable treatment outcomes in people with drug-susceptible TB”; (3) “Socioeconomic, psychosocial, and family- or society-related barriers contributing to unfavorable treatment outcomes in people with drug-susceptible TB”; (4) “Health system barriers contributing to unfavorable treatment outcomes in people with drug-susceptible TB.”

We thank the reviewer for this feedback, which has greatly improved the clarity and impact of the presentation of findings for people with drug-susceptible TB.

*3. "Reasons" for care
I find figures 2, 4 and 7 less intuitive. I appreciate that meta-analysis has not been performed for the effect sizes in Figure 1 etc., but as these are displaying simple proportions is there any scope to pool estimates here, and/or present data in more synthesized form? If Forest plot are maintained, I would suggest 1) using a consistent 0-100 scale (as in Fig 2 but not in 4 or 7) for consistency and to make it clearer that these are percentages, 2) adding vertical gridlines, at least for 0 and 100% so that the reader can more easily orientate what the proportions are, 3) consider further grouping so that factors which are essentially identical are clearly linked (e.g. in Fig 4, the 5 studies which report "died before treatment initiation" are not readily distinguishable from the one which reports "previous adverse effects", a completely distinct concept.*

In response to this feedback, we have modified these figures in the following manner: (1) all figures on “reasons” for unfavorable outcomes now use a uniform 0 to 100 scale at the bottom; (2) we have denoted that these are percentages both with a “Percent” label over the reporting of the exact numerical estimates and confidence intervals in these plots and by use of a “%” sign after each number along the y-axis; and (3) for some of these plots with a large number of findings, we have included further groupings with subheadings—for example, for Fig L in the S6 Appendix, we have included subheadings for “TB medication adverse effects or treatment burden” and “Symptoms or illness severity during treatment.” We did not include the lines at 0 and 100 as we felt after trying this that it might be more distracting than helpful.

We appreciate this feedback, which has added much greater clarity to these figures and resulted in a more cogent presentation of findings.

*4. Definitions of exposures and outcomes
I am not sure medication non-adherence can be considered an exposure for Gap 4, and it is inconsistent to include it as both an exposure and an outcome (595-596; 1028-1031). Most of the risk factors in Gap 4 will have their effects through non-adherence. Presumably the adjusted analyses have not adjusted for non-adherence? To me it would be more logical to include as an outcome only (although there is a large literature on the risk factors for non-adherence which might not have been specifically captured in this search?), or to exclude. Similarly "lack of sputum conversion" (lines 584-587) is surely not an exposure but a marker of incipient unfavorable outcome, as are post-treatment symptoms (809-811, 832-834) for Gap 5.*

We appreciate this feedback. A challenge in this review is that some exposure variables may have direct causal effects, or serve as mediators, or both. Medication nonadherence is one such example, and there are other examples in which variables serve as both outcomes in the review and as exposures. For example, loss to follow-up (i.e., treatment non-completion) is routinely considered to be an unfavorable outcome within TB programs given perceived associations with poorer long-term health outcomes. However, loss to follow-up is also used as an exposure in studies evaluating TB post-treatment outcomes; for example, in Fig 11 in the revised manuscript, loss to follow-up is associated with elevated post-treatment mortality. What is compelling about including loss to follow-up in our analysis of post-treatment outcomes is that these findings affirm the empirical basis for considering loss to follow-up as an unfavorable on-treatment outcome.

With regard to medication adherence and similar variables, we include it as an outcome given strong perceptions of its importance as a proxy for longer-term unfavorable on-treatment outcomes, but we also feel that it should be included as an exposure given ongoing debates about the association between medication nonadherence (in the absence of treatment-noncompletion) and subsequent TB treatment outcomes. There are also debates regarding different measures of nonadherence patterns and associations with unfavorable TB treatment outcomes (see for example: Stagg, et al. ERJ Open Research 2020 6: 00315-2020). Our findings provide insights relevant to these various debates.

Most importantly, we wanted to comprehensively report on the evidence available regarding exposures and outcomes, without picking and choosing which exposures to report. By doing so, readers are then able to interpret the relative importance of different findings and subsequently decide whether some findings may have direct causal effects, serve as mediators, or both.

5. The discussion is excellent and successfully synthesizes a vast amount of data.

We appreciate this feedback, even as we streamlined the Discussion section a bit in the revised manuscript to reduce the word count.

**Minor comments**

*Line 46/91 - "Most" Indians contracting TB disease do not navigate all stages of the care cascade - this is striking and it would be informative to quantify and reference.*

Reference 5 included in main manuscript describes the TB care cascade for India for 2013, and this analysis found that less than 40% of people with incident TB disease in India completed the care cascade. However, given that the care cascade may have changed since then, and as we cannot cite a reference in the Abstract, we now say that “Many Indians do not navigate all care cascade stages…” in the Abstract.

*Table 3 - where relevant, percentages would be useful.*

We opted not to report percentages in Table 3 for a few reasons. First, it is unclear what the denominator would be for the total number of studies in each gap, as each gap could be considered its own review. Second, for other cells, the denominator would often vary and should ideally represent the total number of studies in a given gap, but this is hard to communicate with clarity without a footnote for each cell. For other rows, such as the “States and union territories covered by included studies” there can be more than one state included in a given study.

Given these complexities, we felt that percentages could be confusing or misleading, and we have opted to avoid inserting percentages in Table 3.

*Line 392 - "a few studies finding about half" - could be more precise*

We have updated this line to be more precise: “…3 studies finding about half of people faced these barriers…” (Results section, sub-section “Gap 1—Barriers contributing to people with TB symptoms in the community not having sought care,” sub-subsection on “Reasons reported by people with TB symptoms for not seeking care,” paragraph 1, sentence 1).

*Line 553 - suggest maintain "unfavorable" rather than "suboptimal" for consistency*

We have now modified the manuscript to always use “unfavorable” (and not “suboptimal”) when referring to adverse treatment outcomes, for consistency.

*Line 776 - Clarify: is this 93% of patients who were LTFU, or 93% of all those starting TB treatment?*

We have revised the manuscript to ensure that all findings regarding “reasons” assume a denominator of people who experience unfavorable outcomes (not all people who started treatment). We have modified the specific sentence noted by the reviewer as follows:

“Across 3 studies surveying people with RR or MDR TB who were lost to follow-up or experienced nonadherence, reasons included: migration out of the area (reported by 93% of people in 1 study [142]), lack of family support (reported by 15% of people in 1 study [143]), and medication adverse effects (reported by 7% to 75% of people across 3 studies [142–144]) (Fig M in the S6 Appendix).”

Throughout all of our descriptions of findings regarding “reasons,” we always note that these surveys were conducted among people with TB who were “lost to follow-up,” or other unfavorable outcomes relevant to that gap, to provide clarity that the denominator comprises those who had unfavorable outcomes in a care cascade gap.

*Line 890-891 - rephrase this sentence?*

This sentence has been removed with the editing and shortening of the Discussion section.

*Table 4 - this would benefit from denominators. i.e. for each Gap, how many studies reported on the exposure, and of those, how many showed a significant effect. The current color-coding scheme based on absolute number of studies is a little misleading, as Gap 4 contains so many more studies than the others - using proportions of studies may be more informative.*

Based on the feedback from the editors and other reviewers as well, we have moved these tables to the S6 Appendix as extended results to provide a high-level summary of findings. We have also removed the colors in the figure. For various reasons, including percentages in this figure may be problematic. While we agree that this better contextualizes data in relation to the number of studies within each gap, it could still be misleading as the total number of studies within a gap that evaluated a given exposure may vary. As such, we still provide readers with this table to provide a helpful summary, but we have reduced its prominence within the manuscript.

In place of these tables, we have added a new figure (Fig 12) to the Discussion section which qualitatively summarizes findings across both types of analyses (i.e., “factors” and “reasons”), which we feel will be more helpful to readers.

**Table 5 - specify "high" severity of TB illness**

We have made this modification. As previously noted, we have moved this table to the S6 Appendix.

**Table 4&5 - is the legend intentionally obscuring the bottom right cell?**

The legend has been removed as colors were removed as requested by the editors.

**Line 1056-1065 -for the general reader, can the authors comment on the generalizability of findings outside of India? Should other countries be performing similar analyses?**

We appreciate this suggestion and have added the following text to the Discussion section, in the first paragraph of sub-section on the “Strengths and limitations of the revew”: “Given that people with TB globally often come from socioeconomic disadvantage, our findings may have relevance for other high TB incidence countries. Our novel approach provides a roadmap for similar analyses to understand reasons for losses in the TB care cascade in other global settings.”

**Line 1086-1087 - "While quality of care is better in the government TB program, client experience is generally better in the private sector" - this does not seem to be fully supported by the data presented (especially the first half of the statement), or to be one of the core conclusions of the study.**

We have deleted this sentence.

**Reviewer #3:**

*In this ambitious systematic review, the authors have brought together two decades worth of data on factors that put Indians at risk of falling through gaps in the cascade of care. The gaps identified by the authors are excellent for conceptualizing the weaknesses in the prevailing system. This paper brings together important insights. I have no major concerns.*

We appreciate this feedback. Our goal was indeed to use systematic review methods but to use a novel approach and frameworks to understand barriers experienced by people with TB across all stages of India’s care cascade. Our attempt at a comprehensive approach is aimed at providing information that would be useful to TB program managers, researchers, and funders.

*I did note that for gaps 4 and 5, some key papers published in the last 2 decades were not included. I have included some of them below. Perhaps the authors would consider including these in their analysis.*

Thank you for noting these articles that were not included in our prior manuscript version. Some of these had not been included as they were not within cut-off date used for our previous search, or because they did not meet inclusion criteria. With the updating of our search and findings through a cut-off date of August 14, 2023, some of these papers are now all included in the review when they met our inclusion criteria. We have provided specific notes on these studies below.

Bhargava A, Chatterjee M, Jain Y, Chatterjee B, Kataria A, Bhargava M, Kataria R, D'Souza R, Jain R, Benedetti A, Pai M. Nutritional status of adult patients with pulmonary tuberculosis in rural central India and its association with mortality. PloS one. 2013 Oct 24;8(10):e77979.

This paper was already included in our prior manuscript version and continues to be included, with findings reported in the main manuscript.

Sinha P, Ponnuraja C, Gupte N, Prakash Babu S, Cox SR, Sarkar S, Mave V, Paradkar M, Cintron C, Govindarajan S, Kinikar A. Impact of Undernutrition on Tuberculosis Treatment Outcomes in India: A Multicenter, Prospective, Cohort Analysis. Clinical Infectious Diseases. 2023 Apr 15;76(8):1483-91.

This paper was not included within our prior search date cut-off but is now included in after the updated search, with findings reported in the main manuscript.

Ambadekar NN, Zodpey SP, Soni RN, Lanjewar SP. Treatment outcome and its attributes in TB-HIV co-infected patients registered under Revised National TB Control Program: a retrospective cohort analysis. Public Health. 2015;129(6):783-789. doi:10.1016/j.puhe.2015.03.006

This paper has now been included in the review, with findings reported in the main manuscript.

Madan C, Chopra KK, Satyanarayana S, et al. Developing a model to predict unfavourable treatment outcomes in patients with tuberculosis and human immunodeficiency virus co-infection in Delhi, India. PLOS ONE. 2018;13(10):e0204982. doi:10.1371/journal.pone.0204982

This paper has now been included in the review, with findings reported in the main manuscript.

Saini S, Singh M, Garg A. A retrospective cohort study of treatment outcome among HIV positive and HIV negative TB patients in Chandigarh, India. Indian Journal of Community Health. 2016;28(2):145-150.

This paper was included in the review and continues to be included; however, it is not cited in the main manuscript as it does not include relevant adjusted analyses (unadjusted findings are reported in Table D in the S4 Appendix).

Gharat V, Modi BP, Patel P, Nayak S, Pawar A, Bansal RK. A Study on Socio-demographic Profile and Outcome of Tuberculosis in HIV-TB Co-infected Cases in Surat City, Western India. 2017;8(12):4.

This paper was not included in the review because, while it reported treatment outcomes, it did not report findings regarding factors associated with unfavorable TB treatment outcomes. It therefore did not meet inclusion criteria.

Narayanan S, Swaminathan S, Supply P, et al. Impact of HIV infection on the recurrence of tuberculosis in South India. J Infect Dis. 2010;201(5):691-703. doi:10.1086/650528

This paper did not meet criteria for inclusion in the review because it was evaluating whether TB recurrence was due to exogenous reinfection versus relapse. It therefore was not relevant to the study question.

Jenks JD, Kumarasamy N, Ezhilarasi C, Poongulali S, Ambrose P, Yepthomi T, Devaraj C, Benson CA. Improved tuberculosis outcomes with daily vs. intermittent rifabutin in HIV-TB coinfected patients in India. The International Journal of Tuberculosis and Lung Disease. 2016 Sep 1;20(9):1181-4.

This paper did not meet inclusion criteria for the review.

*Additionally, given the deep thought these authors have put into the gaps in care, they should consider developing a conceptual framework and presenting it in a unifying figure to ease dissemination of their hard work.*

We greatly appreciate this feedback. We considered various approaches to summarizing findings from this large review in a manner that would be helpful to readers. Based on feedback from the editors and reviewers, we moved two tables that summarized the numbers of studies reporting findings for specific exposures to the S6 Appendix, as it was not an optimal summary.

In place of these tables, based in feedback from Reviewer 3, we have added a new figure (Fig 12) to the Discussion section which qualitatively summarizes findings across both types of analyses (i.e., “factors” and “reasons”), which we feel will be more helpful to readers. While this is not a “conceptual framework,” we feel that it at least serves as a unifying figure summarizing findings to ease dissemination, as suggested by the reviewer. More in-depth integration of the diverse array of findings in this review may require future analysis papers. We thank the reviewer for this feedback, which has greatly improved communication of our study findings.

**Reviewer #4:**

**General comments**

*This in many ways is a heroic piece of work, bringing together a huge array of literature across 5 major steps in the TB care cascade, focused on risk factors and explanations.
It suffers from its own complexity and space limitations inherent in journal requirements for original papers.*

We appreciate both the positive comments and the challenges raised by the reviewer. We conducted this admittedly ambitious project with the goal of providing comprehensive insights into barriers across the TB care cascade in a manner that may be useful to TB program managers, researchers, and funders. We believe that the common patterns across this large array of studies may be helpful to inform future care delivery interventions to address India’s TB epidemic, which is the largest in the world accounting for nearly one-third of global TB deaths. While the manuscript is comprehensive, we believe that there is considerable benefit to presenting it as a single manuscript, as there are unique understandings that emerged from looking at common findings across gaps. In addition, the scientific approach to this systematic review was similar across gaps.

In our revision, we have taken seriously the feedback from the reviewer regarding the complexity of the manuscript, and we have made efforts to streamline the manuscript and the reporting of the findings, including: (1) reducing the word count for the Introduction, Methods, Results, and Discussion to reduce the overall manuscript length by 2000 words, (2) revising our presentation of Gap 4 considerably to combine populations of people with new TB, previously treated TB, or both in a manner that simplifies communication of common findings for people with drug-susceptible TB, and (3) revising all of the figures to ensure clarity of presentation as discussed further below.

We have also tried to align the main manuscript and supplementary information in a manner that might make all findings accessible to readers (e.g., Gap 1 aligns with the S1 Appendix, Gap 2 aligns with the S2 Appendix, etc.). Readers can then easily access the information for specific gaps that they are interested in.

*It is unclear how quality of studies was really handled and the level of standardisation of results summaries that is realistic or possible.*

Our quality or risk of bias criteria were developed and applied to a prior systematic review of the TB cascade of care in India (Subbaraman et al. PLoS Med 13(10): e1002149), because of variable guidelines for assessing the quality of these observational studies and the variability in study approaches across gaps. In response to the reviewer’s feedaback, we communicate the rationale and needs for slightly different criteria for the different gaps in the revised Methods section:

“In our prior review, we developed quality criteria relevant to studies of unfavorable outcomes in India’s TB care cascade [5], because of variable guidelines for assessing the quality of these observational studies and the variability in study approaches across gaps. For example, Gap 1 studies involved population-based screening to identify people with TB symptoms (with risks of bias including suboptimal sampling and survey response rates); Gaps 2 and 3 involved follow-up of people not yet engaged in TB care (with risks including suboptimal tracking approaches); while Gap 4 and 5 studies involved cohort or case-control designs. For the current review, we used quality criteria similar to those in the prior review, because these criteria describe methodological rigor and risk of bias (Table B in each of the S1-S5 Appendices).” (Methods section, subsection on Quality assessement,” first paragraph).

It is important to provide readers with insights into the quality or risk of bias of studies in a review, and with insights into what quality or risk of bias measured may not have been reported or addressed in included studies. A separate question is how the quality or risk of bias of studies should influence the reporting of findings in systematic reviews. In systematic reviews of drug trials, for example, in which randomized trials are overwhelmingly used to test drug effects, there are clearer approaches to stratifying by study quality or risk of bias.

Our review is different, in that we are looking at “real world” observational studies and trying to understand barriers to care in a manner that contributes to implementation science. For example, it is unclear how some risk of bias metrics might influence our analyses of “reasons” for falling out of care reported by people who experienced unfavorable outcomes. For this reason, we thought it was critical to provide readers with insights into the quality of studies, even as we report findings across studies unless they involved convenience sampling, which would lead to concerns about how representative their sample was of an underlying population.

*Formatting on figures in particular is inconsistent and the some are not ideal for the type of data being presented.*

We appreciate this feedback and have now revised all the figures in the paper to standardize their presentation in the following ways:

(1) For the figures presenting adjusted findings from regression analyses (e.g., odds ratios, risk ratios, or hazard ratios), we have ensured that there is a central line at “1” and that a “0” is provided on plots to ensure the directionality of findings is clear.
(2) For the “reasons” why people were lost at specific care cascade steps, we have ensured that all x-axes provide a full range from 0 to 100 (a standardized x-axis) and put a “%” sign after every x-axis value so that readers are clear that these findings represent percentages or proportions.
(3) Reporting of all study names, study locations, and footnotes to provide additional information on outcomes or types of effect estimates are now standardized, as are the figure captions.

*Some tables don't work very well as ways to present the data they represent and some integrated tables seem only to be presented in the discussion, when perhaps they should be more prominent in the results.*

The tables in the Methods section of the manuscript help to provide readers with information on the definitions, populations, and outcomes for each care cascade gap, as well as the framework we use to organize and report study findings. The study findings are largely communicated through figures, and we have now standardized the presentation of these tables as recommended by the reviewer.

With regard to the two tables that summarized study findings across gaps (formerly Tables 4 and 5, now Tables B and C in the S6 Appendix in the revised manuscript), we agree that these provided an important summary of findings across care cascade gaps. We have moved reference to these tables to the Results section, though the actual tables are now in the S6 Appendix.

Although these summary tables have been moved to the supplementary appendix, we have created a new figure (Figure 12) that is now located in the Discussion section, as it presents a qualitative summary of findings across care cascade gaps. We believe that this is a more reader-friendly presentation of high-level findings, and it allows readers to compare findings across gaps.

*The results section is very wordy and too conversational and too repetitive of the figures/tables.*

We appreciate this feedback and have reduced the word count wherever possible for the Results section, such that the word count for that section alone is reduce by nearly 600 words, despite the inclusion of findings from 47 new studies in the review.

*The discussion lacks really clear guidance for interventions that realistically could be implemented or for future research.*

We did provide some guidance on how this information could be used to inform interventions in the Discussion section. First, we provide guidance on specific subpopulations that should be a focus of efforts to reduce losses of people in the care cascade. These include men, older individuals, and, importantly, people with previous TB, especially those who were lost to follow-up from their prior treatment. Most importantly, we highlighted that people with early or minimal symptoms are much harder to retain in care. Understanding these risk factors can facilitate targeting of interventions and support. Second, we highlight an array of barriers that should be addressed by TB programs, which include work constraints and transportation barriers to clinics. The solutions to these problems may vary widely (e.g., disability insurance linked to TB, laws against being fired from work for TB, taking care to peoples’ homes or providing transportation or transportation vouchers). As such, we did not want to be too speculative about interventions in the discussion, as these may vary based on local context. Third, we point out several conditions that can be addressed directly in clinical care, including provision of counseling to improve TB knowledge, provision of treatment for unhealthy alcohol use or smoking cessation, and nutritional support. These are just some of the implications for interventions discussed in the Discussion.

*A major weakness of a cascade approach is the lack of focus on time delays - this is not dealt with adequately.*

We agree that care cascades evaluate losses from stages of care, rather than delays in completing stages of care, which are also important to inform public health interventions to address the TB epidemic. However, systematic reviews of TB “delay” studies in India have been performed previously (see for example, Sreeramareddy et al. Int J TB Lung Dis 2014;18(3):255-266), so insights regarding factors associated with TB delays in India are already available in the literature.

In addition, delay studies have some notable limitations. First, delay studies reflect samples of people who are interviewed regarding their delays after they have reached TB care and started TB treatment. As such, they represent a biased sample of people who do (eventually) reach subsequent care stages and provide limited insights regarding people who do not reach subsequent stages of care. Second, delay studies do not provide insights into on-treatment and post-treatment outcomes, because they usually interview people regarding their delays at the time of treatment initiation.

As such, our manuscript builds upon previous reviews of delays while providing new insights into losses across stages of care.

In response to the reviewer’s feedback, we have acknowledged lack of inclusion of delay studies as a potential limitation of our review in the Discussion section, paragraph before the Conclusion section:

“Finally, our approach to Gap 1 focused on care-seeking by people with TB symptoms; however, studies of TB diagnostic delays [169] and healthcare provider behavior using standardized patients [180–183] both suggest that health system barriers, especially poor quality of care, contribute substantially to Gap 1 losses. People developing interventions for Gap 1 should read these studies in parallel with our review findings.”

*I think this body of work is best as a very large report, such as a WHO resource document report, and it is not straightforward after that to work out how to crystallise into a journal publication, unless there is scope to include more figures and tables in the main manuscript (as opposed to appendices).*

We should note that a scientific process was used to conduct this review, including pre-registration in PROSPERO and following of the PRISMA guidelines for systematic reviews. As such, we do feel that this represents original scientific research meriting consideration by a peer reviewed journal. In addition, we have put in numerous efforts, as described above to streamline communication and make the manuscript accessible for readers, even as we acknowledge that this study contains considerable information.

*Alternatively, fewer tables and figures and a focus on tables 4 and 5 might be an option. Some, non-exhaustive points on the manuscript are covered below.*

As previously noted, we have kept Tables 4 and 5 (now Tables B and C in the S6 Appendix), but moved them to the supplemental materials as recommended by the editors. In the place of these tables, we have include a new Figure 12 that we believe communicates high-level findings of the review in a more accessible way.

**Introduction**

*This is quite well written and concise.*

We appreciate this feedback and note that the Introduction has been made even more concise in the revised manuscript.

**Methods**

*It is not clear, for those unfamiliar with the PECO framework, what it actually is and how it is being applied in this study.*

The PECO framework is very similar to the PICO framework (population / intervention / comparison / outcome), which is a widely used starting point for defining systematic reviews of interventions. In this review, we extracted findings regarding exposures (rather than interventions) from the included papers. As such, we felt that it was more appropriate to use the PECO framework (populations / exposures / comparisons / outcomes); however, PECO is simply a variation of the PICO framework. We should note that both PICO and PECO are widely used.

For individuals unfamiliar with the PECO framework, we have now referenced a paper (citation [16] in the main manuscript) that describes this framework in the Methods section, sub-section entitled “PECO framework,” paragraph 1, sentence 1:

“Morgan RL, Whaley P, Thayer KA, Schünemann HJ. Identifying the PECO: A framework for formulating good questions to explore the association of environmental and other exposures with health outcomes. Environ Int. 2018;121: 1027–1031. doi:10.1016/j.envint.2018.07.015”

*It is confusing as to whether the search included only studies on India or whether it included studies from other countries.*

Search terms are included in Table A in each of the S1-S5 Appendices. Notably, the umbrella term “India” and several variations of this terms are included in the table for all of the searches.

*The messaging around quality is confusing - lines 260-65.  For example: how could there be a commitment to quality at the same time as there is a commitment only to exclude studies that used convenience sampling?*

It is important to provide readers with insights into the quality or risk of bias of studies in a review, and with insights into what quality or risk of bias measured may not have been reported or addressed in included studies. A separate question is how the quality or risk of bias of studies should influence the reporting of findings in systematic reviews. In systematic reviews of drug trials, for example, in which randomized trials are overwhelmingly used to test drug effects, there are clearer approaches to stratifying by study quality or risk of bias.

Our review is different, in that we are looking at “real world” observational studies and trying to understand barriers to care in a manner that contributes to implementation science. For example, it is unclear how some risk of bias metrics might influence our analyses of “reasons” for falling out of care reported by people who experienced unfavorable outcomes. For this reason, we thought it was critical to provide readers with insights into the quality of studies, even as we report findings across studies unless they involved convenience sampling, which would lead to concerns about how representative their sample was of the underlying population.

*It is not clear how the social-ecological framework was applied or what it is.
Table 2 looks like it would be better as an appendix*

Thank you for this feedback, which highlights the need to provide more information on this framework: We have provided additional explanation and citations related to the social-ecological framework in the Method section, subsection entitled “Framework for organizing and visualizing findings,” paragraph 1, first sentence:

“Informed by the social-ecological model [21,22]—which looks at the interplay of risk factors at the individual, family, community, and society levels—we organized findings into “person-, family-, or society-related factors” and “health system factors,” with subcategories, to inform future interventions (Table 2).”

One citation refers to Bronfenbrenner’s original articulation of the social-ecological model, and one is a reference to this model’s use in infectious disease research:

Bronfenbrenner U. Ecological systems theory. Six theories of child development: Revised formulations and current issues. Jessica Kingsley Publishers; 1992. pp. 187–249.

Baral S, Logie CH, Grosso A, Wirtz AL, Beyrer C. Modified social ecological model: a tool to guide the assessment of the risks and risk contexts of HIV epidemics. BMC Public Health. 2013;13: 482. doi:10.1186/1471-2458-13-482

We feel that Table 2 is very important to keep within the main manuscript, as it provides readers with clarity regarding how we are organizing, reporting, and visualizing findings for all Forest plots.

**Results**

*Table 3 is cumbersome with too many NA results.*

We feel that Table 3 provides readers with a snapshot of the overall number, characteristics, and quality of studies in the review. Given that this review spanned multiple care cascade gaps, which involved different types of studies, quality and risk of bias criteria also varied. As such, some quality or risk of bias criteria are not relevant to certain care cascade gaps in the review.

We appreciate the concern about many cells being population with “NA”. In response to the concern over “NA” (i.e., not applicable) cells in this table, we have now specified which gap each quality or risk of bias criterion pertains to. For example, we now have changed the text within column 1 (row 14) to state: “Studies that were medium or low quality for the proportion of the estimated population screened for TB symptoms (Gap 1 only).”

By stating that this criterion is only relevant to Gap 1 (as Gap 1 involved cross-sectional, population-based studies), this helps to clarify why the other columns for the other gaps are denoted as “NA.”

*Also, the subheading of 'type of study design' does not seem to relate to the two lines below - ie. these are not study designs. It is also not clear whether the settings are mutually exclusive or not.*

For Table 3, we agree that the column subheading did not appropriately refer to types of study designs. We have instead modified this subheading to stay: “Studies contributing to findings reported in the main manuscript.” We have also modified the description of the study findings underneath to provide information regarding how these are reported, as followed: “Studies reporting factors associated with unfavorable outcomes from adjusted (i.e., multivariable) regression analyses” and “Studies reporting reasons from surveys of people who experienced unfavorable outcomes.” We hope this revised language provides clarity for the two different types of Forest plots readers will see in the Results section.

Under study setting, we have now tried to clarify that the urban, rural, or both study setting rows are mutually exclusive by revising these to: “Studies conducted in a rural setting only,” “Studies conducted in an urban setting only,” and “Studies conducted in both rural and urban settings.” By using the words “only” in the first two, this clarifies that studies must mutually exclusively fall into one of these rows.

We appreciate this feedback from the reviewer, which helps us to provide greater clarity to readers.

*The language speaking to the Gaps in relation to the figures and tables is too loose and should be tightened up and be more formal. It is too conversational and tends to 'cherry pick'.*

We have tried to tighten up the language in the Results section considerably, by cutting words when possible. We have also tried to provide more comprehensive insights into the systematic review findings, in a manner that also builds upon feedback from Reviewers 1 and 2.

Specifically, for any variables with statistically significant findings in any adjusted analyses, we have now also reported all studies that provide non-significant findings for those same variables in any adjusted analyses in the Forest plot captions and in the main text of the Results section. This has resulted in changes throughout the Results section and in the captions of nearly all Forest plots reporting findings from regression analyses. Please note that, for a few Forest plots, especially in Gaps 2 or 3, there were no studies reporting non-significant findings for variables included in the Forest plot, so these additions have not been made to these Forest plot captions. The inclusion of non-significant findings provides readers with the totality of the evidence for relevant variables.

We hope this addresses any concerns the reviewer may have about “cherry-picking” evidence, and we thank them for this critical feedback.

*The x axis scale of figure 1 is problematic, including the arrows on the graph. Font size across x axes on the graphs is inconsistent too.*

We appreciate this feedback and have now revised all the figures in the paper to standardize their presentation in the following ways:

(1) For the figures presenting adjusted findings from regression analyses (e.g., odds ratios, risk ratios, or hazard ratios), we have ensured that there is a central line at “1” and that a “0” is provided on plots to ensure the directionality of findings is clear.
(2) For the “reasons” why people were lost at specific care cascade steps, we have ensured that all x-axes provide a full range from 0 to 100 (a standardized x-axis) and put a “%” sign after every x-axis value so that readers are clear that these findings represent percentages or proportions.
(3) Reporting of all study names, study locations, and footnotes to provide additional information on outcomes or types of effect estimates are now standardized, as are the figure captions.

*Figure 2 which reports percentages and confidence intervals does not need to be in the format that one would expect when comparing odds ratios. It could be a line graph or similar. It is also not clear how consistent the definitions for eg 'a lack of…' were across studies. This is true for other similar subsequent figures - and I note that these are not formatted consistently.*

As noted above, for these Forest plots reporting percentages, we have now used a uniform x-axis for all of these figures, ranging from 0 to 100. We appreciate the feedback from the reviewer, which has helped us to improve presentation of all figures.

We feel that there is benefit to presenting these findings regarding percentages in a Forest plot, as it allows readers to quickly visualize findings that affect a large percentage of people across studies, versus those that affect a relatively small percentage of people. For example, in Figure 2, readers can quickly visualize that “work pressures” and “lack of symptom severity” are reasons for lack of care seeking reported by a relatively larger percentage of people across studies, while alcohol use is reported by a small percentage of people.

Regarding definitions for each care cascade gap, we have been very careful to: (1) provide the outcomes used in each paper as footnotes—e.g., for Gap 4, different types of unfavorable outcomes are denoted in footnotes to each paper; (2) compare similar unfavorable outcomes in different gaps in the same plots or sections—e.g., for Gap 2, we divide up findings by non-completion of different types of test modalities (e.g., X-ray vs. sputum microscopy vs. nucleic acid amplification testing); and (3) to provide detailed definitions in Table 1 and in each of the S1-S5 Appendices.

*It is not clear why some gaps have no associated figures in the main body of the paper. If this is because of restrictions on figures, one would be inclined to revisit how the data across the gaps are presented, such as by using Figure 2A to F for example.*

We have included figures that are more “data-rich” in the main manuscript, as these figures contain more information and are more valuable to readers. We have placed figures that contain less data in the S6 Appendix, and we have ensured that these are organized in an accessible manner with a table of contents in the S6 Appendix that allows readers to quickly access relevant figures. The rationale for including some figures in the S6 Appendix is that these figures contain few findings, and, for that reason, they findings and trends in the findings can be more easily summarized in the text.

*Figure 5 and 6: It seems unnecessary to make these two distinct figures - perhaps figures A and B would be better? Similar issues arise when looking at the remaining figures.*

We appreciate this feedback from the reviewer, which is concordant with feedback from Reviewer 2. We have taken the reviewer’s advice to combine these populations of people with drug-susceptible TB. Particularly after our updates of the search to August 2023 resulted in the inclusion of about 40 additional studies in Gap 4, we realized that combining the analyses for these 3 populations (people with new TB, people with previously treated TB, and people with either new or previously treated TB) results in greater clarity and impact for the reader. All 3 of these populations are now collectively referred to as “people with drug-susceptible TB,” but we have still been very careful to designate findings from each specific population using footnotes to the study names in the Forest plots.

This feedback resulted in substantial reorganization of the Gap 4 Results section. Given the large volume of findings across these 3 subpopulations of people with drug-susceptible TB, we have now divided reporting of Results for this population into the following sub-sections: (1) “Demographic factors contributing to unfavorable treatment outcomes in people with drug-susceptible TB”; (2) “Clinical factors contributing to unfavorable treatment outcomes in people with drug-susceptible TB”; (3) “Socioeconomic, psychosocial, and family- or society-related barriers contributing to unfavorable treatment outcomes in people with drug-susceptible TB”; (4) “Health system barriers contributing to unfavorable treatment outcomes in people with drug-susceptible TB.”

We thank the reviewer for this feedback, which has greatly improved the clarity and impact of the presentation of findings for people with drug-susceptible TB.

*An issue is that the presentation of the data by cascade steps does not make it easy to see commonalities in factors between steps, although tables 4 and 5 go some way towards addressing this, although it seems only introduced in the discussion and not in the results.*

We agree that helping readers to understand commonalities in risk factors across care cascade stages is an important contribution of this paper, and it is important to present this with clarity. We have moved Tables 4 and 5 to reference them in the Results section, but these are now placed at the end of the S6 Appendix (cited as Tables B and C in that appendix). This ensures that readers will be able to understand common findings across care cascade gaps.

At the same time, given that these tables have been moved to the supplementary appendix, we have created a new figure (Fig 12) that qualitatively summarizes important findings across care cascade gaps—both common findings across gaps and unique but important findings within gaps. This figure is placed at the end of the Results section and more accessibly communicates the high-level finding of the review.

*Also, time delays are not considered, which is a problem with cascade analyses.*

We agree that care cascades evaluate losses from stages of care, rather than delays in completing stages of care, which are also important to inform public health interventions to address the TB epidemic. However, systematic reviews of TB “delay” studies in India have been performed previously (see for example, Sreeramareddy et al. Int J TB Lung Dis 2014;18(3):255-266), so insights regarding factors associated with TB delays in India are already available in the literature.

In addition, delay studies have some notable limitations. First, delay studies reflect samples of people who are interviewed regarding their delays after they have reached TB care and started TB treatment. As such, they represent a biased sample of people who do (eventually) reach subsequent care stages and provide limited insights regarding people who do not reach subsequent stages of care. Second, delay studies usually do not provide insights into on-treatment and post-treatment outcomes, because they usually interview people regarding their delays at the time of treatment initiation.

As such, our manuscript builds upon previous reviews of delays while providing new insights into losses across stages of care.

In response to the reviewer’s feedback, we have acknowledged lack of inclusion of delay studies as a potential limitation of our review in the Discussion section, paragraph before the Conclusion section:

“Finally, our approach to Gap 1 focused on care-seeking by people with TB symptoms; however, studies of TB diagnostic delays [169] and healthcare provider behavior using standardized patients [180–183] both suggest that health system barriers, especially poor quality of care, contribute substantially to Gap 1 losses. People developing interventions for Gap 1 should read these studies in parallel with our review findings.”

**Discussion**

*This is generally reasonably well written, but as noted above it should not be the place where results tables are described for the first time. It would be reasonable to have a table, however, on possible policy changes and options for improvement.*

We have moved Tables 4 and 5 to reference them in the Results section, but these are now placed at the end of the S6 Appendix (now cited as Tables B and C in that appendix). This ensures that readers will be able to understand common findings across care cascade gaps.

At the same time, given that these tables have been moved to the supplementary appendix, we have created a new figure (Fig 12) that qualitatively summarizes important findings across care cascade gaps—both common findings across gaps and unique but important findings within gaps.

*I am not sure I agree that the following conclusion is supported by the study: "while quality of care is better in the government TB program, client experience is generally better in the private sector."*

We have deleted this sentence.
