# Supplementary material for: Barriers to engagement in the care cascade for tuberculosis disease in India: A systematic review of quantitative studies
Source: PLoS Med. 2024 May 28;21(5):e1004409. doi: 10.1371/journal.pmed.1004409 (PMC11166313; doi:10.1371/journal.pmed.1004409)
Supplement: Attachment — Submitted filename: Response to Reviewers comments PMED 22 April 2024.docx [file pmed.1004409.s011.docx]

**RESPONSES TO THE EDITORS**:

**General comments**

*1) Please include page numbers and line numbers in the manuscript file. Use continuous line numbers (do not restart the numbering on each page). For review purposes, we have added line numbers to the file “Main manuscript - Barriers to TB care cascade - Clean.docx” (attached).*

We have included page numbers and line numbers in the manuscript file, as suggested.

*2) We note that you have switched between the terms gender and sex throughout the manuscript. Please note that the terms gender and sex are not interchangeable (as discussed in* [*https://www.who.int/health-topics/gender*](https://www.who.int/health-topics/gender)*); please use the appropriate term (we suggest "sex").*

We have replaced “gender” with “sex” in Table 2, row 1. This was the sole place where the terms were interchanged, and we appreciate you letting us know.

**Abstract**

*l.2: Please define “TB” at first use.*

We defined TB at first use.

**Author Summary**

*l.60: Please exchange “barriers” with “factors”.*

We exchanged “barriers” with “factors”.

**Methods and Results**

*1) In line with Reviewer #2's comments and after discussion with the Academic Editor, we ask you to include the nonsignificant results in the figures instead of showing only the significant results. You may add a footnote to the relevant results to indicate that these are non-significant results.*

We appreciate this feedback and sought further clarification by email with Dr. Alexandra Schaefer on the PLOS Medicine editorial team. Dr. Schaefer responded as follows:

“We have decided that the inclusion of nonsignificant results will not be a mandatory editorial request. We feel that the figure description transparently mentions the studies (and factors) that contain non-significant findings, and that only the significant findings are presented in the graphs. However, we would encourage you to consider including the nonsignificant findings in the forest plots, as this is considered best practice.”

After extensive discussion within our team, we ultimately deferred inclusion of the non-significant findings, because we were concerned regarding the clarity and accessibility of findings for readers. For example, the forest plots that currently have the most data (Figs. 5 and 9 in the main manuscript and Figs. I and J in the S6 Appendix) will have an additional 33% to 75% increase in findings presented in the plots with inclusion of non-significant findings. We anticipated that this would have meant that more plots will be moved to the supplementary appendix, which means that readers will not be able to visualize them in the main manuscript. In addition, currently the readers can access these non-significant findings based on citation by looking in the S1-S5 appendices (Table D in each), where all findings are reported. Hence, we have opted to not include these nonsignificant findings in forest plots and have acknowledged non-inclusion of these findings as a limitation in the Discussion, section on “Strengths and limitations of the review,” paragraph 2:

“Another limitation in visualization of findings is that we did not include statistically non-significant findings in our Forest plots due to space constraints; however, these non-significant estimates are available for readers in Table D in each of the S1-S5 appendices. Any future meta-analyses conducted using our review findings should ensure inclusion of relevant non-significant estimates that we reported.”

*2) ll.147-162: Under "Search strategy and study selection", please include the initials of the two reviewers who assessed the articles for eligibility. Please also include the initials under "Data extraction".*

We had a total of 5 reviewers who assessed the articles for eligibility and extracted data across the various gaps and searches. We have included the initials of all 5 reviewers in parentheses.

*3) We have noted that throughout the main text you use vague descriptions, such as “half of people”; “one-third of people” (l.287/l.289), which we feel will be difficult for the reader to imagine what these descriptions mean in terms of actual numbers. Could you provide actual event numbers in brackets?

The same goes for Figures 2, 4, and 6. The percentages give little insight into the magnitude of the numbers presented (2% of 1000 people or 10000 makes a big difference). Therefore, we suggest adding the actual numbers (nominator/denominator). Please revise throughout the main manuscript.*

Based on feedback, we have revised several figures to ensure inclusion of appropriate numerators and denominators in Forest plots of “reasons” for unfavorable care cascade outcomes. Specifically, Figs 2, 4, 6, and 8 in the main manuscript have been revised, as have Figs C, E, G, H, L and M in the S6 appendix. In each Forest plot, we have now reported the numerator/denominator, percentage, and 95% confidence interval for each finding. We also made appropriate changes throughout the text of the main manuscript to report numerator/denominator for these findings where appropriate, though in a very few instances the text makes reference to general trends across multiple findings and it was not appropriate to report every numerator/denominator.

*4) l.318 ff: "(reported by 15% in 1 study [42])" (example) - Similar to the comment above, we suggest reporting the nominator and denominator. Please revise once again throughout the main manuscript.*

We made appropriate changes throughout the text of the main manuscript to report numerator/denominator for these findings where appropriate, though in a very few instances the text makes reference to general trends across multiple findings and it was not appropriate to report every numerator/denominator.

*5) Figure 6: Please define “DOT/DOTS”.*

We defined DOTS (directly observed therapy short course) as suggested in the figure caption (“DOT” specifically is not included in the figure).

**Discussion**

*1) l.870: Please temper claims of primacy of results by stating, "to our knowledge" or something similar.*

We were unable to find where these claims were made in our manuscript, despite a thorough review. Can the Editor help us point out to the specific line number, so that we can address it accordingly?

*2) Please remove the "Conclusion" subheading. The Conclusion paragraph should be a continuous part of the Discussion section.*

We removed the “Conclusion” subheading, as suggested.

**References**

*1) Where website addresses are cited, please specify the date of access and use the word “accessed” instead of “cited” (e.g. [accessed: 12/06/2023]).*

We made these changes, as suggested.

**Social Media**

*To help us extend the reach of your research, please provide any X (formerly known as Twitter) handle(s) that would be appropriate to tag, including your own, your co-authors’, your institution, funder, or lab. Please enter in the submission form any handles you wish to be included when we post about this paper.*

- @TJhaveriMD
- @jhaveri_disha
- @atgalivanche
- @mayalubecks
- @DominicVoehler
- @ruvandhi
- @HShewade
- @JessicaHaberer
- @paimadhu

**Comments from Reviewers**

**Reviewer #1:** *The authors have addressed all my points including the important one about non-statistically-significant effects.

Michael Dewey*

Thank you.

**Reviewer #2:** *It was a pleasure to read the updated version of this article. It has been greatly improved by the edits made, and strikes a balance between comprehensively and consistently reporting the entirety of the work done, while also providing a clear synthesis of the findings.*

Thank you.

*My only remaining comment: while I appreciate the inclusion of the non-significant findings (also requested by Reviewer 1), I am not sure why it's not possible to also include these on the forest plots themselves (as opposed to just in the captions), to more accurately display the entirety of the data. I can see that this might be crowded but I worry that the current presentation may be misleading. To pick one example, I find it interesting that in most studies for Gap 4, educational attainment was not significantly associated with worse outcomes - not the impression given by Figure 5. Is this due to wide confidence intervals (suggesting that people with less education are indeed at consistently higher risk of LTFU and may need more tailored support) or because there was truly no effect (suggesting that in most settings education does not act as an obstacle to completing TB treatment)? This seems like exactly the kind of heterogeneity a forest plot is designed to show. This is a suggestion, and I defer to the statistical reviewer (Reviewer 1), editors and authors on this point.*

We acknowledge and understand your viewpoint regarding the inclusion of the nonsignificant findings in the forest plots, which would be considered best practice.

However, after extensive discussion within our team, we ultimately deferred inclusion of the non-significant findings, because we were concerned regarding the clarity and accessibility of findings for readers. For example, the forest plots that currently have the most data (Figs. 5 and 9 in the main manuscript and Figs. I and J in the S6 Appendix) will have an additional 33% to 75% increase in findings presented in the plots with inclusion of non-significant findings. We anticipated that this would have meant that more plots will be moved to the supplementary appendix, which means that readers will not be able to visualize them in the main manuscript. In addition, currently the readers can access these non-significant findings based on citation by looking in the S1-S5 appendices (Table D in each), where all findings are reported. Hence, we have opted to not include these nonsignificant findings in forest plots and have acknowledged non-inclusion of these findings as a limitation in the Discussion, section on “Strengths and limitations of the review,” paragraph 2:

“Another limitation in visualization of findings is that we did not include statistically non-significant findings in our Forest plots due to space constraints; however, these non-significant estimates are available for readers in Table D in each of the S1-S5 appendices. Any future meta-analyses conducted using our review findings should ensure inclusion of relevant non-significant estimates that we reported.”

*Otherwise many thanks for the opportunity to review an interesting piece of work.*

**Reviewer #4:** *The authors have addressed all my comments/concerns adequately*

Thank you.

Any attachments provided with reviews can be seen via the following link:
<https://www.editorialmanager.com/pmedicine/l.asp?i=898027&l=K6WGHYVH>

**General Editorial Requests**

*1) We ask every co-author listed on the manuscript to fill in a contributing author statement. If any of the co-authors have not filled in the statement, we will remind them to do so when the paper is revised. If all statements are not completed in a timely fashion this could hold up the re-review process. Should there be a problem getting one of your co-authors to fill in a statement we will be in contact. YOU MUST NOT ADD OR REMOVE AUTHORS UNLESS YOU HAVE ALERTED THE EDITOR HANDLING THE MANUSCRIPT TO THE CHANGE AND THEY SPECIFICALLY HAVE AGREED TO IT.*

We have not changed author information.

*2) Please ensure that the paper adheres to the PLOS Data Availability Policy (see* [*http://journals.plos.org/plosmedicine/s/data-availability*](http://journals.plos.org/plosmedicine/s/data-availability)*), which requires that all data underlying the study's findings be provided in a repository or as Supporting Information. For data residing with a third party, authors are required to provide instructions with contact information for obtaining the data. PLOS journals do not allow statements supported by "data not shown" or "unpublished results." For such statements, authors must provide supporting data or cite public sources that include it.*

This has been done.

*3) Please review your reference list to ensure that it is complete and correct. If you have cited papers that have been retracted, please include the rationale for doing so in the manuscript text, or remove these references and replace them with relevant current references. Any changes to the reference list should be mentioned in the rebuttal letter that accompanies your revised manuscript.*

This has been done.
